# Synthesis and In Vitro Anticancer Activity of Novel 4-Aryl-3-(4-methoxyphenyl)-1-phenyl-1*H*-pyrazolo[3,4-*b*]pyridines Arrest Cell Cycle and Induce Cell Apoptosis by Inhibiting CDK2 and/or CDK9

**DOI:** 10.3390/molecules28176428

**Published:** 2023-09-04

**Authors:** Basma S. Almansour, Faizah A. Binjubair, Alaa A.-M. Abdel-Aziz, Sara T. Al-Rashood

**Affiliations:** Department of Pharmaceutical Chemistry, College of Pharmacy, King Saud University, P.O. Box 2457, Riyadh 11451, Saudi Arabia; 443203434@student.ksu.edu.sa (B.S.A.); fbinjubair@ksu.edu.sa (F.A.B.); almoenes@ksu.edu.sa (A.A.-M.A.-A.)

**Keywords:** pyrazolo[3,4-*b*]pyridines, anti-cancer, cell cycle, apoptosis, CDK2, CDK9

## Abstract

Two series of pyrazolo[3,4-*b*]pyridine derivatives, **9a**–**h** and **14a**–**h**, are synthesized and evaluated for their anti-cancer potency towards Hela, MCF7, and HCT-116 cancer cell lines. Compound **9a** showed the highest anticancer activity with IC_50_ = 2.59 µM against Hela when compared with doxorubicin (IC_50_ = 2.35 µM). Compound **14g** revealed cytotoxicity IC_50_ = 4.66 and 1.98 µM towards MCF7 and HCT-116 compared to doxorubicin with IC_50_ = 4.57 and 2.11 µM, respectively. Compound **9a** exhibited cell cycle arrest at the S phase for Hela, whereas **14g** revealed an arresting cell cycle for MCF7 at G2/M phase and an arresting cell cycle at S phase in HCT-116. In addition, **9a** induced a significant level of early and late apoptosis in Hela when compared with the control cells, whereas **14g** induced an apoptosis in MCF7 and HCT-116, respectively. Compounds **9a** (IC_50_ = 26.44 ± 3.23 µM) and **14g** (IC_50_ = 21.81 ± 2.96 µM) showed good safety profiles on normal cell line WI-38. Compounds **9a** and **14g** showed good inhibition activity towards CDK2, with IC_50_ = 1.630 ± 0.009 and 0.460 ± 0.024 µM, respectively, when compared with ribociclib (IC_50_ = 0.068 ± 0.004). Furthermore, **9a** and **14g** showed inhibitory activity towards CDK9 with IC_50_ = 0.262 ± 0.013 and 0.801 ± 0.041 µM, respectively, related to IC_50_ of ribociclib = 0.050 ± 0.003. Docking study for **9a** and **14g** exhibited good fitting in the CDK2 and CDK9 active sites.

## 1. Introduction

**Figure 1:**
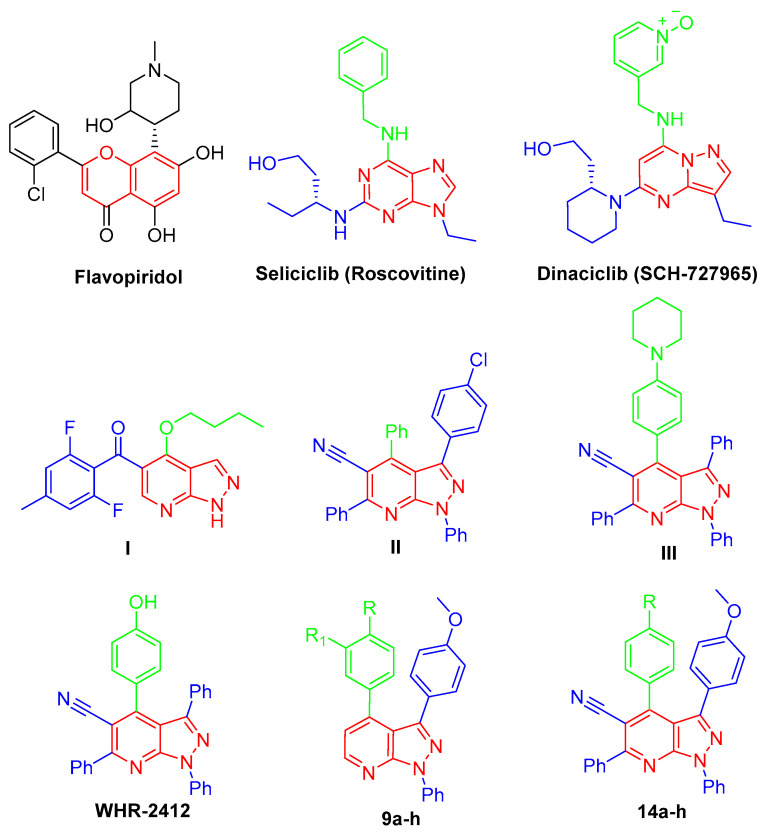
Chemical structures of flavopiridol, roscovitine, dinaciclib, pyrazolo[3,4-*b*]pyridines **I**–**III, WHR-2412**, and targeted compounds **9a**–**h** and **14a**–**h**.

Biomedical research has focused on how living cells grow and divide since the theory of spontaneous generation was disproved in the late seventeenth century, and the interest in the subject only increased as it became clear that sustained cellular proliferation was central to the initiation and progression of cancer, and today, sustained proliferative capacity is considered a hallmark of cancer [1]. Regulation of proliferation is defined by the work of Hartwell, Nurse, and Hunt, and they also defined the role of cyclin-dependent kinases (CDKs); hence, the 2001 Nobel Prize in Physiology and Medicine was awarded to them [2]. The cyclin-dependent protein kinases (CDKs) are protein-serine/threonine kinases that belong to the CMGC family (CDKs, mitogen-activated protein kinases, glycogen synthase kinases, and CDK-like kinases) [3]. The human genome encodes 21 CDKs, although only 7 (CDK 1, 2, 3, 4, 6, 10, 11) have a direct crucial role in the progression of the cell cycle, while other CDKs play an indirect role via activation of other CDKs (CDK 3), regulation of transcription (CDK7, 8, 9) or neuronal function (CDK 5). Different families of cyclins, the regulatory subunits required for CDK activity, have been identified, and their expression fluctuates significantly throughout the phases of the cell cycle [4]. CDK inhibitors may be used to treat cancer, diabetes, kidney disease, neurological illness, and infectious disease [5,6]. A variety of chemical classes, typically planar heteroaromatic structures, such as flavonoid, purine, indenopyrazole, arylcarbazole, indolinone, oxindole, pyrimidine, thiazole, and indirubin, have been described as CDK inhibitors [7]. CDKs have been considered promising targets for the treatment of cancers and other diseases due to their crucial roles in the regulation of cell cycle and transcription. Since the 1990s, the first-generation CDK inhibitors have been explored [8]. These molecules include benzopyran derivative flavopiridol [9,10], imidazolo [4,5-*d*]pyrimidine roscovitine [11,12], and pyrazolo [1,5-*a*]pyrimidine dinaciclib (SCH-727965) [13,14] (Figure 1).

In the last few decades, anticancer drugs have been developed from chemically synthesized compounds including pyrazole derivatives where their anticancer activity is due to the inhibition of various targets such as CDKs [15]. Important pyrazole-containing drugs were available in the market, such as Celecoxib, which showed very promising anticancer activity against prostate cancer cells [16,17]. On the other hand, pyridine derivatives serve as promising anticancer agents in the field; they acquired huge attention in current medicinal research due to their impact in curing numerous vicious ailments, such as breast cancer, myeloid leukemia, and idiopathic respiratory fibrosis [18,19]. Hybridization of pyrazole and pyridine scaffold is of great importance in cancer treatment, such as via sorafenib, regorafenib, vismodegib, and crizotinib. Pyrazolo[3,4-*b*]pyridine scaffold is an important central moiety of several anticancer agents, such as in the case of compounds **I**–**III** [20,21,22,23], which showed an interesting anti-cancer activity through different mechanisms, in addition to **WHR-2412**, which can exert its antitumor impact through CDK2 inhibition [24,25] (Figure 1).

There are three binding sites that have been reported in the CDK monomer: the ATP-competitive binding site (Site I) in addition to two non-competitive binding sites, II and III. Site I, the main target site of CDK2 inhibitors, has the impressive capacity to accommodate various ligands, including flat heterocyclic rings. On the other side, site III accommodates short peptide ligands, whereas the binding mechanism of Site II remains ambiguous. Furthermore, when CDK is subjected to the cyclin binding process, the resulting conformational changes give rise to a variation of the ATP binding site and then generate an allosteric binding site, i.e., Site IV, which requires the binding inhibitors to possess extraordinarily high inhibitory affinities. In precis, for designing potent CDK2 and CDK9 inhibitors, we can arrange the following principles: (1) developing inhibitors with flat heterocyclic rings for site I; (2) designing short peptide ligands for Site III; and (3) employing binding molecules which have extraordinarily high inhibitory affinities for Site IV [26,27].

**Figure 2:**
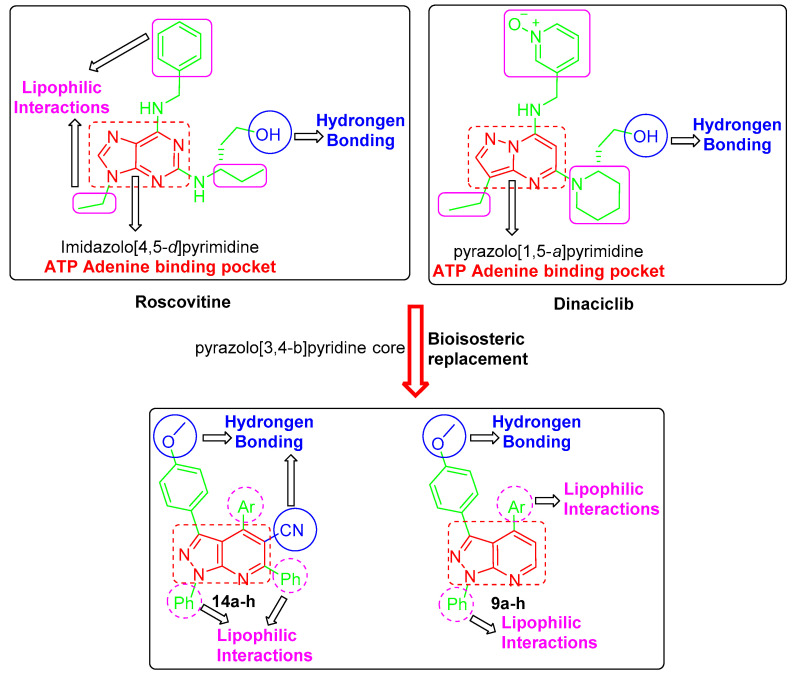
Design of the targeted pyrazolo[3,4-*b*]pyridine derivatives **9a**–**h** and **14a**–**h**.

In the light of the mentioned data, several drug design approaches were exploited in order to develop the target pyrazolo[3,4-*b*]pyridine derivatives, **9a**–**h** and **14a**–**h**, (Figure 2). First, the bioisosteric replacement strategy was utilized to replace the core heterocycle pyrazolo [1,5-*a*]pyrimidine in dinaciclib and imidazolo [4,5-*d*]pyrimidine core in roscovitine by the pyrazolo[3,4-*b*]pyridine core in the target structures. These fused heterocyclic are expected to be fitted into the ATP adenine binding pocket. Moreover, both roscovitine and dinaciclib are tethered with the hydroxyl functionality that is responsible for the hydrogen bonding. As a result, the methoxy functionality was grafted into the structure of our target compounds to ensure the requisite hydrogen bonding.

Furthermore, the cyano functional group was incorporated in target compounds **14a**–**h** to enhance the hydrogen bonding interactions (Figure 2). Moreover, the target structures were decorated with diverse lipophilic motifs to establish the required hydrophobic interactions within the CDK2 binding site. The variation of substitutions in **9a**–**h** and **14a**–**h** was adopted to provide different lipophilic environments (Figure 2). Interestingly, a pilot molecular modeling study showed a plausible binding mode and different interactions of the target molecules within their proposed target CDK2. As expected, a preliminary molecular modeling 

study showed that the designed pyrazolo[3,4-*b*]pyridine derivatives **9a**–**h** and **14a**–**h** succeed in achieving the required hydrogen bonding and hydrophobic interactions.

## 2. Results and Discussion

### 2.1. Chemistry

**Scheme 1:**
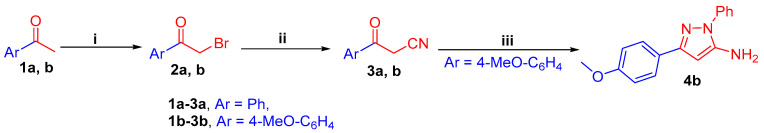
(**i**) Br_2_, AcOH, stirring, 1 h, rt; (**ii**) KCN, EtOH/H_2_O, stirring, 2 h, rt; (**iii**) PhNHNH_2_, EtOH, reflux, 6 h.

3-(Phenyl/4-methoxyphenyl)-3-oxopropanenitrile **3a, b**, are prepared via the bromination of acetophenones **1a**, **b** to afford 2-bromo-1-(phenyl/4-methoxyphenyl)ethan-1-ones **2a**, **b**, followed by cyanation reaction (Scheme 1). Then, the key intermediate 3-(4-methoxyphenyl)-pyrazol-5-amine derivative **4b** is synthesized through the reaction of oxopropanenitrile **3b** with phenylhydrazine in refluxing ethanol (Scheme 1).

**Scheme 2:**
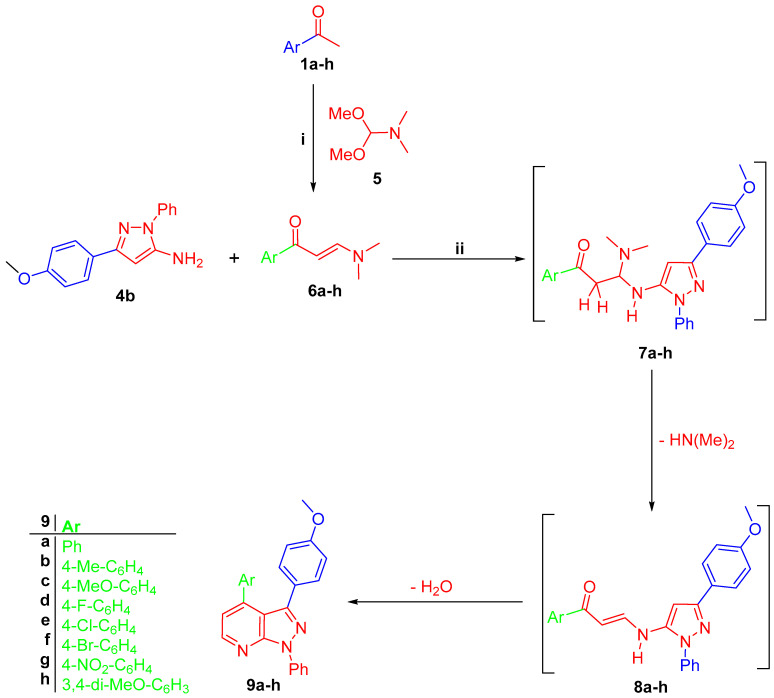
(**i**) DMF-DMA, anhydrous xylene, reflux 8 h; (**ii**) glacial AcOH, refluxed for 4–6 h.

Amino pyrazole derivative **4b** reacted with prop-2-en-1-ones **6a**–**h**, which was prepared by the reaction of acetophenones **1a, b** with dimethylformamide-dimethylacetal (DMF-DMA) **5** in anhydrous xylene, to give 3-(4-methoxyphenyl)-pyrazolo[3,4-*b*]pyridines **9a**–**h**, respectively (Scheme 2). The latter reaction is proposed to proceed through *michael* addition of exocyclic NH_2_ of amino pyrazole **4b** into the double bond of enaminones **6a**–**h** to form intermediate **7a**–**h**, which lost a dimethylamine molecule to give intermediate **7a**–**h**, which gave the final targeted compounds **9a**–**h** through an intramolecular cyclo-condensation reaction (Scheme 2).

The ^1^HNMR of **9a**–**h** revealed the doublet signals of pyridine H_2_ and H_3_ around *δ* 8.60 and 8.00 *ppm*, respectively, with coupling constant (*J*) around 6.5 Hz. ^1^HNMR of **9a**–**h** showed the singlet signal of the methoxy group of the pyrazole 4-methoxyphenyl group around *δ* 3.87 *ppm*, whereas compounds **9b** and **9c** revealed an additional signal of methyl and methoxy groups at *δ* 2.41 and 3.855 *ppm*, respectively. Moreover, the signals of two methoxy groups of **9h** appeared at *δ* 3.86 and 3.93 *ppm*, respectively. ^13^CNMR of **9a**–**h** showed the signals of pyrazole aryl –*C*H_3_O-C_6_H_4_ carbon at *δ* 55.75 *ppm,* in addition to the signals of SP^3^ carbons of pyridine aryl for –*C*H_3_-C_6_H_4_ in **9b**, –*C*H_3_O-C_6_H_4_ in **9c** and *para* and *meta*–*C*H_3_O-C_6_H_3_ in **9h** at *δ* 21.38, 55.79, 55.90, and 56.07 *ppm*, respectively.

**Scheme 3:**
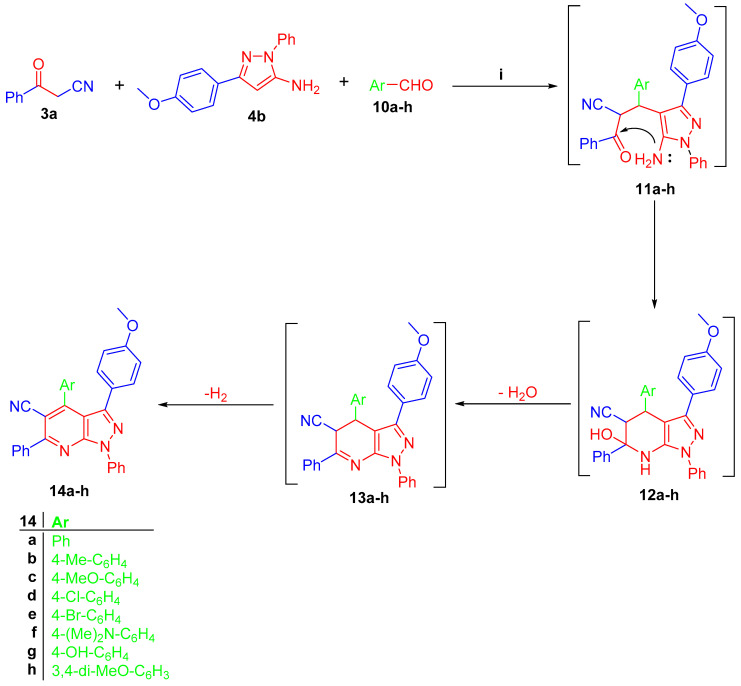
(**i**) EtOH, TEA (catalytic), reflux 6–10 h.

On the other side, one-step three components reaction of pyrazol-5-amine **4b**, 3-oxo-3-phenylpropanenitrile (**3a**) and the aromatic aldehydes **10a**–**h** give pyrazolo[3,4-*b*]pyridine-5-carbonitriles **14a**–**h**, respectively (Scheme 3). The IR of **14a**–**h** appeared as the characteristic sharp band of C≡N at 2218–2226 cm^−1^. ^1^HNMR of **14a**–**h** exhibited the singlet signal of the methoxy group of the pyrazole 4-methoxyphenyl group around *δ* 3.72–3.76 *ppm*. The ^1^HNMR of **14g** showed the D_2_O exchangeable signal of -O*H* as a broad singlet at *δ* 9.90 *ppm*, whereas the ^1^HNMR of **14f** showed the signal of two methyl groups of -N(Me) as one singlet with integration equal to 6 protons at *δ* 2.93 *ppm*. The latter reaction proceeded via the condensation reaction between 3-oxo-3-phenylpropanenitrile (**3b**) and aldehydes **10a**–**h**, then the addition of H4 of pyrazole to give intermediate **11a**–**h**. The intramolecular cyclocondensation of **11a**–**h** gives the target pyrazolo[3,4-*b*]pyridine derivatives **14a**–**h**, respectively (Scheme 3).

### 2.2. Biological Evaluation

#### 2.2.1. Anti-Cancer Activity of **9a**–**h** and **14a**–**h** against Hela, HCT-116, and MCF7 Cancer Cell Lines

The cytotoxicity of compounds **9a**–**h** and **14a**–**h** against three cell lines, namely, cervical cancer Hela, breast cancer MCF7, and colon HCT-116, compared with doxorubicin as a reference compound was performed using MTT assay. The cytotoxic concentration (IC_50_ in µM) is illustrated in Table 1. Among the screened compounds, **9a** and **14g** showed significant anticancer activity. Compound **9a** showed the highest anticancer activity, with IC_50_ = 2.59 µM against Hela cell lines when compared with doxorubicin (IC_50_ = 2.35 µM) (Table 1). Compounds **14g** revealed cytotoxicity IC_50_ = 4.66 and 1.98 µM towards MCF7 and HCT-116 cell lines compared to doxorubicin with IC_50_ = 4.57 and 2.11 µM, respectively (Table 1).

#### 2.2.2. SAR Studies

The potent anticancer activity of compounds **9a** and **14g** exhibited the impact of the electronic characteristics of substitution phenyl in position 4 of the pyridine ring with respect to its substituents in the *para* position. In compounds **9a**–**h**, these substitutions in **9b**–**h** gave low anticancer activity when compared with *para*-unsubstituted phenyl in **9a** with respect to Hela cell line. The 3,4-dimethoxyphenyl in **9h** improves the anticancer activity against Hela cell line when compared with **9b**–**f**, but it is still less than that of **9a**. Compound **14g**, with *para*-hydroxy substitution in the same phenyl ring, showed the highest anticancer activity against Hela among series **14a**–**h** but still less than the standard reference drug. Compound **14g**, with OH group in the phenyl group of position 4 in pyridine moiety, in addition to the Ph group in position 2 and the CN function in position 3 of the pyridine moiety, enhanced its anticancer activity towards MCF7 and HCT-116 cell lines. In the light of previous data, it can be noticed that the pyrazolo[3,4-*b*]pyridine system in compounds **9a**–**h** and **14a**–**h,** in addition to the substitutions around it, are necessary for their anticancer activity.

#### 2.2.3. Cell Cycle and Apoptosis

##### Cell Cycle

**Figure 3:**
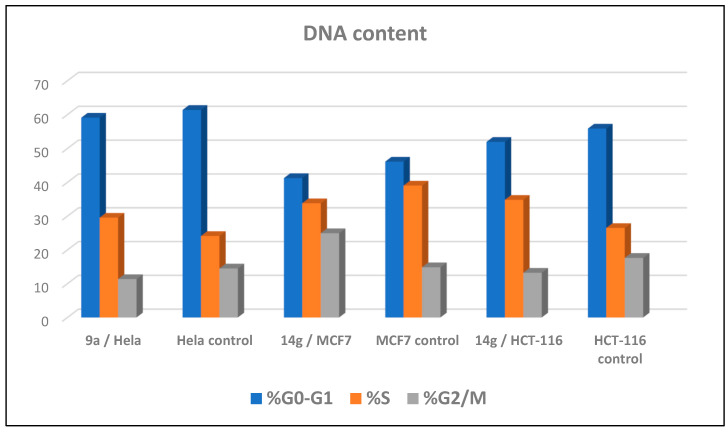
Representative diagram for cell cycle analyses for **9a** against Hela and **14g** against MCF7 and HCT-116, respectively.

**Figure 4:**
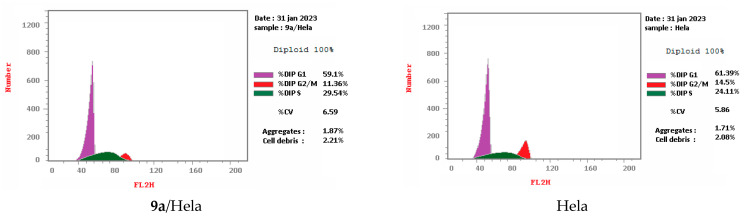

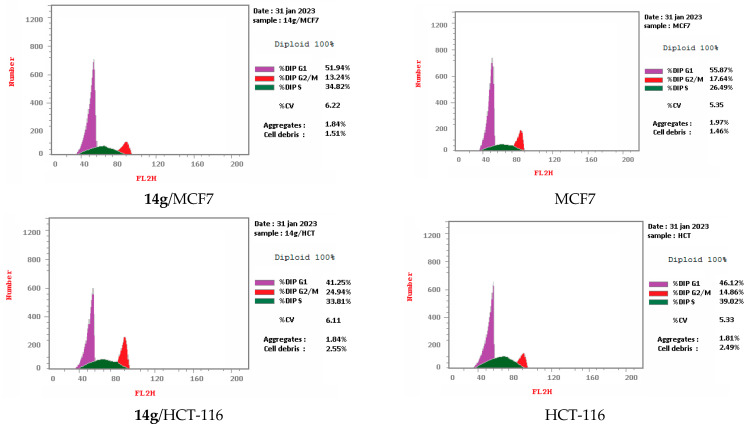
Cell cycle analyses for **9a** against Hela and **14g** against MCF7 and HCT-116, respectively.

Cell cycle arrest of cervical cancer cells Hela, breast cancer cells MCF7, and colon cancer cells HCT-116 were assessed for compounds **9a** and **14g** at their IC_50_. Compound **9a** exhibited high cell accumulation (29.54%, 1.23 fold) for the Hela cell line at the S phase (Control; 24.11%) that signifies cell cycle arrest at the S phase (Table 2, Figure 3 and Figure 4). Compound **14g** exhibited cell cycle arrest for breast cell line MCF7 at G2/M—the phase with DNA accumulation, 24.94%, (1.68 fold), compared with the control, 14.86% (Table 2, Figure 2 and Figure 3). In addition, the compound **14g** arresting cell cycle at S phase in HCT-116 cell lines with cell accumulation = 34.82% (1.97 fold) when compared with the control cells (17.64%) (Table 2, Figure 3 and Figure 4).

###### Apoptosis Assay

**Figure 5:**
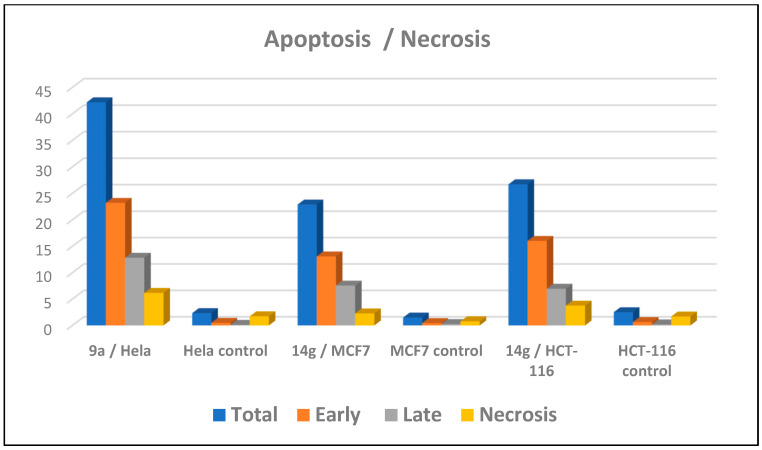
Representative diagram for apoptosis analyses for **9a** against Hela and **14g** against MCF7 and HCT-116, respectively.

**Figure 6:**
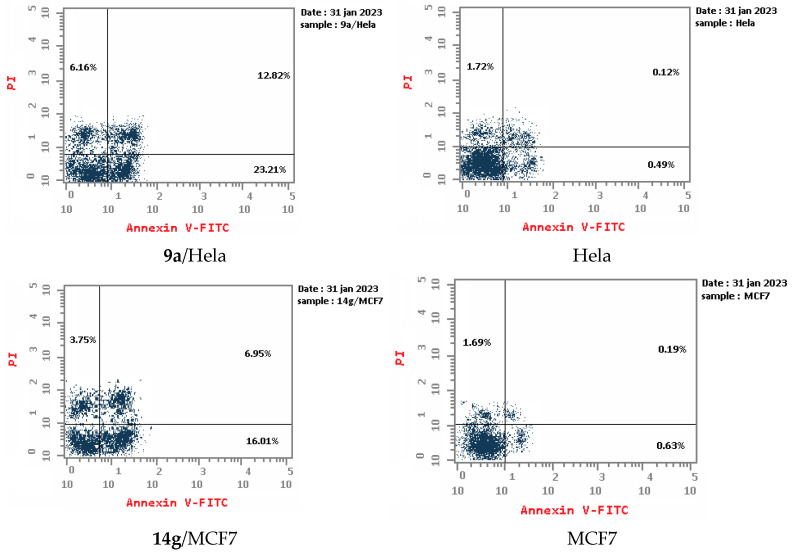

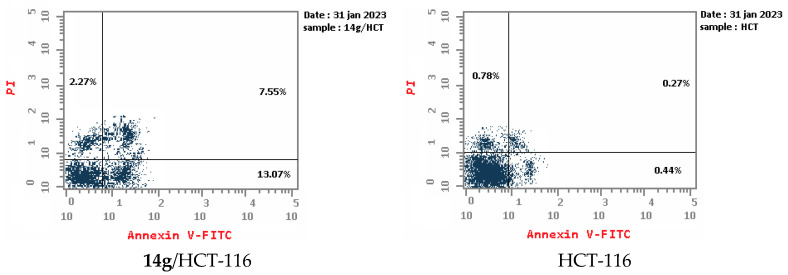
Apoptosis analyses for **9a** against Hela and **14g** against MCF7 and HCT-116, respectively.

An apoptotic assay using Annexin V/PI analysis for compounds **9a** and **14g** is evaluated. Compound **9a** induced a significant level of early and late apoptosis (total = 42.19) in the Hela cell line when compared with the control cells (Table 3, Figure 5 and Figure 6). Compound **14g** revealed total apoptosis = 22.89 and 26.71 in MCF7 and HCT-116 cell lines, respectively, compared with the control cells (Table 3, Figure 5 and Figure 6).

#### 2.2.4. Cytotoxicity of **9a** and **14g** on Normal Cell Line

The cytotoxicity of the most effective compounds **9a** and **14g** towards human normal cell line WI-38 was evaluated comparing with doxorubicin as a reference drug using MTT assay. Compounds **9a** (IC_50_ = 26.44 ± 3.23 µM) and **14g** (IC_50_ = 21.81 ± 2.96 µM) showed good safety profiles on normal cell line WI-38 when compared with doxorubicin (IC_50_ = 15.60 ± 0.37 µM) (Table 4).

#### 2.2.5. CDK2 and CDK9 Enzyme Assay Inhibition of **9a** and **14g**

The in vitro CDK2 and CDK9 inhibitory activity of **9a** and **14g** was assayed using ribociclib as a standard CDK inhibitor, and their IC_50_ (µM) are illustrated in Table 5. Compounds **9a** and **14g** showed good inhibition activity towards CDK2 with IC_50_ = 1.630 ± 0.009 and 0.460 ± 0.024 µM, respectively, when compared with ribociclib (IC_50_ 0.068 ± 0.004). Furthermore, compounds **9a** and **14g** showed inhibitory activity towards CDK9 with IC_50_ = 0.262 ± 0.013 and 0.801 ± 0.041 µM, respectively, related to IC_50_ of ribociclib = 0.050 ± 0.003.

### 2.3. Molecular Modeling

**Figure 7:**
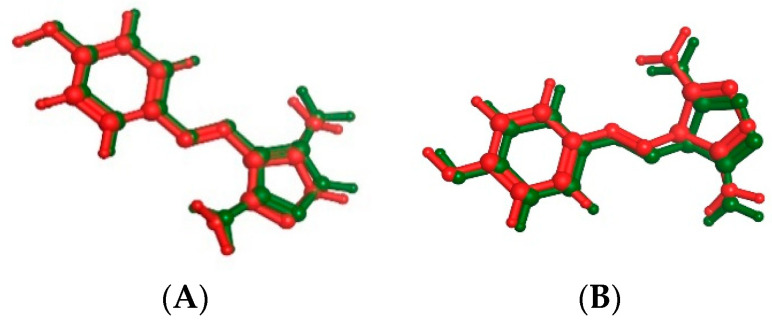
The superimposition of co-crystallized (red) and docking pose (green) of CAN508 in CDK2 (**A**) and CAN508 in CDK9 (**B**) active sites.

A molecular docking study on compounds **9a** and **14g** in the active site of CDK2 and CDK9 enzyme to correlate the in vitro *enzyme* activity with their binding mode with enzymes’ active sites using the molecular operating environment (MOE) 2019.02 and 3D coordinates of CDK2 and CDK9 (PDB IDs 3tnw and 3tn8 with resolution 2 and 2.95 Å, respectively). Valid docking protocol is established through the complexing of CDK2 and CDK9 with CAN508 as a potent CDK inhibitor with 0.265 and 0.4337 Å (Figure 7). The interaction modes of the ligand with the active sites should not be only determined as the highest energy scored protein–ligand complex used during docking but also bears in mind the conformers of each compound which are mostly associated with bioactive conformations. Based on the binding free energies and their correlation with the inhibitory activities, we can give a more quantitative explanation of the structure–activity relationship of the inhibitory mechanism for these compounds [28,29,30]. The output of the docking simulation is the scoring function which reflects the binding free energy dG in Kcal/mol. This step showed an energy score of −11.7195 and −10.045 kcal/mol for CAN508 in CDK2 and CDK9, respectively.

#### 2.3.1. Docking of **9a** and **14g** in CDK2 Binding Site

**Figure 8:**
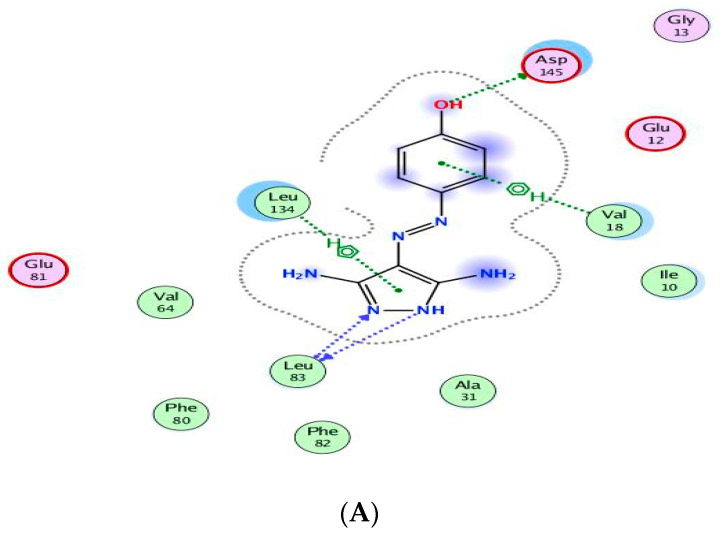

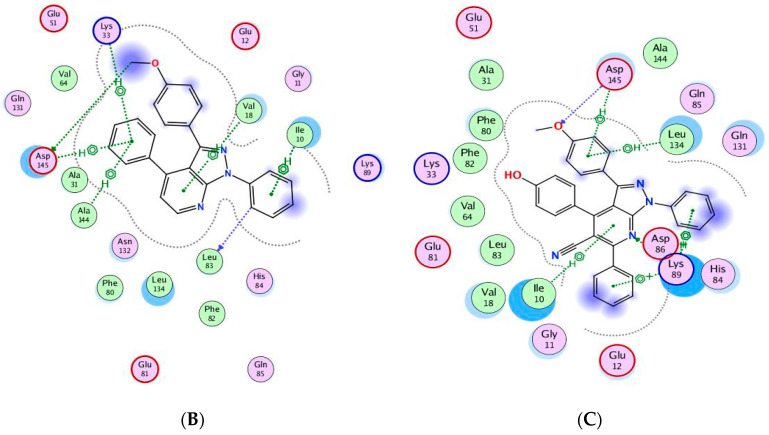
Two-dimensional binding interactions of CAN508 (**A**), **9a** (**B**), and **14g** (**C**) in the CDK2 binding site in which green arrows represent H-bond interaction in the sidechain, blue arrows represent H-bond interaction in the backbone, the benzene/H structure on the green bond represents pi–hydrogen interactions, and the benzene/+ structure on the green bond represents pi–cation interactions.

The binding of CAN508 with CDK2 active site showed the formation of two H-bonds with Asp145 and Leu83 residues, and two pi–H bonds with Val18 and Leu134 (Figure 8A). Docking simulations for **9a** and **14g** exhibited a good fit in the CDK2 active site with docking scores −13.0738 and −14.924 kcal/mol, respectively, when compared with that of CAN508 (−11.7195 kcal/mol). The higher binding free energy of **9a** and **14g**, compared with CAN508, explained their good affinity to CDK2 active site. The binding patterns of **9a** (Figure 8B) and **14g** (Figure 8C) are consistent with the crystallographic binding of CAN508 in the CDK2 active site.

**Figure 9:**
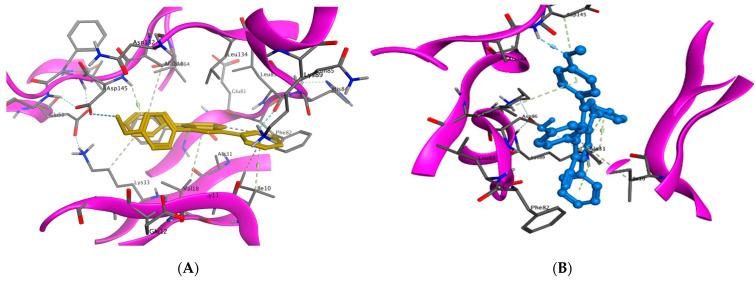
Binding interactions (3D diagram) for **9a** (**A**) and **14g** (**B**) in the CDK2 binding site.

For example, the methoxy group in the phenylmethoxy ring of both compounds **9a** and **14g** was H-bonded with the key residue Asp145. The latter H-bond highlighted the important role of the methoxy group of the phenyl ring in these compounds in CDK9 inhibition. Moreover, binding with Asp145 by pi–hydrogen bond through the phenyl ring of pyridine in **9a** and the phenylmethoxy ring in **14g** are noticed. Both compounds bind with Ile10 by pi–hydrogen bond through a phenyl ring attached to pyrazole in compound **9a** and the pyridine ring in compound **14g**. Compound **9a** binds the key residue Leu83 by a hydrogen bond through its phenyl ring attached to pyrazole. Further pi–hydrogen interactions are observed between compound **9a** and Val18, Lys33 and Ala144 (Figure 9A). Compound **14g** is anchored by the key residue Lys89 by hydrogen, pi–cation, and pi–hydrogen bonds. Further pi–hydrogen interaction between compound **14g** and Leu134 is observed (Figure 9B).

#### 2.3.2. Docking of **9a** and **14g** in CDK9 Binding Site

**Figure 10:**
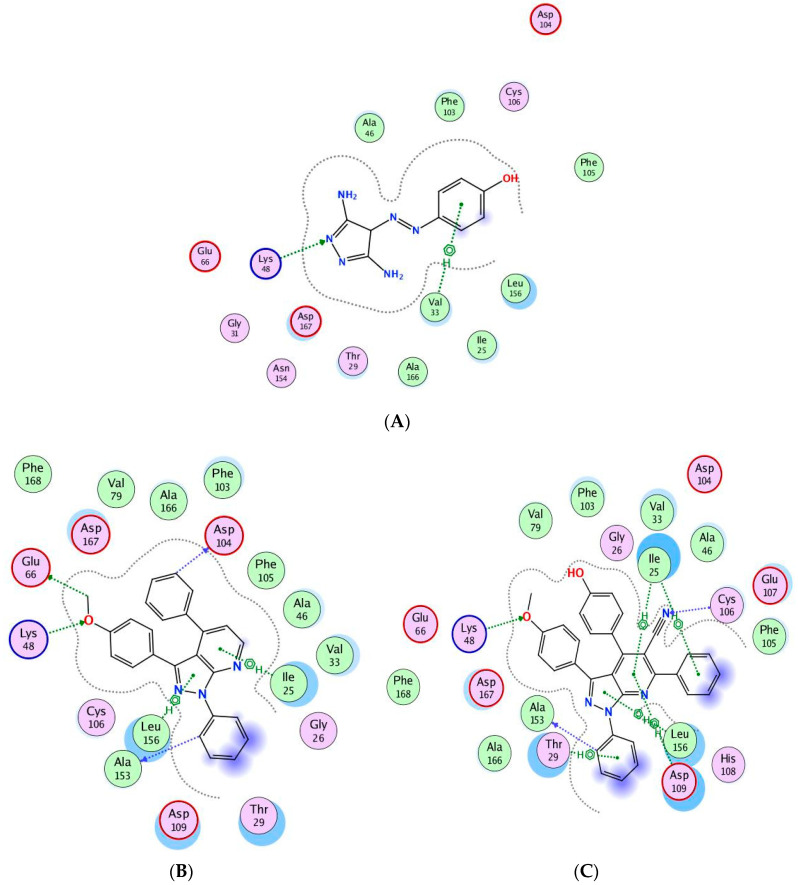
Binding interactions (2D diagram) for CAN508 (**A**), **9a** (**B**), and **14g** (**C**) in the CDK9 binding site in which green arrows represent H-bond interaction in the sidechain, blue arrows represent H-bond interaction in the backbone, and the benzene/H structure on the green bond represents pi–hydrogen interactions.

Compounds **9a** and **14g** showed good docking scores with high free binding energy (−10.6742 and −10.1599 kcal/mol) compared to CAN508 (−10.045 kcal/mol). Figure 10A shows that CAN508 formed an H-bond with the Lys48 residue and a pi–hydrogen interaction with Val33 in the CDK9 active site. Interestingly, **9a** and **14g** revealed essential interactions in CDK9 active sites (Figure 10B,C). For instance, both compounds formed an H-bond with essential key residue Lys48 via the oxygen atom of the phenylmethoxy ring. This illustrates the importance of additional methoxy group in our compounds. The phenyl ring attached to pyrazole forms a hydrogen bond with the key residue Ala153 in both compounds. Both compounds form pi–hydrogen interactions leu156 and Ile25 via pyrazole and pyridine rings, respectively, noting that there is an additional interaction in compound **14g** via the phenyl group of the pyridine ring and this explains the stronger binding and score in this compound.

**Figure 11:**
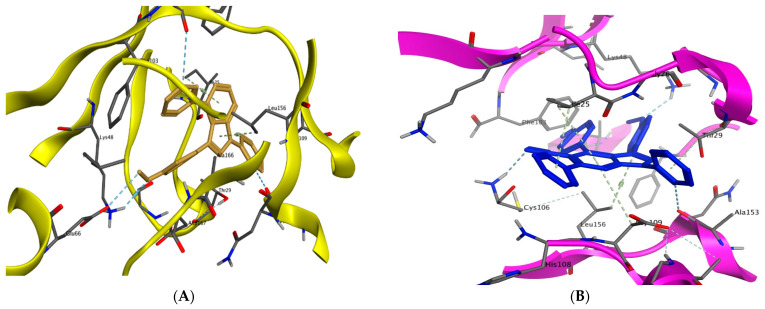
Binding interactions (3D diagram) of **9a** (**A**) and **14g** (**B**) in the CDK9 binding site.

Compound **9a** forms two hydrogen bonds with Glu66 and Asp104 via the methoxy group and phenyl ring attached to pyridine, respectively. Compound **14g** forms a hydrogen bond with the key residue Cys106 via the nitrogen atom in the nitrile group in addition to two pi–hydrogen interactions with Asp104 and Leu156 (Figure 11A,B).

## 3. In Silico ADME Study

A small molecule’s therapeutic activity is determined by its ability to reach the aimed target at a sufficient concentration, which can be determined by its ADME properties. Lipinski’s rule-of-five states if a small molecule has the potential to be a medication. SwissADME provides rapid but reliable prediction data about a small molecule’s physicochemical parameters (such as molecular weight, partition coefficient, solubility, topological surface area, and so on), as well as pharmacokinetics and drug likeness [31,32]. This method may forecast lead molecules with desired drug-like features and can be used to change or improve a molecule such that it has all desirable lead-like or drug-like properties. As a result, we employed the SwissADME model to forecast the properties of our newly created molecules **9a**–**h** and **14a**–**h**. According to the data gathered from this web tool, all compounds obey the Veber rule (Table 6) with zero violations indicating their drug-likeliness.

Compounds **9g** and **9h** have zero violations of Lipinski’s rule of five, whereas the rest have one violation, with the exception of **14c**–**f**, which has two violations. All compounds had acceptable clogP values in the range of 4.00–6.00. Topological polar surface area (TPSA) is the surface sum of all the polar atoms in a molecule, and the acceptable range is 20–130 Å, with all of our compounds falling inside this range. Except for **9a** and **9c**, none of the compounds penetrated the blood–brain barrier (BBB). Pgp is an efflux transporter that pushes xenobiotics out of cells, resulting in clearance [31,32]. Except for **9a**–**f** and **9h**, all compounds were anticipated to be non-substrates for P-gp. Except for **14c**–**f** compounds, all compounds had a bioavailability score of 0.55. Compounds **9a**–**h** have good GI absorption, whereas compounds **14a**–**h** have poor GI absorption. Except for **9g**, which has two brenk alerts, all compounds have zero pain and brenk alerts.

SwissADME provides a BOILED-Egg intrinsic model for predicting BBB entry and passive gastrointestinal absorption (HIA). It is a fantastic strategy that is based on two descriptors: WLOGP (lipophilicity) and TPSA (apparent polarity). The white region of the BOILED-Egg indicates a high likelihood of passive absorption through the gastrointestinal tract, but the yellow part indicates a high likelihood of reaching the brain. P-gp is a multidrug resistance efflux pump that is in charge of drug clearance. The presence or absence of PGP in a drug candidate is shown by blue or red spots in the BOILED-Egg plot.

**Figure 12:**
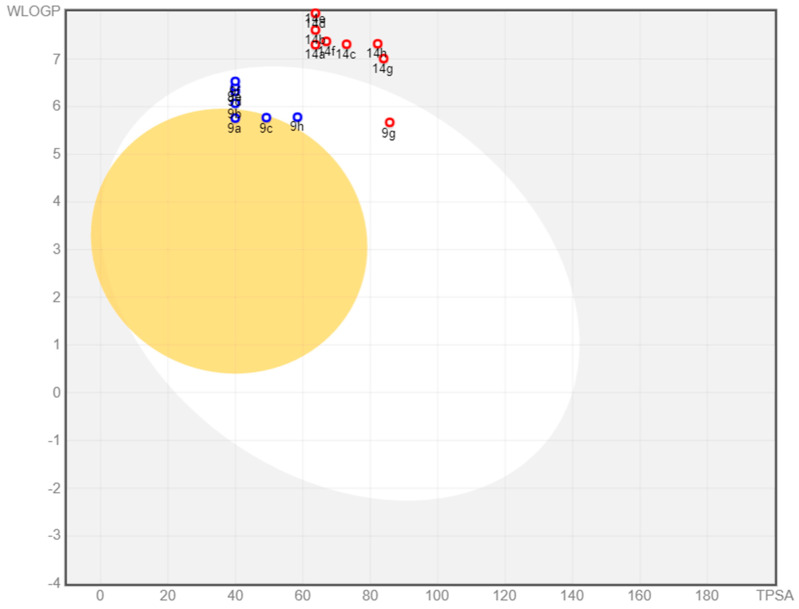
Spread of **9a**–**h** and **14a**–**h** compounds on the BOILED-Egg plot.

Figure 12 illustrates the distribution of **9a**–**h** and **14a**–**h** on BOILED-Egg. Only **9a** and **9c** compounds were found in the yellow zone, indicating that they are BBB penetrant, whereas the remaining compounds are not. The presence of **9a**–**h** compounds in the white region indicated proper absorption, whereas **14a**–**h** compounds were identified outside the white region, indicating inadequate GI absorption. Among all compounds, seven were anticipated to be P-gp substrates (PGP+) (blue dot), while the remaining compounds were not subjected to the active efflux P-gp pump (PGP-) (red dot).

## 4. Conclusions

Novel two series pyrazolo[3,4-*b*]pyridine derivatives **9a**–**h** and **14a**–**h** were prepared and assessed for their anti-cancer activity against Hela, MCF7, and HCT-116 cancer cell lines. Compounds **9a** and **14g** showed the highest anticancer activity, the highest cell cycle arrest, and a significant level of early and late apoptosis. They showed good safety profiles on normal cell line WI-38. They also showed good inhibition activity towards CDK2 and CDK9. Compounds **9a** and **14g** showed essential interactions in their docking in CDK2 and CDK9 active sites. In the light of these results, the situation definitely calls for additional research to understand the effect of substitutions around pyrazolo[3,4-*b*]pyridine central moiety and the activity of a such class of compounds.

## 5. Experimental Section

### 5.1. Chemistry

2-Bromo-1-(phenyl/4-methoxyphenyl)ethan-1-ones **2a**, **b** [33], 3-(phenyl/4-methoxyphenyl)-3-oxopropanenitriles **3a**, **b** [34], aminopyrazole **4b** [35], and enamenones **5a**–**g** [36] were prepared following the reported method. Instruments and thermal analysis results are listed in Supporting Data.

#### 5.1.1. Synthesis of Pyrazolo[3,4-*b*]pyridine Derivatives **9a**–**h**

A mixture of 3-(4-methoxyphenyl)-1-phenyl-1*H*-pyrazol-5-amine (**4b**) (0.265 g, 1 mmol) and enamenones **6a**–**h** (1 mmol) in glacial acetic acid (30 mL) was refluxed for 4–6 h. After cooling, the formed precipitate was filtered, washed with EtOH, and crystallized from EtOH/DMF to yield the corresponding pyrazolo[3,4-*b*]pyridine derivatives **9a**–**h**, respectively.

##### 3-(4-Methoxyphenyl)-1,4-diphenyl-1*H*-pyrazolo[3,4-*b*]pyridine (**9a**)

White powder, 72% yield; mp 161–163 °C; IR *ν*_max_/cm^−1^ 1593 (C=N), 1501 (C=C), 1250 (C–O); ^1^H-NMR *δ* 3.87 (s, 3H, OMe), 7.16 (d, *J* = 8.8 Hz, 2H, ArHs), 7.39 (t, *J* = 7.4 Hz, 1H, ArH), 7.51–7.67 (m, 5H, ArHs), 8.01 (d, *J* = 8.5 Hz, 1H, H3 of pyridine), 8.09 (d, *J* = 8.7 Hz, 2H, ArHs), 8.27 (d, *J* = 7.3 Hz, 2H, ArHs), 8.46 (d, *J* = 7.2 Hz, 2H, ArHs), 8.70 (d, *J* = 8.5 Hz, 1H, H2 of pyridine); ^13^C-NMR *δ* 55.77 (C of OMe), 113.95, 115.03 (2C), 115.99, 121.05 (2C), 125.01, 126.23, 127.80 (2C), 128.85 (2C), 129.48 (2C), 129.70 (2C), 130.33, 132.65, 138.62, 139.68, 143.92, 151.39, 156.42, 160.38; MS *m/z* (*%*) 377.08 (M^+^,21.80). For C_25_H_19_N_3_O (377.45): Calc.: C, 79.55; H, 5.07; N, 11.13. Found: C, 79.31; H, 5.20; N, 11.40.

##### 3-(4-Methoxyphenyl)-1-phenyl-4-(p-tolyl)-1*H*-pyrazolo[3,4-*b*]pyridine (**9b**)

White powder, 75% yield; mp 186–188 °C; IR *ν*_max_/cm^−1^ 1593 (C=N), 1501 (C=C), 1250 (C–O); ^1^H-NMR *δ* 2.41 (s, 3H, Me), 3.87 (s, 3H, OMe), 7.16 (d, *J* = 8.8 Hz, 2H, ArHs), 7.39 (d, *J* = 8.0 Hz, 3H, ArHs), 7.64 (t, *J* = 7.8 Hz, 2H, ArHs), 7.99 (d, *J* = 8.6 Hz, 1H, H3 of pyridine), 8.09 (d, *J* = 8.9 Hz, 2H, ArHs), 8.17 (d, *J* = 8.4 Hz, 2H, ArHs), 8.46 (d, *J* = 8.6 Hz, 2H, ArHs), 8.69 (d, *J* = 8.5 Hz, 1H, H2 of pyridine); ^13^C-NMR *δ* 21.38 (C of Me), 55.75 (C of OMe), 113.73, 115.01 (2C), 115.70, 120.99 (2C), 125.05, 126.17, 127.68 (2C), 128.82 (2C), 129.68 (2C), 130.07 (2C), 132.50, 135.84, 139.72, 140.07, 143.88, 151.40, 156.42, 160.35; MS *m/z* (*%*) 391.47 (M^+^, 52.80). For C_26_H_21_N_3_O (391.47): Calc.: C, 79.77; H, 5.41; N, 10.73. Found: C, 79.54; H, 5.68; N, 10.98.

##### 3,4-Bis(4-methoxyphenyl)-1-phenyl-1*H*-pyrazolo[3,4-*b*]pyridine (**9c**)

White powder, 66% yield; mp 198–200 °C; IR *ν*_max_/cm^−1^ 1600 (C=N), 1501(C=C), 1254 (C–O); ^1^H-NMR *δ* 3.855 (s, 3H, OMe), 3.865 (s, 3H, OMe), 7.09–7.18 (m, 4H, ArHs), 7.37 (t, *J* = 7.4 Hz, 1H, ArH), 7.63 (t, *J* = 7.8 Hz, 2H, ArHs), 7.94 (d, *J* = 8.6 Hz, 1H, H3 of pyridine), 8.07 (d, *J* = 8.6 Hz, 2H, ArHs), 8.23 (d, *J* = 8.7 Hz, 2H, ArHs), 8.45 (d, *J* = 8.0 Hz, 2H, ArHs), 8.63 (d, *J* = 8.5 Hz, 1H, H2 of pyridine); ^13^C-NMR *δ* 55.75 (C of OMe), 55.79 (C of OMe), 113.38, 114.86 (2C), 115.01 (2C), 115.34, 120.96 (2C), 125.09, 126.13, 128.81 (2C), 129.24 (2C), 129.67 (2C), 130.98, 132.42, 139.75, 143.88, 151.44, 156.20, 160.33, 161.29; MS *m/z* (*%*) 407.33 (M^+^, 14.18). For C_26_H_21_N_3_O_2_ (407.47): Calc.: C, 76.64; H, 5.19; N, 10.31. Found: C, 76.86; H, 5.32; N, 10.47.

##### 4-(4-Fluorophenyl)-3-(4-methoxyphenyl)-1-phenyl-1*H*-pyrazolo[3,4-*b*]pyridine (**9d**)

White powder, 74% yield; mp 171–173 °C; IR *ν*_max_/cm^−1^ 1597 (C=N), 1505 (C=C), 1250 (C–O), 1227 (C-F); ^1^H-NMR *δ* 3.87 (s, 3H, OMe), 7.15 (d, *J* = 8.7 Hz, 2H, ArHs), 7.40 (q, *J* = 8.6 Hz, 3H, ArHs), 7.63 (t, *J* = 7.9 Hz, 2H, ArHs), 8.00 (d, *J* = 8.5 Hz, 1H, H3 of pyridine), 8.08 (d, *J* = 8.7 Hz, 2H, ArHs), 8.33 (dd, *J* = 8.7, 5.7 Hz, 2H, ArHs), 8.43 (d, *J* = 8.6 Hz, 2H, ArHs), 8.70 (d, *J* = 8.5 Hz, 1H, H2 of pyridine); ^13^C-NMR *δ* 55.77 (C of OMe), 113.86, 115.03 (2C), 115.82, 116.27, 116.49, 121.11 (2C), 124.96, 126.28, 128.86 (2C), 129.71 (2C), 130.02, 130.11, 132.77, 134.76, 139.62, 143.93, 151.28, 155.36, 160.39, 165.03; MS *m/z* (*%*) 395.64 (M^+^, 26.35). For C_25_H_18_FN_3_O (395.44): Calc.: C, 75.93; H, 4.59; N, 10.63. Found: C, 76.09; H, 4.72; N, 10.91.

##### 4-(4-Chlorophenyl)-3-(4-methoxyphenyl)-1-phenyl-1*H*-pyrazolo[3,4-*b*]pyridine (**9e**)

White powder, 72% yield; mp 210–212 °C; IR *ν*_max_/cm^−1^ 1599 (C=N), 1501 (C=C), 1254 (C–O); ^1^H-NMR *δ* 3.87 (s, 3H, OMe), 7.15 (d, *J* = 8.4 Hz, 2H, ArHs), 7.39 (t, *J* = 7.4 Hz, 1H, ArH), 7.63 (dd, *J* = 8.3, 6.3 Hz,, 4H, ArHs), 8.03 (d, *J* = 8.5 Hz, 1H, H3 of pyridine), 8.09 (d, *J* = 8.6 Hz, 2H, ArHs), 8.29 (d, *J* = 8.3 Hz, 2H, ArHs), 8.42 (d, *J* = 8.0 Hz, 2H, ArHs), 8.72 (d, *J* = 8.6 Hz, 1H, H2 of pyridine); ^13^C-NMR *δ* 55.78 (C of OMe), 110.10, 115.08, 115.96, 121.18 (2C), 122.66, 124.92, 126.63, 128.89, 129.56 (2C), 129.75 (2C), 135.61, 137.43, 139.56, 144.00, 149.39, 151.27, 152.74, 153.76, 155.17, 160.42, 161.98; MS *m/z* (*%*) 412.40 (M^+^+1, 36.04), 411.71 (M^+^, 53.37). For C_25_H_18_ClN3O (411.89): Calc.: C, 72.90; H, 4.41; N, 10.20. Found: C, 73.14; H, 4.60; N, 10.43.

##### 4-(4-Bromophenyl)-3-(4-methoxyphenyl)-1-phenyl-1*H*-pyrazolo[3,4-*b*]pyridine (**9f**)

Pale yellow powder, 65% yield; mp 217–219 °C; IR *ν*_max_/cm^−1^ 1596 (C=N), 1501 (C=C), 1254 (C–O), 1227 (C–F); ^1^H-NMR *δ* 3.86 (s, 3H, OMe), 7.14 (d, *J* = 8.2 Hz, 2H, ArHs), 7.38 (t, *J* = 7.4 Hz, 1H, ArHs), 7.62 (t, *J* = 7.8 Hz, 2H, ArHs), 7.76 (d, *J* = 8.1 Hz, 2H, ArHs), 7.99 (d, *J* = 8.5 Hz, 1H, H3 of pyridine), 8.07 (d, *J* = 8.2 Hz, 2H, ArHs), 8.19 (d, *J* = 8.1 Hz, 2H, ArHs), 8.41 (d, *J* = 8.0 Hz, 2H, ArHs), 8.69 (d, *J* = 8.5 Hz, 1H, H2 of pyridine); ^13^C-NMR *δ* 55.76 (C of OMe), 107.26, 114.15, 115.23 (2C), 115.92, 121.15 (2C), 126.47, 128.85, 129.65, 129.76 (2C), 131.76 (2C), 132.13, 143.93, 139.75, 145.47, 151.24, 154.43, 154.97, 157.59, 162.42, 166.54; MS *m/z* (*%*) 456.20 (M^+^, 23.09). For C_25_H_18_BrN_3_O (456.34): Calc.: C, 65.80; H, 3.98; N, 9.21. Found: C, 65.97; H, 4.06; N, 9.42.

##### 3-(4-Methoxyphenyl)-4-(4-nitrophenyl)-1-phenyl-1*H*-pyrazolo[3,4-*b*]pyridine (**9g**)

Orange powder, 68% yield; mp > 300 °C; IR *ν*_max_/cm^−1^ 1597 (C=N), 1520 (C-NO_2_), 1497 (C=C), 1343 (C–NO_2_), 1250 (C–O); ^1^H-NMR *δ* 3.88 (s, 3H, OMe), 7.18 (d, *J* = 8.3 Hz, 2H, ArHs), 7.42 (s, 1H, ArH), 7.67 (d, *J* = 8.2 Hz, 3H, ArHs), 8.16–8.43 (m, 7H, 6 ArHs + H3 of pyridine), (d, *J* = 8.1 Hz, 4H, ArHs), 8.56 (d, *J* = 8.4 Hz, 2H, H2 of pyridine); MS *m/z* (*%*) 422.27 (M^+^, 43.75). For C_25_H_18_NO_3_ (422.44): Calc.: C, 71.08; H, 4.30; N, 13.26. Found: C, 70.89; H, 4.57; N, 13.47.

##### 4-(3,4-Dimethoxyphenyl)-3-(4-methoxyphenyl)-1-phenyl-1*H*-pyrazolo[3,4-*b*]pyridine (**9h**)

White powder, 76% yield; mp 165–167 °C; IR *ν*_max_/cm^−1^ 1593 (C=N), 1520, 1505 (C=C), 1250 (C–O–C); ^1^H-NMR *δ* 3.86 (s, 3H, OMe), 3.87 (s, 3H, OMe), 3.93 (s, 3H, OMe), 7.14 (dd, *J* = 8.5, 6.0 Hz, 3H, ArHs), 7.37 (t, *J* = 7.4 Hz, 1H, ArHs), 7.59–7.67 (m, 2H, ArHs), 7.82–7.91 (m, 2H, ArHs), 8.00 (d, *J* = 8.6 Hz, 1H, H3 of pyridine), 8.08 (d, *J* = 8.8 Hz, 2H, ArHs), 8.48 (d, *J* = 7.4 Hz, 2H, ArHs), 8.63 (d, *J* = 8.6 Hz, 1H, H2 of pyridine); ^13^C-NMR *δ* 55.74 (C of OMe), 55.90 (C of OMe), 56.07 (C of OMe), 110.77, 112.28, 115.01, 115.44 (2C), 120.72, 120.90 (2C), 125.07, 126.14, 128.80 (2C), 129.63 (2C), 131.06, 132.30, 139.69, 143.89, 149.43, 151.02, 151.32, 156.12, 160.32; MS *m/z* (*%*) 437.66 (M^+^, 21.67). For C_27_H_23_N_3_O_3_ (437.50): Calc.: C, 74.13; H, 5.30; N, 9.60. Found: C, 74.40; H, 5.42; N, 9.78.

#### 5.1.2. Synthesis of Pyrazolo[3,4-*b*]pyridine Derivatives **14a**–**g**

A mixture of aminopyrazole **4b** (0.265 g, 1 mmol), propanenitrile **3a** (0.145 g, 1 mmol), and the appropriate aromatic aldehydes **10a**–**g** (1 mmol) in absolute EtOH (30 mL) was refluxed for 6–10 h in the presence of TEA (catalytic). The formed precipitated was filtered, washed with EtOH, and crystallized from EtOH/DMF to yield pyrazolo[3,4-*b*]pyridine derivatives **14a**–**h**, respectively.

##### 3-(4-Methoxyphenyl)-1,4,6-triphenyl-1*H*-pyrazolo[3,4-*b*]pyridine-5-carbonitrile (**14a**)

White powder, 74% yield; mp 229–231 °C; IR *ν*_max_/cm^−1^ 2218 (C≡N), 1610 (C=N), 1559, 1497 (C=C), 1250 (C–O); ^1^H-NMR *δ* 3.72 (s, 3H, OMe), 6.65 (d, *J* = 8.7 Hz, 2H, ArHs), 7.03 (d, *J* = 8.6 Hz, 2H), 7.32 (t, *J* = 7.5 Hz, 2H, ArHs), 7.39–7.47 (m, 4H, ArHs), 7.60–7.66 (m, 5H, ArHs), 8.00–8.02 (m, 2H, ArHs), 8.29 (d, *J* = 7.4 Hz, 2H); ^13^C-NMR *δ* 55.61 (C of OMe), 102.37, 112.26, 113.54 (2C), 118.02, 122.04 (2C), 123.87, 127.35, 128.50 (2C), 128.99 (2C), 129.78 (2C), 129.85 (2C), 129.94 (2C), 130.07, 130.54 (2C), 130.70, 133.93, 138.10, 138.66, 147.06, 150.28, 153.02, 159.66, 160.76; MS *m/z* (*%*) 478.58 (M^+^, 15.55). For C_32_H_22_N_4_O (478.56): Calc.: C, 80.32; H, 4.63; N, 11.71. Found: C, 80.09; H, 4.74; N, 11.94.

##### 3-(4-Methoxyphenyl)-1,6-diphenyl-4-(p-tolyl)-1*H*-pyrazolo[3,4-*b*]pyridine-5-carbonitrile (**14b**)

White powder, 68% yield; mp 203–205 °C; IR *ν*_max_/cm^−1^ 2218 (C≡N), 1609, (C=N), 1559, 1509 (C=C), 1250 (C–O); ^1^H-NMR *δ* 2.34 (s, 3H, Me), 3.72 (s, 3H, OMe), 6.64 (d, *J* = 8.7 Hz, 2H, ArHs), 7.00 (d, *J* = 8.7 Hz, 2H, ArHs), 7.10 (d, *J* = 7.9 Hz, 2H, ArHs), 7.25 (d, *J* = 8.1 Hz, 2H, ArHs), 7.42 (t, *J* = 7.5 Hz, 1H, ArHs), 7.57–7.65 (m, 5H, ArHs), 7.99–8.01 (m, 2H, ArHs), 8.28 (d, *J* = 8.5 Hz, 2H, ArHs); ^13^C-NMR *δ* 21.32 (C of Me), 55.64 (C of OMe), 102.28, 112.39, 113.46 (2C), 118.11, 122.05 (2C), 123.91, 127.34, 128.97 (2C), 129.79 (2C), 129.92 (2C), 130.58 (2C), 130.67, 131.04, 138.13, 138.67, 139.85, 147.10, 150.27, 153.19, 159.70, 160.79; MS *m/z* (*%*) 492.77 (M^+^, 58.66). For C_33_H_24_N_4_O (492.58): Calc.: C, 80.47; H, 4.91; N, 11.37. Found: C, 80.68; H, 4.85; N, 11.53.

##### 3,4-Bis(4-methoxyphenyl)-1,6-diphenyl-1*H*-pyrazolo[3,4-*b*]pyridine-5-carbonitrile (**14c**)

White powder, 65% yield; mp 207–209 °C; IR *ν*_max_/cm^−1^ 2218 (C≡N), 1609 (C=N), 1551, 1509 (C=C), 1250 (C–O); ^1^H-NMR *δ* 3.74 (s, 3H, OMe), 3.78 (s, 3H, OMe), 6.69 (d, *J* = 8.7 Hz, 2H, ArHs), 6.84 (d, *J* = 8.7 Hz, 2H, ArHs), 7.04 (d, *J* = 8.7 Hz, 2H, ArHs), 7.31 (d, *J* = 8.6 Hz, 2H, ArHs), 7.43 (t, *J* = 7.4 Hz, 1H, ArHs), 7.58–7.68 (m, 5H, ArHs), 7.99 - 8.02 (m, 2H, ArHs), 8.29 (d, *J* = 8.5 Hz, 2H, ArHs); ^13^C-NMR *δ* 55.66 (C of OMe), 55.83 (C of OMe), 102.36, 112.51, 113.58 (2C), 113.97 (2C), 118.26, 122.09 (2C), 124.06, 125.96, 127.35, 128.98 (2C), 129.82 (2C), 129.95 (2C), 130.66 (3C), 131.56 (2C), 138.23, 138.73, 147.22, 150.33, 152.99, 159.70, 160.87, 160.94; MS *m/z* (*%*) 508.37 (M^+^, 24.90). For C_33_H_24_N_4_O_2_ (508.58): Calc.: C, 77.94; H, 4.76; N, 11.02. Found: C, 78.12; H, 4.92; N, 11.24.

##### 4-(4-Chlorophenyl)-3-(4-methoxyphenyl)-1,6-diphenyl-1*H*-pyrazolo[3,4-*b*]pyridine-5-carbonitrile (**14d**)

White powder, 70% yield; mp 258–260 °C; IR *ν*_max_/cm^−1^ 2226 (C≡N), 1610 (C=N), 1493 (C=C), 1250 (C–O); ^1^H-NMR *δ* 3.74 (s, 3H, OMe), 6.69 (d, *J* = 6.5 Hz, 2H, ArHs), 7.02 (d, *J* = 6.4 Hz, 2H, ArHs), 7.45–7.32 (m, 5H, ArHs) 7.59–7.63 (m, 5H, ArHs), 8.01 (d, *J* = 6.7, Hz, 2H, ArHs), 8.28 (d, *J* = 8.2 Hz, 2H, ArHs); ^13^C-NMR *δ* 55.71 (C of OMe), 112.77, 113.61 (2C), 115.61, 117.88, 122.07 (2C), 123.73, 127.44, 128.48 (2C), 129.04 (3C), 129.85 (3C), 129.93 (2C), 130.72 (2C), 130.77 (2C), 131.75 (2C), 132.69, 135.16, 138.04, 138.65, 150.23, 159.87; MS *m/z* (*%*) 514.87, (M^+^+1, 19.95), 513.02 (M^+^, 46.40). For C_32_H_21_ClN_4_O (513.00): Calc.: C, 74.92; H, 4.13; N, 10.92. Found: C, 74.81; H, 4.29; N, 11.15.

##### 4-(4-Bromophenyl)-3-(4-methoxyphenyl)-1,6-diphenyl-1*H*-pyrazolo[3,4-*b*]pyridine-5-carbonitrile (**14e**)

White powder, 82% yield; mp 221–223 °C; IR *ν*_max_/cm^−1^ 2226 (C≡N), 1609 (C=N), 1524, 1489 (C=C), 1250 (C-O); ^1^H-NMR *δ* 3.76 (s, 3H, OMe), 6.70 (d, *J* = 8.7 Hz, 2H, ArHs), 7.02 (d, *J* = 8.7 Hz, 2H, ArHs), 7.32 (d, *J* = 8.4 Hz, 2H, ArHs), 7.42 (t, *J* = 7.4 Hz, 1H, ArHs), 7.50 (d, *J* = 8.5 Hz, 2H, ArHs), 7.58–7.69 (m, 5H, ArHs), 8.00–8.02 (m, 2H, ArHs), 8.29 (d, *J* = 7.3 Hz, 2H, ArHs); ^13^C-NMR *δ* 55.68 (C of OMe), 102.07, 112.40, 113.55 (2C), 117.87, 121.93 (2C), 123.63, 123.85, 127.37, 129.03 (2C), 129.80 (2C), 129.90 (2C), 130.67 (2C), 130.78, 131.38 (2C), 131.90 (2C), 132.97, 137.96, 138.60, 146.98, 150.18, 151.70, 159.81, 160.70; MS *m/z* (*%*) 559.62 (M^+^+2, 7.68), 557.96 (M^+^, 25.99). For C_32_H_21_BrN_4_O (557.45): Calc.: C, 68.95; H, 3.80; N, 10.05. Found: C, 69.07; H, 3.94; N, 10.21.

##### 4-(4-(Dimethylamino)phenyl)-3-(4-methoxyphenyl)-1,6-diphenyl-1*H*-pyrazolo[3,4-*b*]pyridine-5-carbonitrile (**14f**)

Pale yellow powder, 63% yield; mp 230–232 °C; IR *ν*_max_/cm^−1^ 2218 (C≡N), 1613 (C=N), 1524, 1489 (C=C), 1250 (C–O); ^1^H-NMR *δ* 2.93 (s, 6H, 2NMe), 3.74 (s, 3H, OMe), 6.55 (d, *J* = 8.9 Hz, 2H, ArHs), 6.68 (d, *J* = 8.8 Hz, 2H, ArHs), 7.05 (d, *J* = 8.7 Hz, 2H, ArHs), 7.17 (d, *J* = 8.8 Hz, 2H, ArHs), 7.42 (t, *J* = 7.5 Hz, 1H, ArHs), 7.57–7.66 (m, 5H, ArHs), 7.98–8.01 (m, 2H, ArHs), 8.29 (d, *J* = 7.3 Hz, 2H); ^13^C-NMR *δ* 40.31 (2C of 2Me), 55.60 (C of OMe), 101.81, 111.59 (2C), 112.28, 113.57 (2C), 118.69, 120.52, 122.08, 124.36, 127.26, 128.92 (2C), 129.78 (2C), 129.95 (2C), 130.54, 130.67 (2C), 131.22 (2C), 138.42, 138.81, 147.39, 150.45, 151.75, 153.68, 159.56, 161.08; MS *m/z* (*%*) 521.28 (M^+^, 19.10). For C_34_H_27_N_5_O (521.62): Calc.: C, 78.29; H, 5.22; N, 13.43. Found: C, 78.43; H, 5.46; N, 13.67.

##### 4-(4-Hydroxyphenyl)-3-(4-methoxyphenyl)-1,6-diphenyl-1*H*-pyrazolo[3,4-*b*]pyridine-5-carbonitrile (**14g**)

White powder, 77% yield; mp 295–297 °C; IR *ν*_max_/cm^−1^ 2222 (C≡N), 1613 (C=N), 1555, 1512 (C=C), 1250 (C–O); ^1^H-NMR *δ* 3.74 (s, 3H, OMe), 6.68 (dd, *J* = 13.4, 8.7 Hz, 4H, ArHs), 7.06 (d, *J* = 8.7 Hz, 2H, ArHs), 7.18 (d, *J* = 8.6 Hz, 2H, ArHs), 7.41 (t, *J* = 7.4 Hz, 1H, ArH), 7.63–7.57 (m, 5H, ArHs), 8.02–7.97 (m, 2H, ArHs), 8.28 (dd, *J* = 8.7, 1.2 Hz, 2H, ArHs), 9.90 (s, D_2_O exchangeable, 1H, OH); ^13^C-NMR *δ* 55.68 (C of OMe), 102.30, 112.32, 113.57 (2C), 115.25 (2C), 118.38, 122.05, 124.19, 124.34, 127.28, 128.94 (2C), 129.77 (2C), 129.94 (2C), 130.60, 130.71 (2C), 131.65 (2C), 138.28, 138.75, 147.23, 150.39, 153.39, 159.44, 159.70, 160.92; MS *m/z* (*%*) 494.63 (M^+^, 13.48). For C_32_H_22_N_4_O_2_ (494.55): Calc.: C, 77.72; H, 4.48; N, 11.33. Found: C, 77.50; H, 4.52; N, 11.50.

##### 4-(3,4-Dimethoxyphenyl)-3-(4-methoxyphenyl)-1,6-diphenyl-1*H*-pyrazolo[3,4-*b*]pyridine-5-carbonitrile (**14h**)

White powder, 69% yield; mp 212–214 °C; IR *ν*_max_/cm^−1^ 2222 (C≡N), 1609 (C=N), 1555, 1505 (C=C), 1258 (C–O); ^1^H-NMR *δ* 3.37 (s, 3H, OMe), 3.73 (s, 3H, OMe), 3.82 (s, 3H, OMe), 6.70 (d, *J* = 8.4 Hz, 2H, ArHs), 6.77 (d, *J* = 2.1 Hz, 1H, ArHs), 7.03 (dd, *J* = 14.1, 8.3 Hz, 3H, ArHs), 7.15 (dd, *J* = 8.2, 2.0 Hz, 1H, ArHs), 7.42 (t, *J* = 7.4 Hz, 1H, ArHs), 7.58–7.66 (m, 5H, ArHs), 7.99–8.02 (m, 2H, ArHs), 8.28 (d, *J* = 8.0 Hz, 2H, ArHs); ^13^C-NMR *δ* 55.60 (C of OMe), 55.73 (C of OMe), 56.22 (C of OMe), 102.24, 111.90, 112.33, 113.52 (2C), 114.48, 118.36, 122.13 (2C), 122.83, 124.36, 126.01, 127.36, 128.97 (2C), 129.81 (2C), 129.95 (2C), 130.64, 130.68 (2C), 138.25, 138.72, 147.18, 148.68, 150.35, 150.68, 152.93, 159.78, 160.94; MS *m/z* (*%*) 538.16 (M^+^, 27.45). For C_34_H_26_N_4_O_3_ (538.61): Calc.: C, 75.82; H, 4.87; N, 10.40. Found: C, 75.96; H, 4.98; N, 10.68.

### 5.2. Biological Screening

#### 5.2.1. MTT Assay for Cytotoxicity

Standard MTT colorimetric assay was applied to test the IC_50_ of compounds **9a**–**h** and **14a**–**h** against three cell lines, namely, cervical cancer Hela, breast cancer MCF7, and colon HCT-116, compared with doxorubicin as a reference compound with the typical reported methodology of MTT colorimetric assay [37].

#### 5.2.2. Cell Cycle Analysis and Apoptotic Assay

Cell cycle analysis and apoptosis study of the 2 most active compounds, **9a** and **14g**, was accomplished using propidium iodide (PI) flow cytomertric analysis according to the reported procedure [38,39]. An apoptotic study using the Annexin V-FITC Apoptosis Detection Kit (K101–25, BioVision^®^, 980 Linda Vista Avenue, Mountain View, CA, USA) at their IC_50_ concentration values was used on the specified cell after 24 h in three successive steps according to the manufacturer’s instructions.

#### 5.2.3. In Vitro CDK Enzyme Assay

The CDK in vitro inhibitory activity of **9a**–**h** and **14a**–**h** toward CDK2 and CDK9 was performed using CDK Assay Kit (BPS Bioscience, USA) at different dilutions of 30, 10, 3, 1, 0.3, 0.1, 0.03, and 0.01 µM, measuring the chemiluminescence using BioTek™ Synergy2 Microplate Reader (BioTek, Santa Clara, CA, USA) [40].

### 5.3. Molecular Docking

Docking studies of compounds **9a** and **14g** were performed using Molecular Operating Environment (MOE 2019.02) software [41,42,43], and the crystal structures of CDK2 and CDK9 in complex with CAN508 were downloaded from the protein databank PDB IDs 3tnw and 3tn8, respectively.

### 5.4. In-Silico SwissADME Predictions

SwissADME is a reliable, free online utility [44] for calculating the physicochemical properties of compounds. By uploading the structures to the website http://www.swissadme.ch/, accessed on 10 March 2023, the bioavailability and pharmacokinetic parameters of any synthetic compound can be determined. The smiles of **9a**–**9h** and **14a**–**14h** structures were imported into the SwissADME environment. Different physiological and pharmacokinetic parameter measurements were exported as a CSV file, from which data were extracted and analyzed.

## Figures and Tables

**Table 1:** Anticancer activity of **9a**–**h** and **14a**–**h** against MCF7, HCT-116, and Hela cancer cell lines.

Compound	IC50% µM		
	Hela	MCF7	HCT116
**9a**	2.59 ± 0.16	6.39 ± 0.46	15.26 ± 1.46
**9b**	13.96 ± 1.25	22.35 ± 3.18	18.01 ± 1.16
**9c**	26.63 ± 3.37	59.04 ± 5.52	43.17 ± 3.26
**9d**	19.59 ± 1.68	13.01 ± 1.33	17.46 ± 0.98
**9e**	48.45 ± 4.20	20.31 ± 3.21	16.58 ± 1.34
**9f**	20.44 ± 1.74	35.56 ± 4.64	16.78 ± 0.76
**9g**	28.26 ± 4.23	26.28 ± 2.85	40.02 ± 3.19
**9h**	7.92 ± 1.11	9.70 ± 1.00	9.69 ± 0.56
**14a**	23.05 ± 1.63	20.72 ± 1.73	25.82 ± 2.58
**14b**	20.98 ± 2.18	21.39 ± 2.05	36.29 ± 1.83
**14c**	41.27 ± 2.79	39.34 ± 3.74	36.73 ± 2.02
**14d**	21.07 ± 2.03	37.21 ± 2.48	31.14 ± 3.54
**14e**	37.33 ± 3.32	34.36 ± 2.59	37.77 ± 2.63
**14f**	46.14 ± 3.70	19.56 ± 1.26	39.96 ± 4.32
**14g**	12.65 ± 1.53	4.66 ± 0.38	1.98 ± 0.17
**14h**	31.69 ± 2.48	38.12 ± 2.76	3.69 ± 0.11
**Doxorubicin**	2.35 ± 0.08	4.57 ± 0.28	2.11 ± 0.20

Data are presented as the mean of the IC_50_ values ± SD from three different experiments.

**Table 2:** Cell cycle analyses for compounds **9a** and **14g** against MCF7, HCT-116, and Hela cancer cell lines.

Compound/Cell Line	DNA Content			Comment
	%G0-G1	%S	%G2/M	
**9a/Hela**	59.1	29.54	11.36	cell growth arrest@ S
**Hela control**	61.39	24.11	14.5	---
**14g/MCF7**	41.25	33.81	24.94	cell growth arrest@ G2/M
**MCF7 control**	46.12	39.02	14.86	---
**14g/HCT-116**	51.94	34.82	13.24	cell growth arrest@ S
**HCT-116 control**	55.87	26.49	17.64	---

**Table 3:** Apoptosis analysis for **9a** and **14g** against MCF7, HCT-116, and Hela cancer cell lines.

Compound	Apoptosis			Necrosis
	Total	Early	Late	
**9a/Hela**	42.19	23.21	12.82	6.16
**Hela control**	2.33	0.49	0.12	1.72
**14g/MCF7**	22.89	13.07	7.55	2.27
**MCF7 control**	1.49	0.44	0.27	0.78
**14g/HCT-116**	26.71	16.01	6.95	3.75
**HCT-116 control**	2.51	0.63	0.19	1.69

**Table 4:** In vitro anticancer activities of **9a** and **14g** against human normal WI-38 cells.

Compound	In Vitro Cytotoxicity on WI-38 (IC_50,_ µM) ^a^
**9a**	26.44 ± 3.23
**14g**	21.81 ± 2.96
**Doxorubicin**	15.60 ± 0.37

**^a^** Data are presented as the mean of the IC_50_ values ± SD from three different experiments.

**Table 5:** CDK2 and CDK9 inhibitory activity (IC_50,_ µM) for **9a**, **14g** and ribociclib.

Compound	IC_50_ (µM)	
	CDK2	CDK9
**9a**	1.630 ± 0.009	0.262 ± 0.013
**14g**	0.460 ± 0.024	0.801 ± 0.041
**Ribociclib**	0.068 ± 0.004	0.050 ± 0.003

**Table 6:** SwissADME prediction of physicochemical properties and bioavailability of **9a**–**9h** and **14a**–**h**.

Compound	Fraction Csp3	Rotatable Bonds	H-bond Acceptors	H-bond Donors	MR	TPSA	Consensus Log P	ESOL Class	GI Absorption	BBB Permeant	Pgp Substrate	Lipinski Violations	Veber Violations	Bioavailability Score	PAINS Alerts	Brenk Alerts
**9a**	0.04	4	3	0	116.23	39.94	4.97	Poorly soluble	High	Yes	Yes	1	0	0.55	0	0
**9b**	0.08	4	3	0	121.19	39.94	5.29	Poorly soluble	High	No	Yes	1	0	0.55	0	0
**9c**	0.08	5	4	0	122.72	49.17	4.91	Poorly soluble	High	Yes	Yes	1	0	0.55	0	0
**9d**	0.04	4	4	0	116.19	39.94	5.27	Poorly soluble	High	No	Yes	1	0	0.55	0	0
**9e**	0.04	4	3	0	121.24	39.94	5.49	Poorly soluble	High	No	Yes	1	0	0.55	0	0
**9f**	0.04	4	3	0	123.93	39.94	5.57	Poorly soluble	High	No	Yes	1	0	0.55	0	0
**9g**	0.04	5	5	0	125.05	85.76	4.16	Poorly soluble	High	No	No	0	0	0.55	0	2
**9h**	0.11	6	5	0	129.21	58.4	4.92	Poorly soluble	High	No	Yes	0	0	0.55	0	0
**14a**	0.03	5	4	0	146.38	63.73	6.03	Poorly soluble	Low	No	No	1	0	0.55	0	0
**14b**	0.06	5	4	0	151.35	63.73	6.38	Poorly soluble	Low	No	No	1	0	0.55	0	0
**14c**	0.06	6	5	0	152.87	72.96	6	Poorly soluble	Low	No	No	2	0	0.17	0	0
**14d**	0.03	5	4	0	151.39	63.73	6.55	Poorly soluble	Low	No	No	2	0	0.17	0	0
**14e**	0.03	5	4	0	154.08	63.73	6.63	Poorly soluble	Low	No	No	2	0	0.17	0	0
**14f**	0.09	6	4	0	160.59	66.97	5.99	Poorly soluble	Low	No	No	2	0	0.17	0	0
**14g**	0.03	5	5	1	148.4	83.96	5.64	Poorly soluble	Low	No	No	1	0	0.55	0	0
**14h**	0.09	7	6	0	159.36	82.19	5.94	Poorly soluble	Low	No	No	1	0	0.55	0	0

## Data Availability

Not applicable.

## Supplementary Materials

The following supporting information can be downloaded at: https://www.mdpi.com/article/10.3390/molecules28176428/s1, Figure S1−S64: NMR spectra; Figure S65−S80: IR spectra; Figure S81−S94: Mass spectra.

## Sample Availability

Samples of the compounds are not available from the authors.
