# Supplementary material for: Synthesis and In Vitro Anticancer Activity of Novel 4-Aryl-3-(4-methoxyphenyl)-1-phenyl-1H-pyrazolo[3,4-b]pyridines Arrest Cell Cycle and Induce Cell Apoptosis by Inhibiting CDK2 and/or CDK9
Source: Molecules. 2023 Sep 4;28(17):6428. doi: 10.3390/molecules28176428 (PMC10490123; doi:10.3390/molecules28176428)
Supplement: Supplementary file 1 [file molecules-28-06428-s001.zip › molecules-2496828-supplementary.pdf]

Supplementary data

Synthesis and in vitro anticancer activity of novel 4-aryl-3-(4-methoxyphenyl)-1-phenyl-1*H*-pyrazolo[3,4-*b*]pyridines arrest cell cycle and induce cell apoptosis by inhibiting CDK2 and/or CDK9

Basma S. Almansour [1], Faizah A. Binjubair [1], Marwa Alsulaimany [2], Alaa A.-M. Abdel-Aziz [1], Sara T. Al-Rashood [1, *]

[1] Department of Pharmaceutical Chemistry, College of Pharmacy, King Saud University, P.O. Box 2457, Riyadh 11451, Saudi Arabia; 443203434@student.ksu.edu.sa (B.S.A.); fbinjubair@ksu.edu.sa (F.A.B.); almoenes@ksu.edu.sa (A.A.-M.A.-A.)
[2] Department of Pharmacognosy & Pharmaceutical Chemistry, College of Pharmacy, Taibah University, Medina, 42353, Saudi Arabia; msulaimany@taibahu.edu.sa (M. A.)
*Correspondence: salrashood@ksu.edu.sa

Keywords: Pyrazolo[3,4-*b*]pyridines; Antiproliferative; Cell cycle; Apoptosis; CDK2; CDK9

Contents

Figure S1. ^{1}H NMR of compound **9a**, full spectrum ……………………...……. 1

Figure S2. ^{1}H NMR of compound **9a**, aromatic region ………………………….. 2

Figure S3. ^{13}C NMR of compound **9a**, full spectrum ……………………….…. 3

Figure S4. ^{13}C NMR of compound **9a**, aromatic region ……………………….…. 4

Figure S5. ^{1}H NMR of compound **9b**, full spectrum …………………………….. 5

Figure S6. ^{1}H NMR of compound **9b**, aromatic region ………………………….. 6

Figure S7. ^{13}C NMR of compound **9b**, full spectrum…………………………….. 7

Figure S8. ^{13}C NMR of compound **9b**, aromatic region………………………….. 8

Figure S9. ^{1}H NMR of compound **9c**, full spectrum……………………….…...... 9

Figure S10. ^{1}H NMR of compound **9c**, aliphatic region………………….............. 10

Figure S11. ^{1}H NMR of compound **9c**, aromatic region ………………………… 11

Figure S12. ^{13}C NMR of compound **9c**, full spectrum………………………........ 12

Figure S13. ^{13}C NMR of compound **9c**, aromatic region ………………………... 13

Figure S14. ^{1}H NMR of compound **9d**, full spectrum ……………………........ 14

Figure S15. ^{1}H NMR of compound **9d**, aromatic region ……………………........ 15

Figure S16. ^{13}C NMR of compound **9d**, full spectrum ………………………….. 16

Figure S17. ^{13}C NMR of compound **9d**, aromatic region ……………………....... 17

Figure S18. ^{1}H NMR of compound **9e**, full spectrum …………………………… 18

Figure S19. ^{1}H NMR of compound **9e**, aromatic region ………………………… 19

Figure S20. ^{1}H NMR of compound **9f**, full spectrum ……………………………. 20

Figure S21. ^{1}H NMR of compound **9f**, aromatic region …………………………. 21

Figure S22. ^{1}H NMR of compound **9g**, full spectrum ……………………........ 22

Figure S23. ^{1}H NMR of compound **9g**, aromatic region ……………………........ 23

Figure S24. ^{1}H NMR of compound **9h**, full spectrum ………………………..... 24

Figure S25. ^{1}H NMR of compound **9h**, aromatic region………………………… 25

Figure S26. ^{13}C NMR of compound **9h**, full spectrum…………………………..... 26

Figure S27. ^{13}C NMR of compound **9h**, aliphatic region………………………… 27

Figure S28. ^{13}C NMR of compound **9h**, aromatic region………………………… 28

Figure S29. ^{1}H NMR of compound **14a**, full spectrum………………………... 29

Figure S30. ^{1}H NMR of compound **14a**, aromatic region……………………….. 30

Figure S31. ^{13}C NMR of compound **14a**, full spectrum………………………….. **31**
Figure S32. ^{13}C NMR of compound **14a**, aromatic region……………………….. **32**
Figure S33. ^{13}C NMR of **14a**, aromatic region 128-131 *ppm*…………………….. **33**
Figure S34. ^{1}H NMR of compound **14b**, full spectrum…………………………... **34**
Figure S35. ^{1}H NMR of compound **14b**, aromatic region………………………... **35**
Figure S36. ^{13}C NMR of compound **14b**, full spectrum………………………….. **36**
Figure S37. ^{13}C NMR of compound **14b**, aromatic region……………………….. **37**
Figure S38. ^{1}H NMR of compound **14c**, full spectrum…………………………... **38**
Figure S39. ^{1}H NMR of compound **14c**, aromatic region………………………... **39**
Figure S40. ^{13}C NMR of compound **14c**, full spectrum………………………….. **40**
Figure S41. ^{13}C NMR of compound **14c**, aromatic region……………………….. **41**
Figure S42. ^{1}H NMR of compound **14d**, full spectrum…………………………... **42**
Figure S43. ^{1}H NMR of compound **14d**, aromatic region………………………... **43**
Figure S44. ^{13}C NMR of compound **14d**, full spectrum………………………….. **44**
Figure S45. ^{13}C NMR of compound **14d**, aromatic region……………………….. **45**
Figure S46. ^{1}H NMR of compound **14e**, full spectrum…………………………... **46**
Figure S47. ^{1}H NMR of compound **14e**, aromatic region………………………... **47**
Figure S48. ^{13}C NMR of compound **14e**, full spectrum………………………….. **48**
Figure S49. ^{13}C NMR of compound **14e**, aromatic region……………………….. **49**
Figure S50. ^{1}H NMR of compound **14f**, full spectrum………………………….... **50**
Figure S51. ^{1}H NMR of compound **14f**, aromatic region………………………… **51**
Figure S52. ^{13}C NMR of compound **14f**, full spectrum…………………………... **52**
Figure S53. ^{13}C NMR of compound **14f**, aliphatic region………………………... **53**
Figure S54. ^{13}C NMR of compound **14f**, aromatic region………………………... **54**
Figure S55. ^{1}H NMR of compound **14g**, full spectrum…………………………... **55**
Figure S56. ^{1}H NMR of compound **14g**, aromatic region………………………... **56**
Figure S57. ^{13}C NMR of compound **14g**, full spectrum………………………….. **57**
Figure S58. ^{13}C NMR of compound **14g**, aromatic region……………………….. **58**
Figure S59. ^{1}H NMR of compound **14h**, full spectrum…………………………... **59**
Figure S60. ^{1}H NMR of compound **14h**, aliphatic region……………………….. **60**
Figure S61. ^{1}H NMR of compound **14h**, aromatic region……………………….. **61**

Figure S62. ^{13}C NMR of compound **14h**, full spectrum…………………………….. **62**
Figure S63. ^{13}C NMR of compound **14h**, aliphatic region……………………….. **63**
Figure S64. ^{13}C NMR of compound **14h**, aromatic region……………………….. **64**
Figure S65. IR of compound **9a**……………………….................................... **65**
Figure S66. IR of compound **9b**……………………….................................... **66**
Figure S67. IR of compound **9c**……………………….................................... **67**
Figure S68. IR of compound **9d**……………………….................................... **68**
Figure S69. IR of compound **9e**……………………….................................... **69**
Figure S70. IR of compound **9f**……………………….................................... **70**
Figure S71. IR of compound **9g**……………………….................................... **71**
Figure S72. IR of compound **9h**……………………….................................... **72**
Figure S73. IR of compound **14a**……………………….................................. **73**
Figure S74. IR of compound **14b**……………………….................................. **74**
Figure S75. IR of compound **14c**……………………….................................. **75**
Figure S76. IR of compound **14d**……………………….................................. **76**
Figure S77. IR of compound **14e**……………………….................................. **77**
Figure S78. IR of compound **14f**……………………….................................. **78**
Figure S79. IR of compound **14g**……………………….................................. **79**
Figure S80. IR of compound **14h**……………………….................................. **80**
Figure S81. Mass of compound **9a**……………………….............................. **81**
Figure S82. Mass of compound **9b**……………………….............................. **82**
Figure S83. Mass of compound **9d**……………………….............................. **83**
Figure S84. Mass of compound **9e**……………………….............................. **84**
Figure S85. Mass of compound **9f**……………………….............................. **85**
Figure S86. Mass of compound **9h**……………………….............................. **86**
Figure S87. Mass of compound **14a**……………………….............................. **87**
Figure S88. Mass of compound **14b**……………………….............................. **88**
Figure S89. Mass of compound **14c**……………………….............................. **89**
Figure S90. Mass of compound **14d**……………………….............................. **90**
Figure S91. Mass of compound **14e**……………………….............................. **91**
Figure S92. Mass of compound **14f**……………………….............................. **92**

Figure S93. Mass of compound **14g**………………………….............................. **93**
Figure S94. Mass of compound **14h**………………………….............................. **94**
Characterization instruments…..……………………………………………… **95**

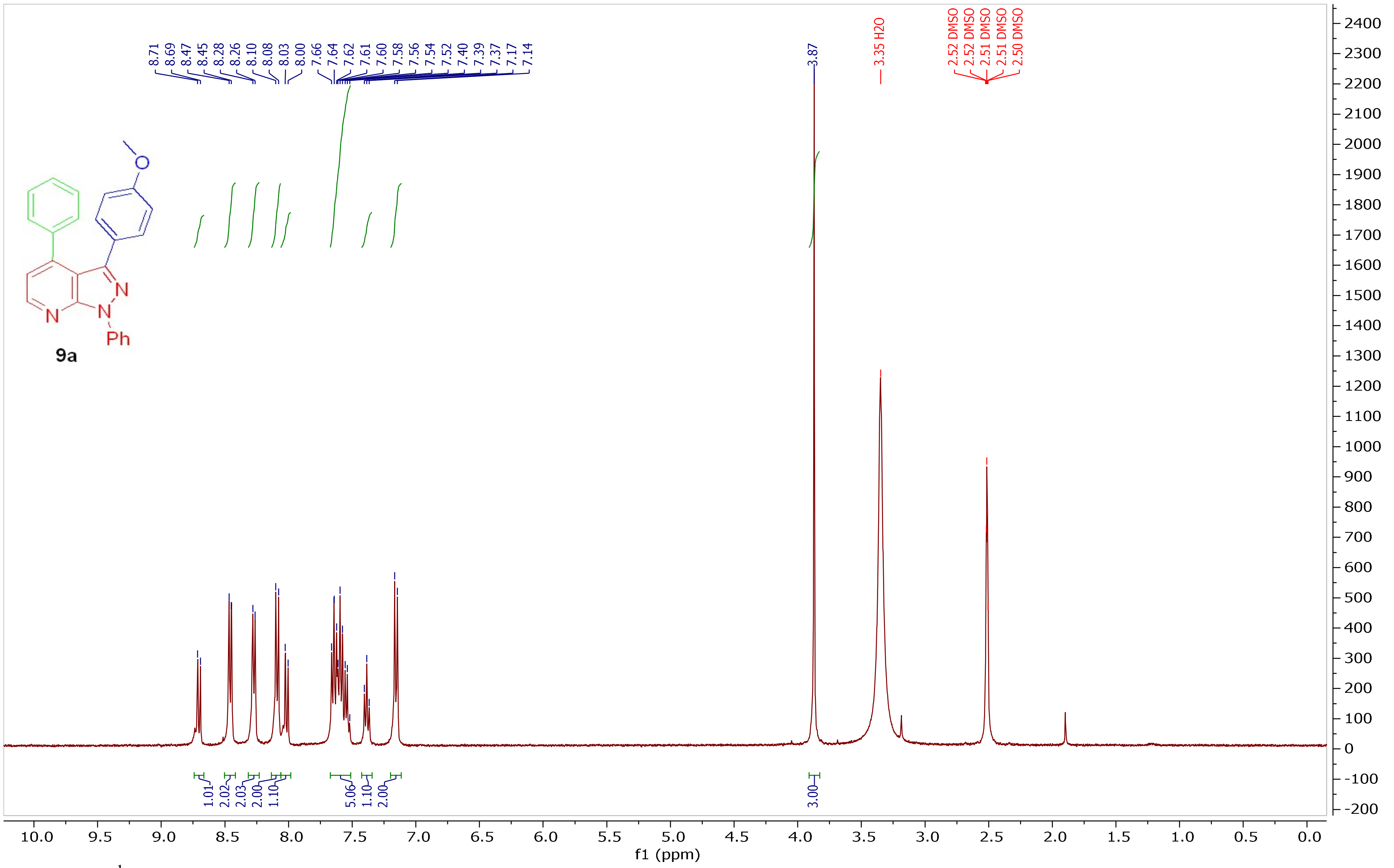

Figure S1. ^{1}H NMR of compound **9a**, full spectrum

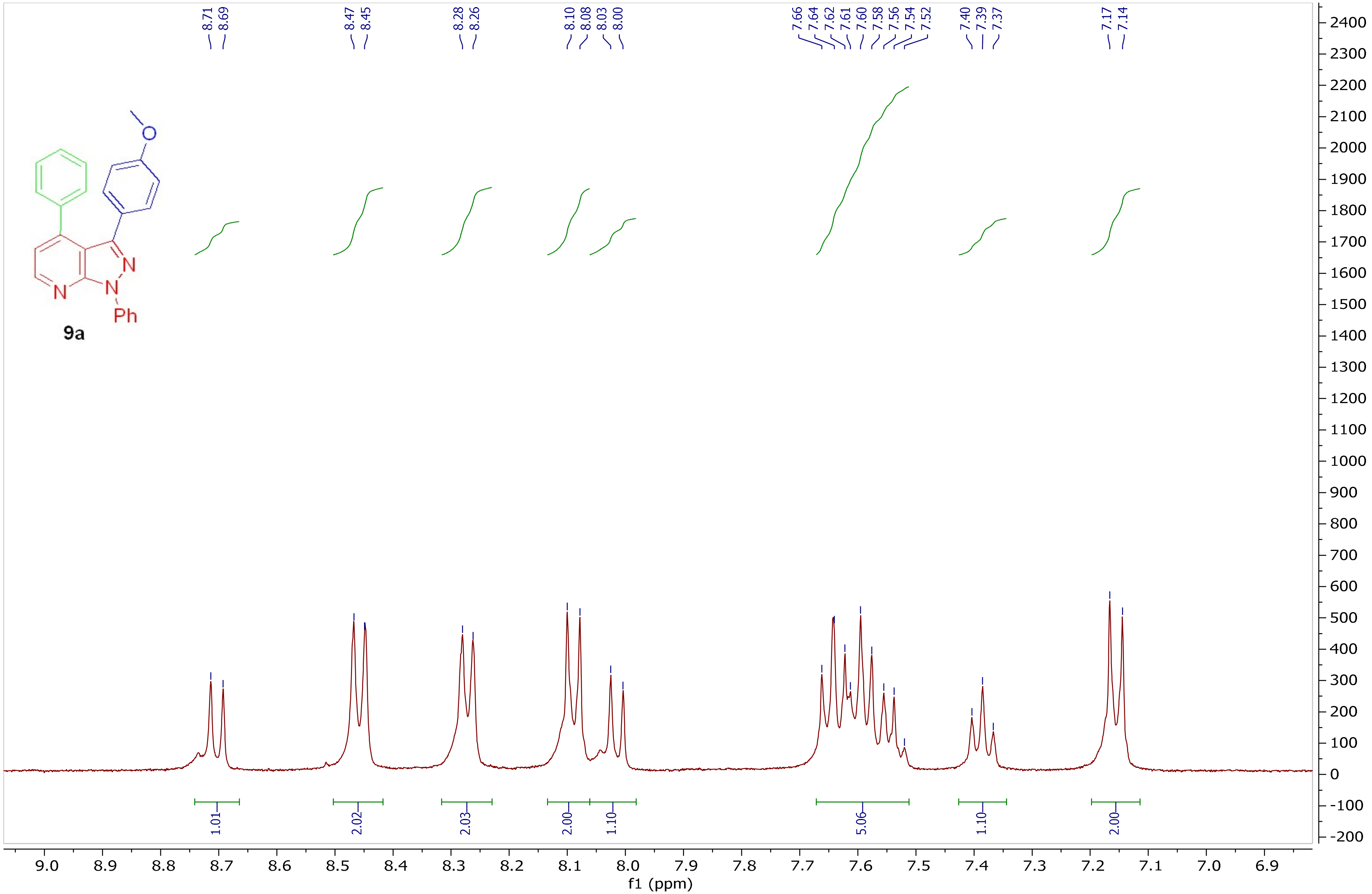

Figure S2. ^{1}H NMR of compound **9a**, aromatic region

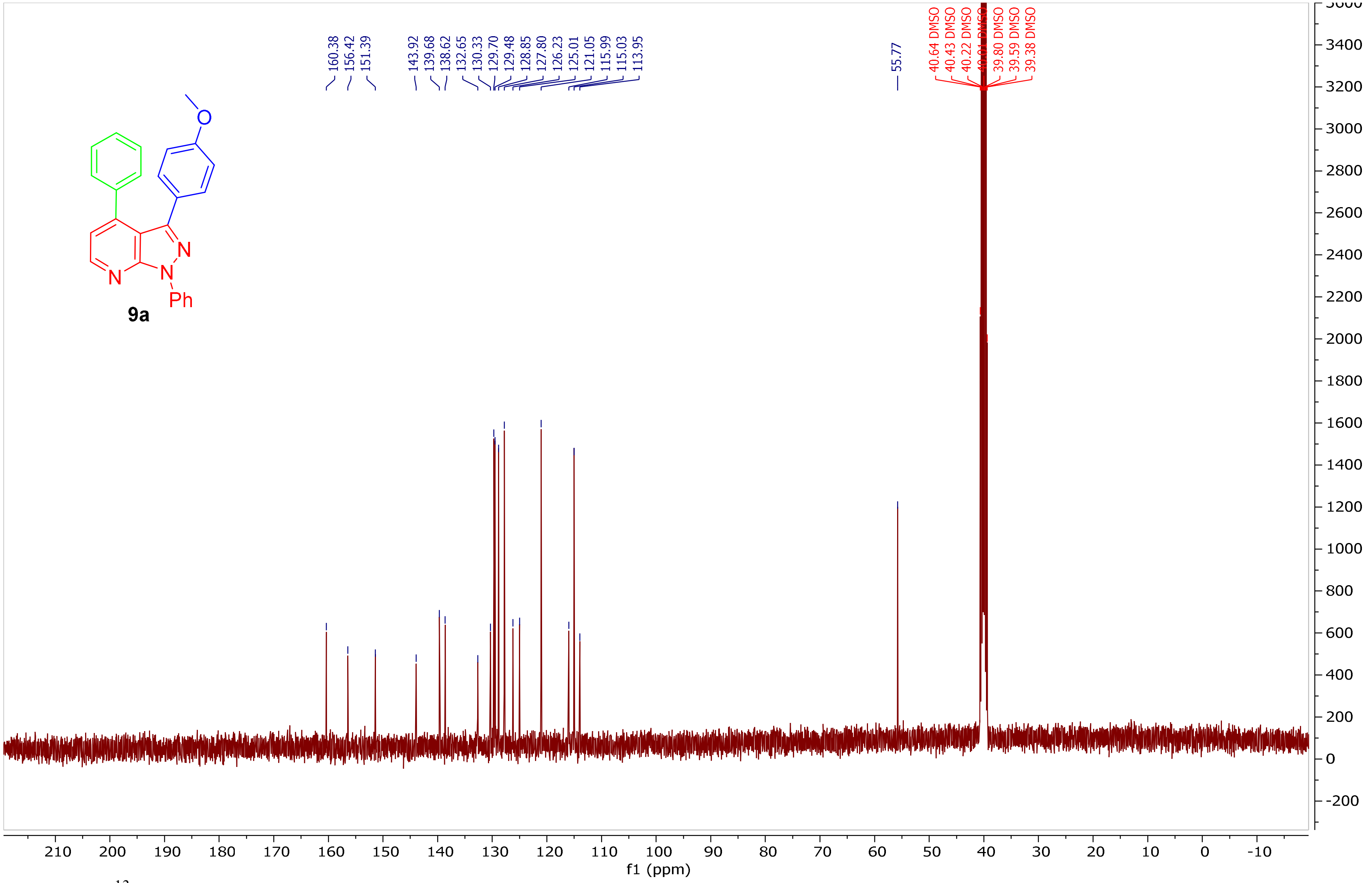

Figure S3. ^{13}C NMR of compound **9a**, full spectrum

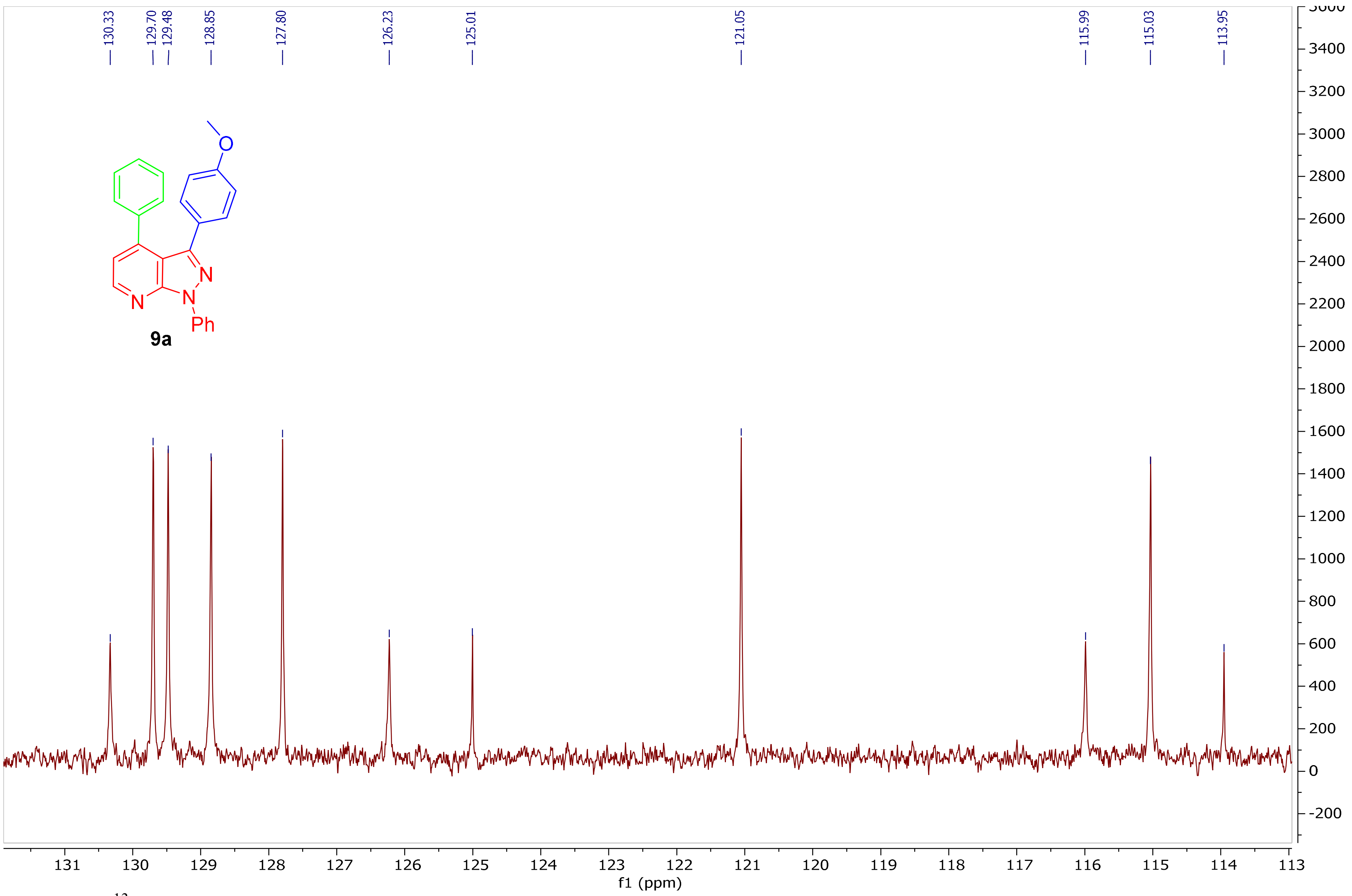

Figure S4. ^{13}C NMR of compound **9a**, aromatic region

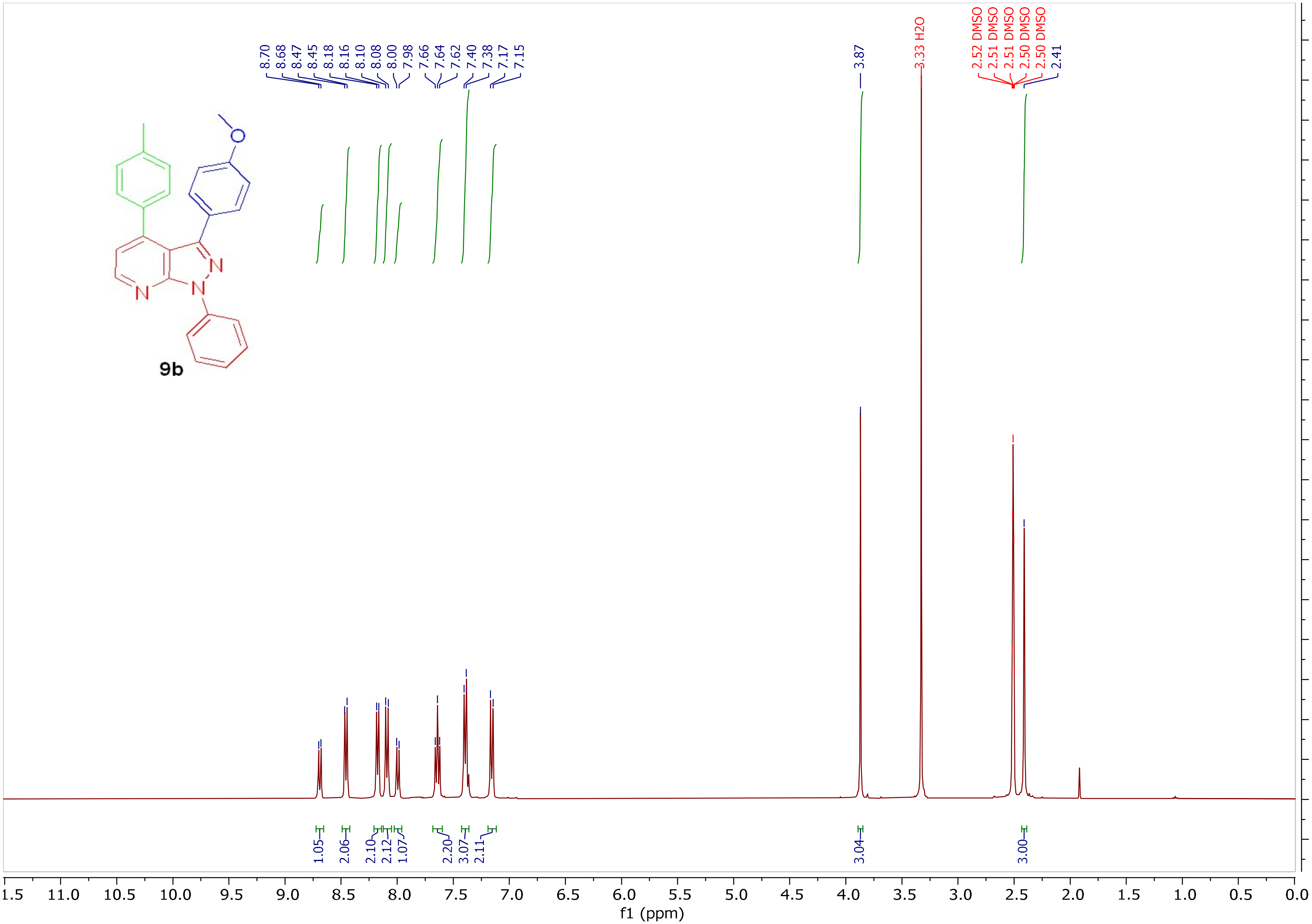

Figure S5. ^{1}H NMR of compound **9b**, full spectrum

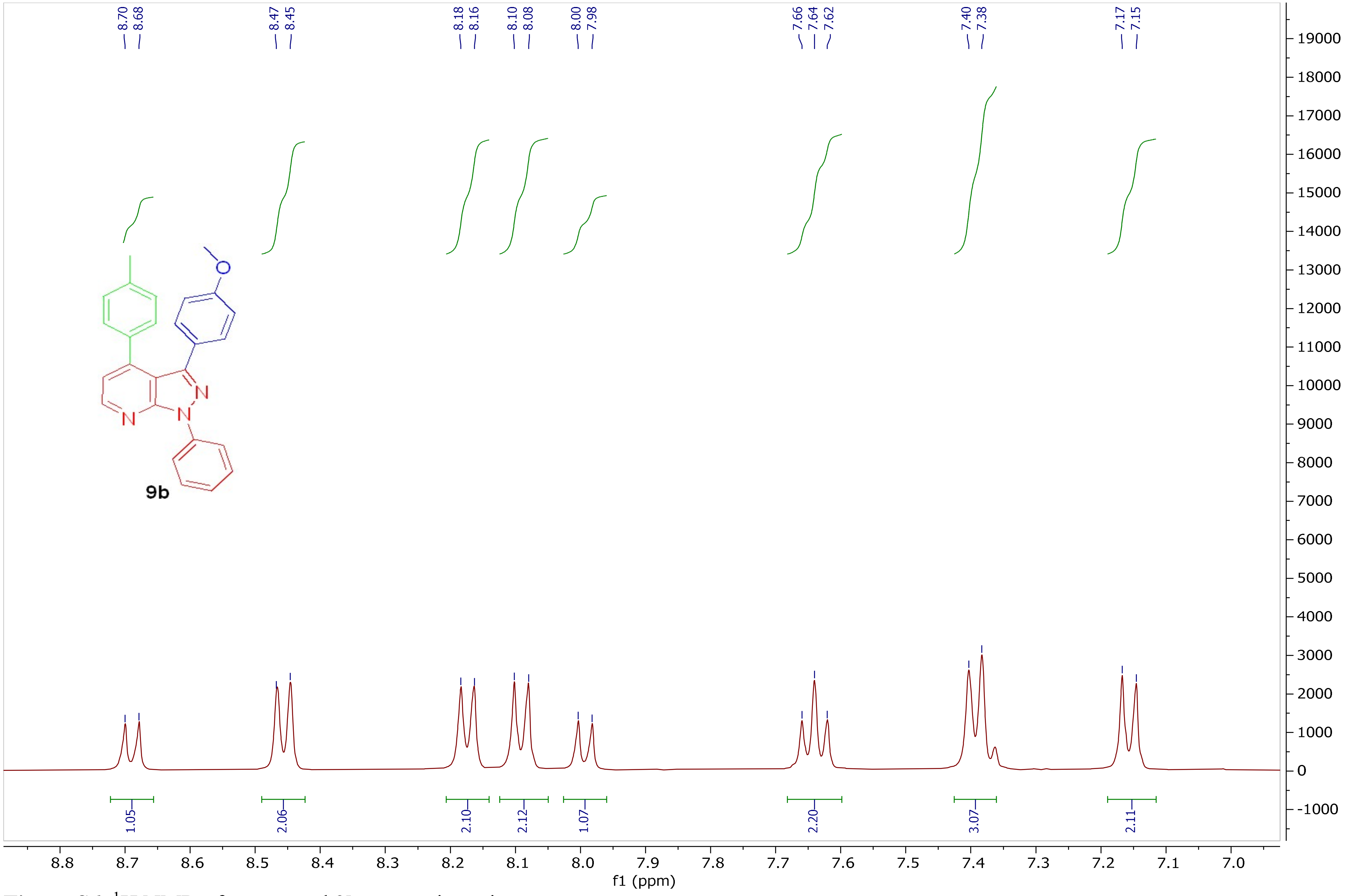

Figure S6. ^{1}H NMR of compound **9b**, aromatic region

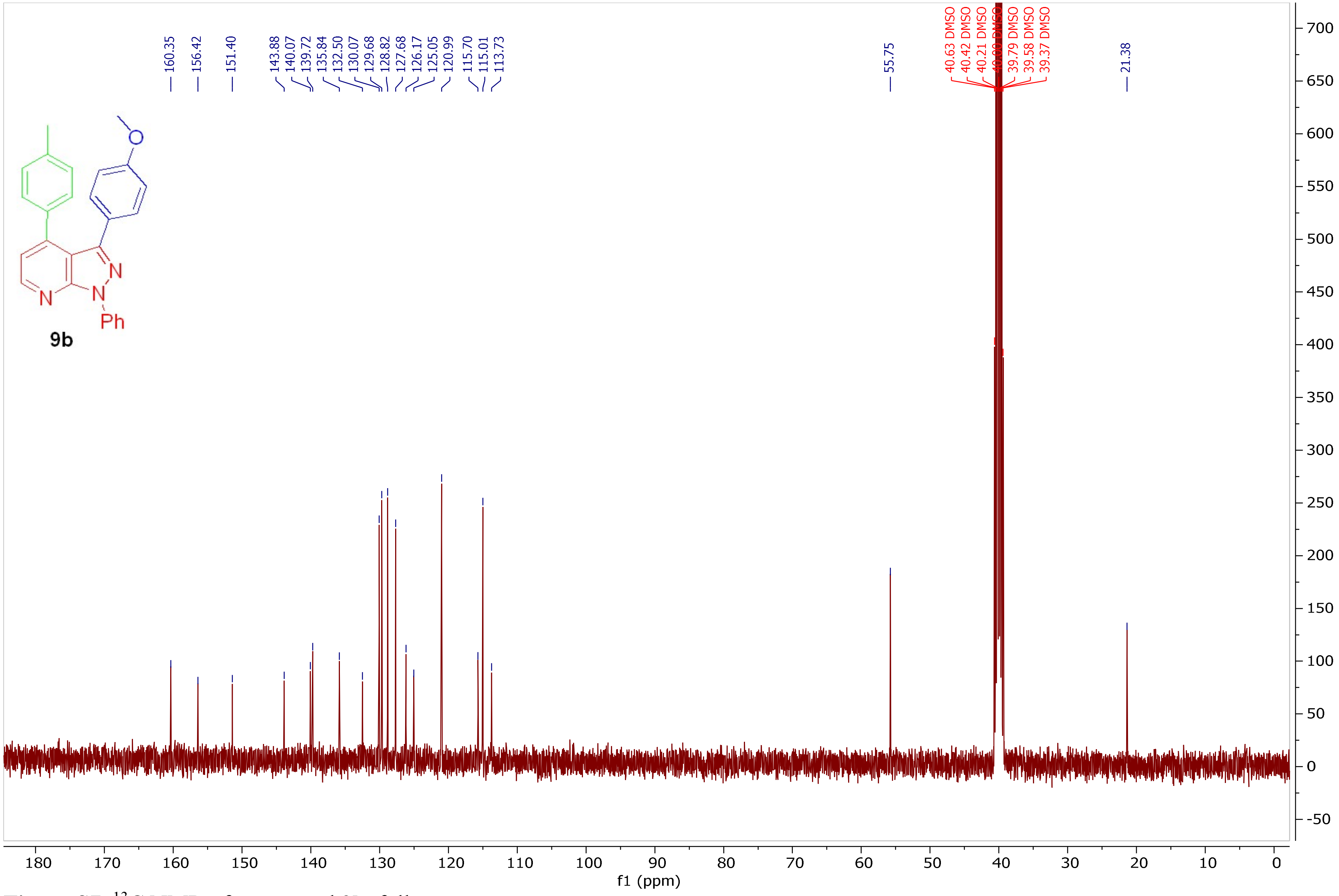

Figure S7. ^{13}C NMR of compound **9b**, full spectrum

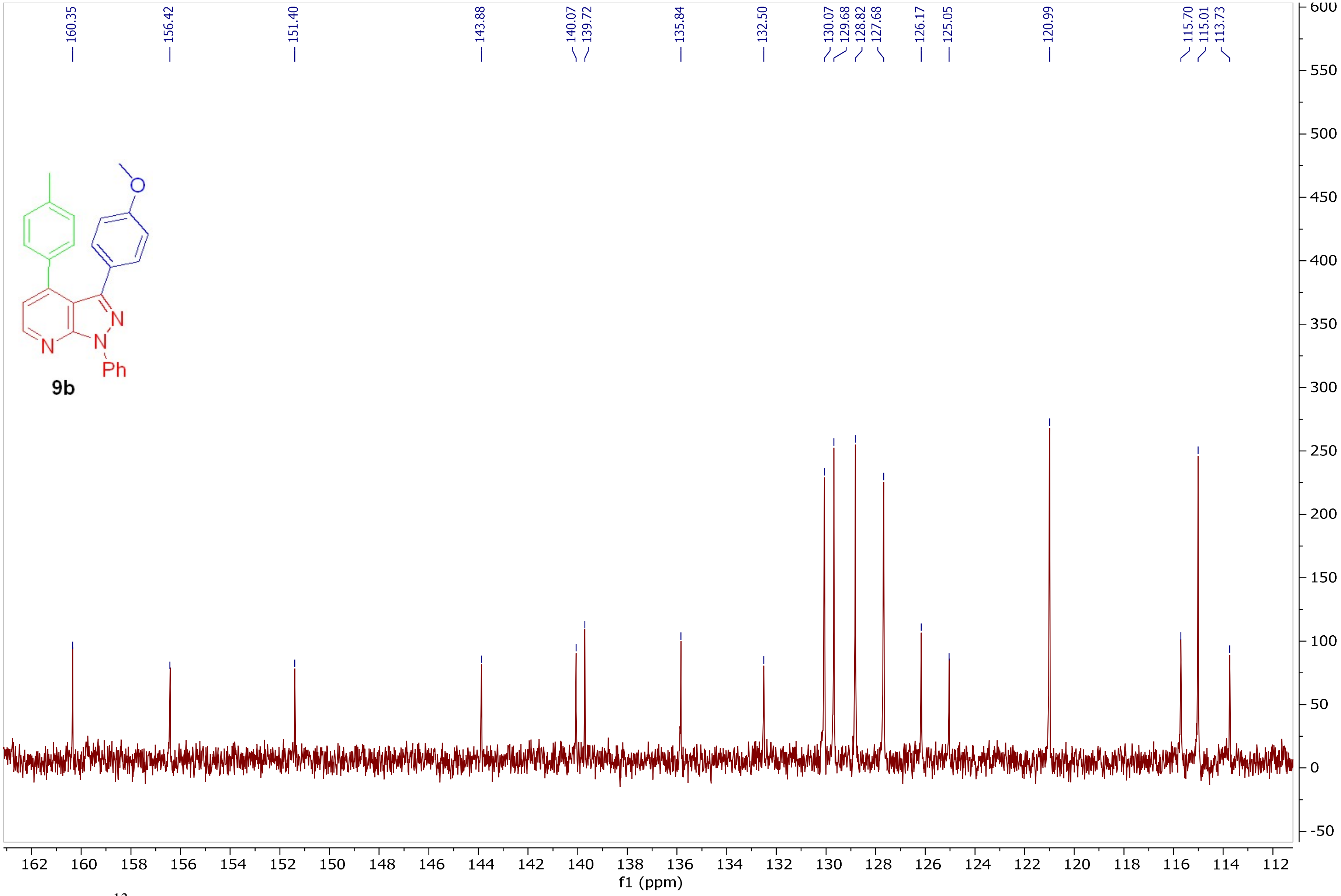

Figure S8. ^{13}C NMR of compound **9b**, aromatic region

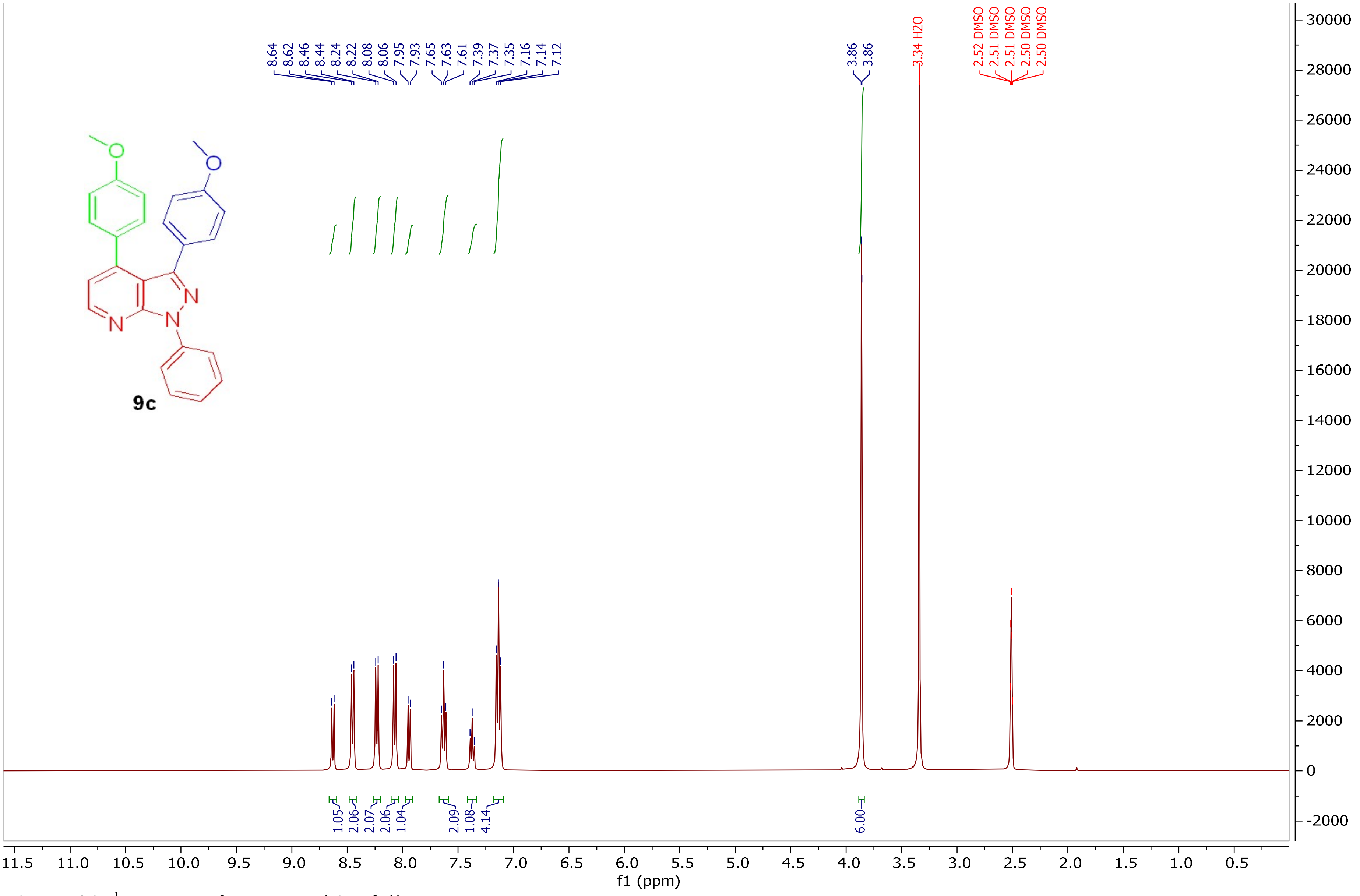

Figure S9. ^{1}H NMR of compound **9c**, full spectrum

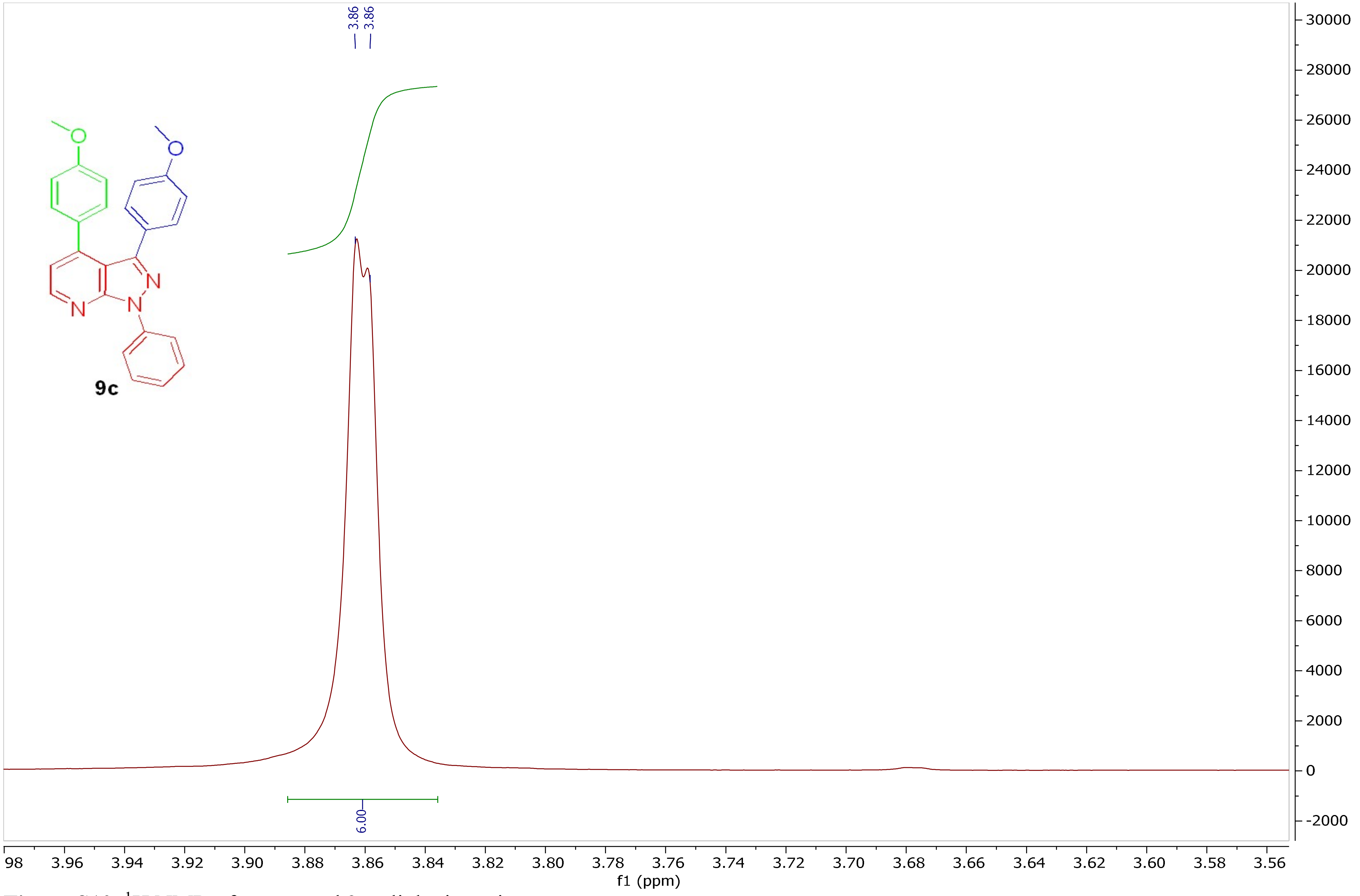

Figure S10. ^{1}H NMR of compound **9c**, aliphatic region

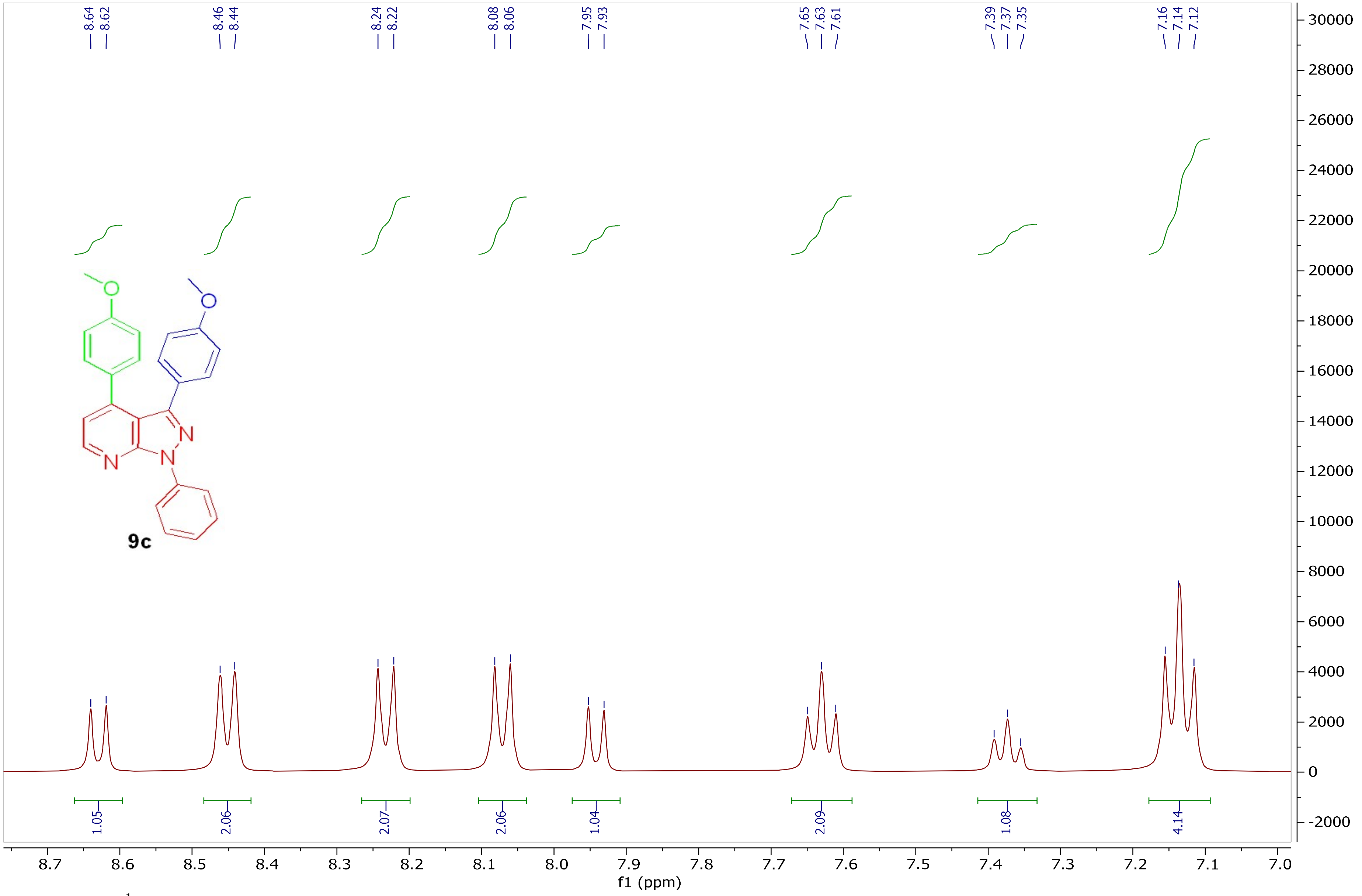

Figure S11. ^{1}H NMR of compound **9c**, aromatic region

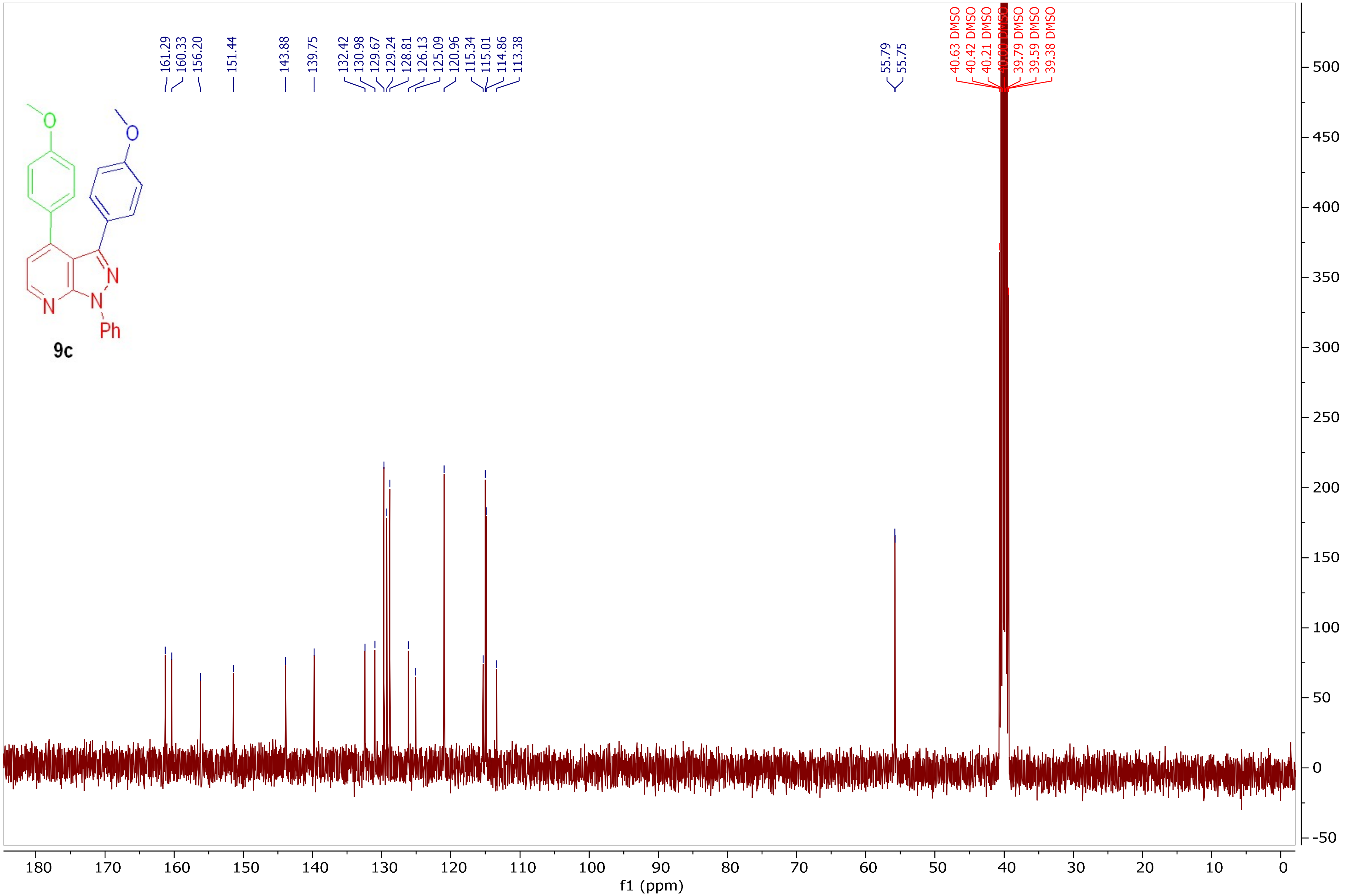

Figure S12. ^{13}C NMR of compound **9c**, full spectrum

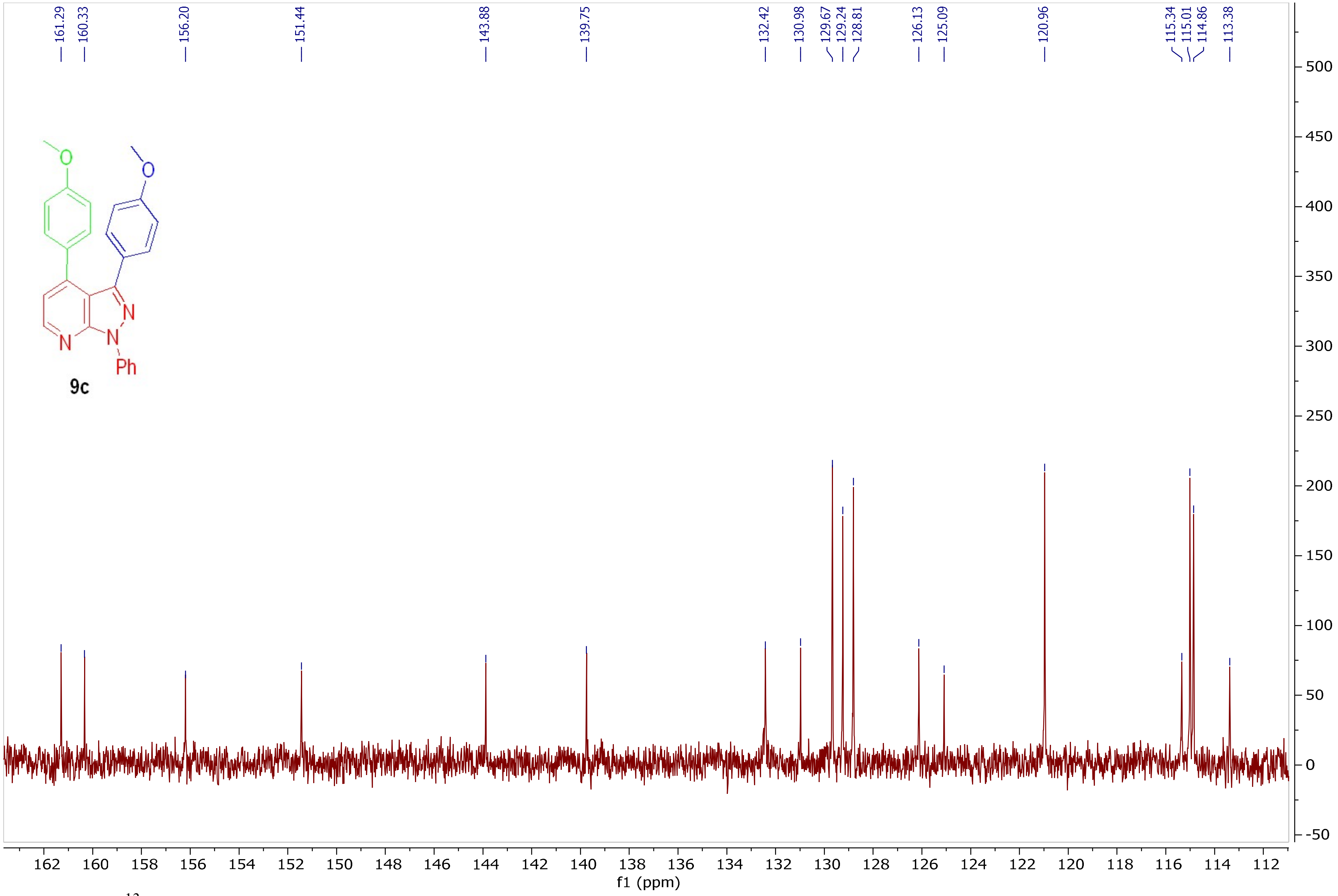

Figure S13. ^{13}C NMR of compound **9c**, aromatic region

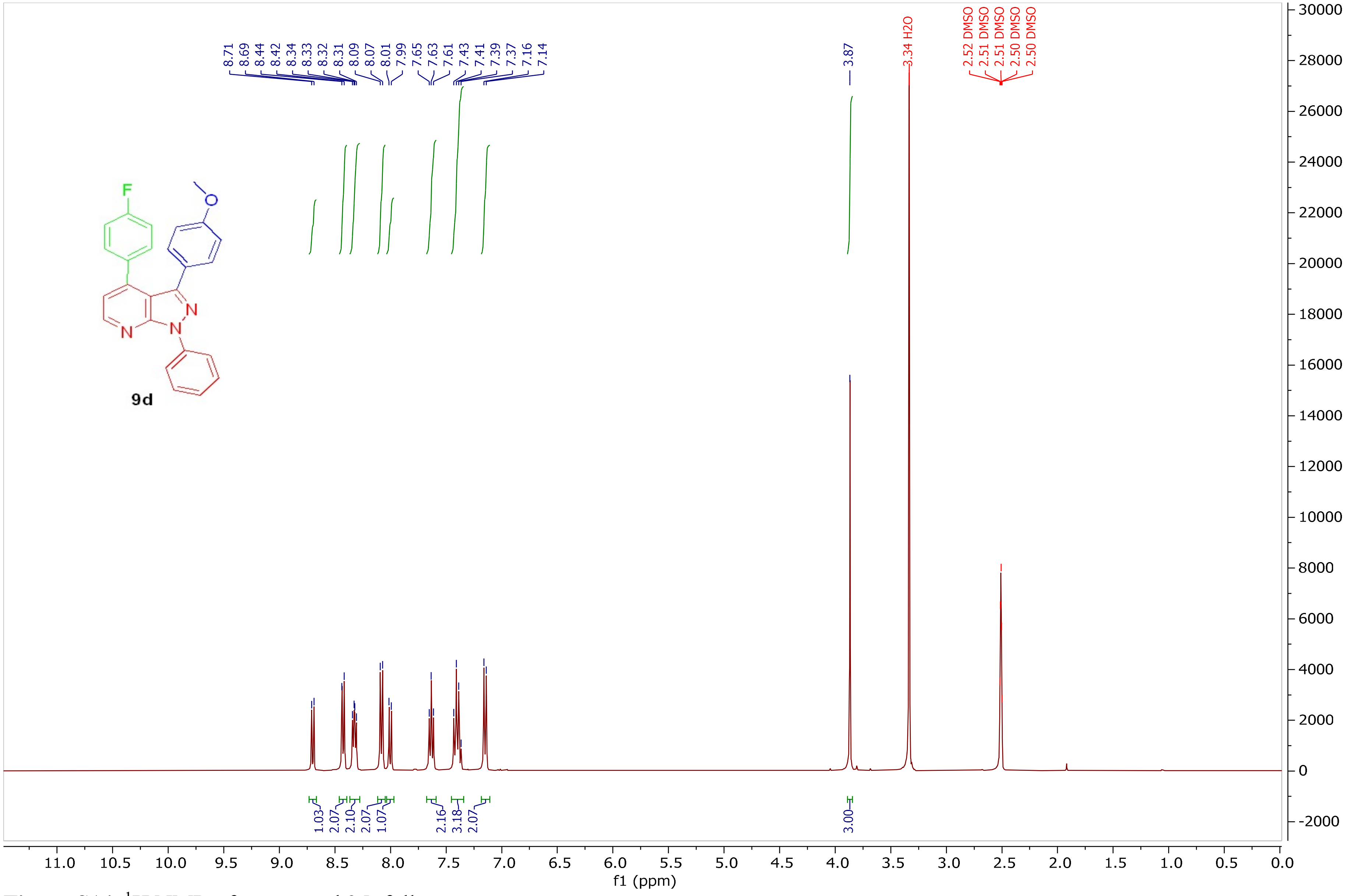

Figure S14. ^{1}H NMR of compound **9d**, full spectrum

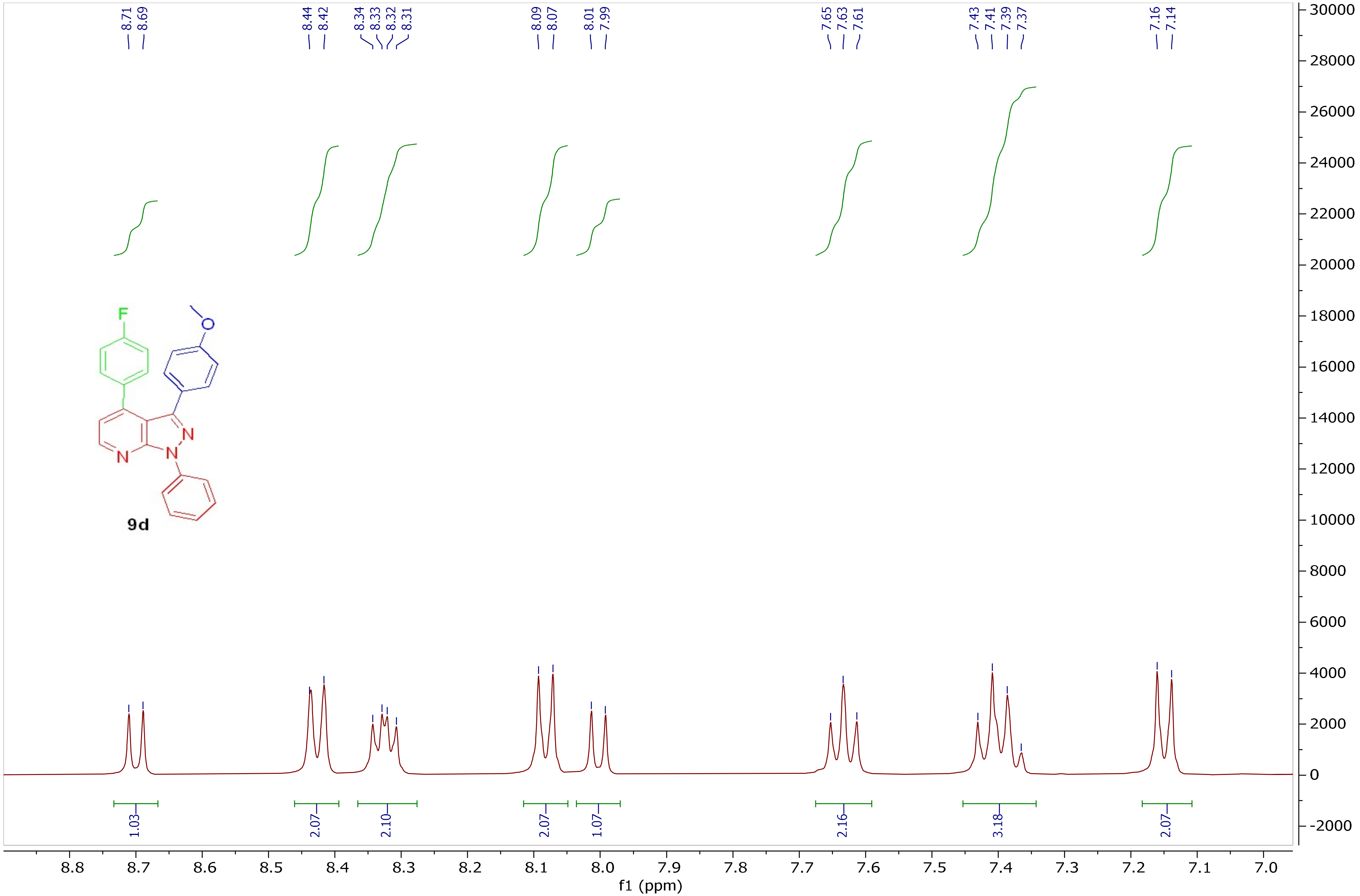

Figure S15. ^{1}H NMR of compound **9d**, aromatic region

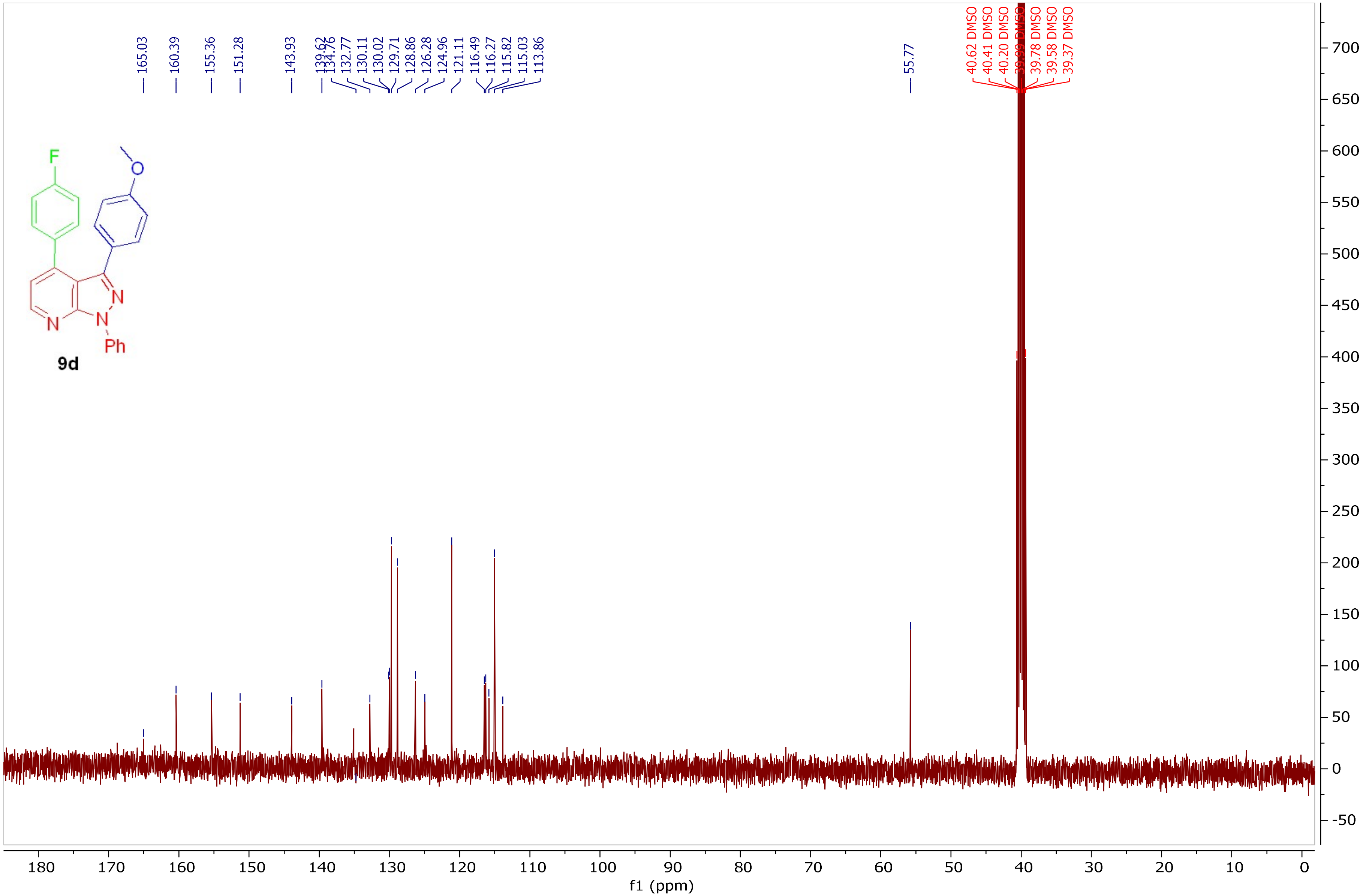

Figure S16. ^{13}C NMR of compound **9d**, full spectrum

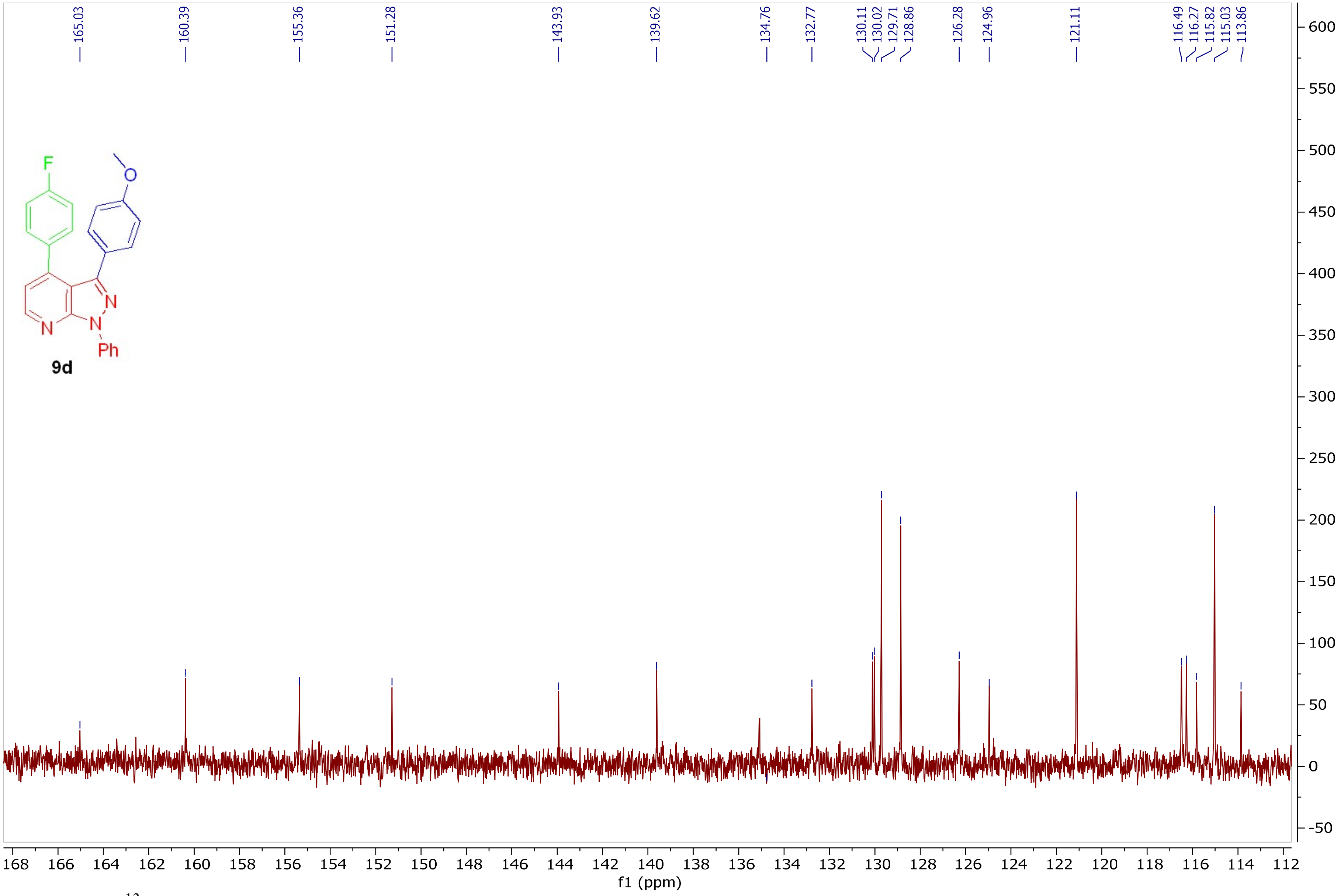

Figure S17. ^{13}C NMR of compound **9d**, aromatic region

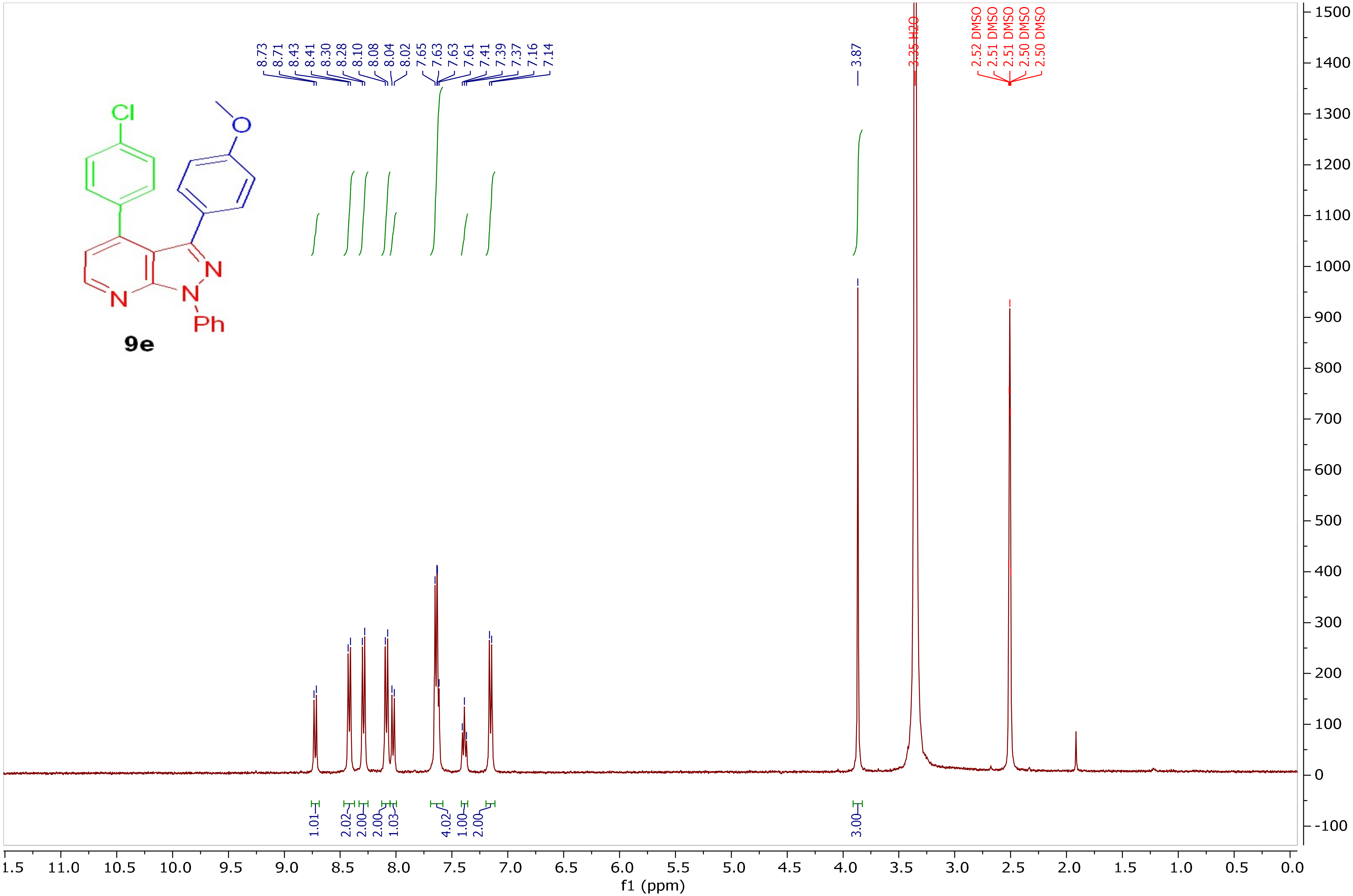

Figure S18. ¹H NMR of compound **9e**, full spectrum

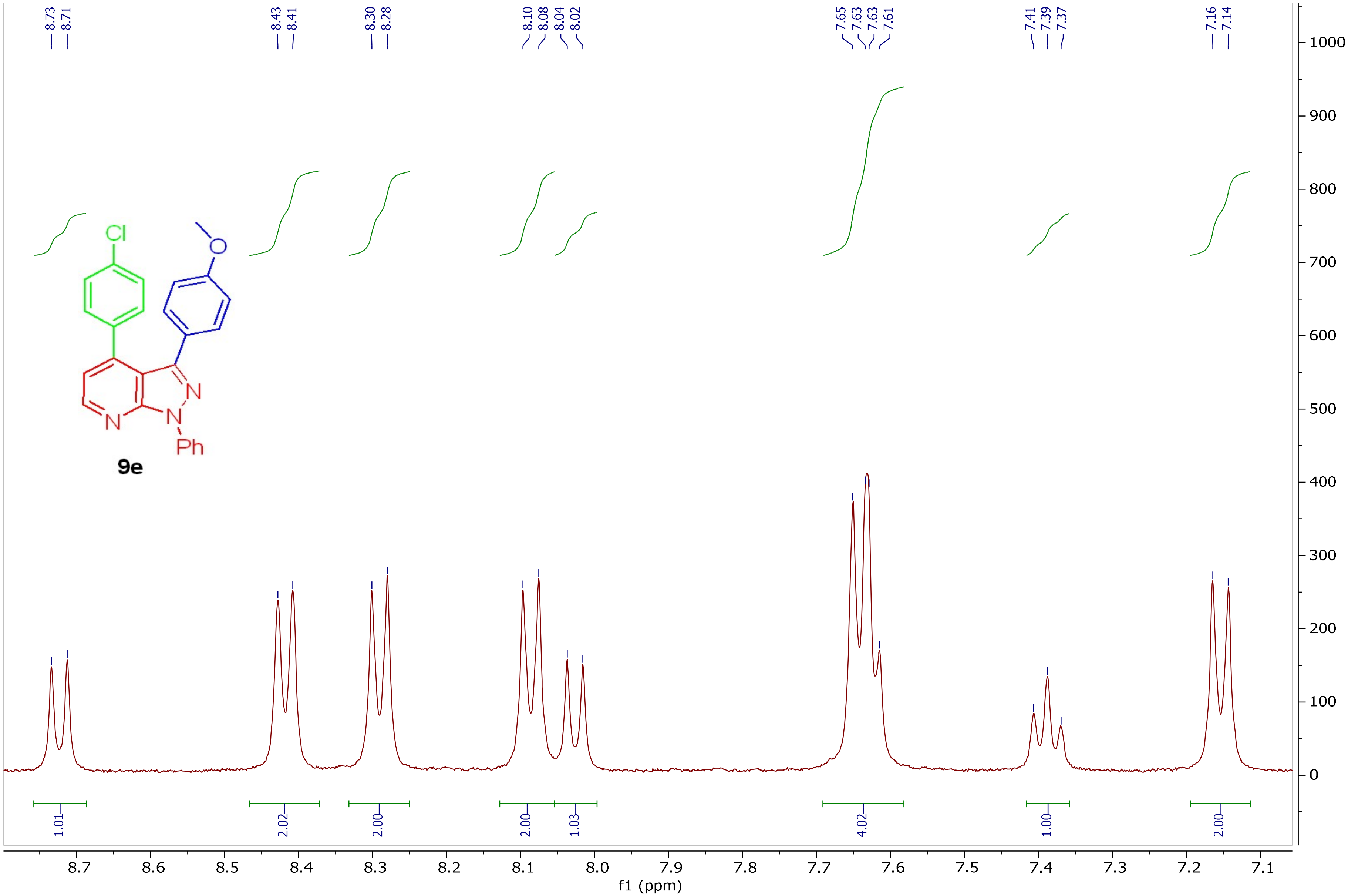

Figure S19. ^{1}H NMR of compound **9e**, aromatic region

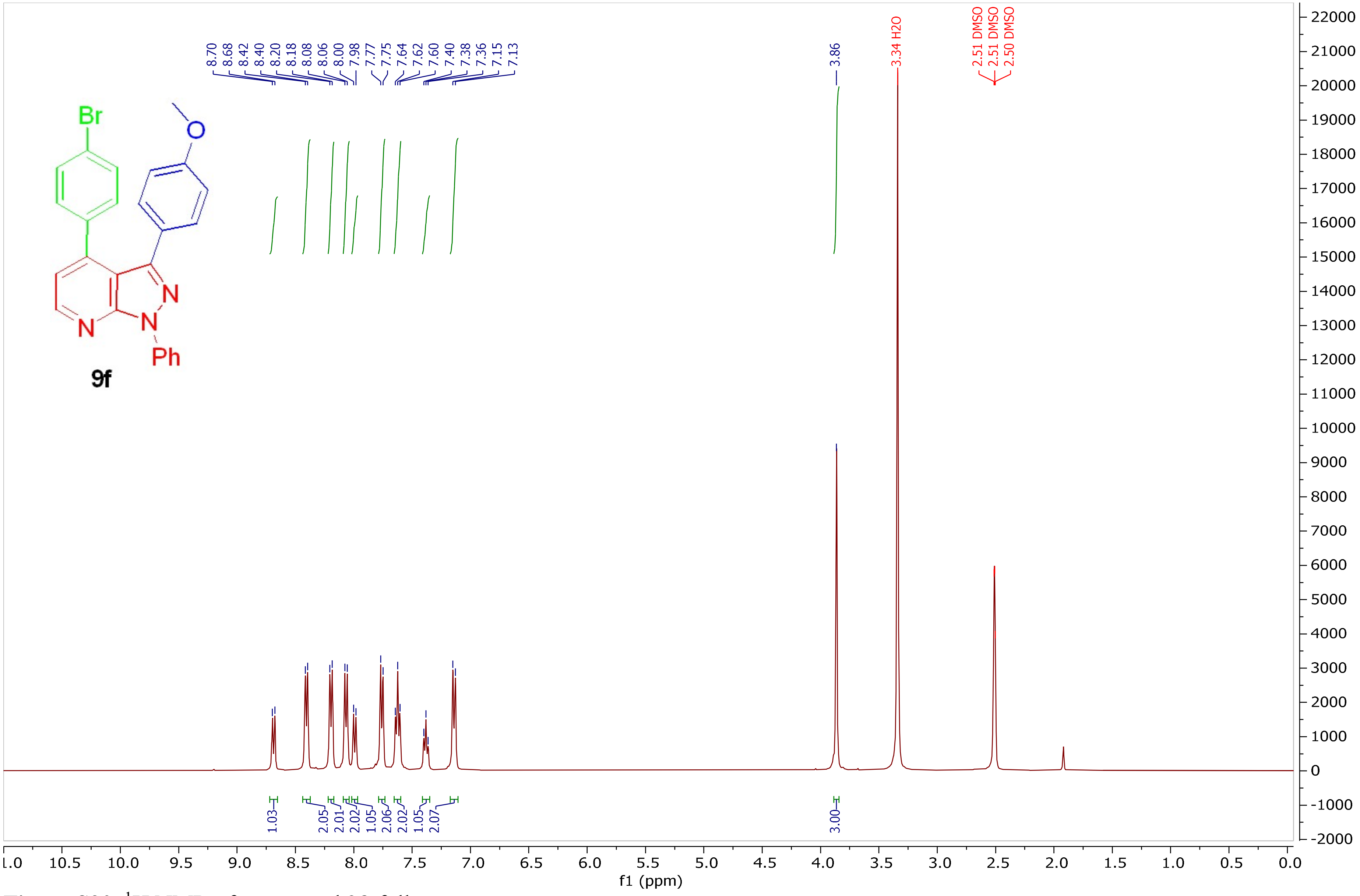

Figure S20. ^{1}H NMR of compound **9f**, full spectrum

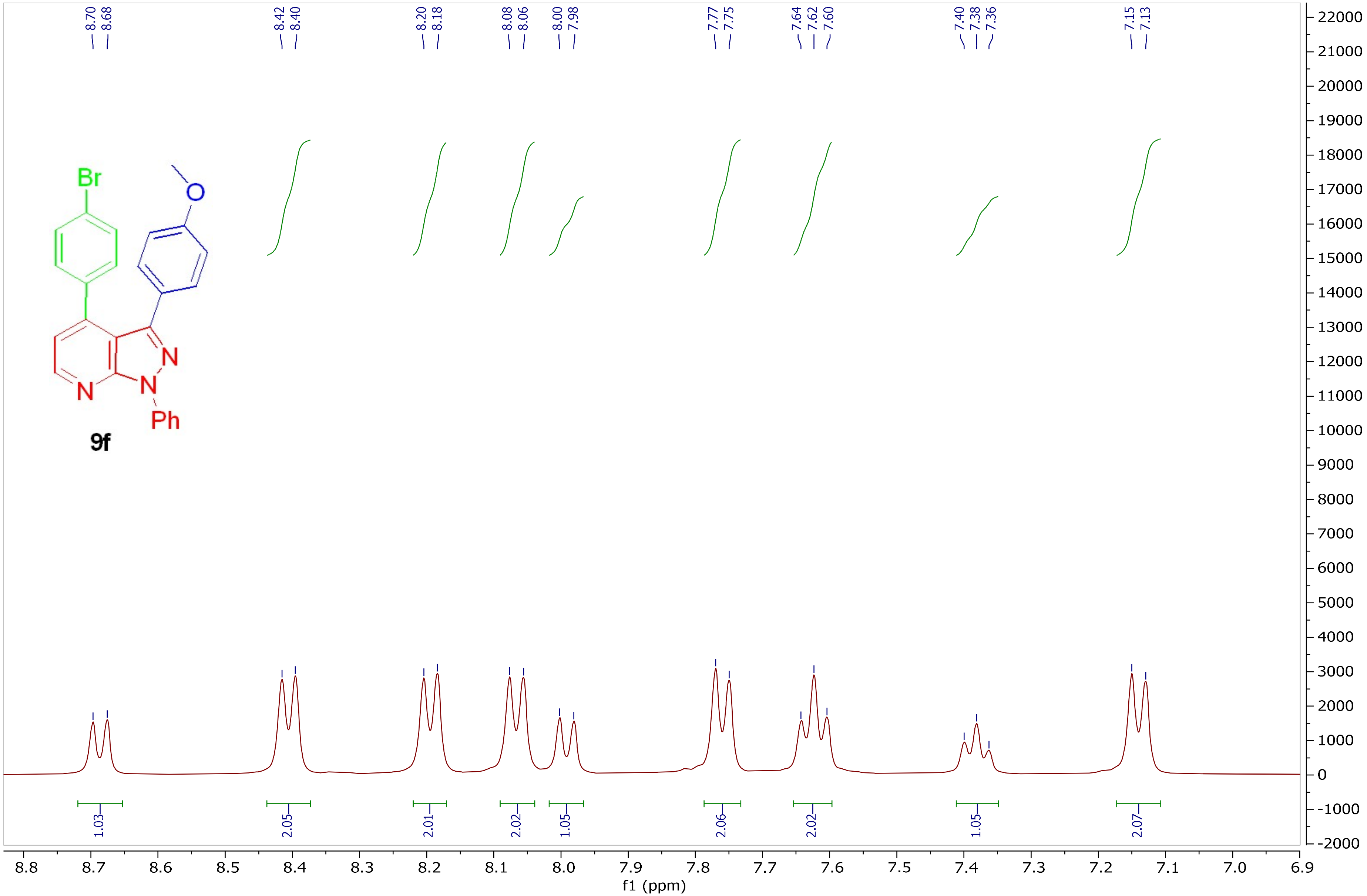

Figure S21. ^{1}H NMR of compound **9f**, aromatic region

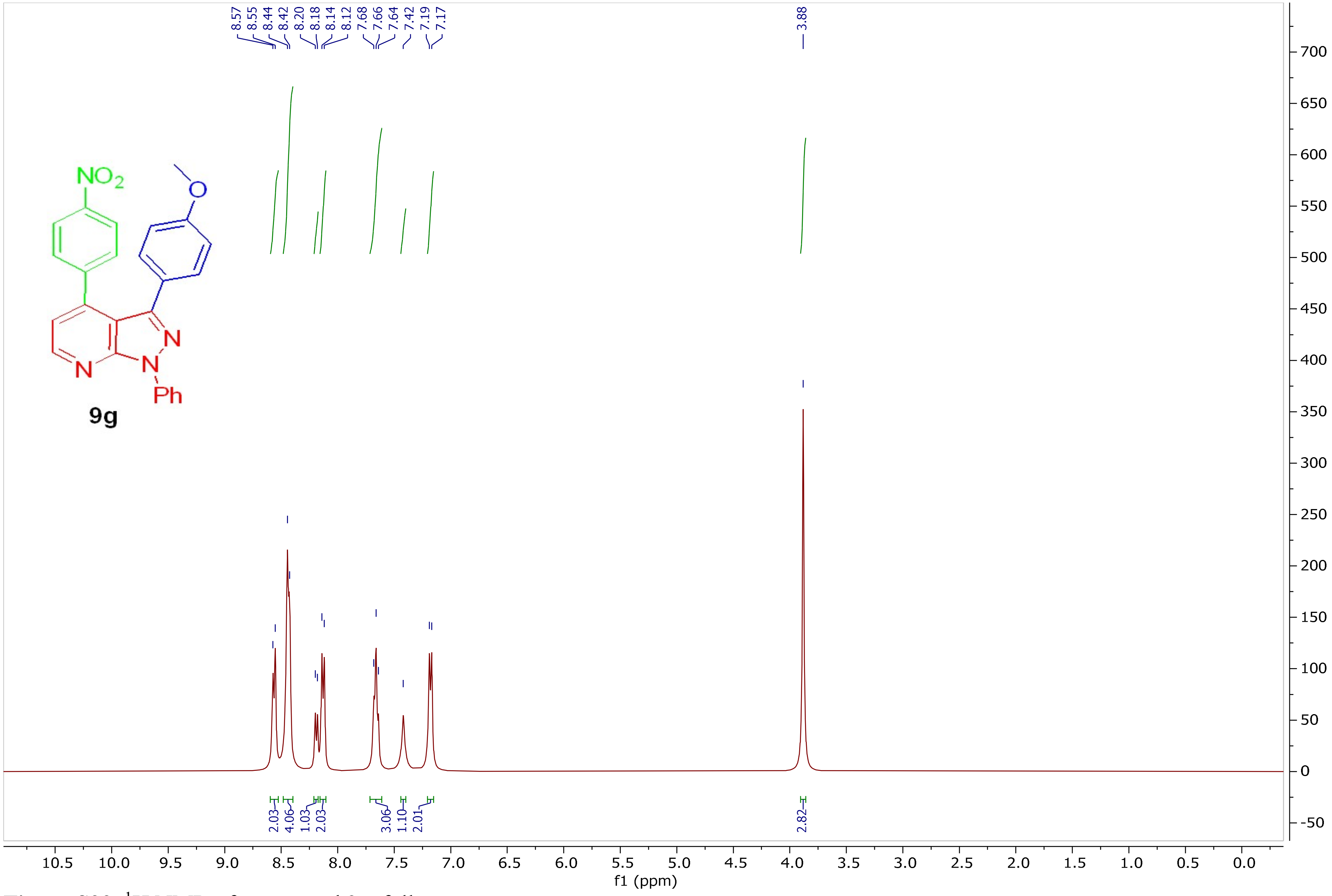

Figure S22. ^{1}H NMR of compound **9g**, full spectrum

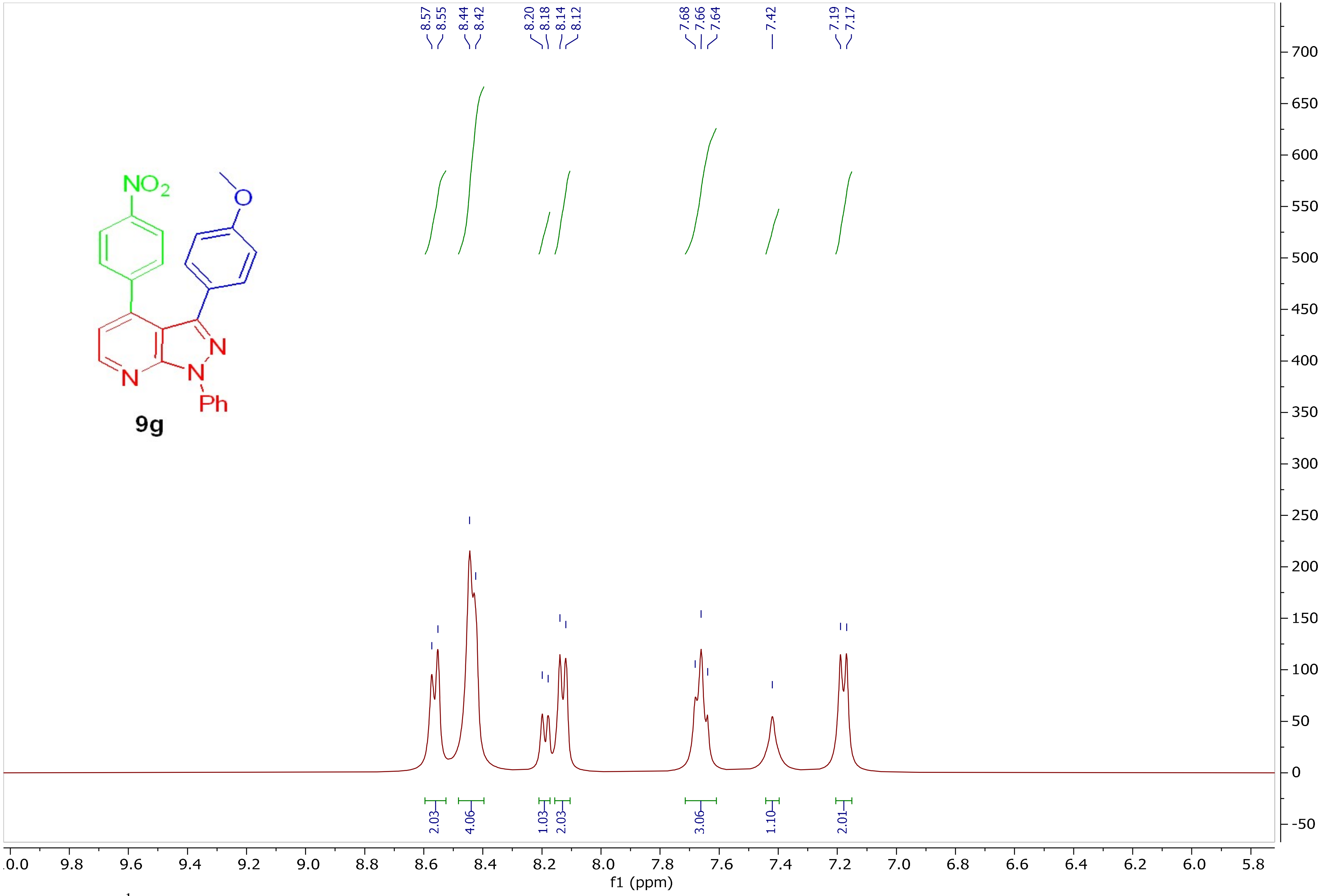

Figure S23. ^{1}H NMR of compound **9g**, aromatic region

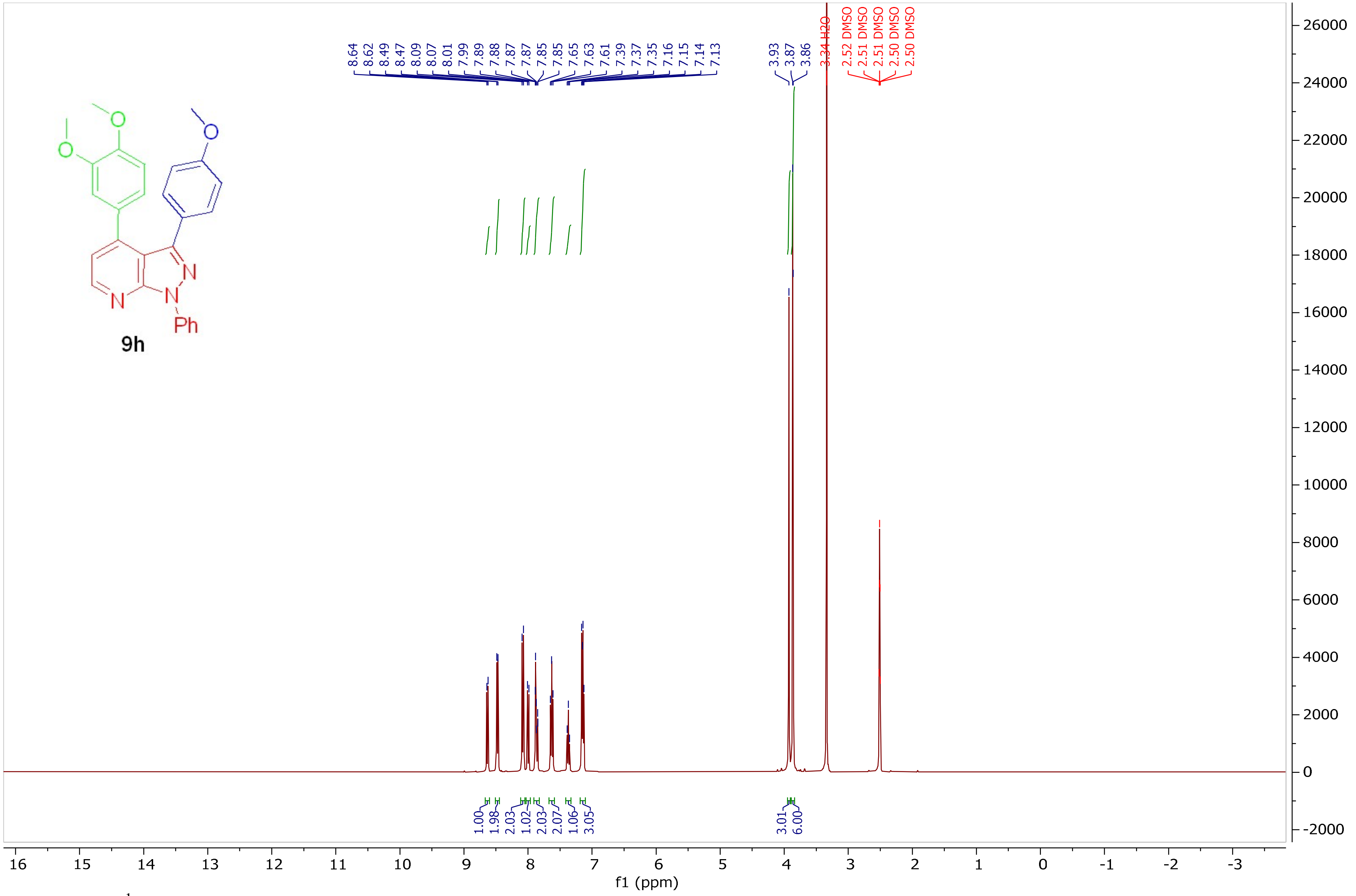

Figure S24. ^{1}H NMR of compound **9h**, full spectrum

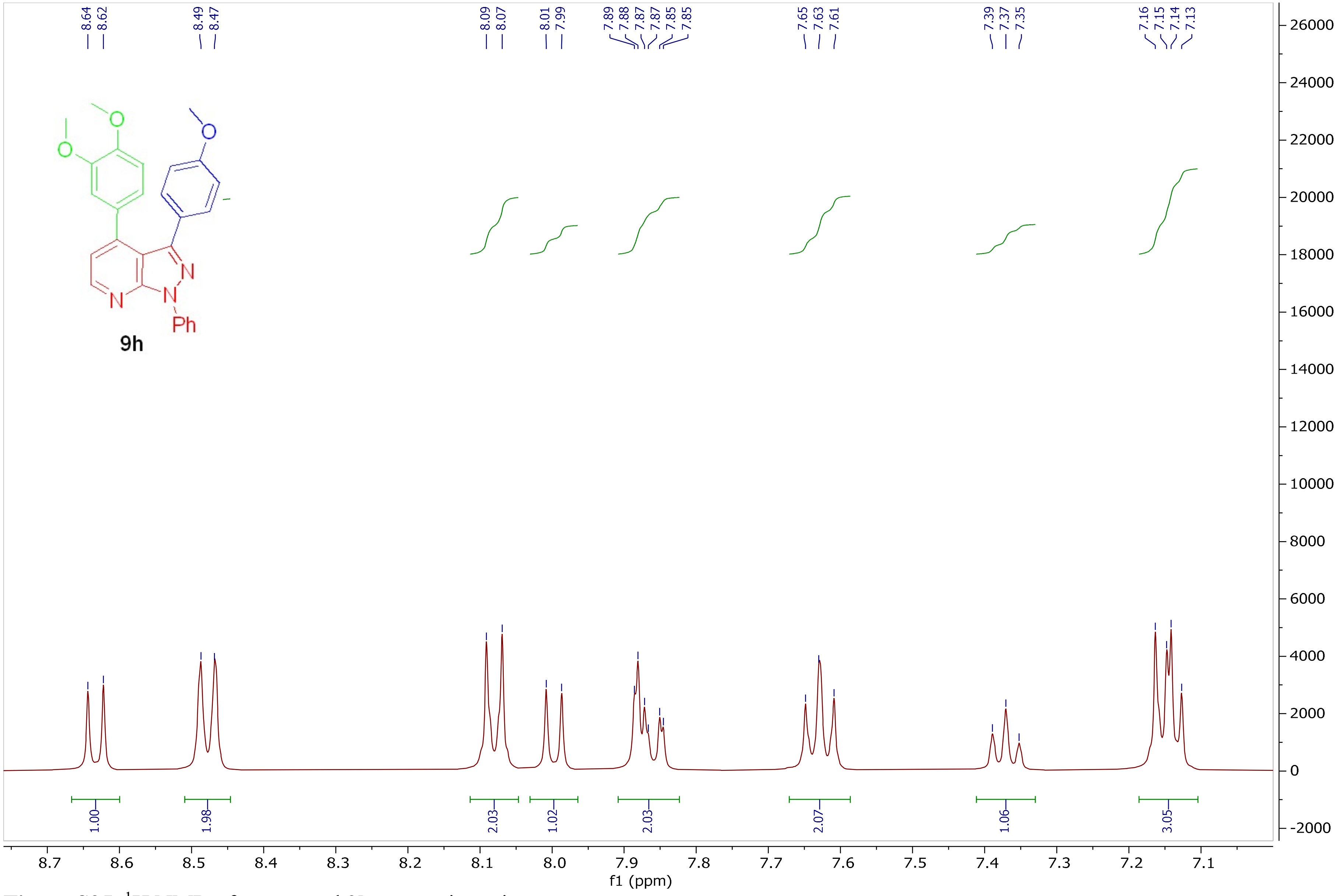

Figure S25. ^{1}H NMR of compound **9h**, aromatic region

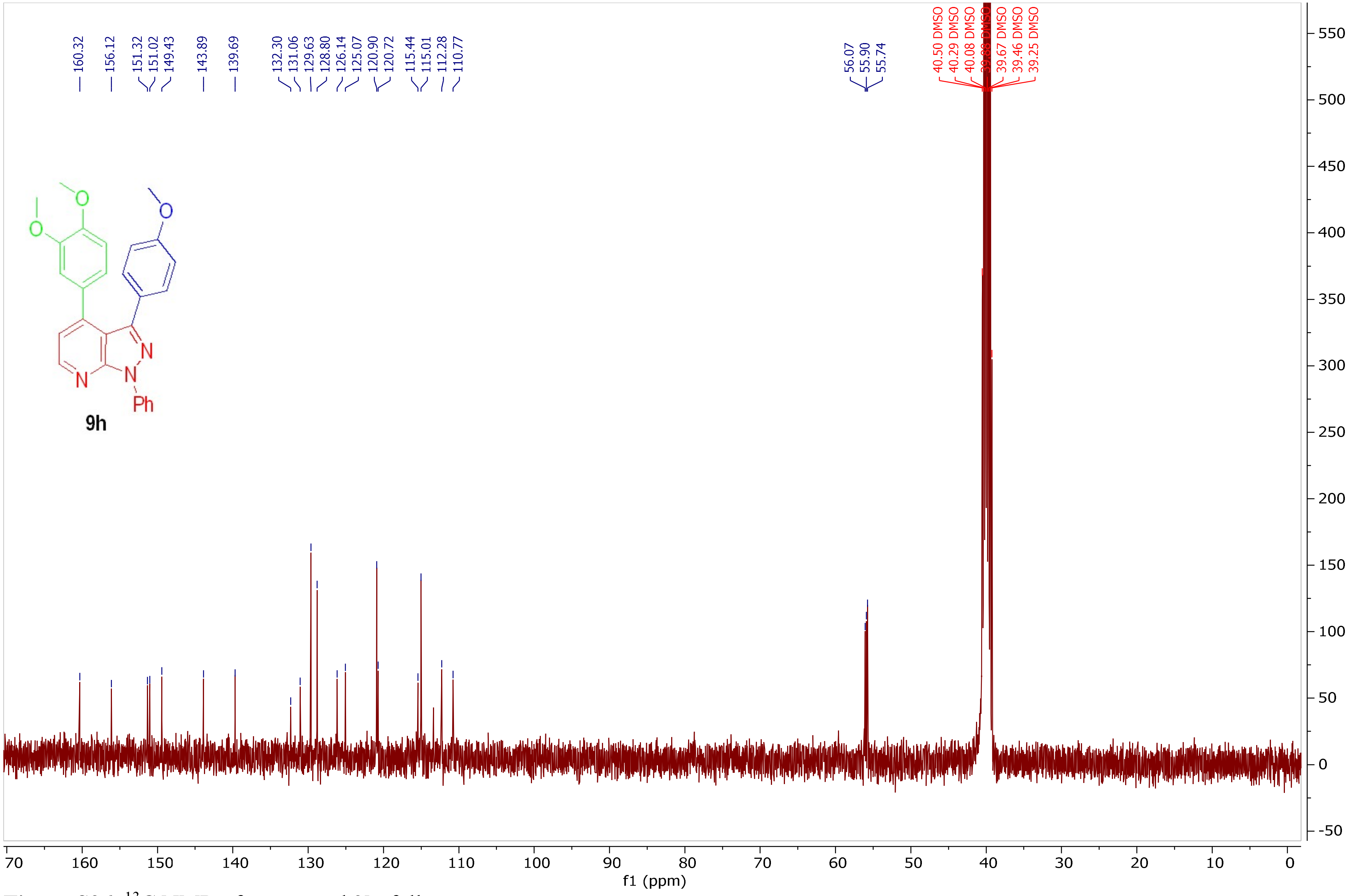

Figure S26. ^{13}C NMR of compound **9h**, full spectrum

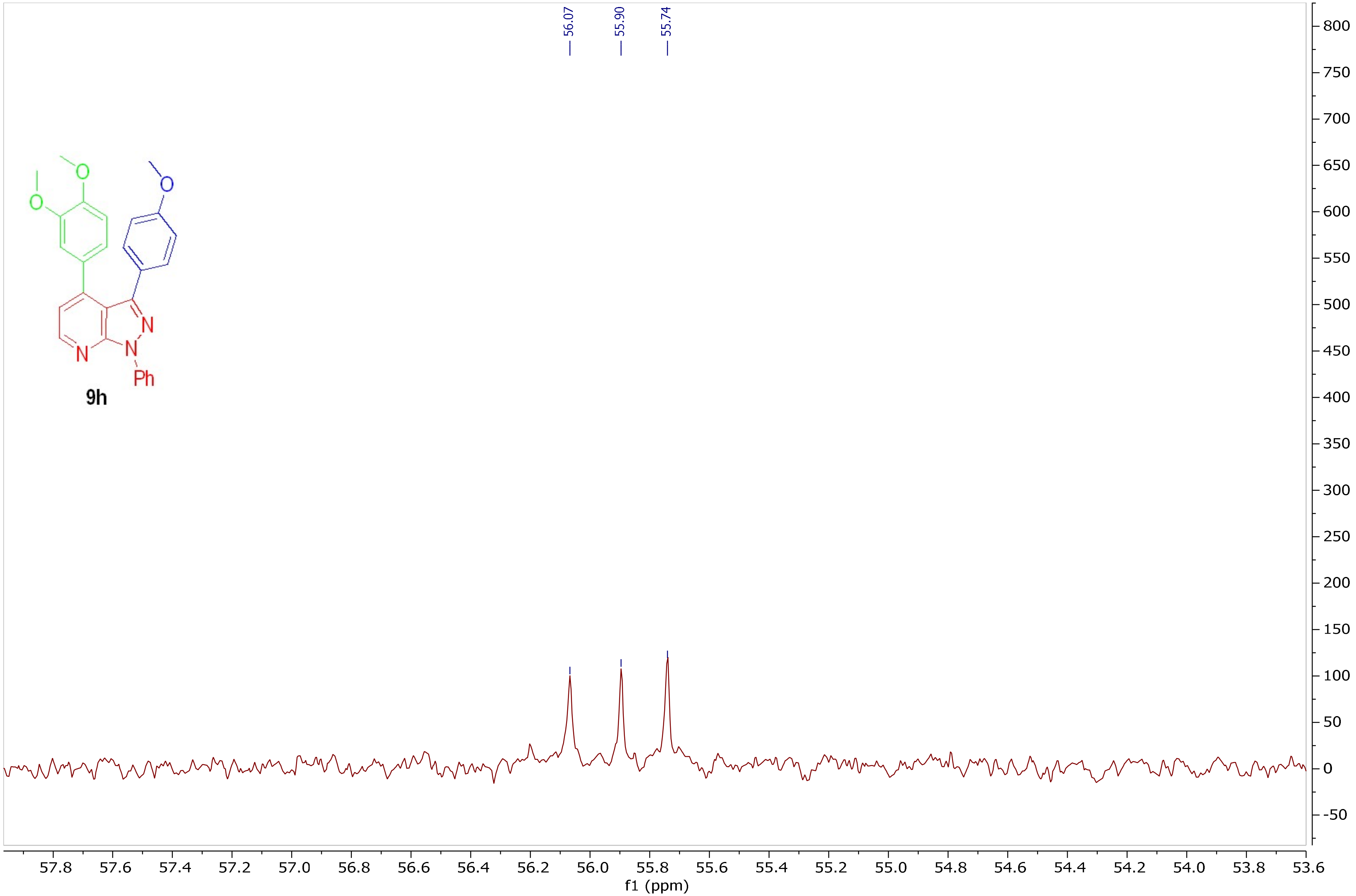

Figure S27. ^{13}C NMR of compound **9h**, aliphatic region

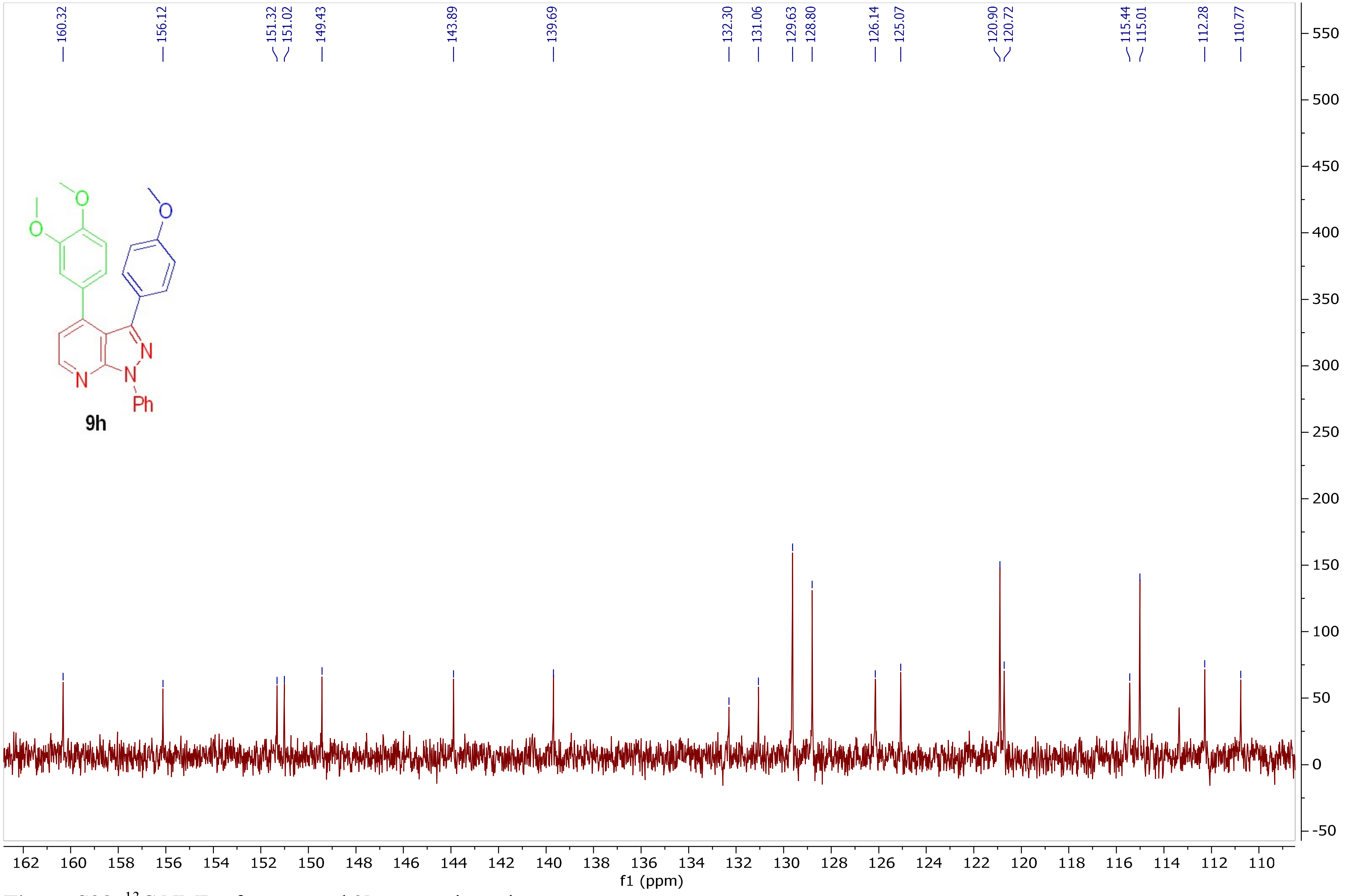

Figure S28. ^{13}C NMR of compound **9h**, aromatic region

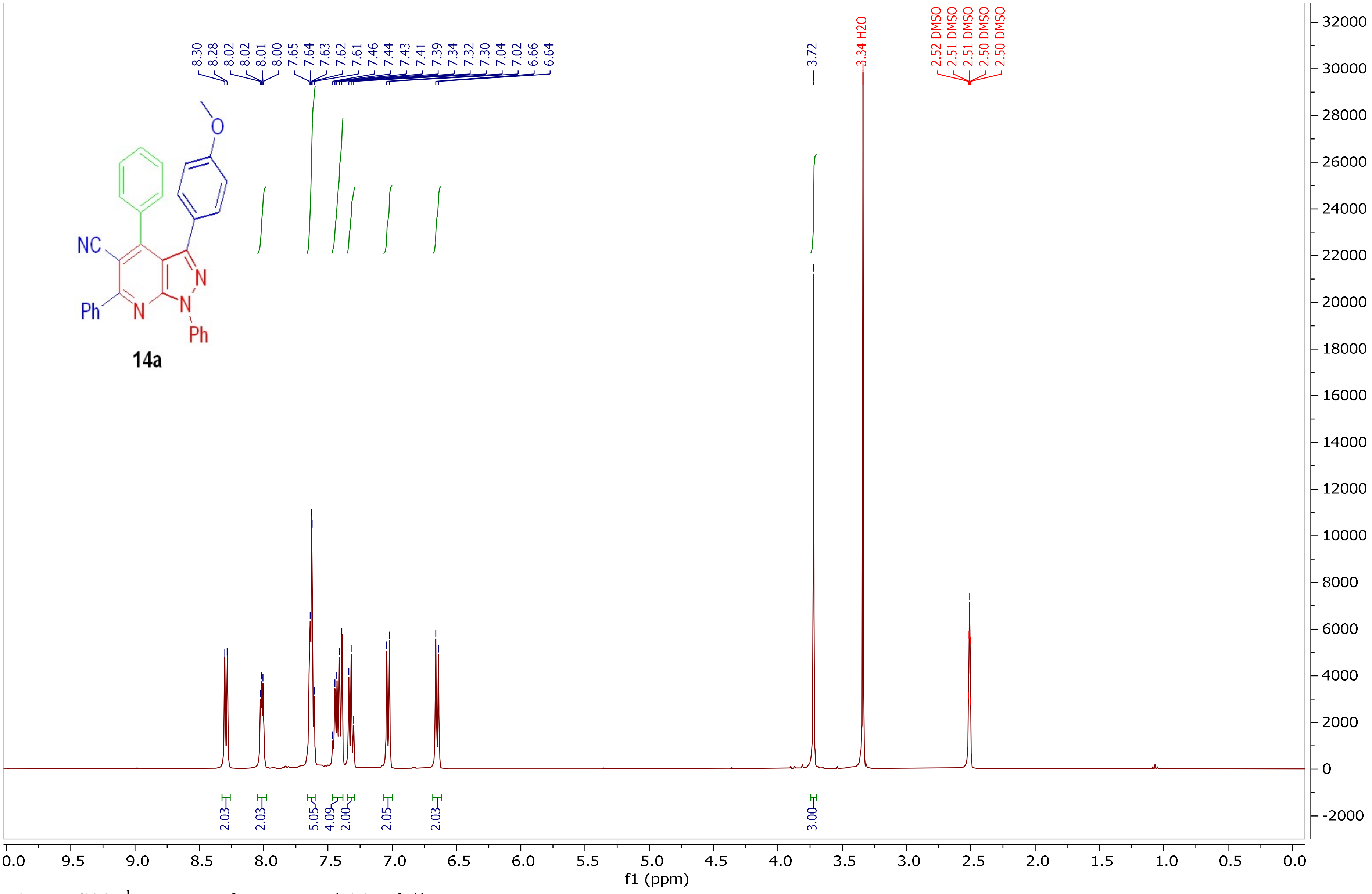

Figure S29. ^{1}H NMR of compound **14a**, full spectrum

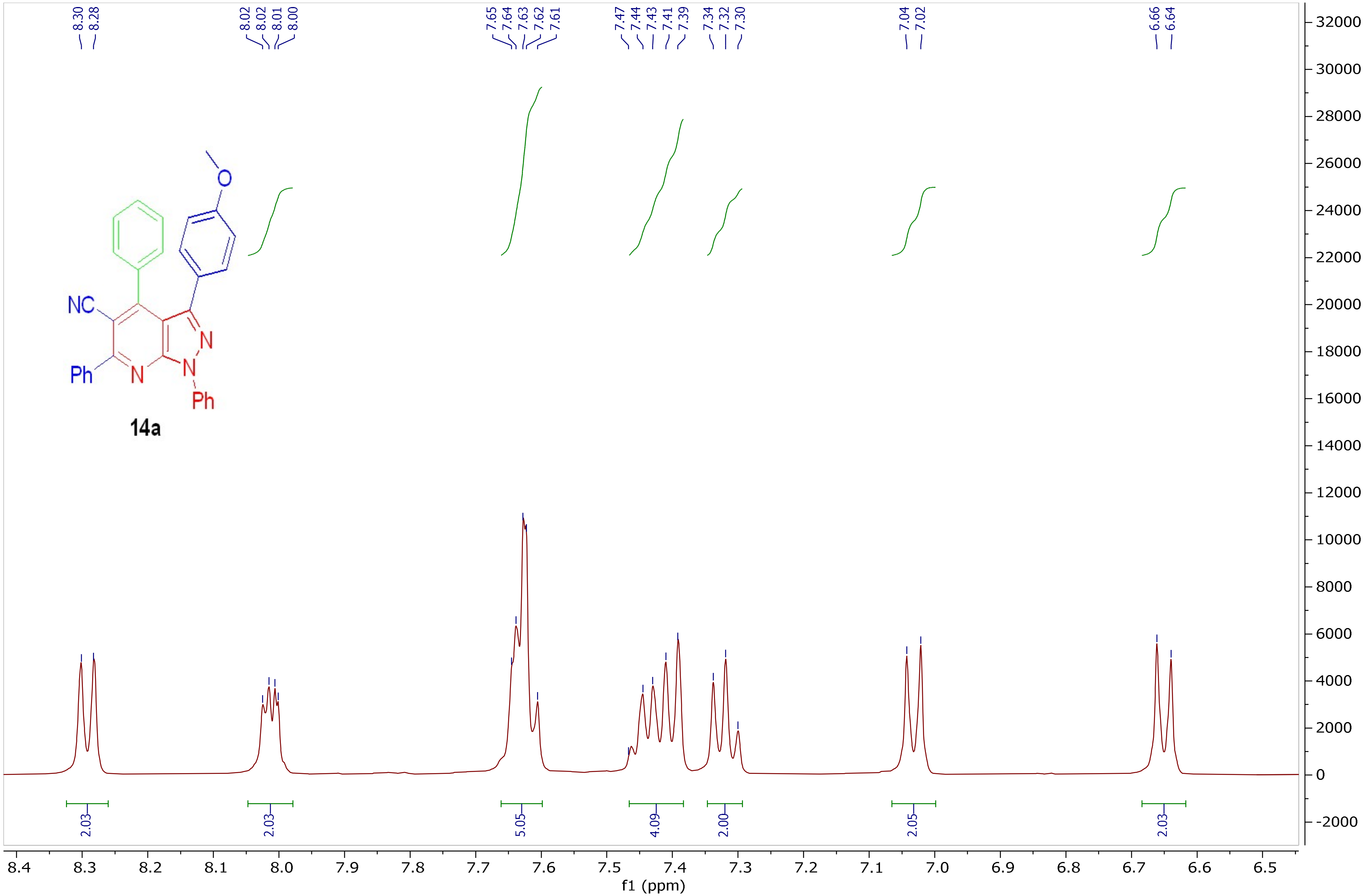

Figure S30. ^{1}H NMR of compound **14a**, aromatic region

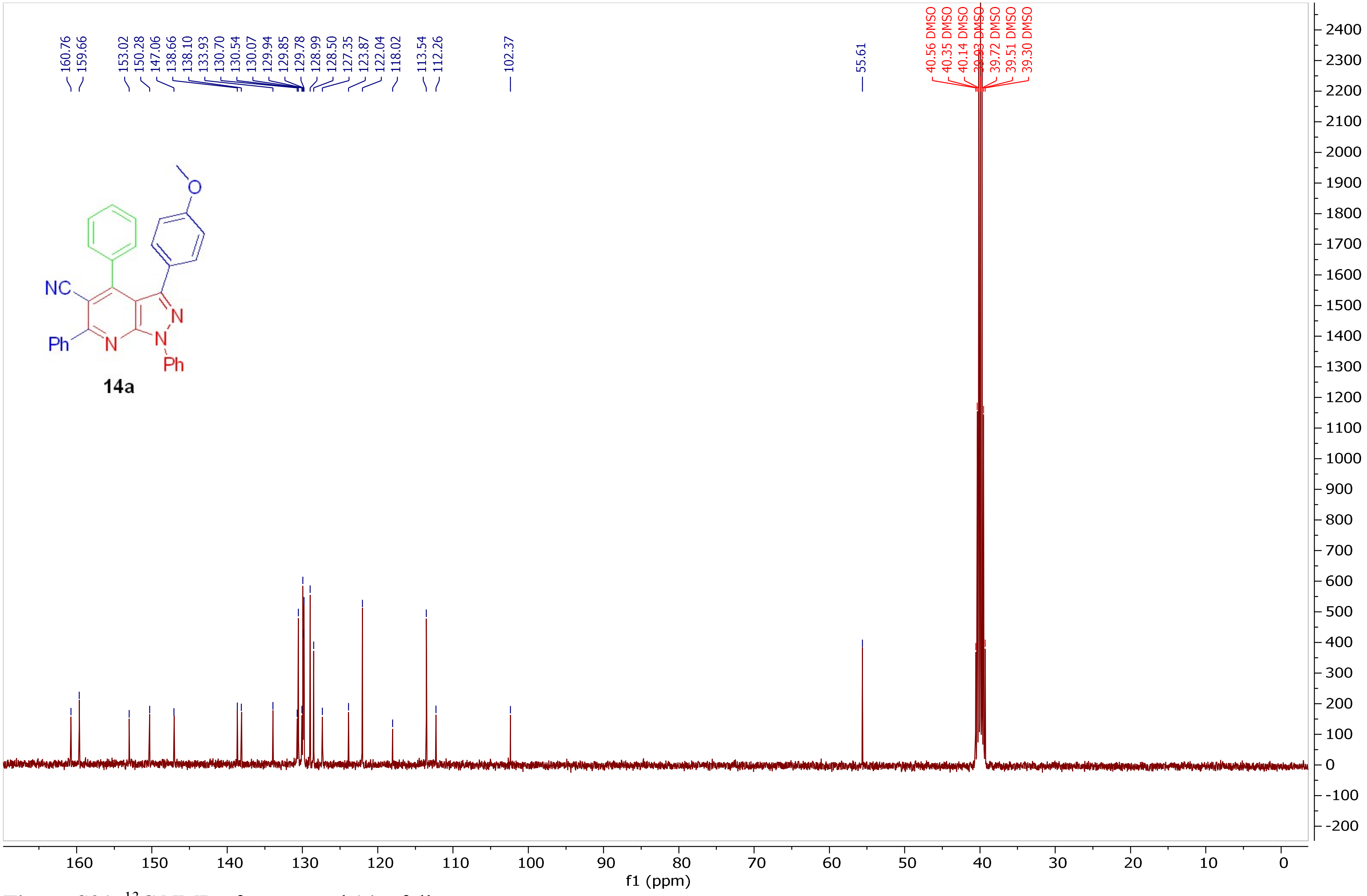

Figure S31. ^{13}C NMR of compound **14a**, full spectrum

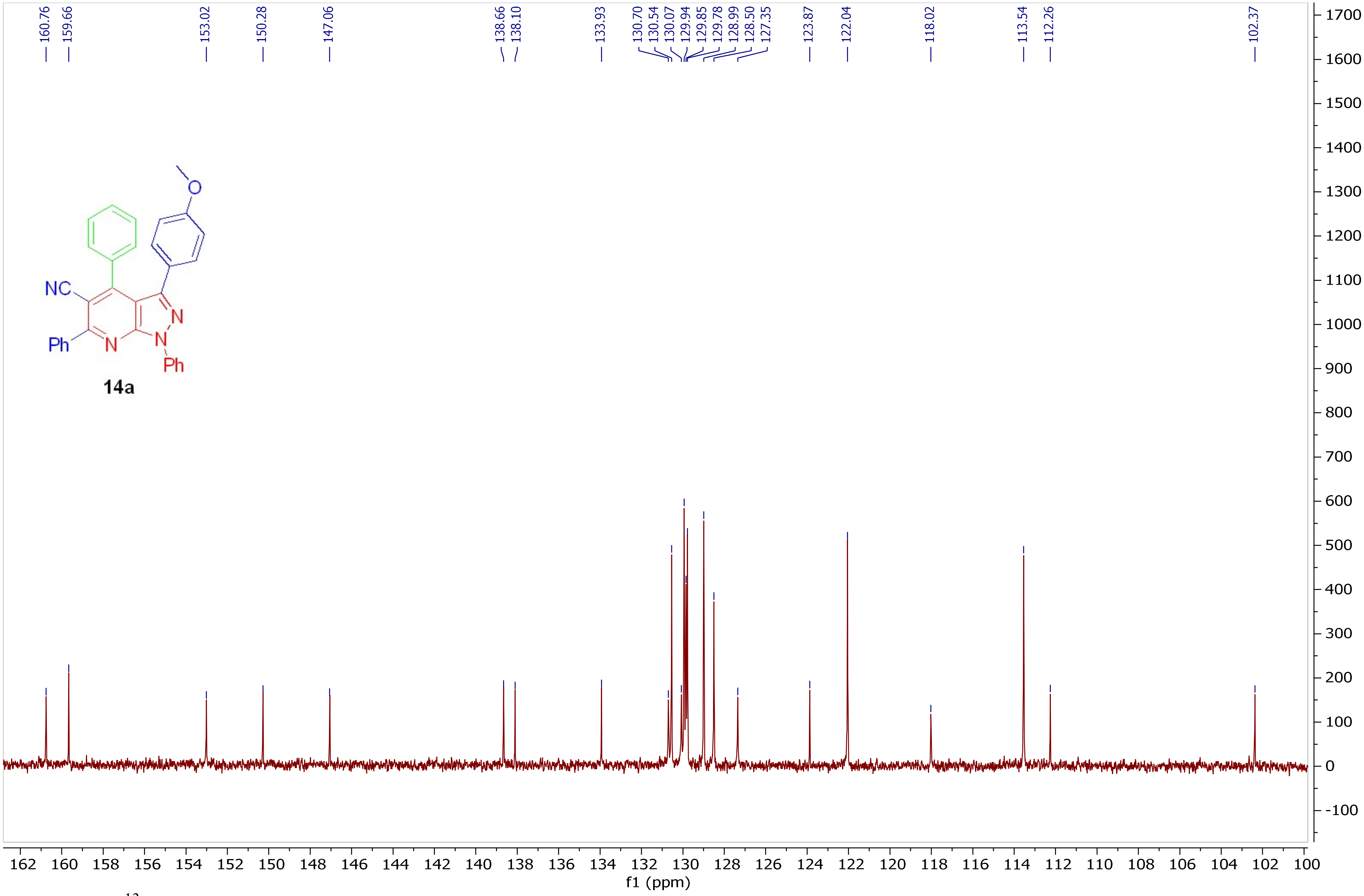

Figure S32. ^{13}C NMR of compound **14a**, aromatic region

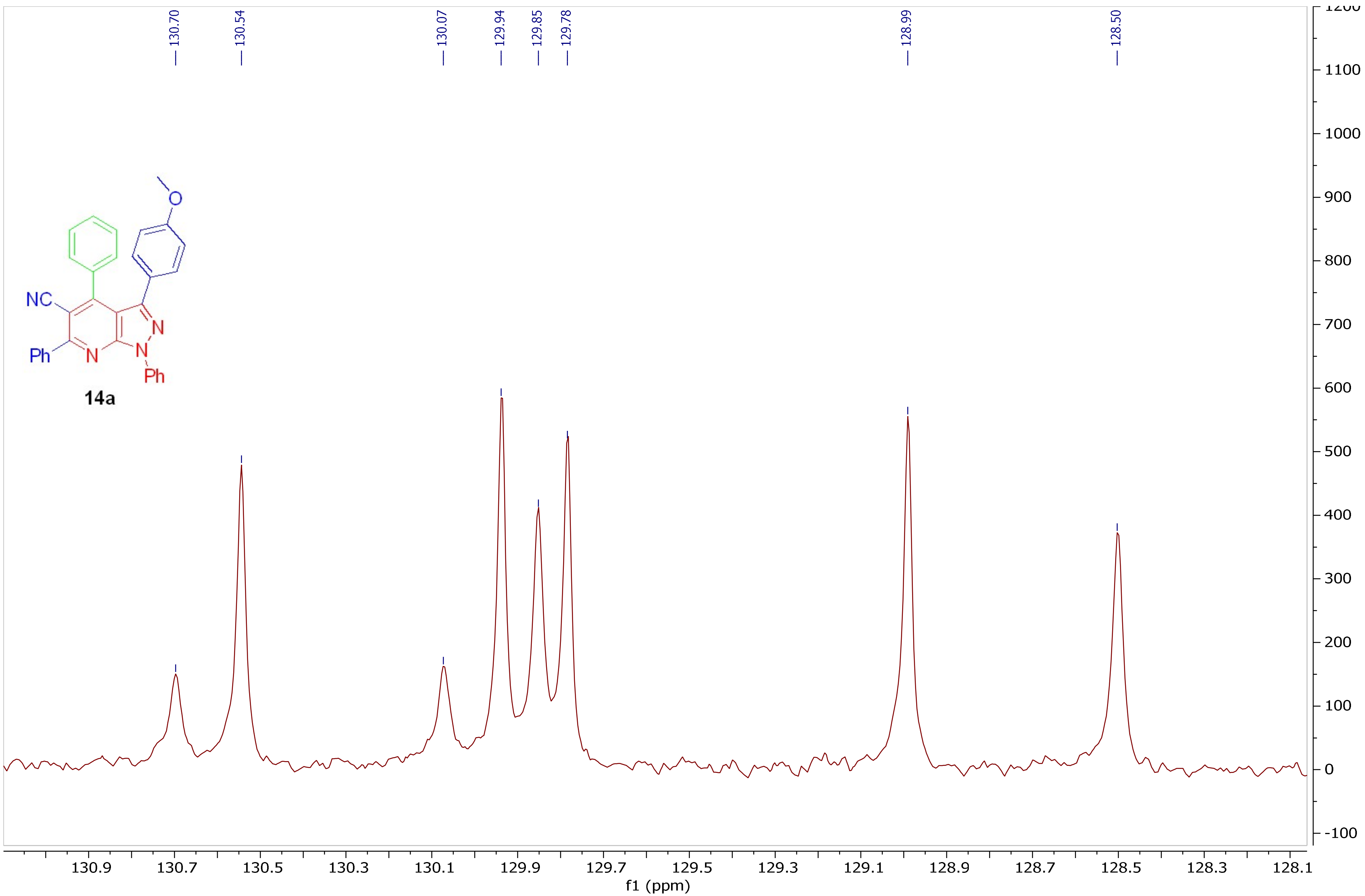

Figure S33. ^{13}C NMR of compound **14a**, aromatic region 128-131 *ppm*

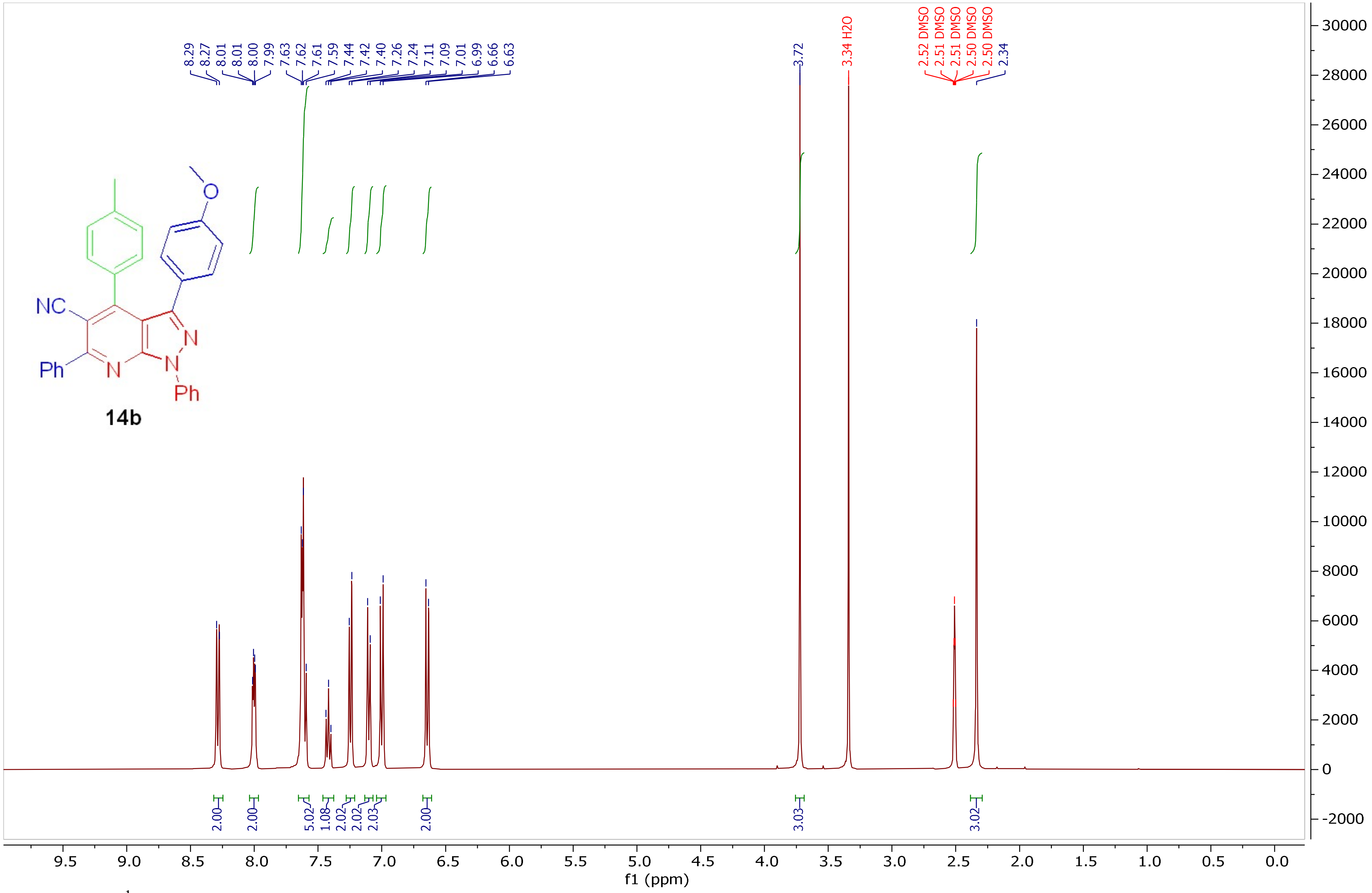

Figure S34. ^{1}H NMR of compound **14b**, full spectrum

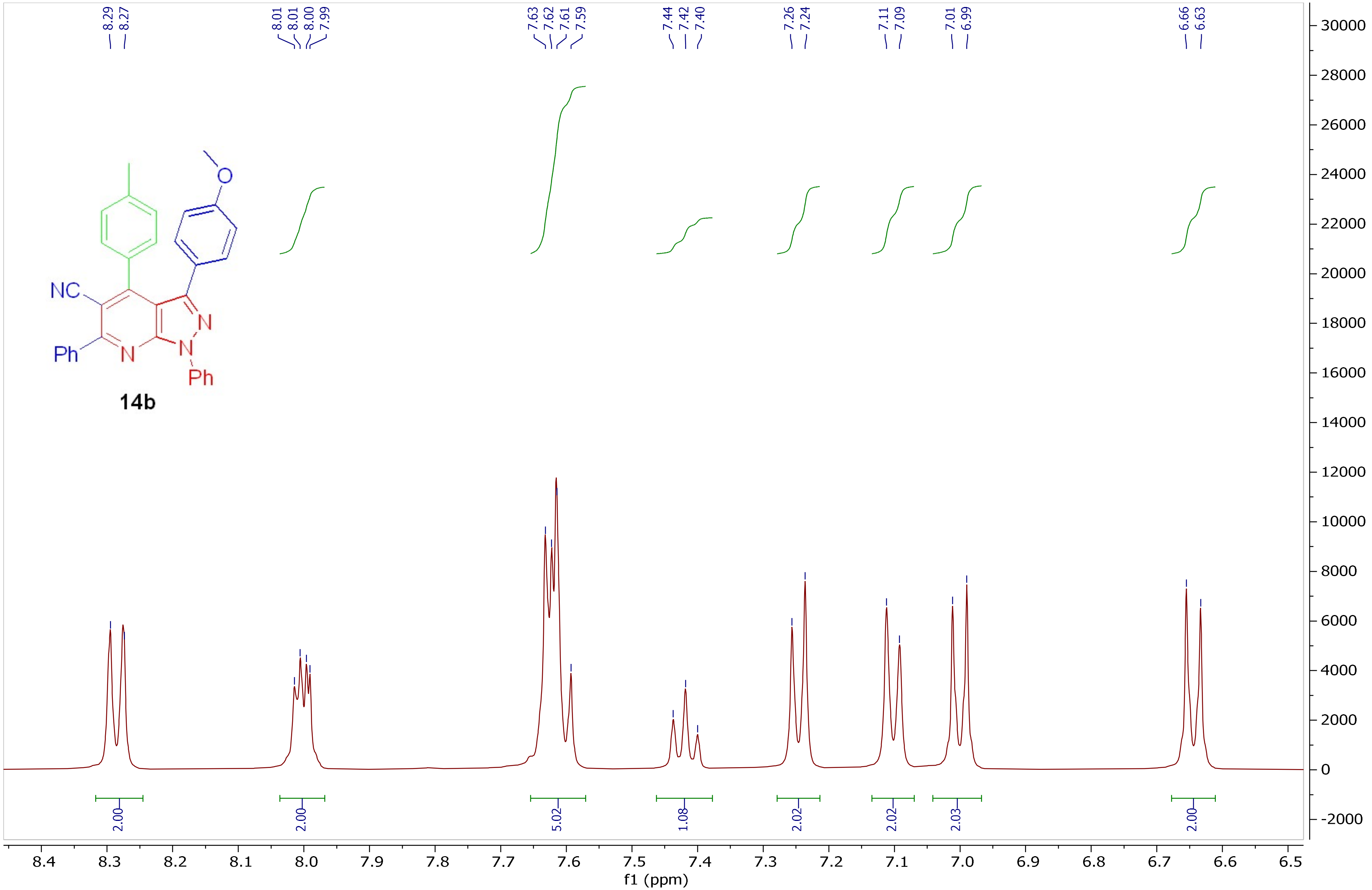

Figure S35. ^{1}H NMR of compound **14b**, aromatic region

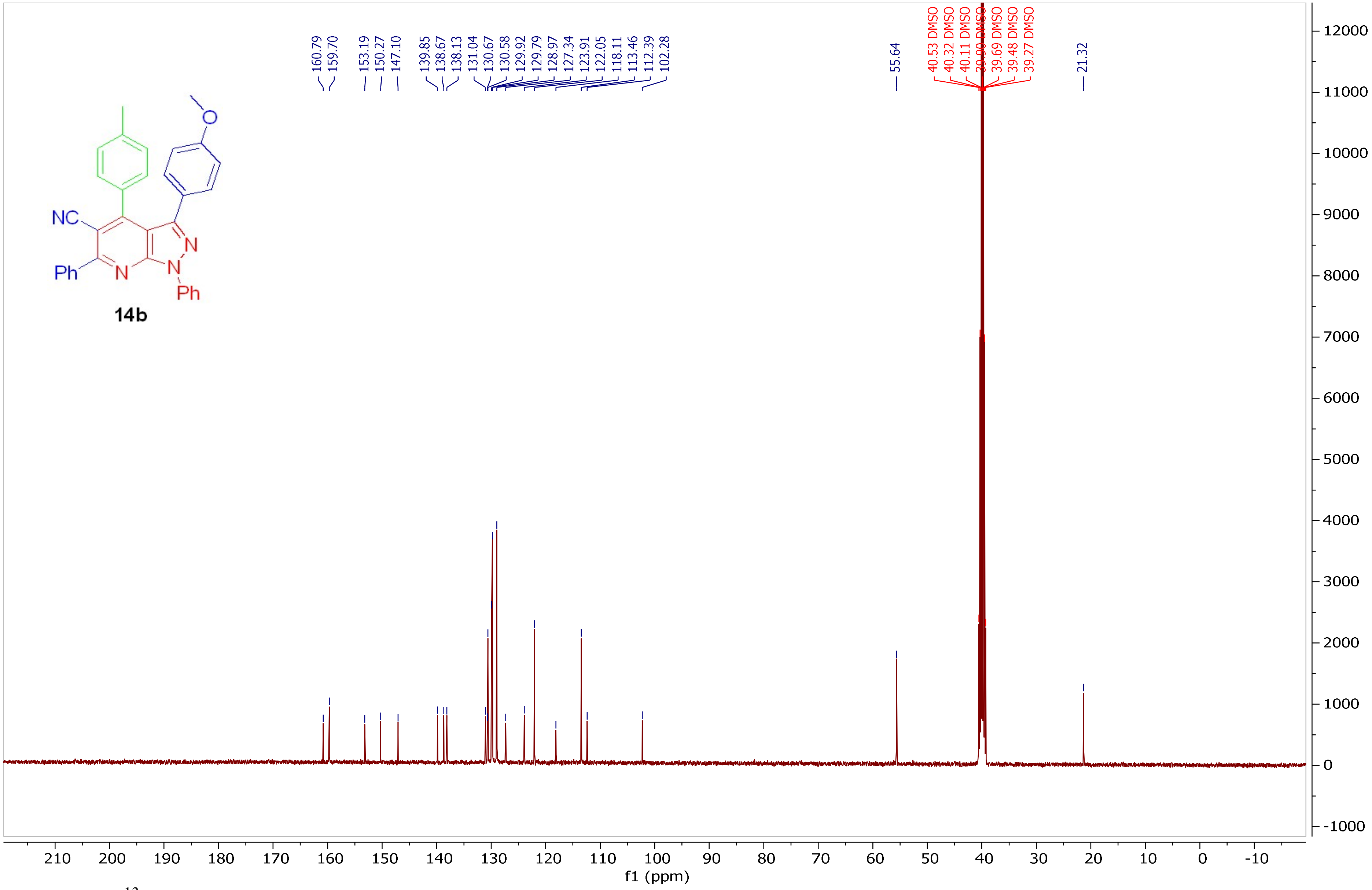

Figure S36. ^{13}C NMR of compound **14b**, full spectrum

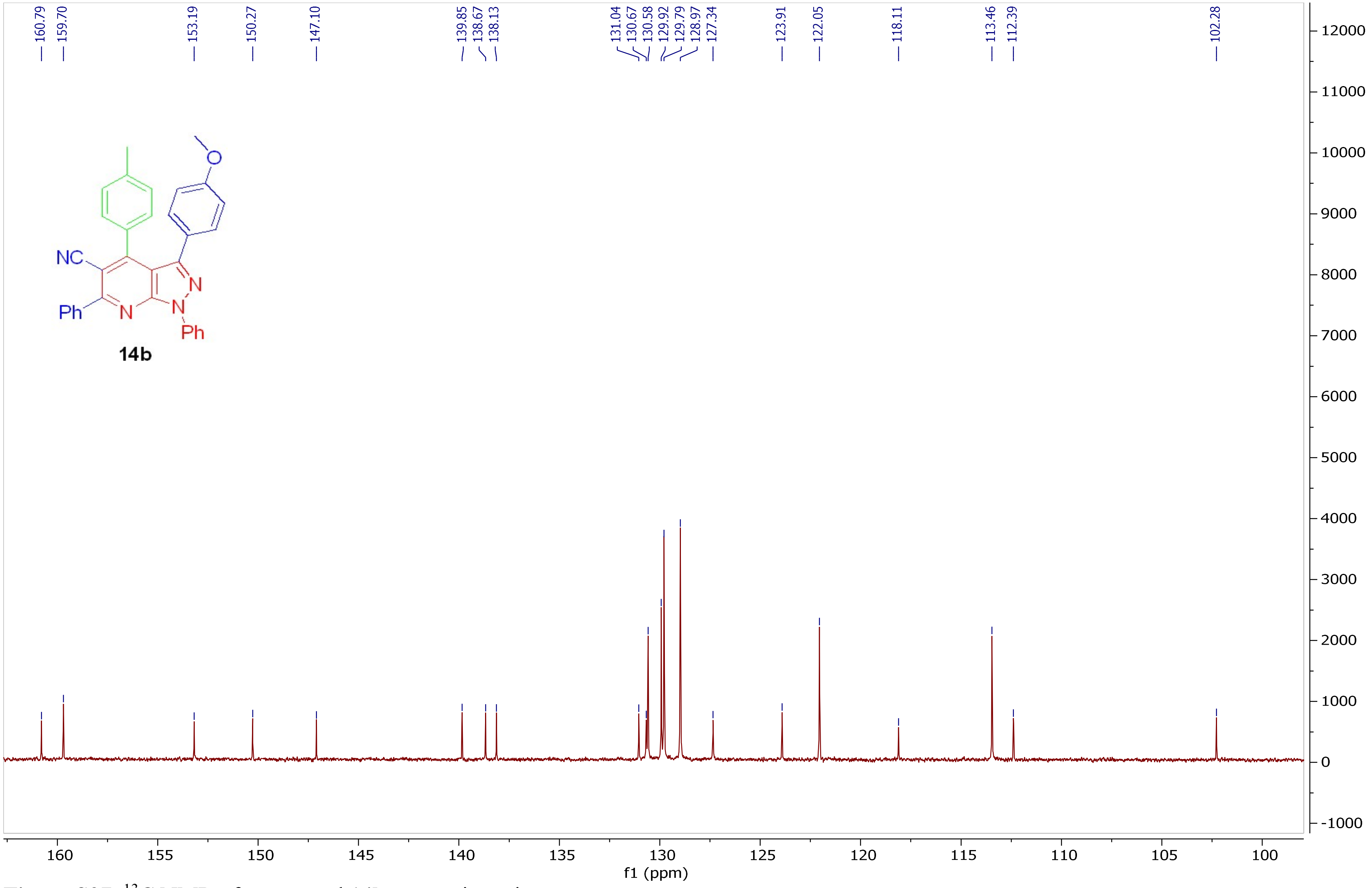

Figure S37. ^{13}C NMR of compound **14b**, aromatic region

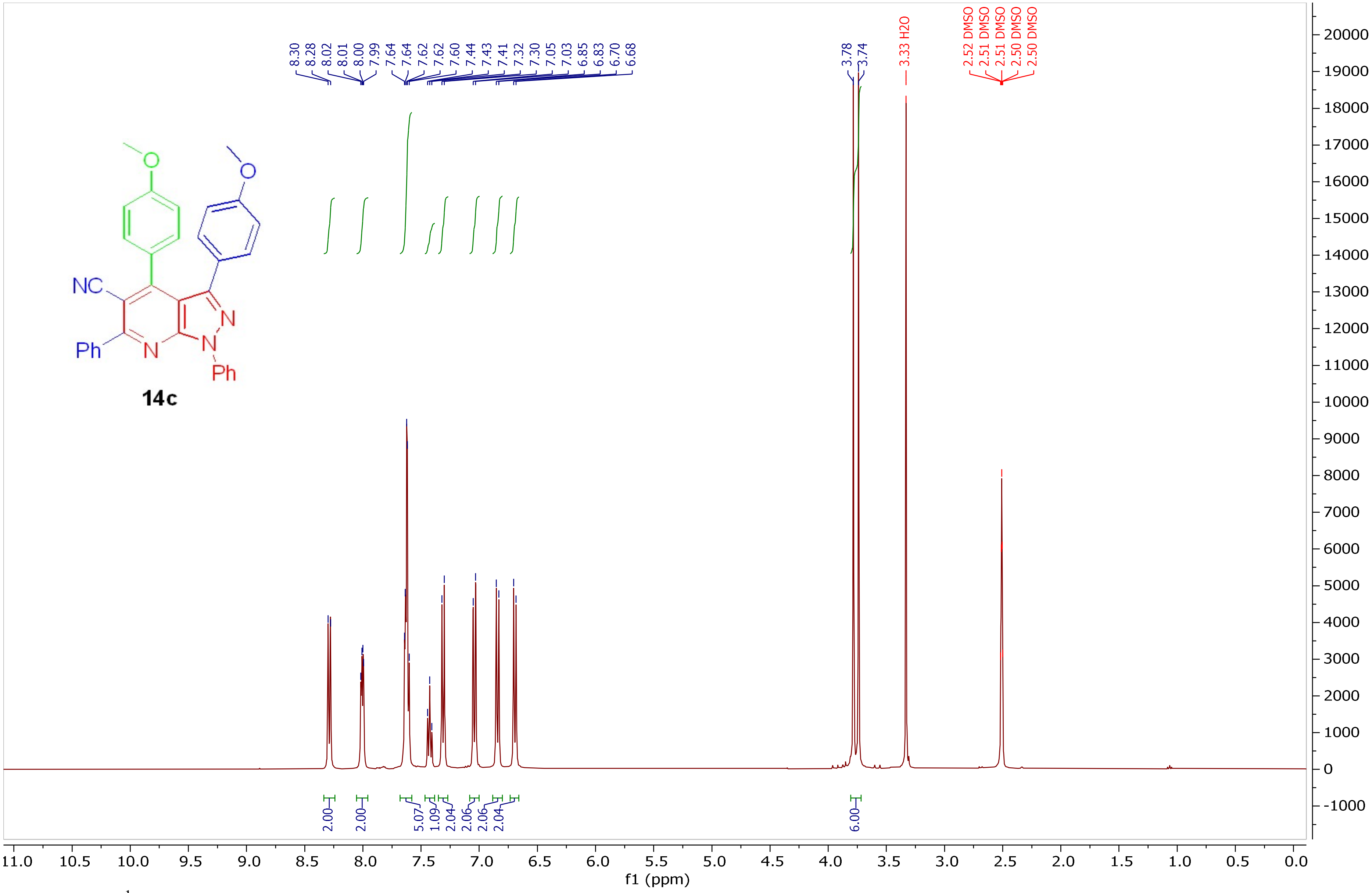

Figure S38. ¹H NMR of compound **14c**, full spectrum

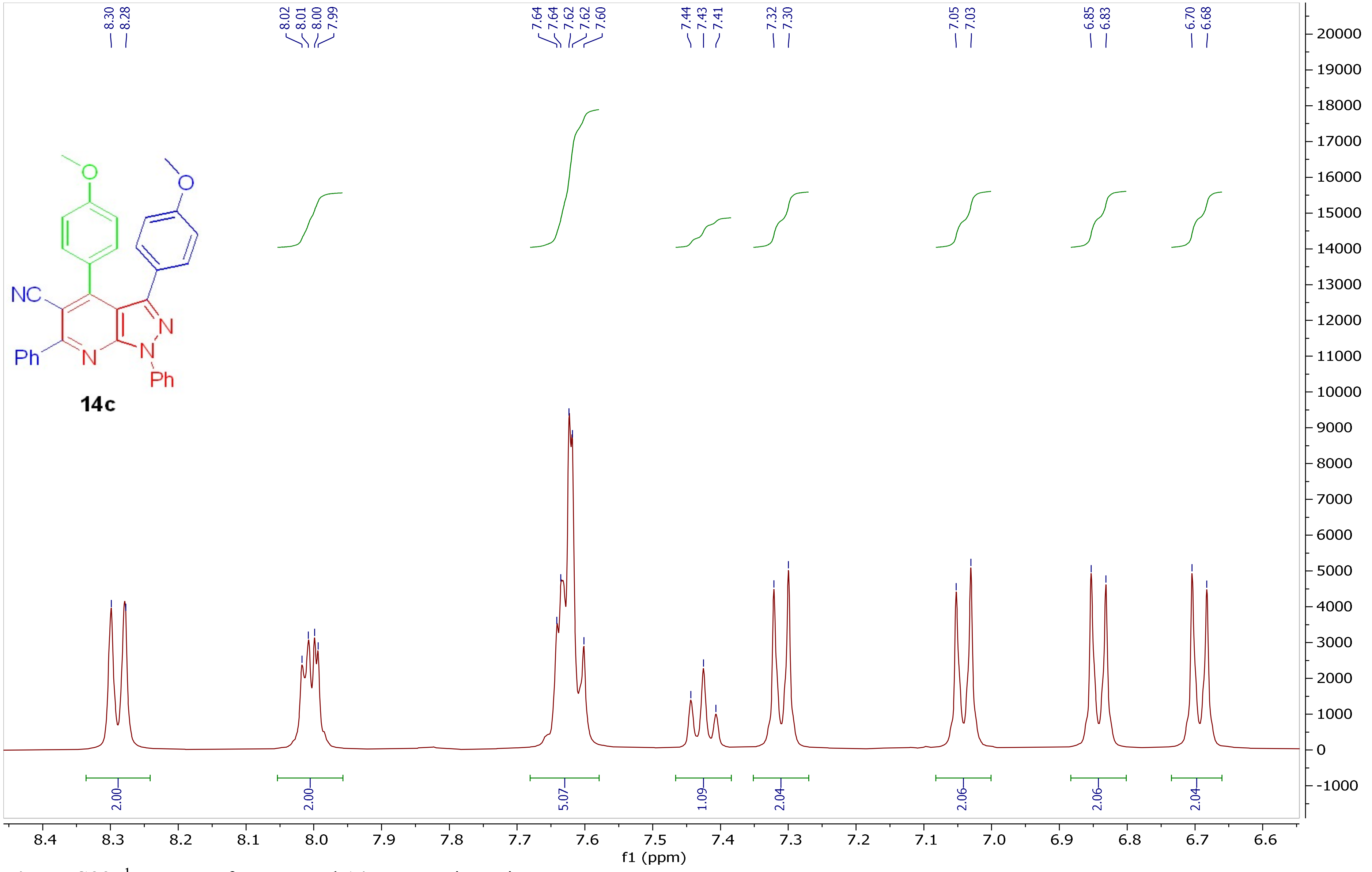

Figure S39. ^{1}H NMR of compound **14c**, aromatic region

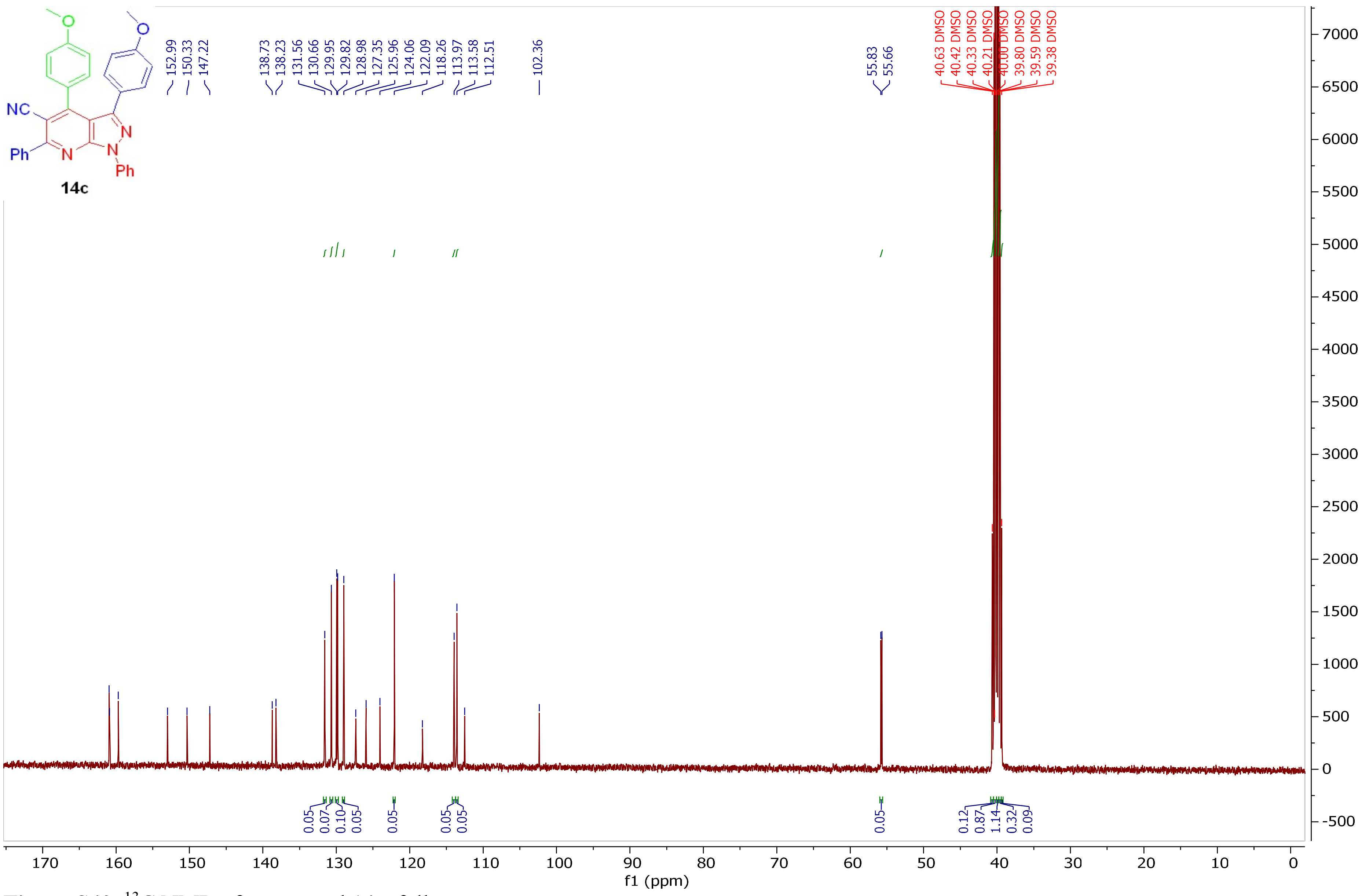

Figure S40. ^{13}C NMR of compound **14c**, full spectrum

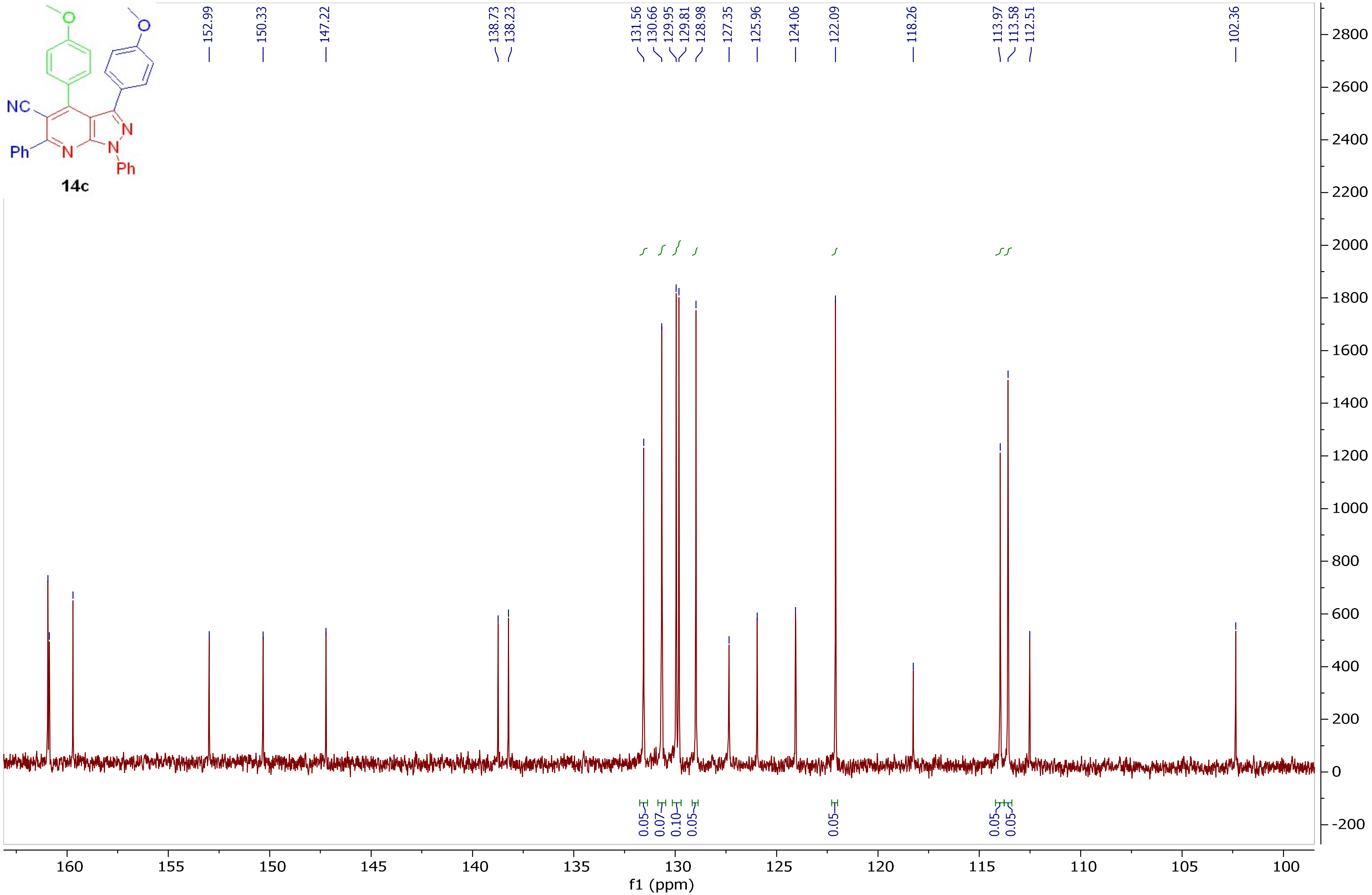

Figure S41. ^{13}C NMR of compound **14c**, aromatic region

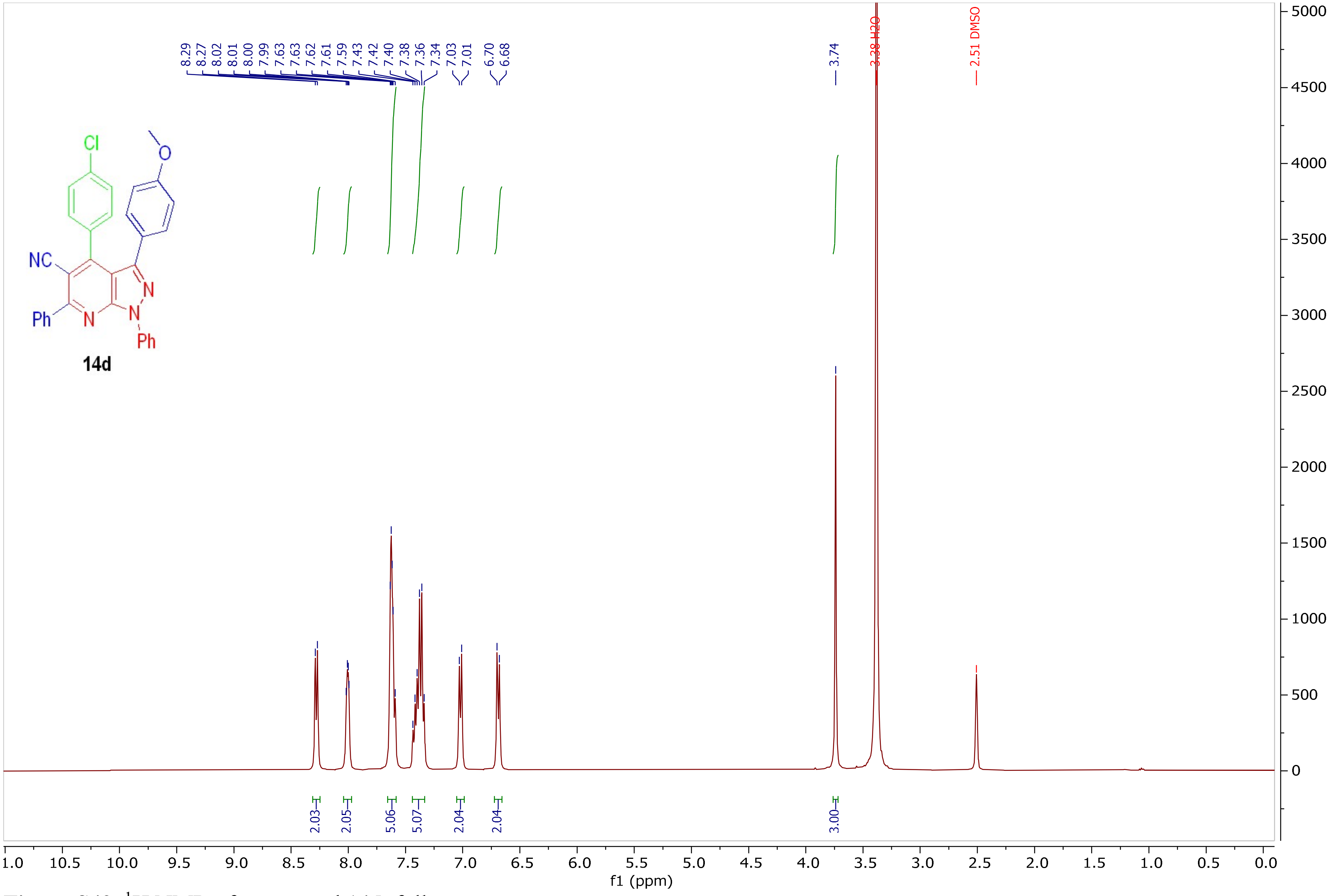

Figure S42. ^{1}H NMR of compound **14d**, full spectrum

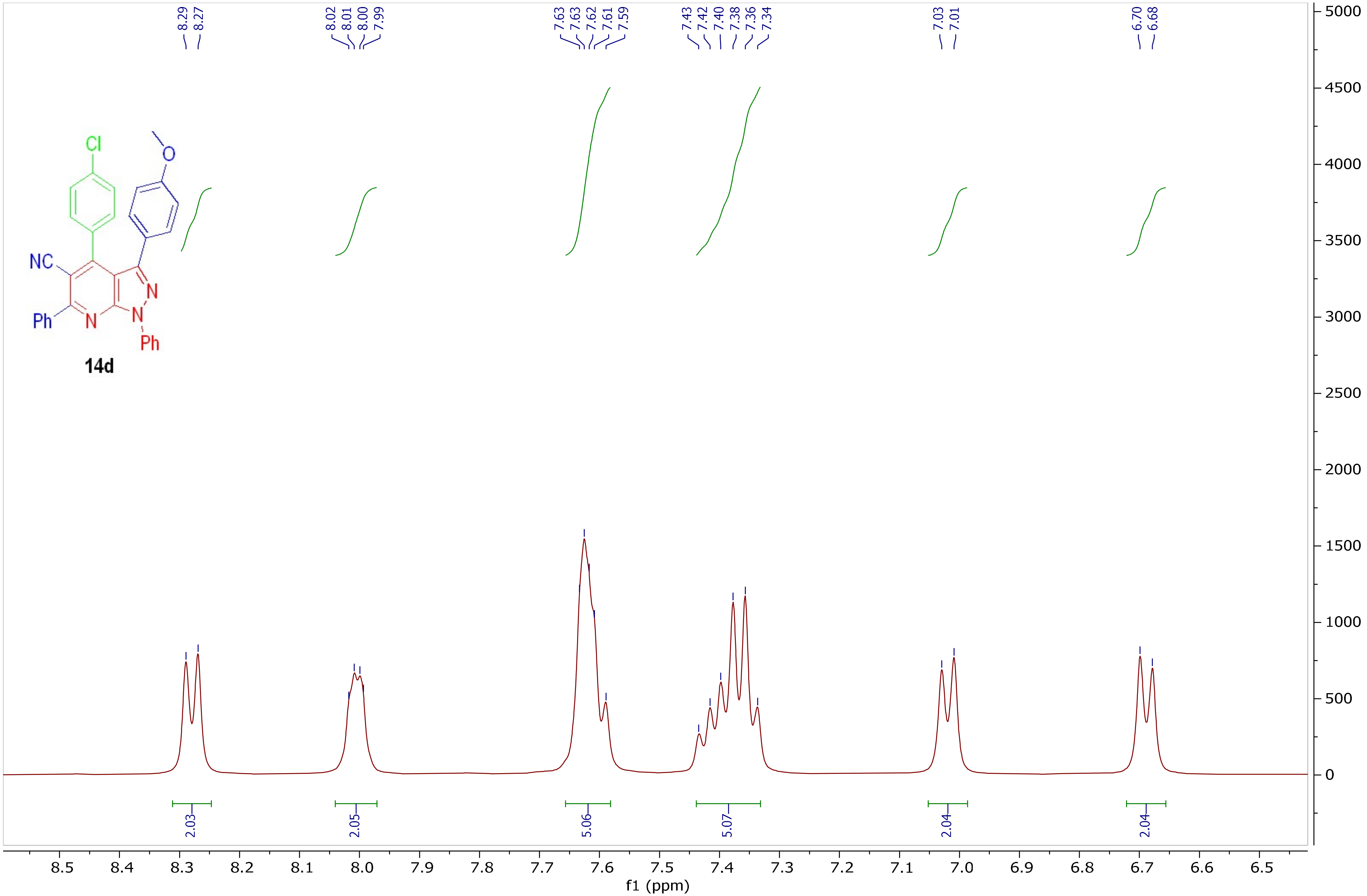

Figure S43. ^{1}H NMR of compound **14d**, aromatic region

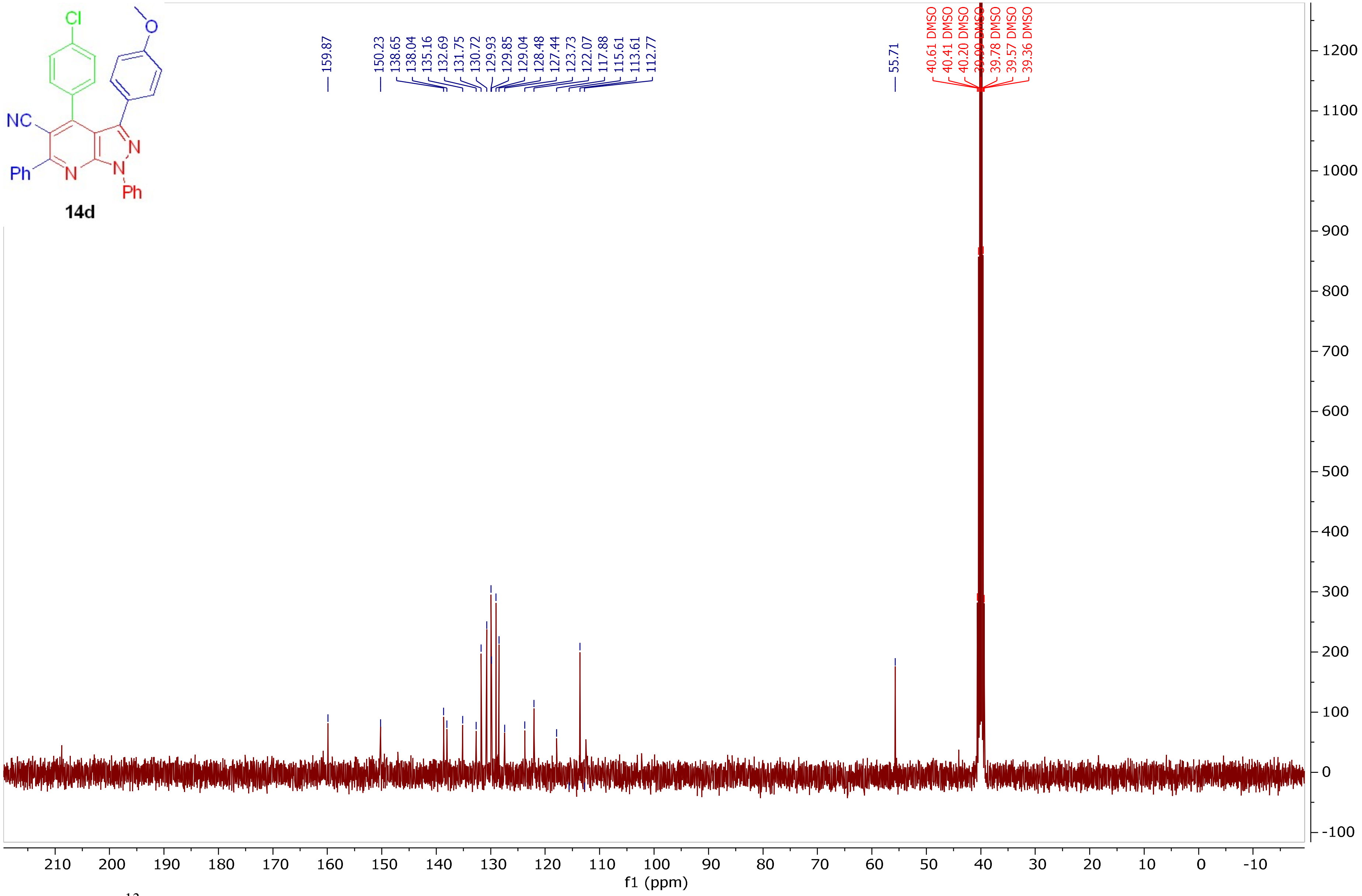

Figure S44. ^{13}C NMR of compound **14d**, full spectrum

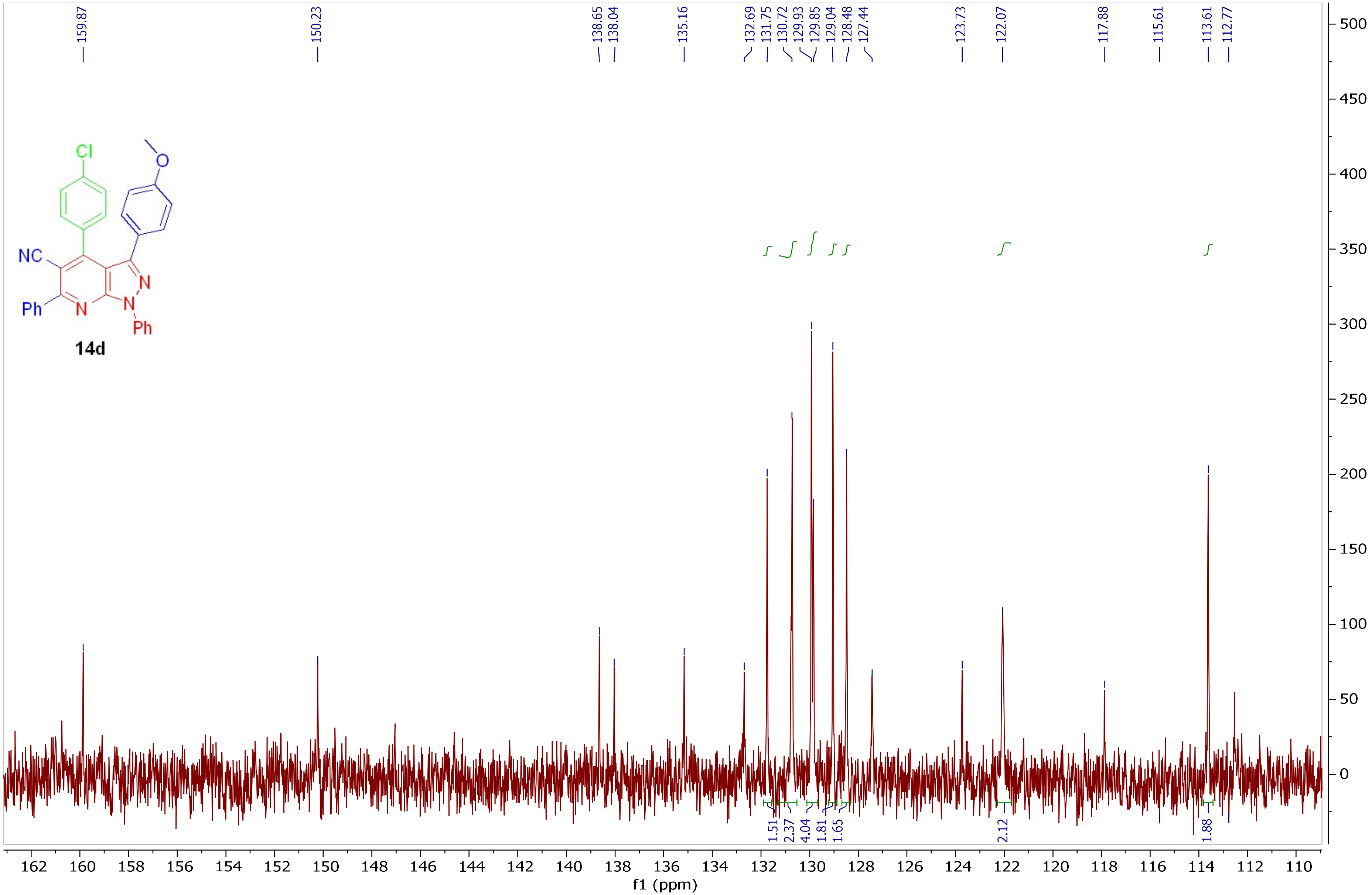

Figure S45. ^{13}C NMR of compound **14d**, aromatic region

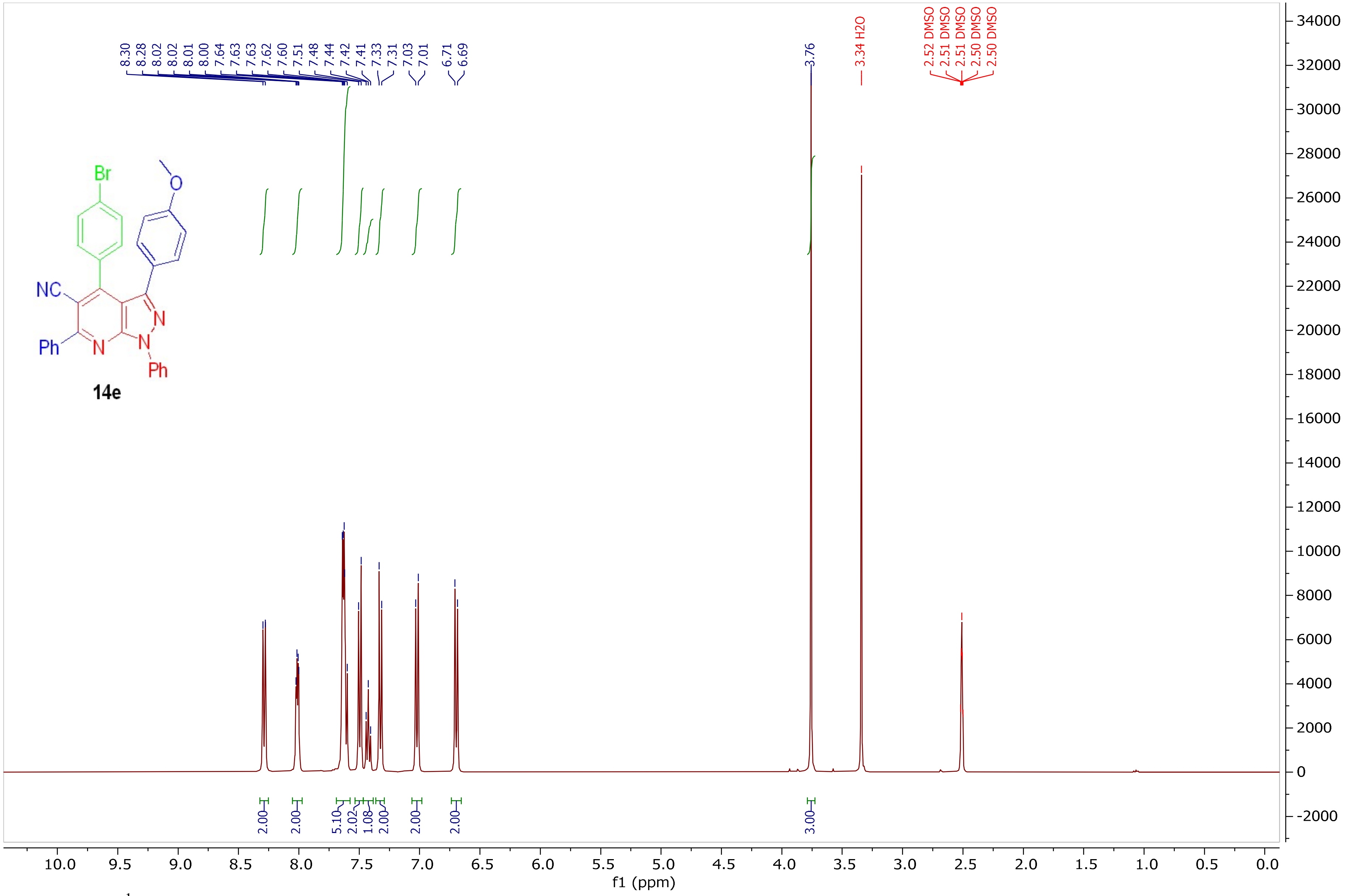

Figure S46. ^{1}H NMR of compound **14e**, full spectrum

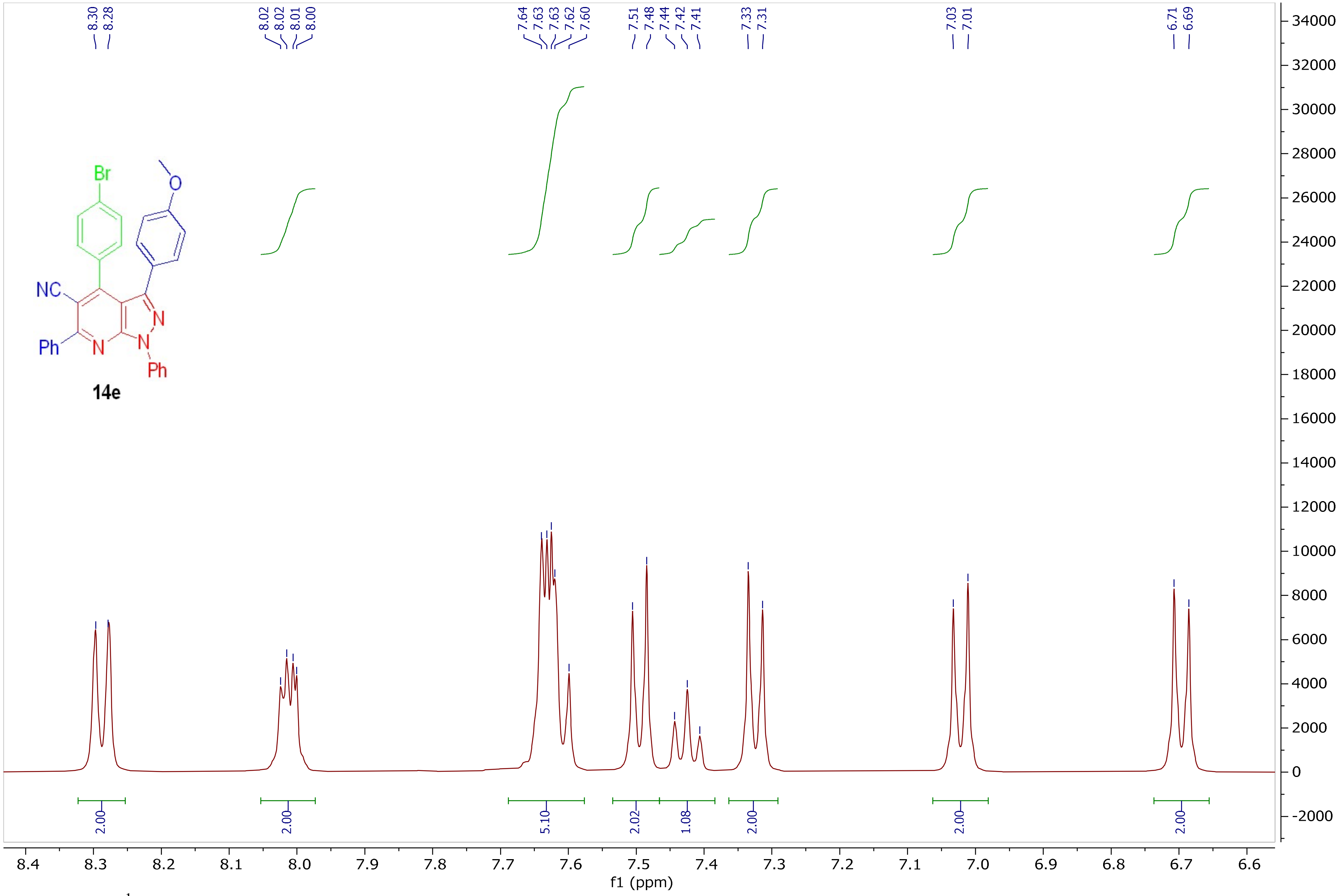

Figure S47. ^{1}H NMR of compound **14e**, aromatic region

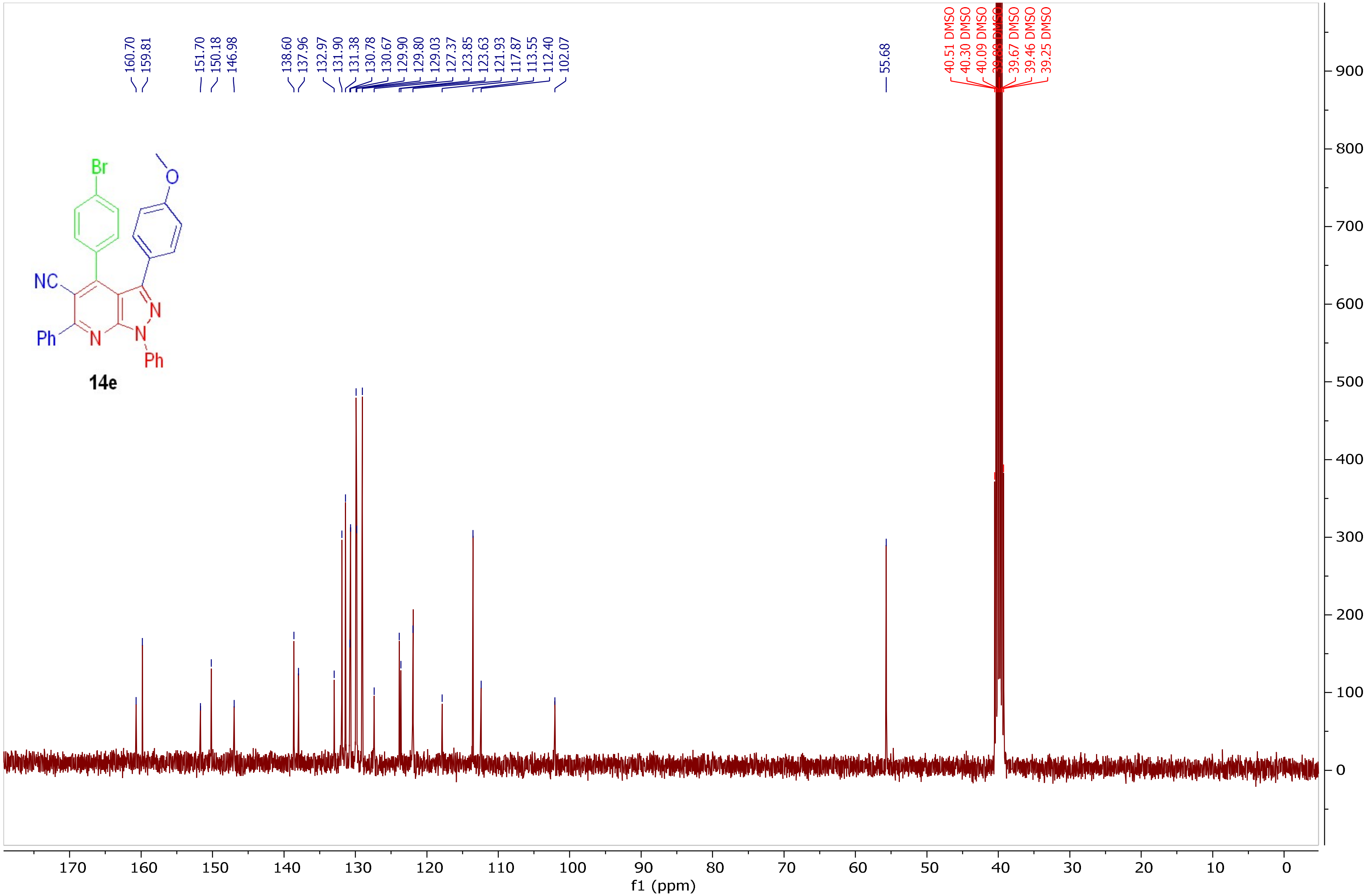

Figure S48. ^{13}C NMR of compound **14e**, full spectrum

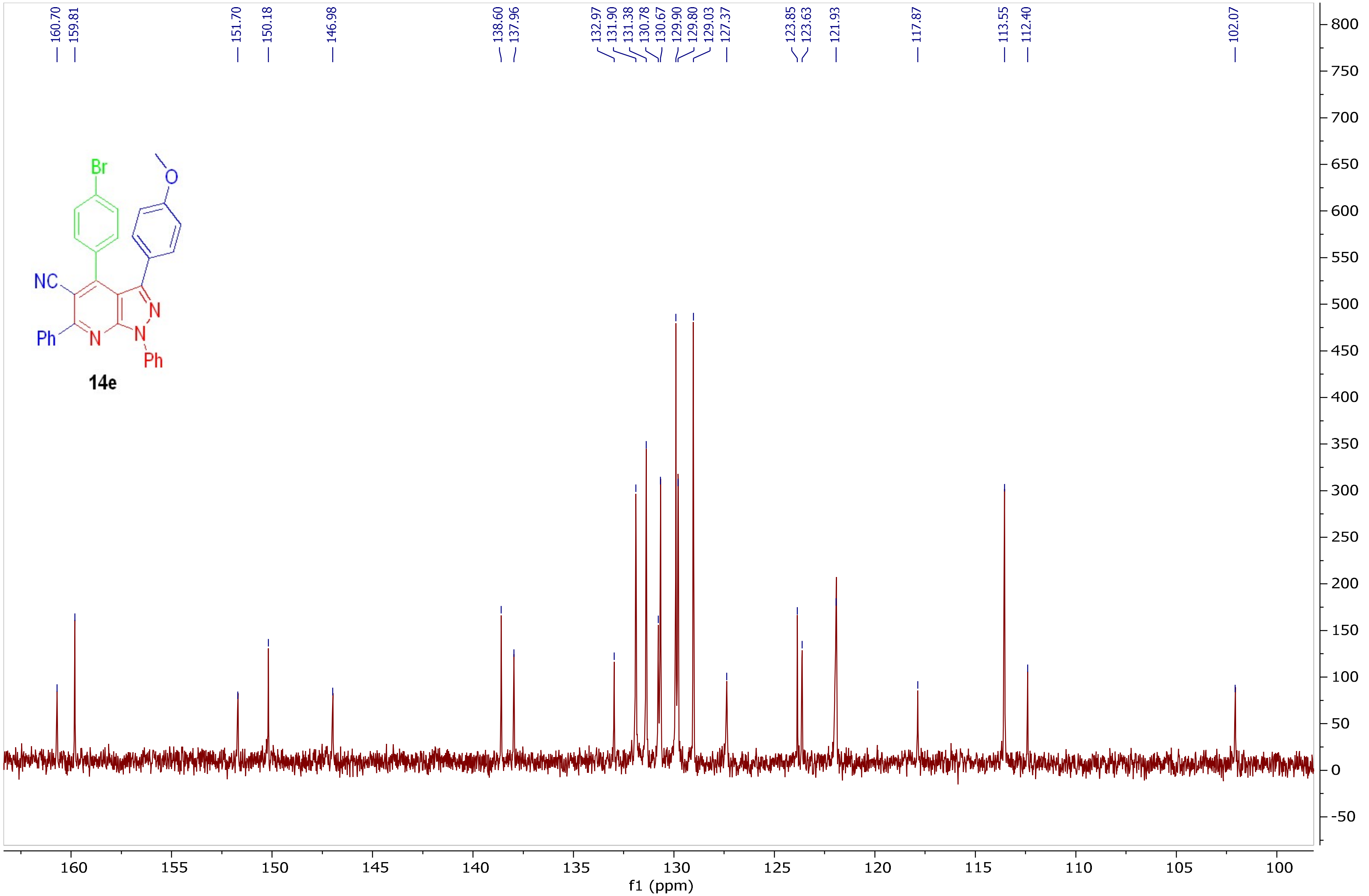

Figure S49. ^{13}C NMR of compound **14e**, aromatic region

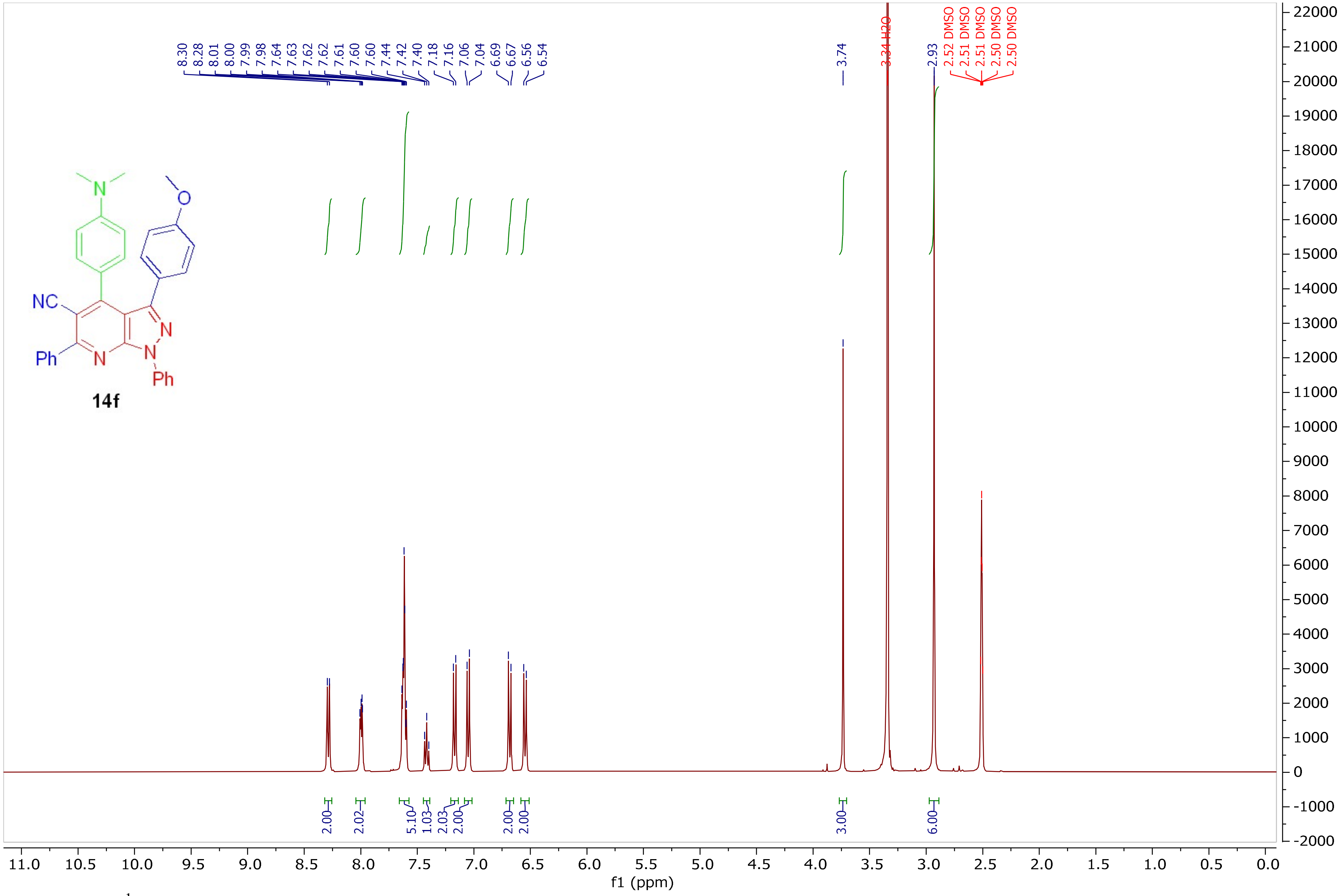

Figure S50. ^{1}H NMR of compound **14f**, full spectrum

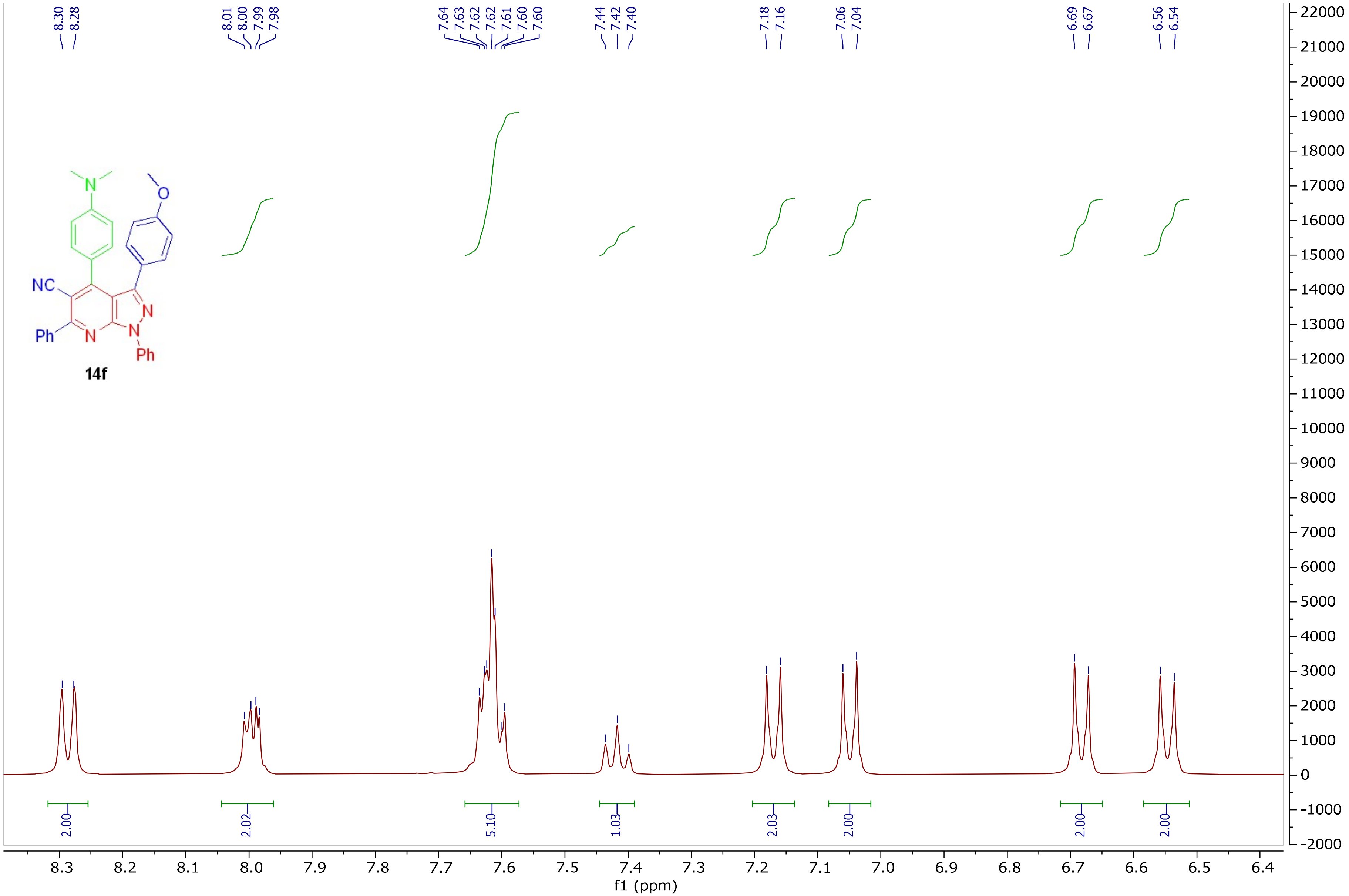

Figure S51. ^{1}H NMR of compound **14f**, aromatic region

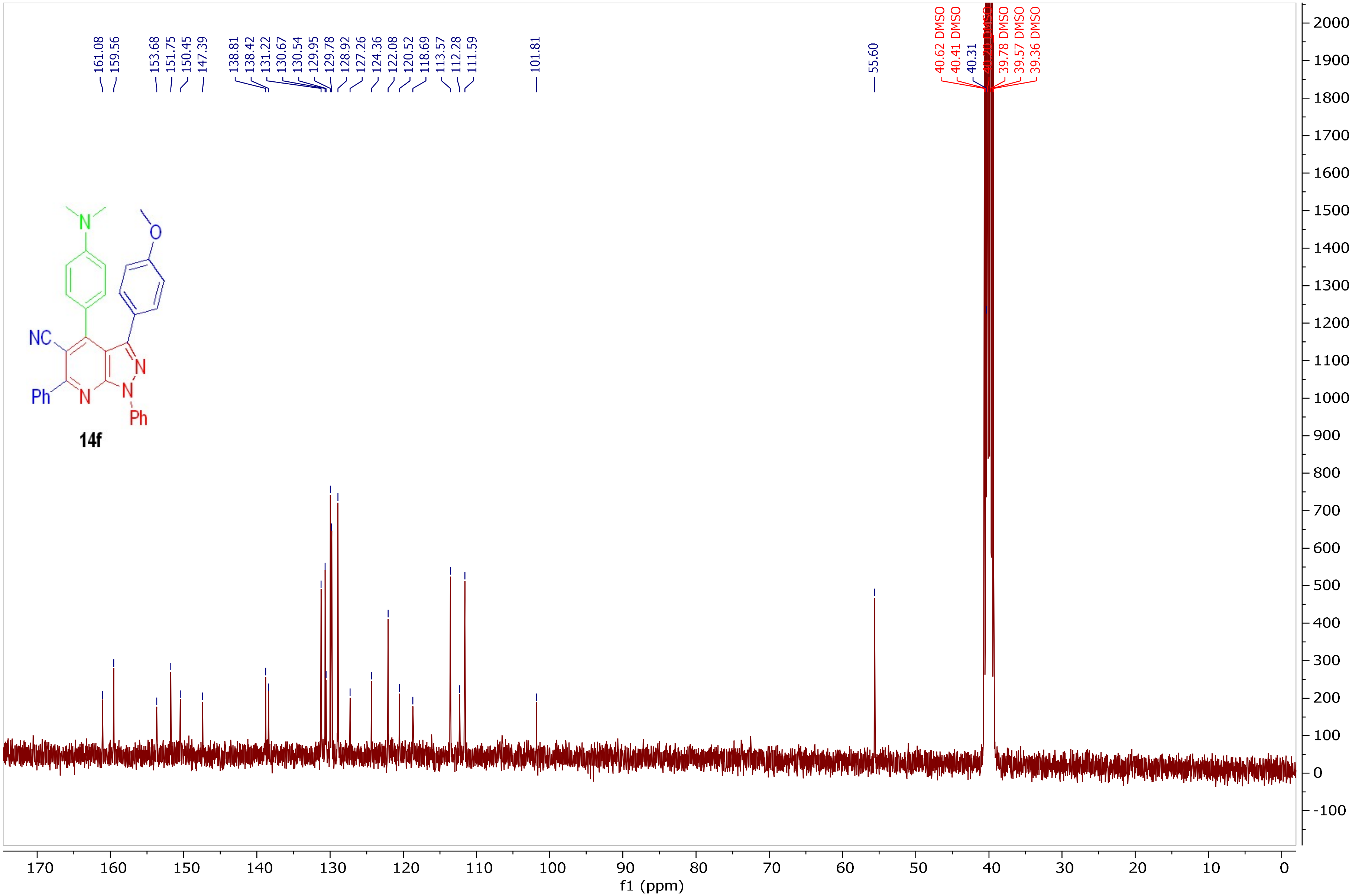

Figure S52. ^{13}C NMR of compound **14f**, full spectrum

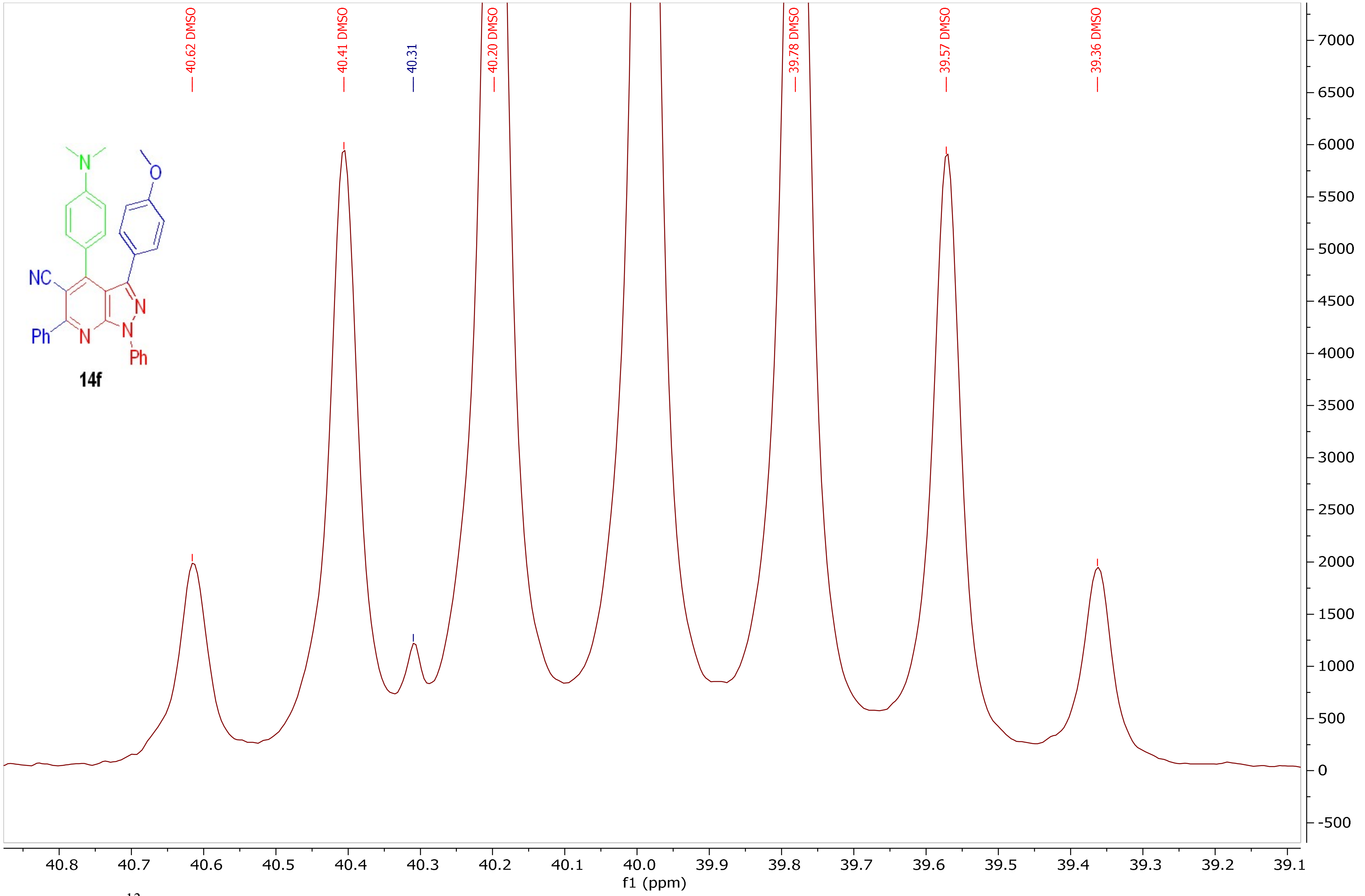

Figure S53. ^{13}C NMR of compound **14f**, aliphatic region

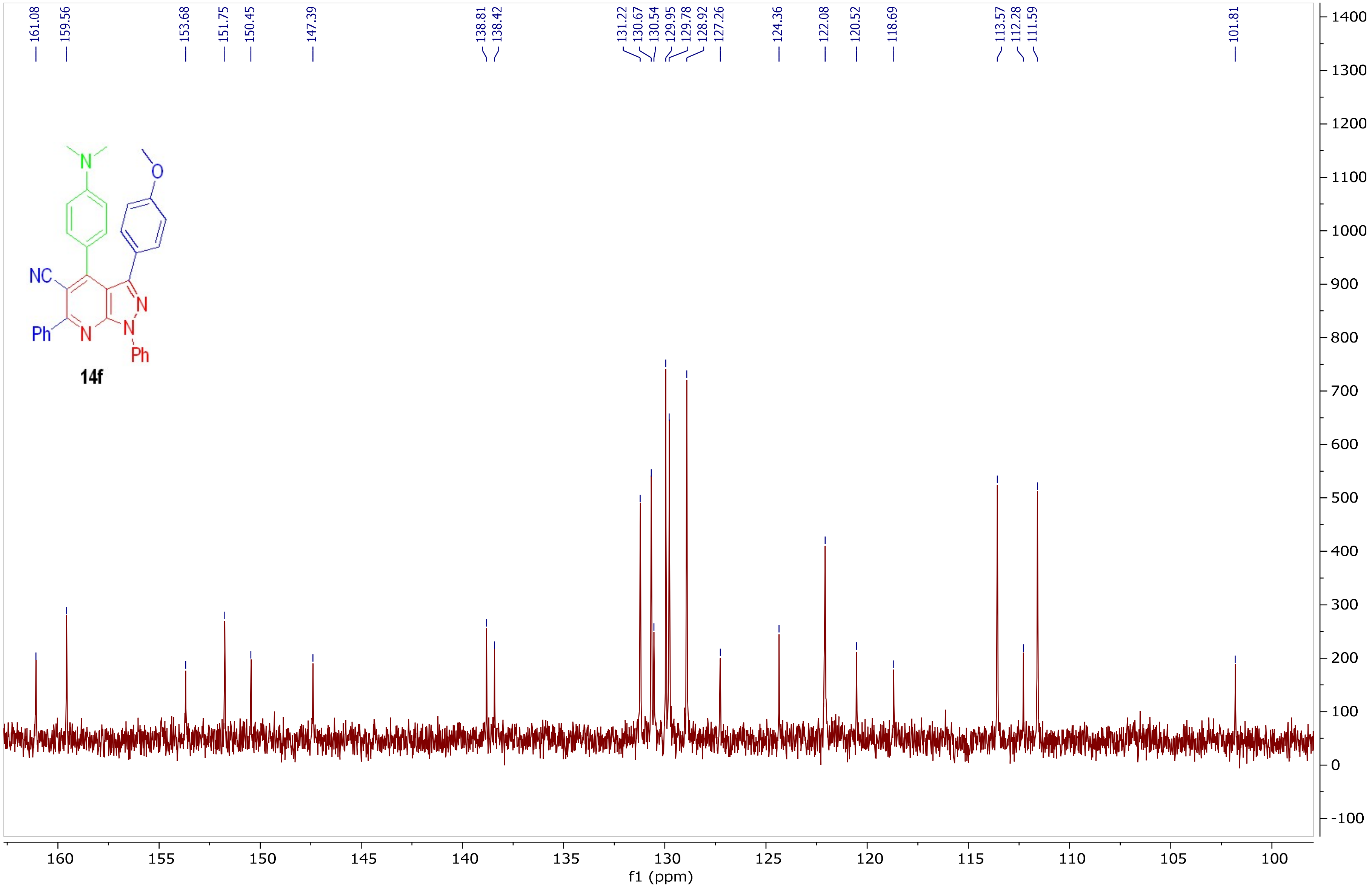

Figure S54. ^{13}C NMR of compound **14f**, aromatic region

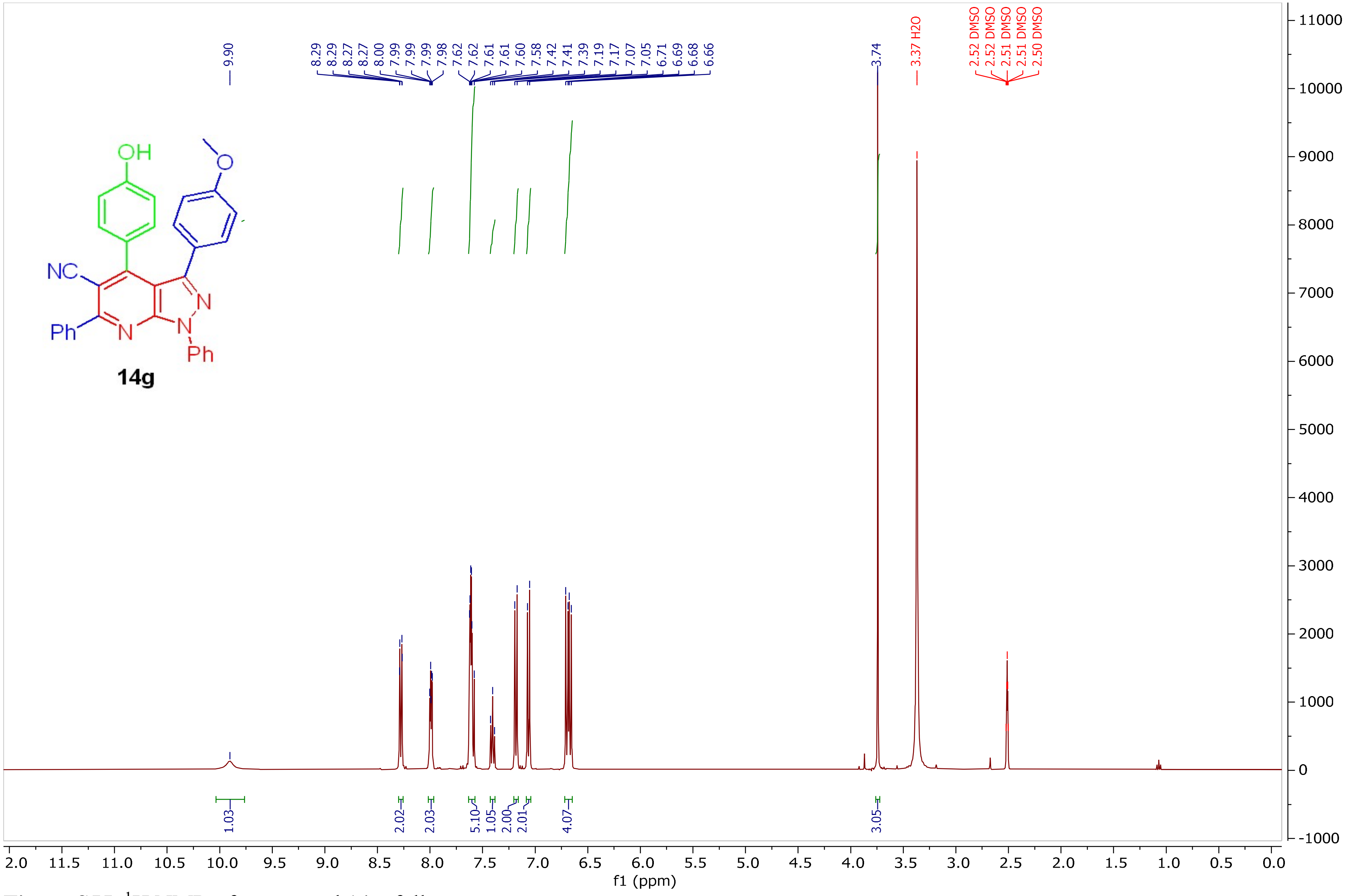

Figure S55. ^{1}H NMR of compound **14g**, full spectrum

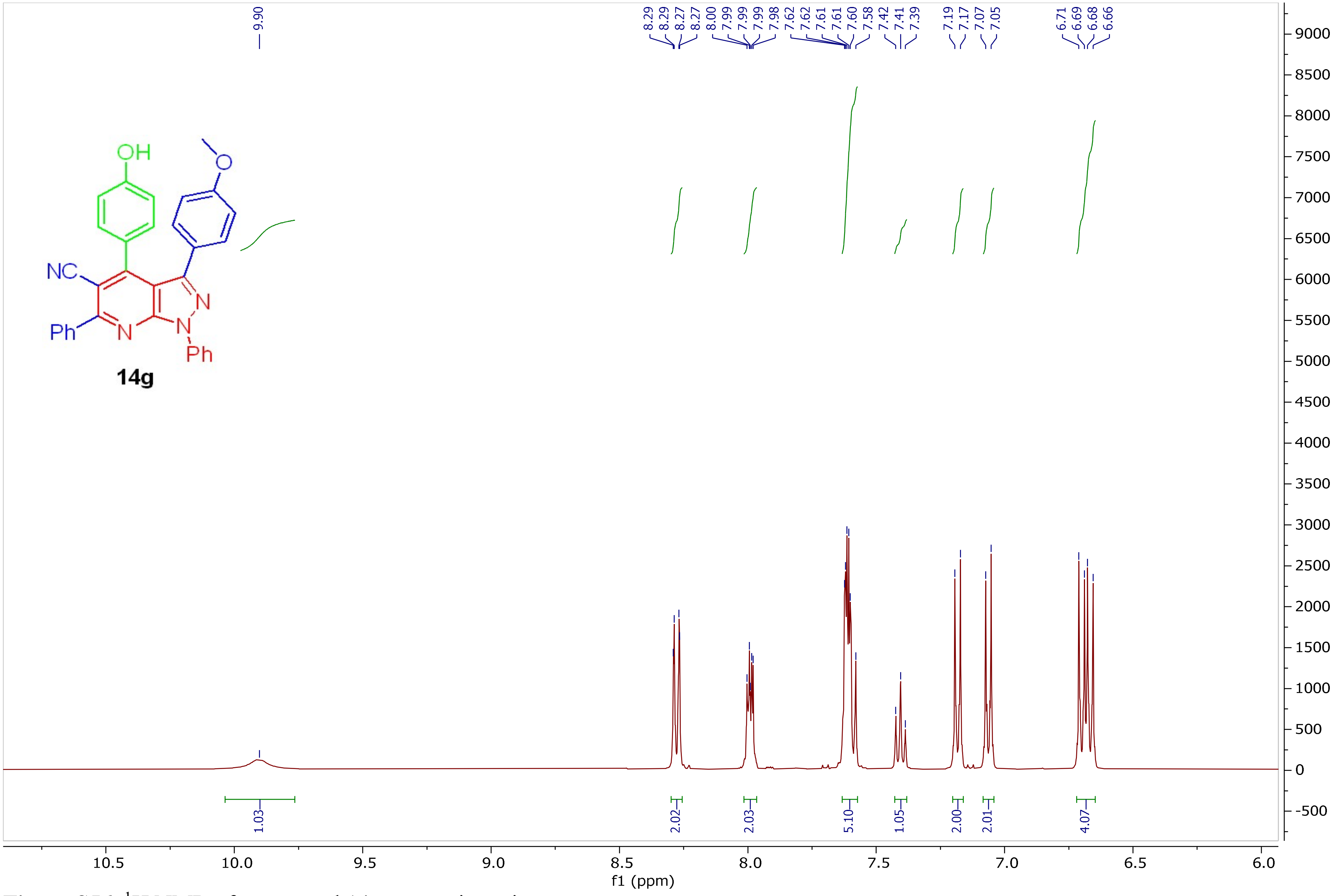

Figure S56. ^{1}H NMR of compound **14g**, aromatic region

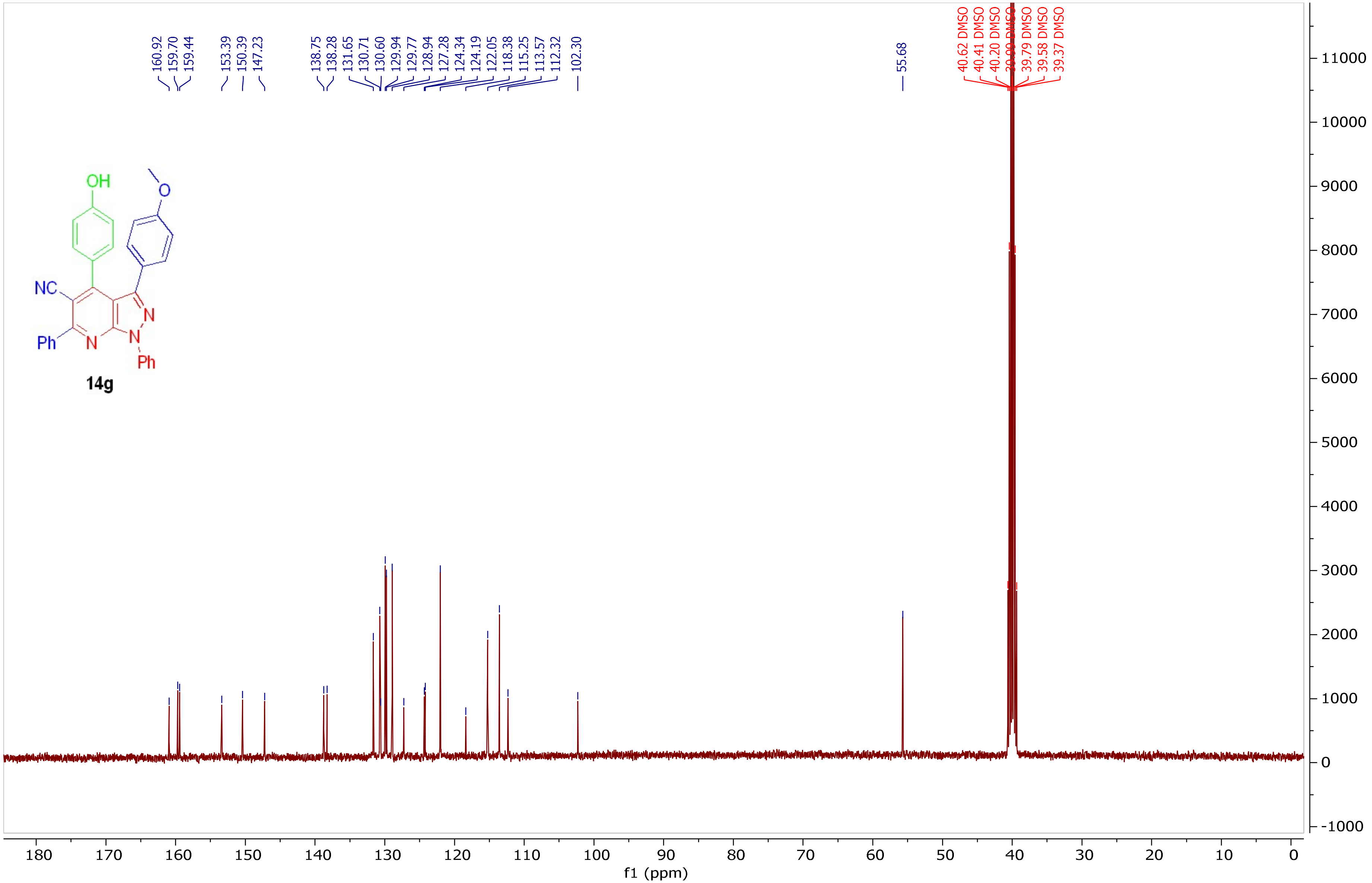

Figure S57. ^{13}C NMR of compound **14g**, full spectrum

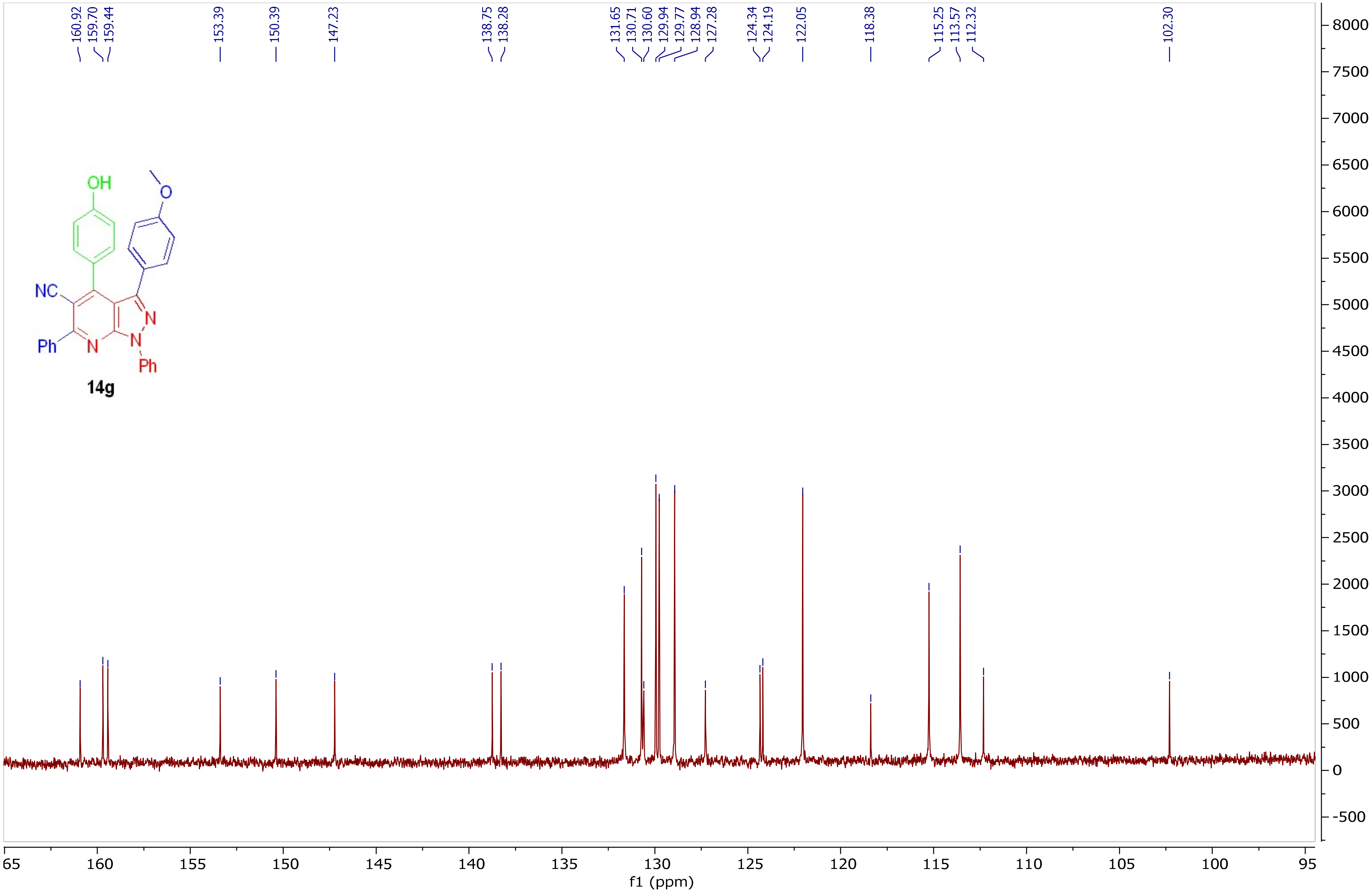

Figure S58. ^{13}C NMR of compound **14g**, aromatic region

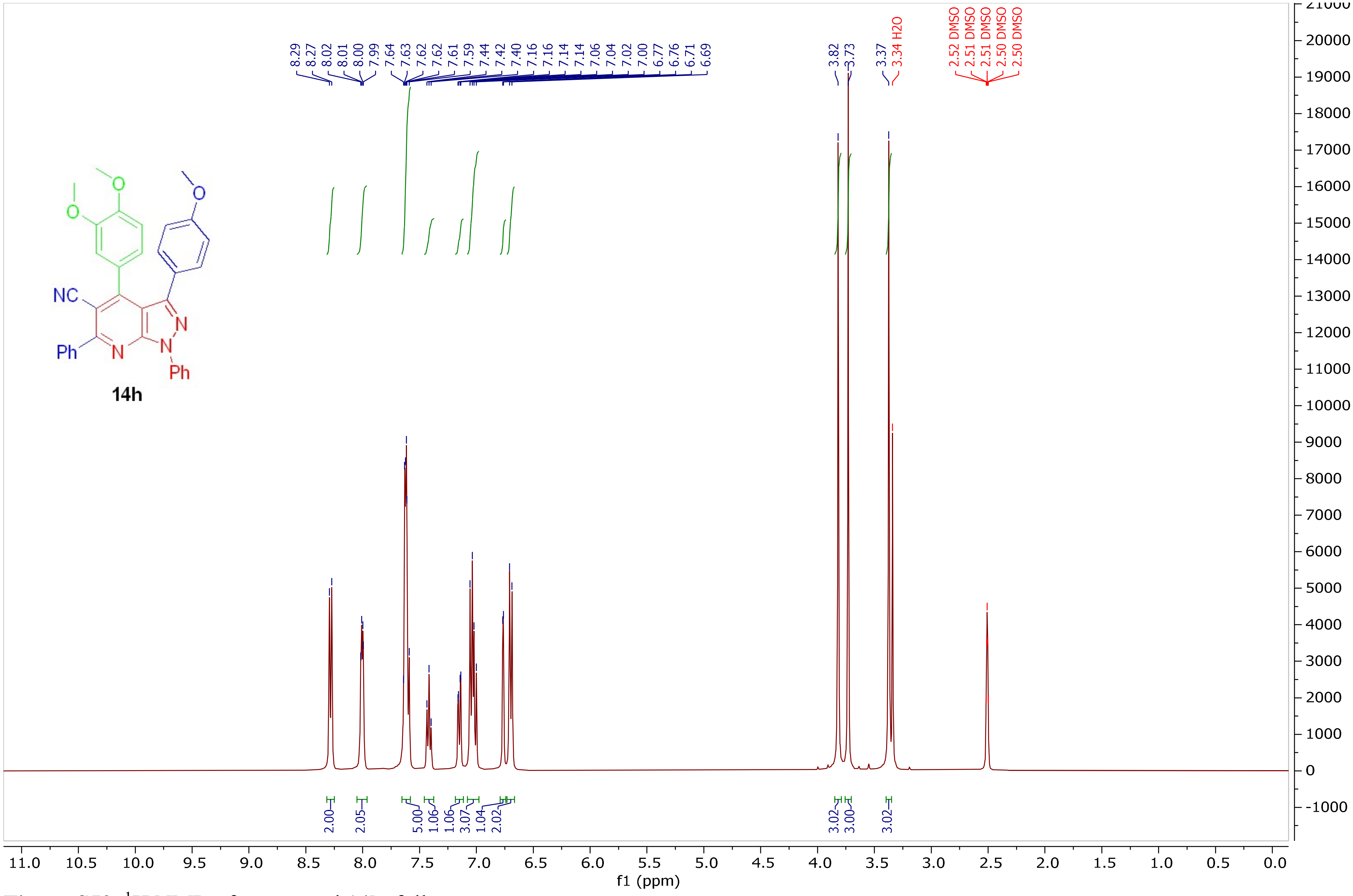

Figure S59. ^{1}H NMR of compound **14h**, full spectrum

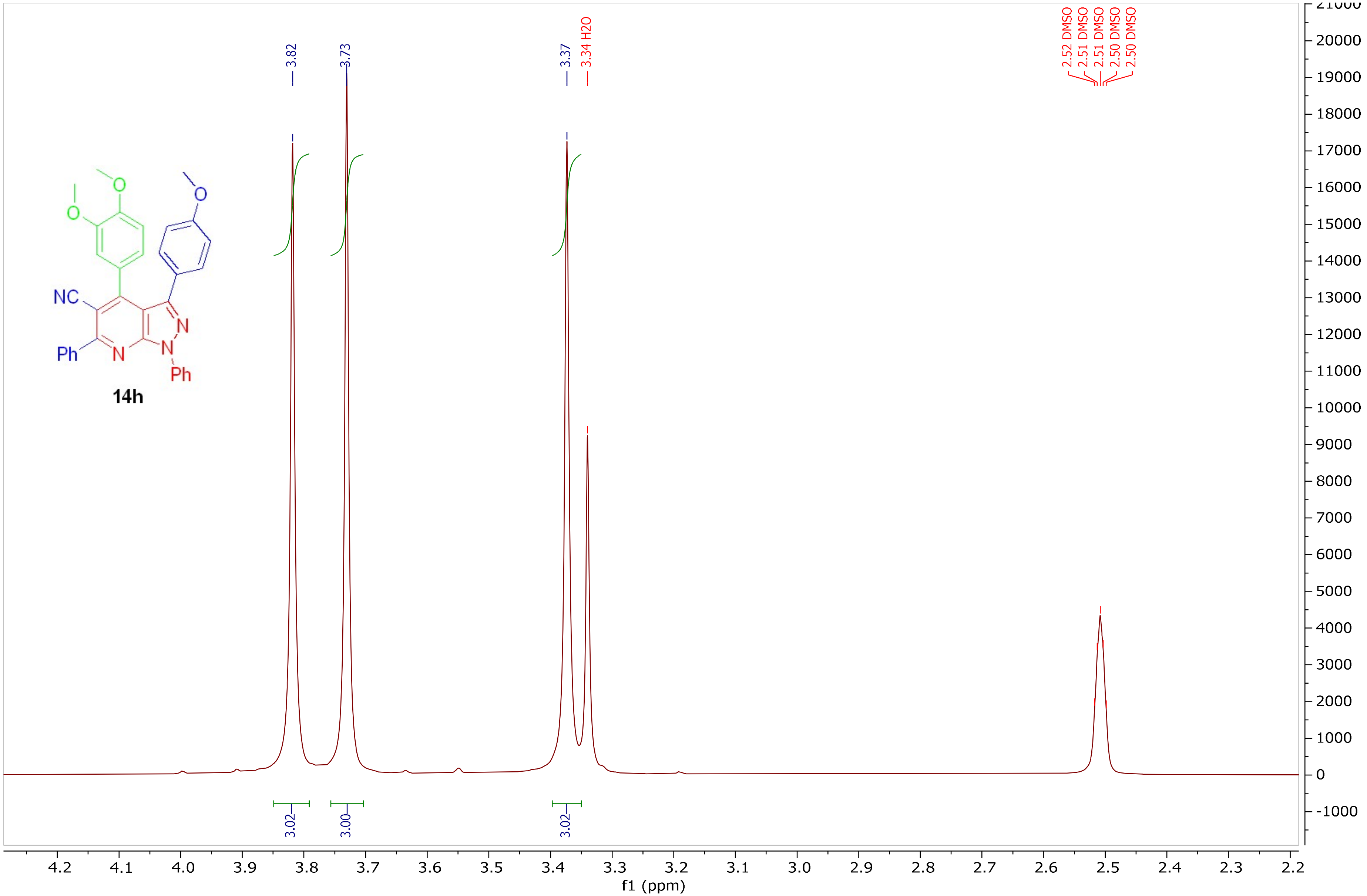

Figure S60. ^{1}H NMR of compound **14h**, aliphatic region

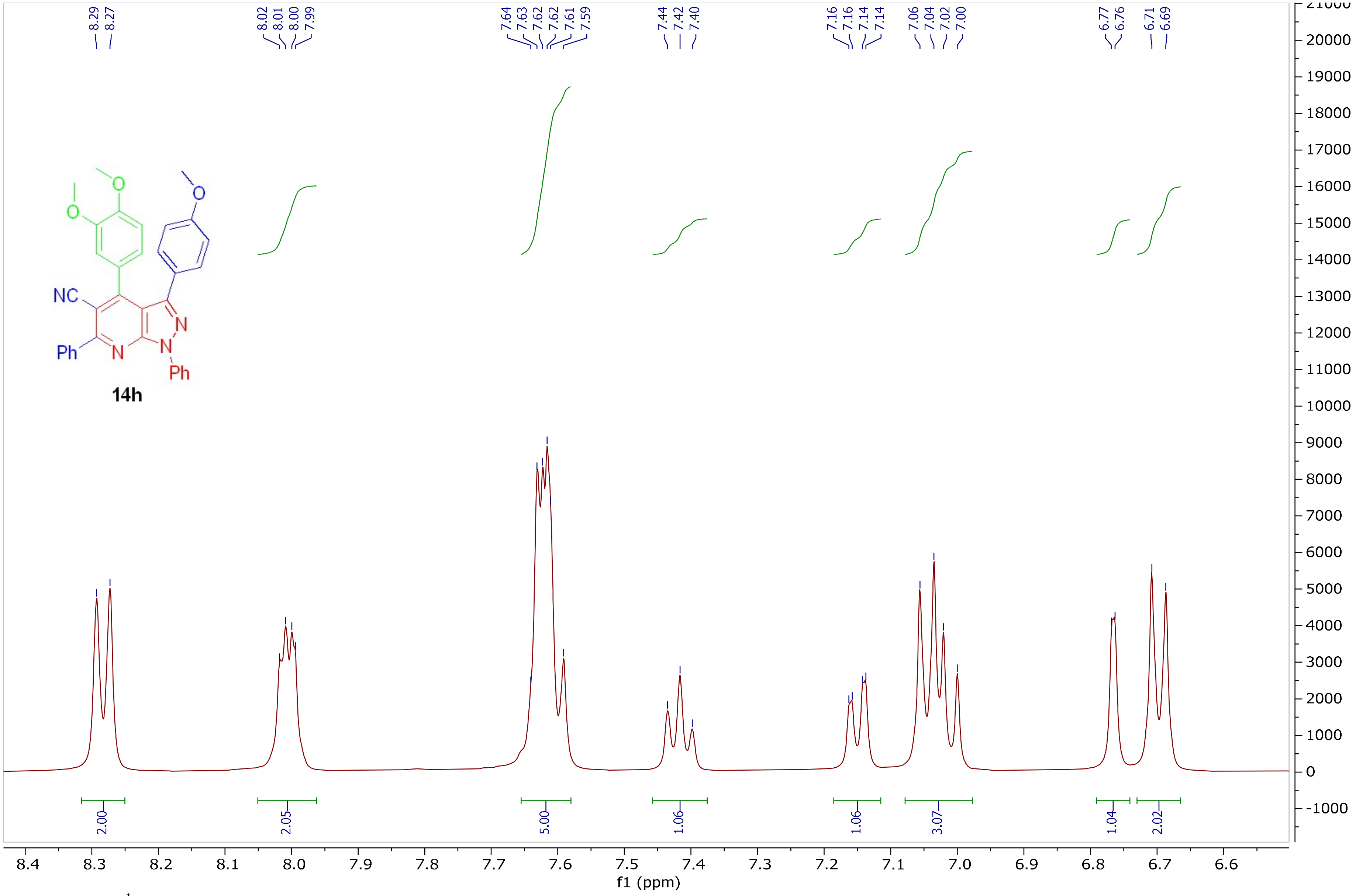

Figure S61. ^{1}H NMR of compound **14h**, aromatic region

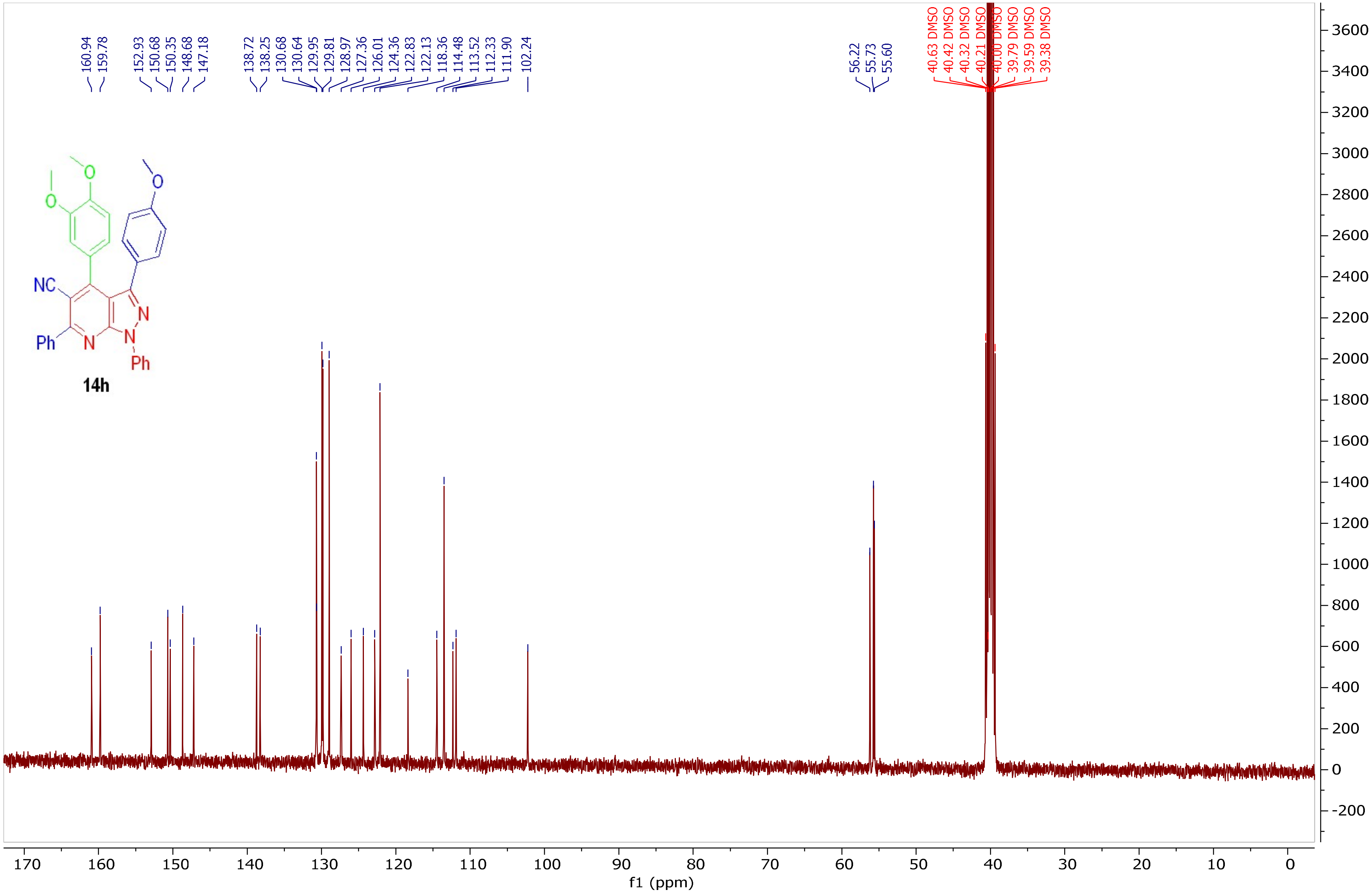

Figure S62. ^{13}C NMR of compound **14h**, full spectrum

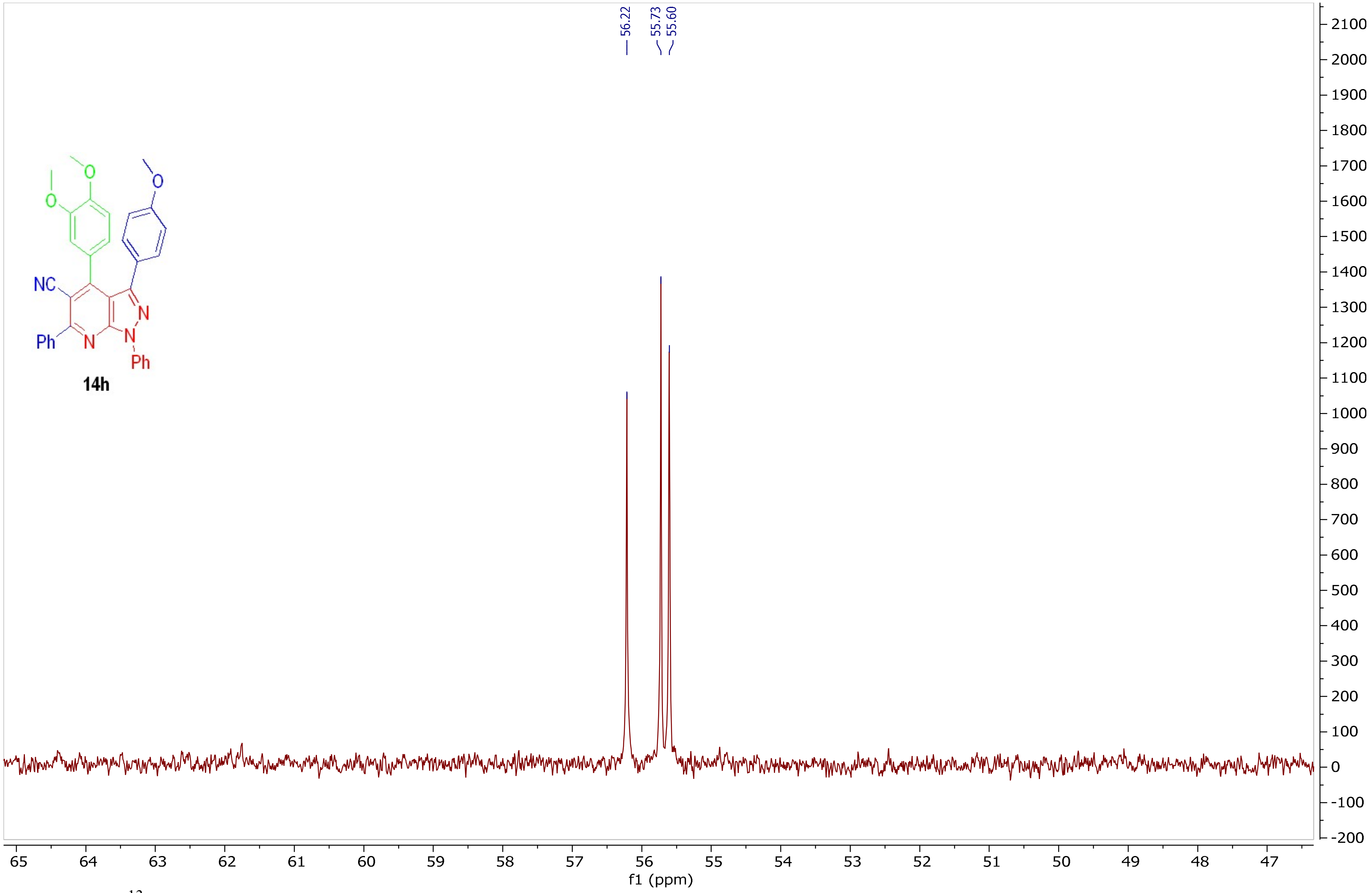

Figure S63. ^{13}C NMR of compound **14h**, aliphatic region

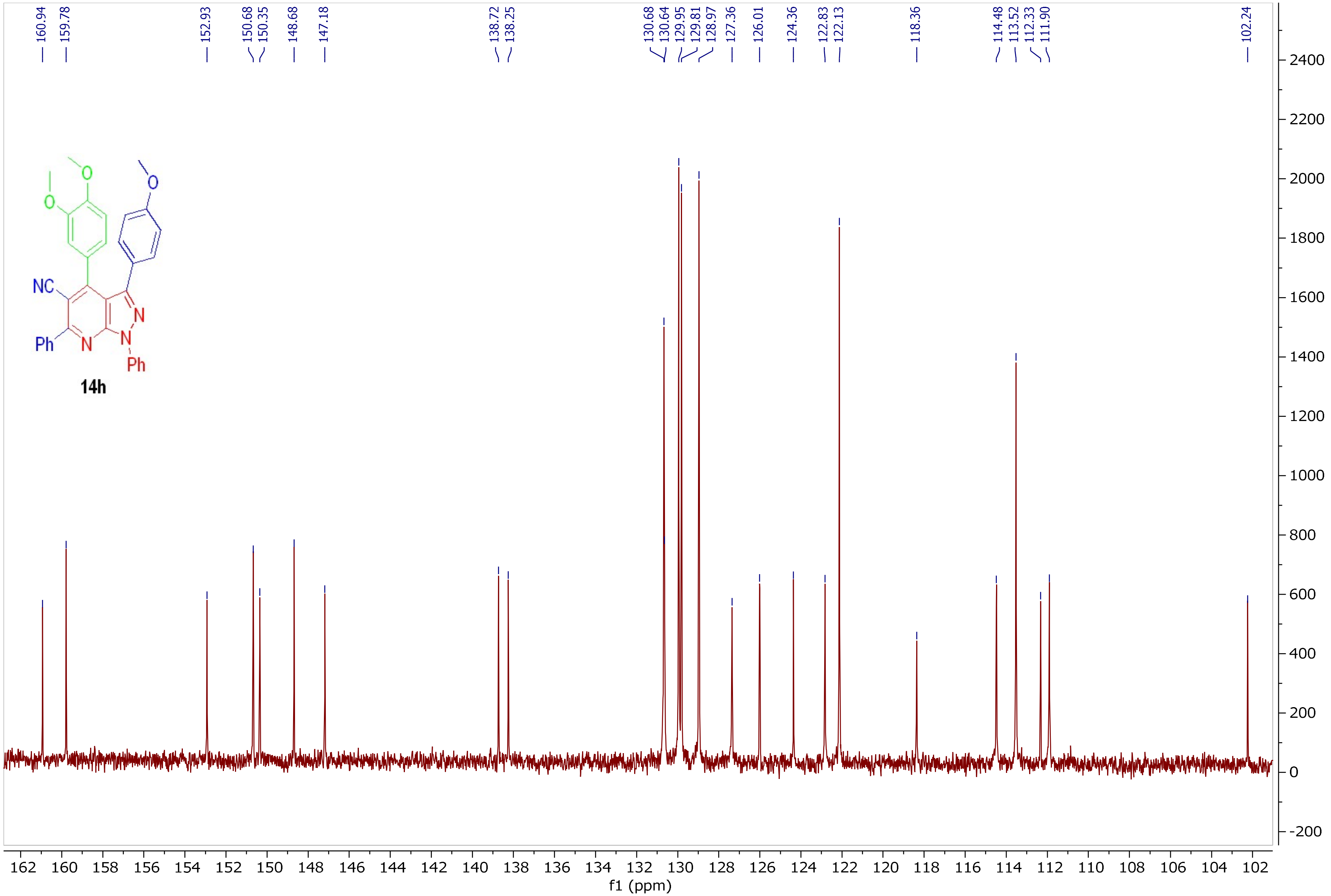

Figure S64. ^{13}C NMR of compound **14h**, aromatic region

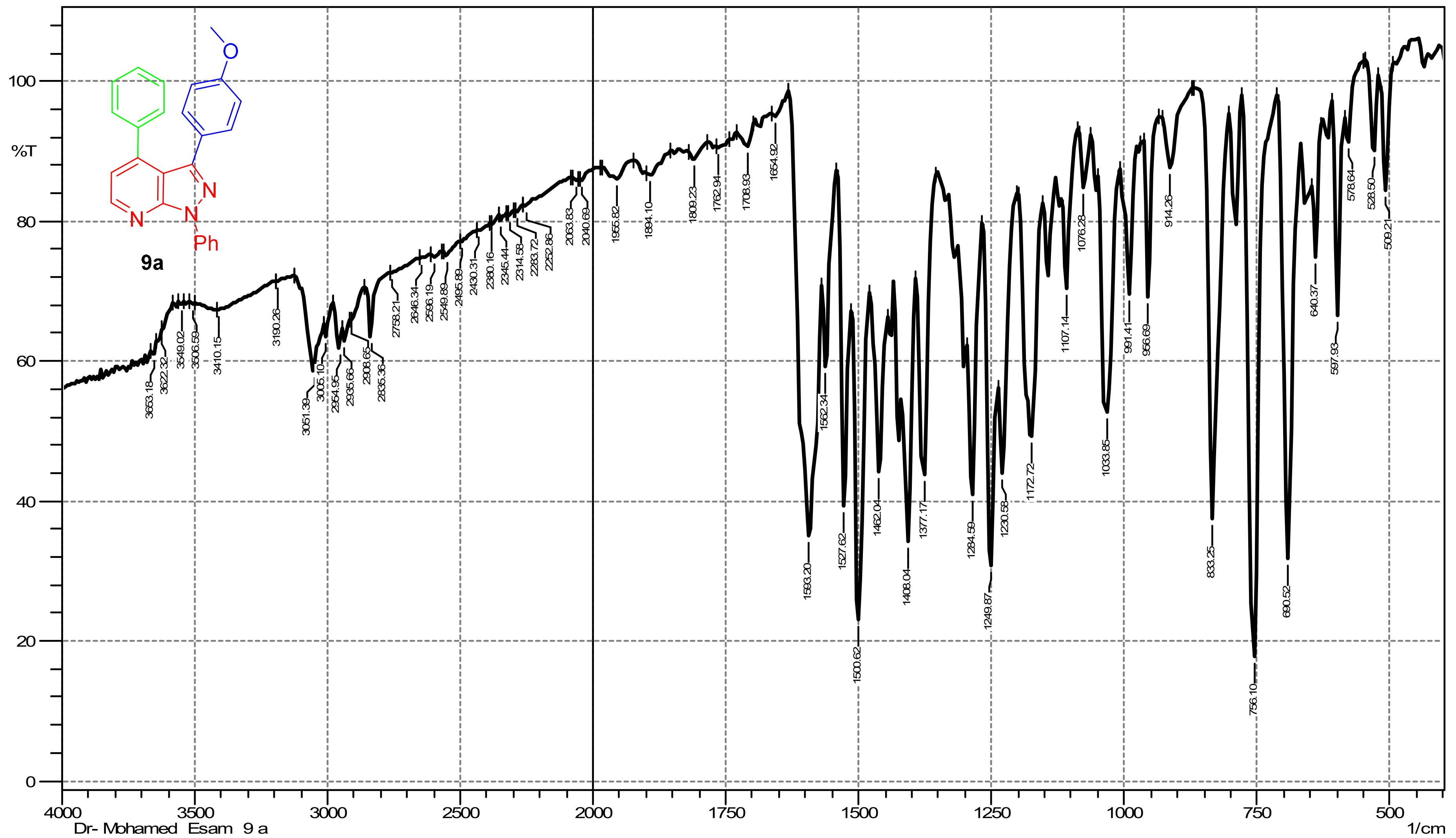

Figure S65. IR of compound **9a**

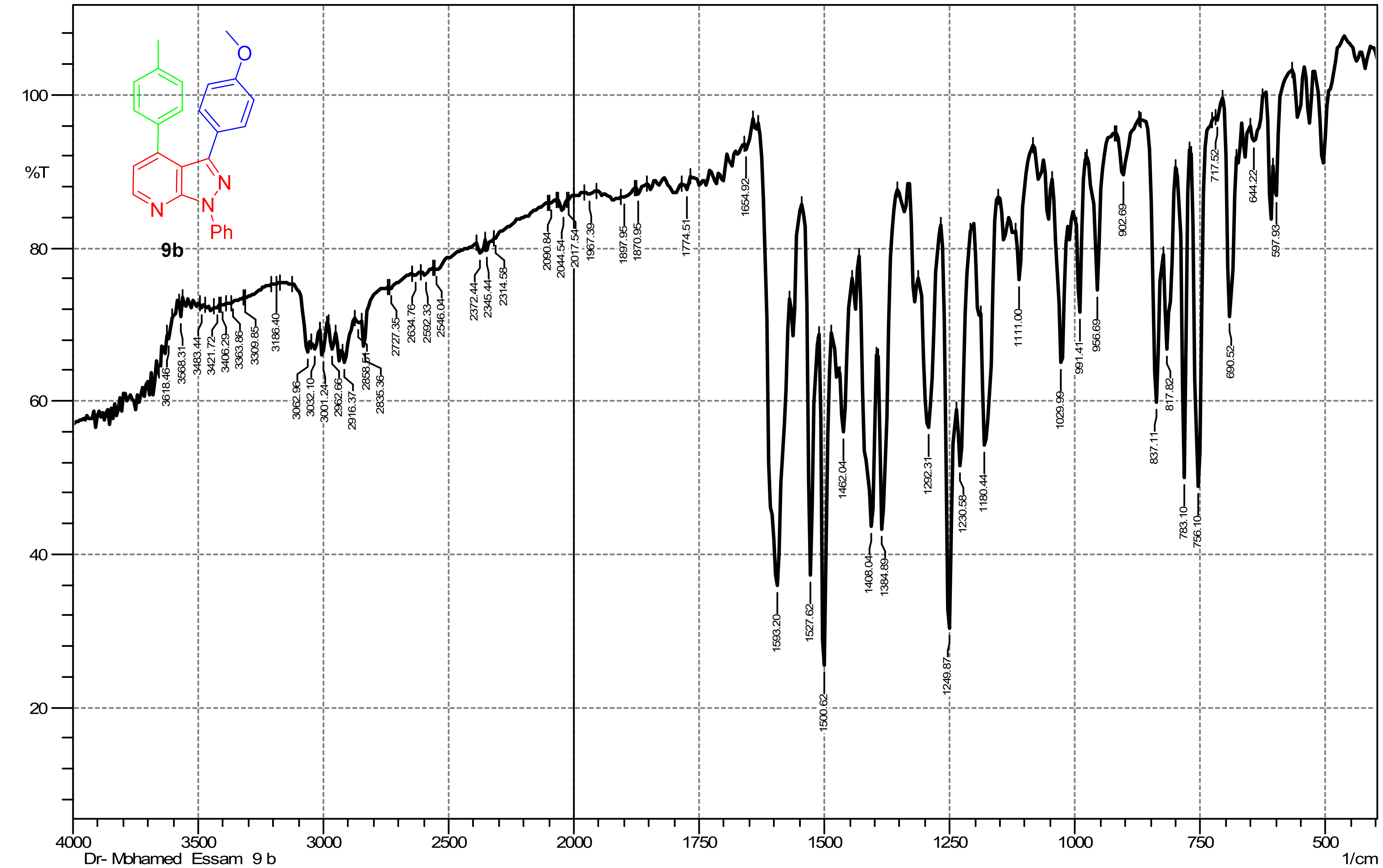

Figure S66. IR of compound **9b**

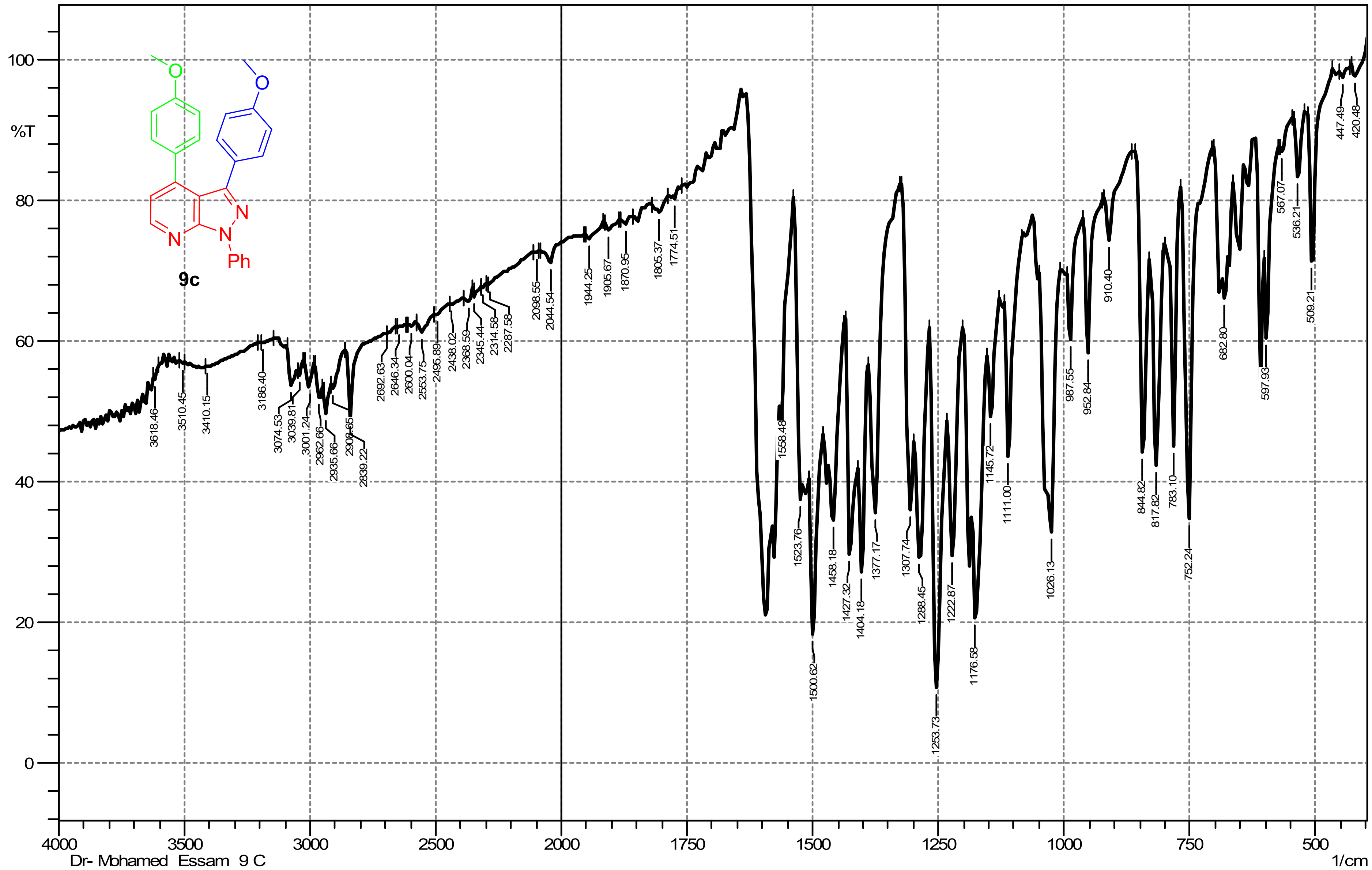

Figure S67. IR of compound **9c**

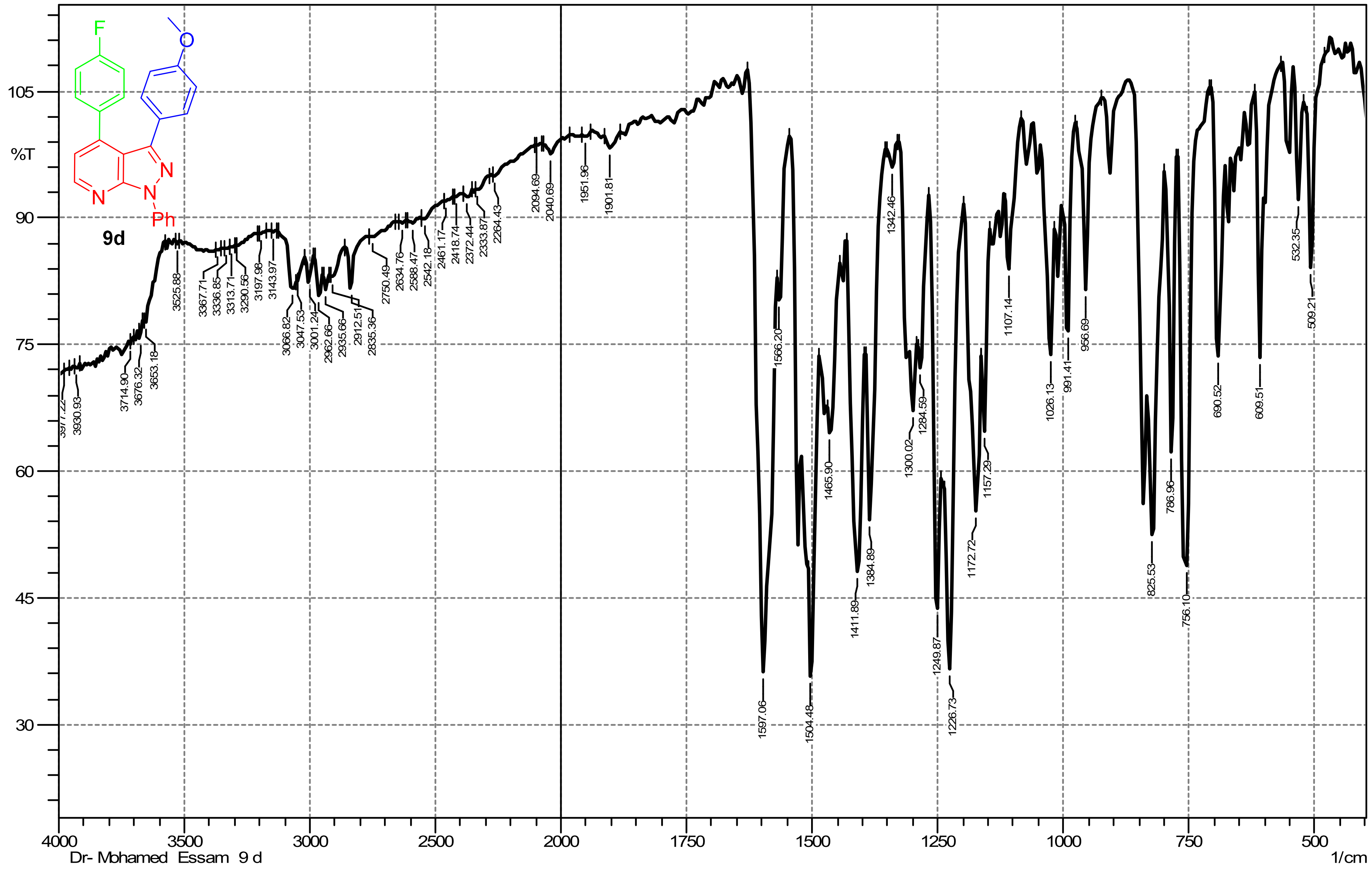

Figure S68. IR of compound **9d**

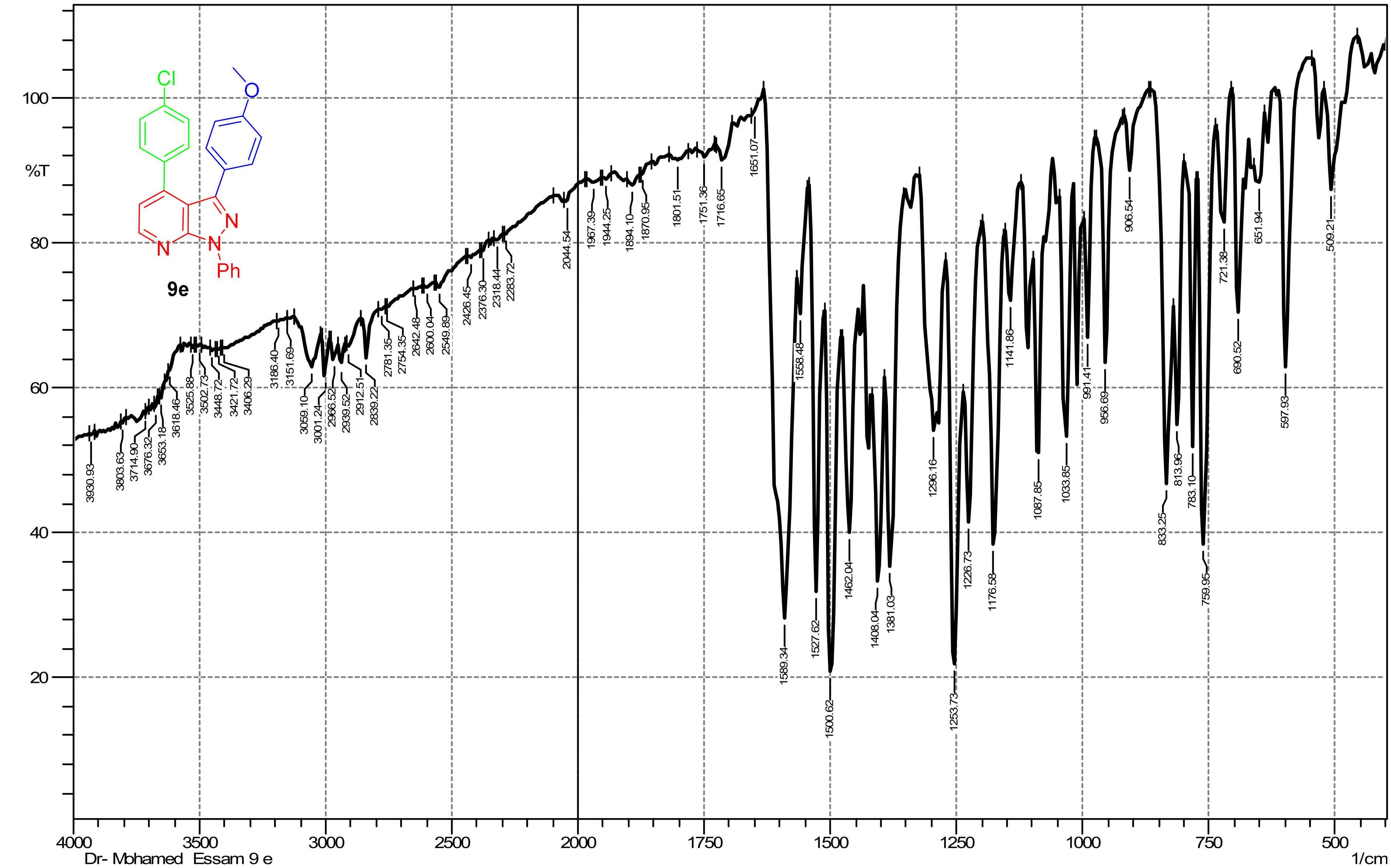

Figure S69. IR of compound **9e**

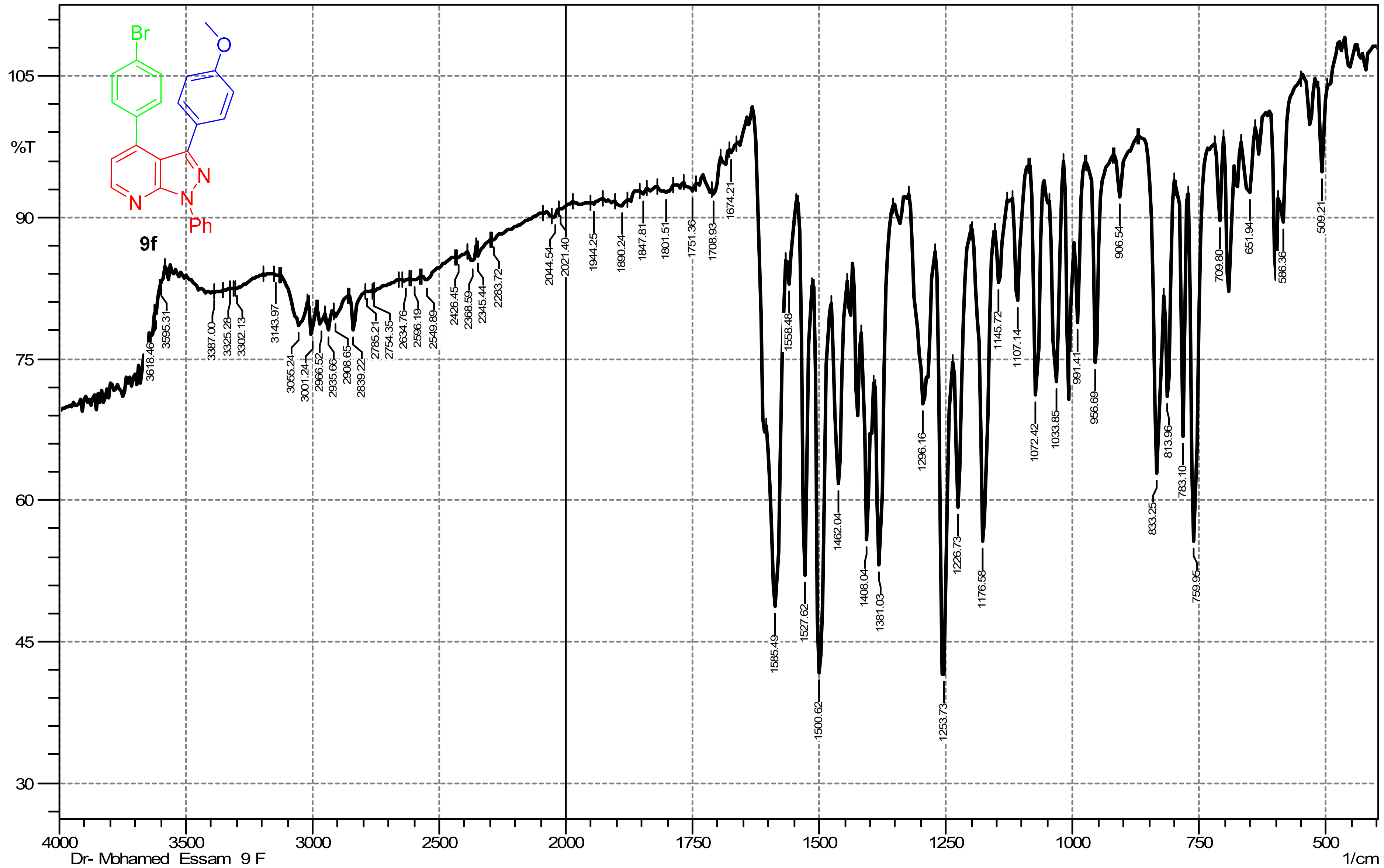

Figure S70. IR of compound **9f**

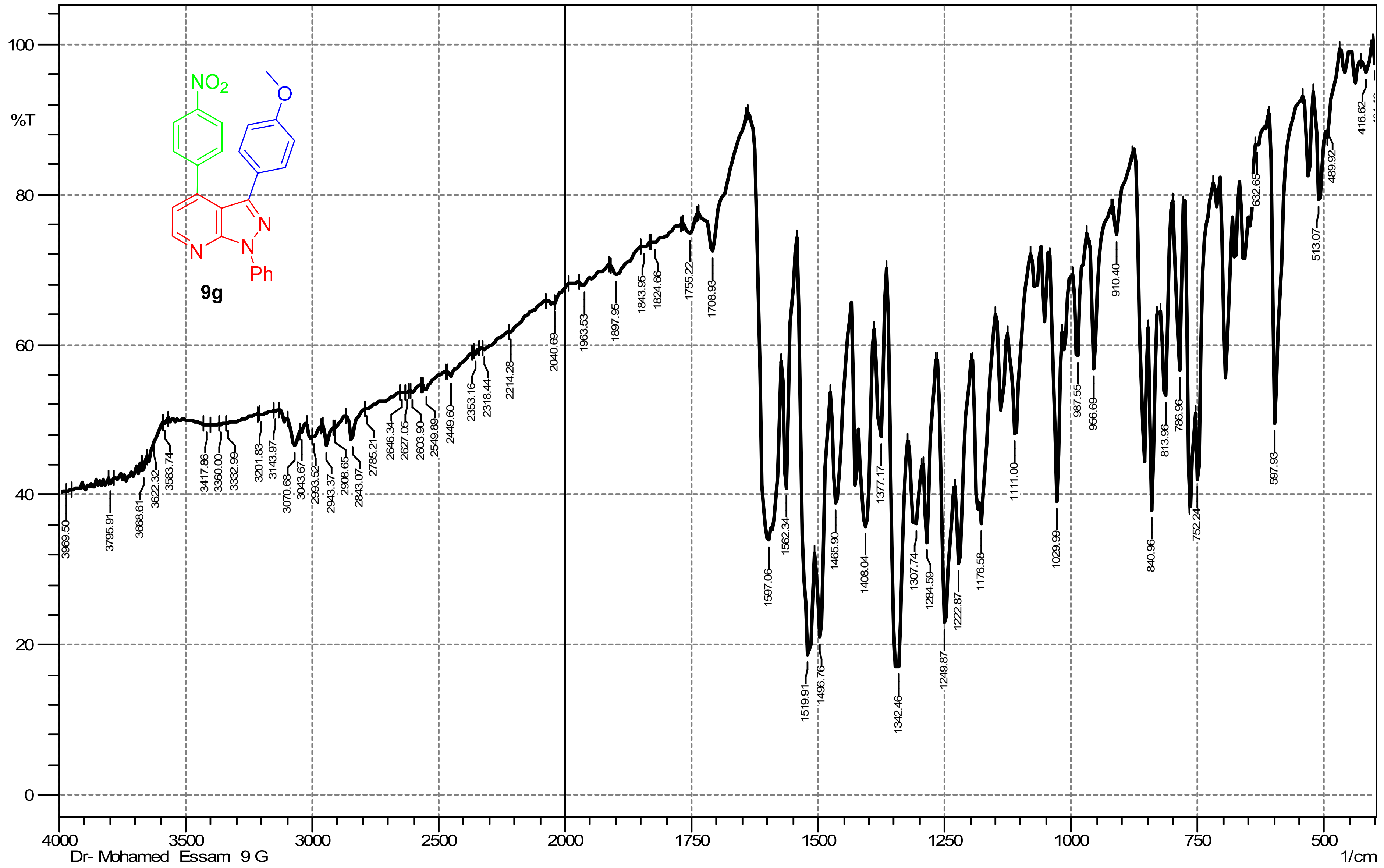

Figure S71. IR of compound **9g**

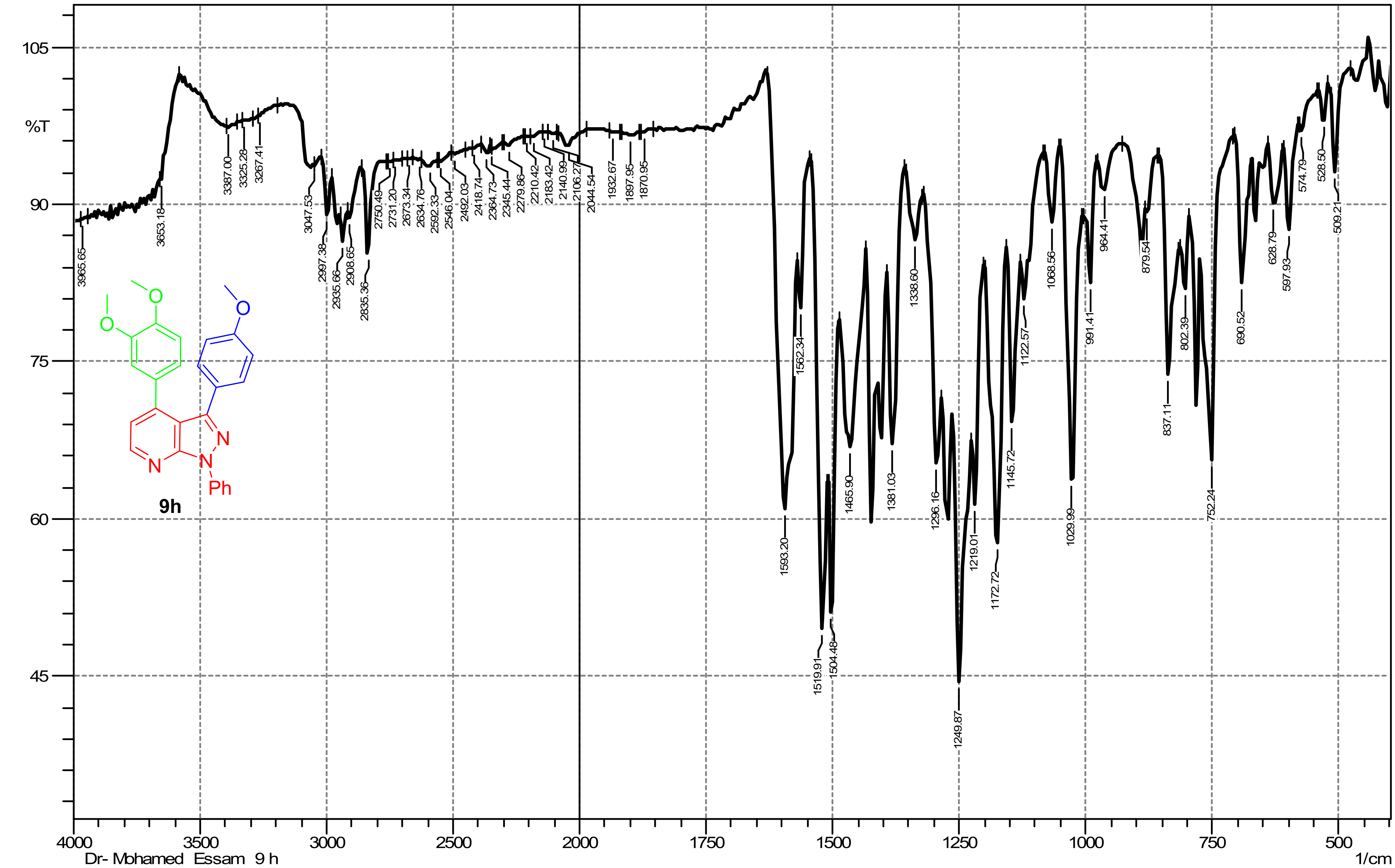

Figure S72. IR of compound **9h**

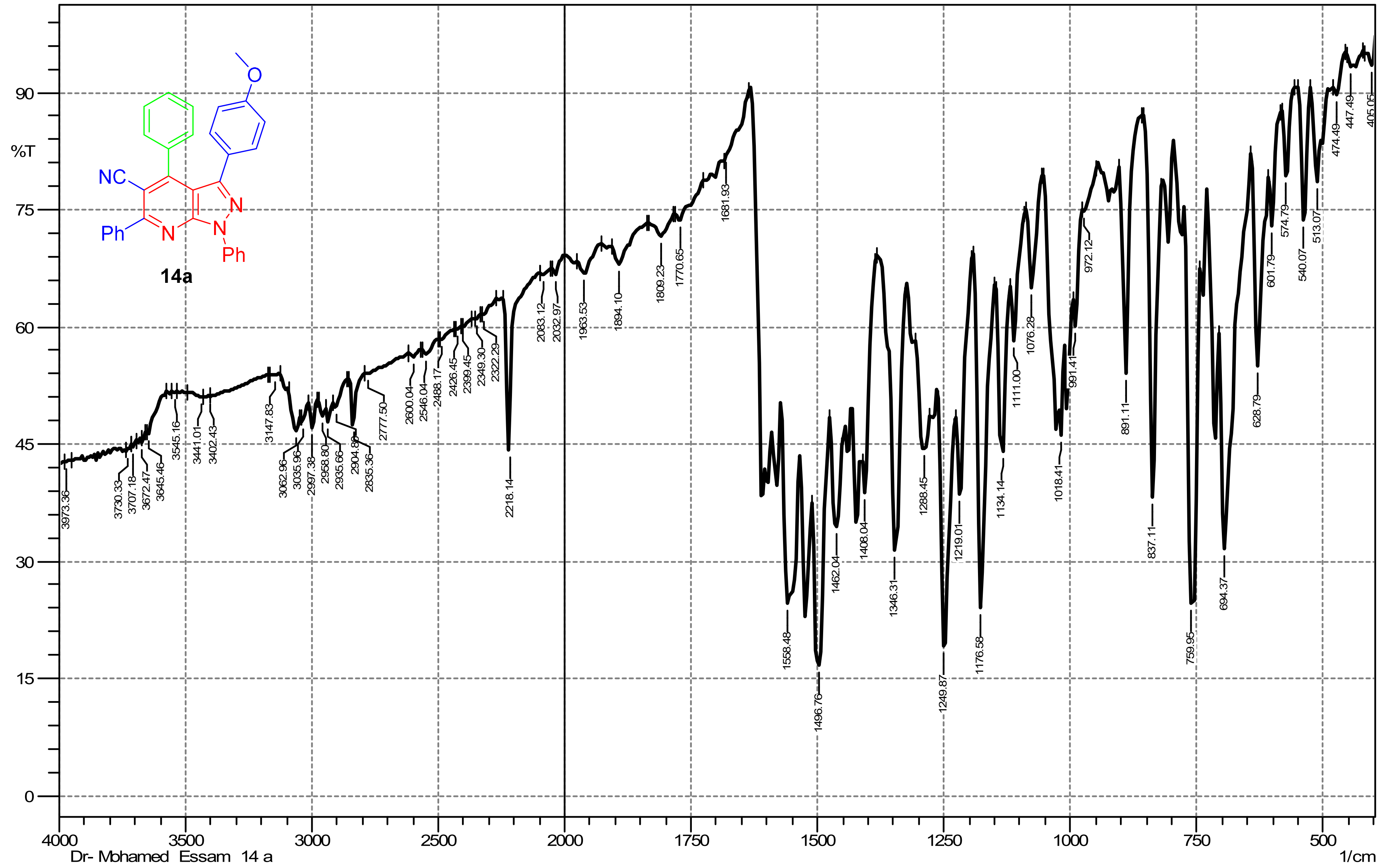

Figure S73. IR of compound **14a**

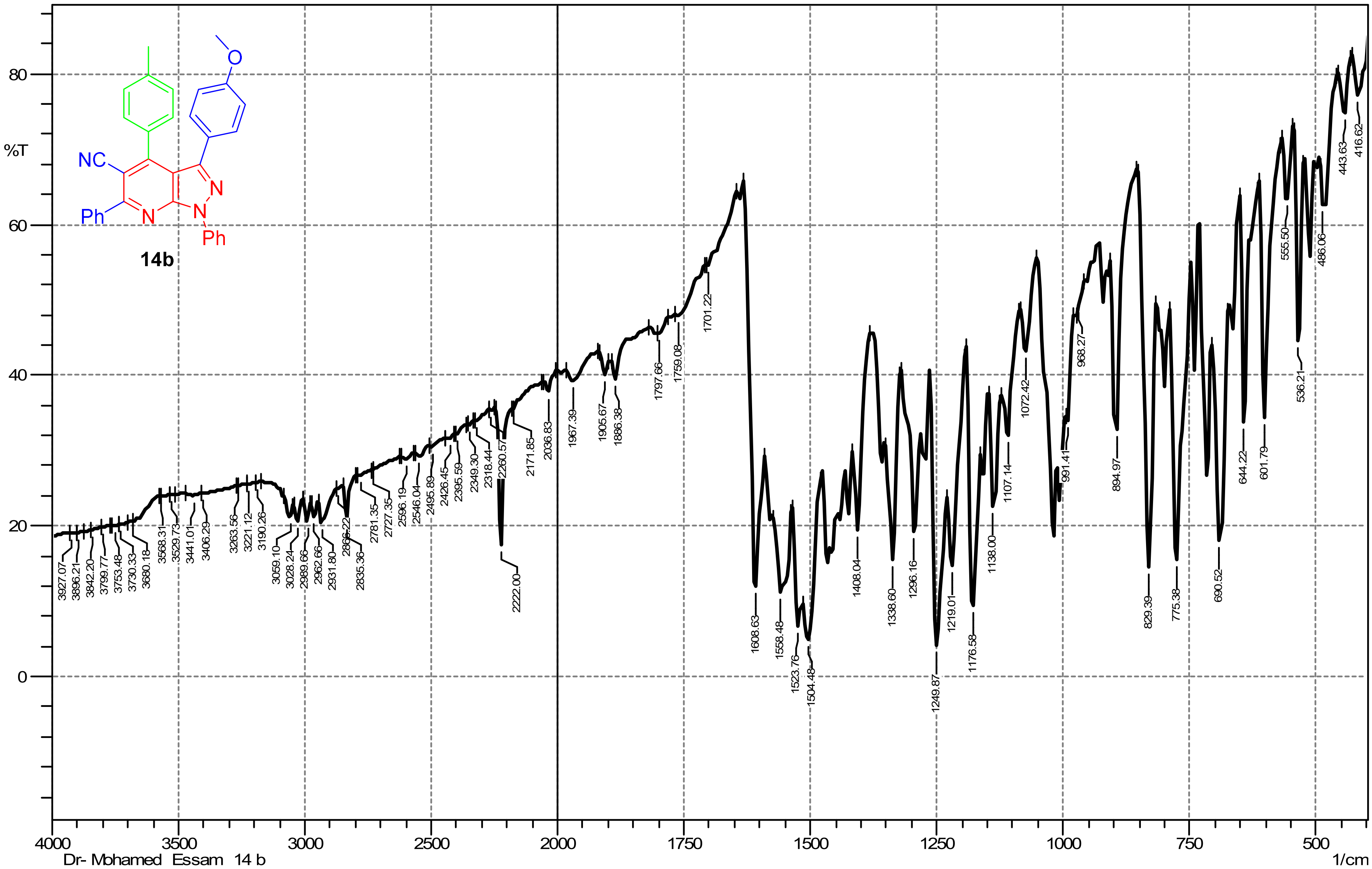

Figure S74. IR of compound **14b**

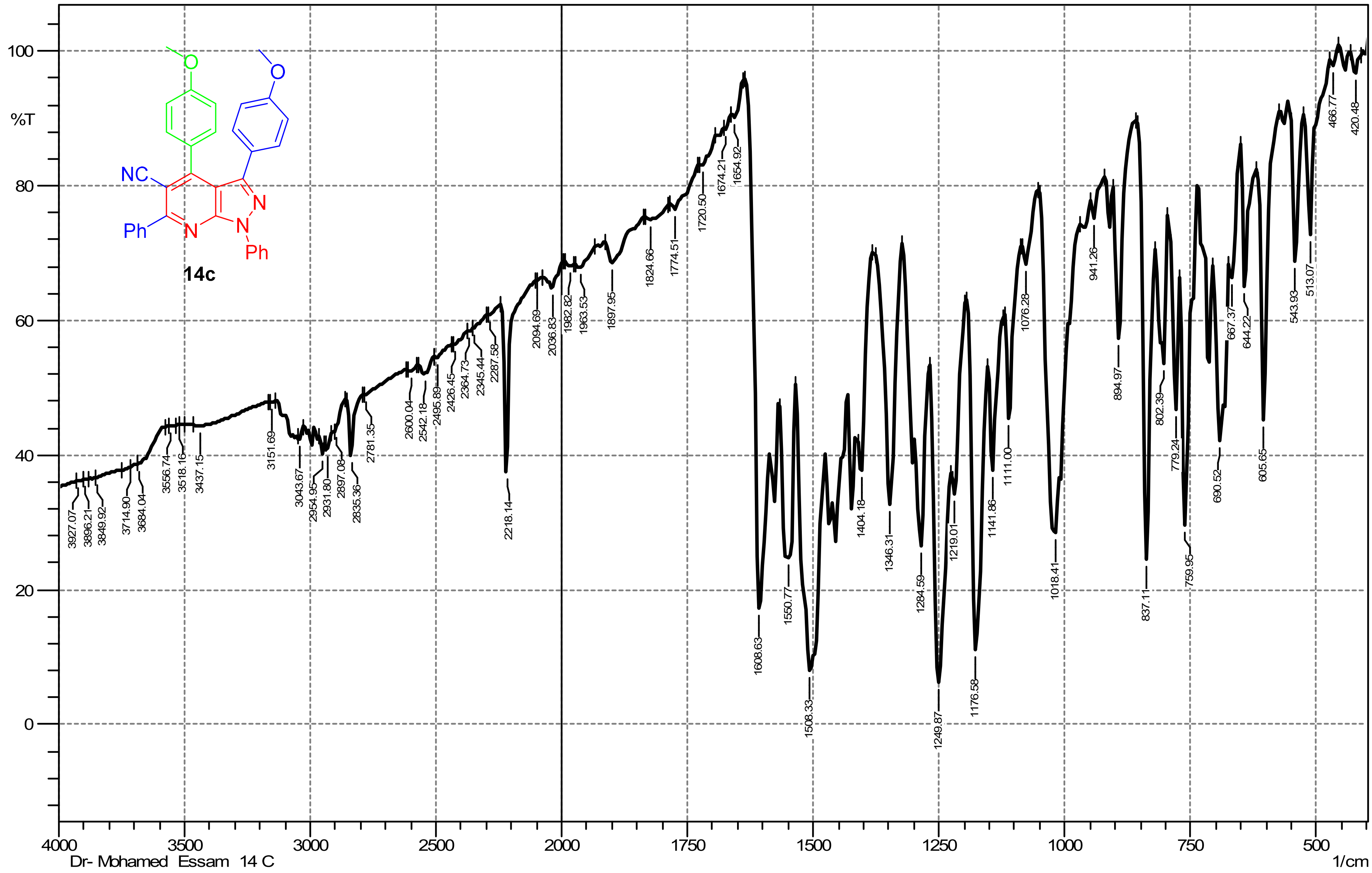

Figure S75. IR of compound **14c**

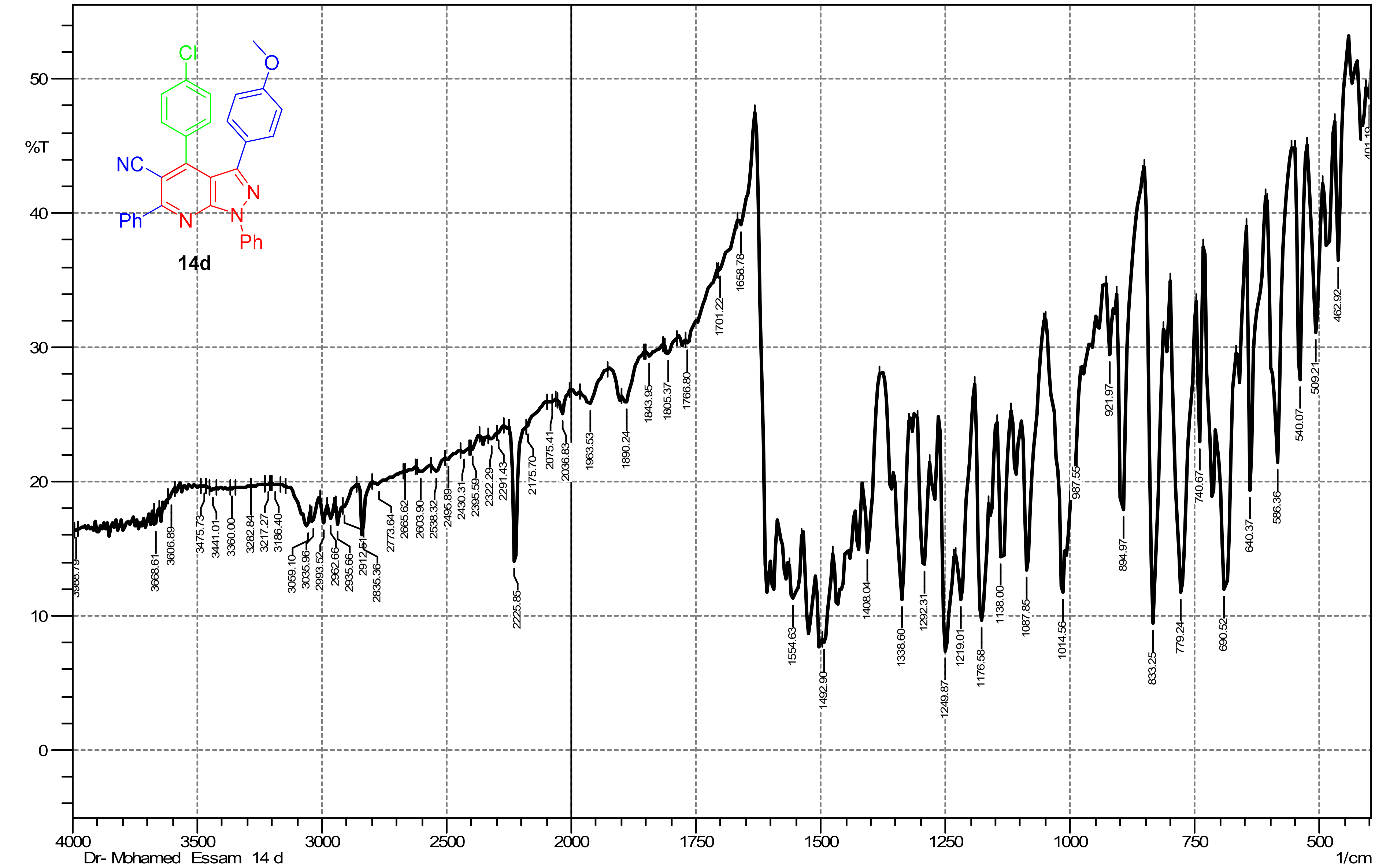

Figure S76. IR of compound **14d**

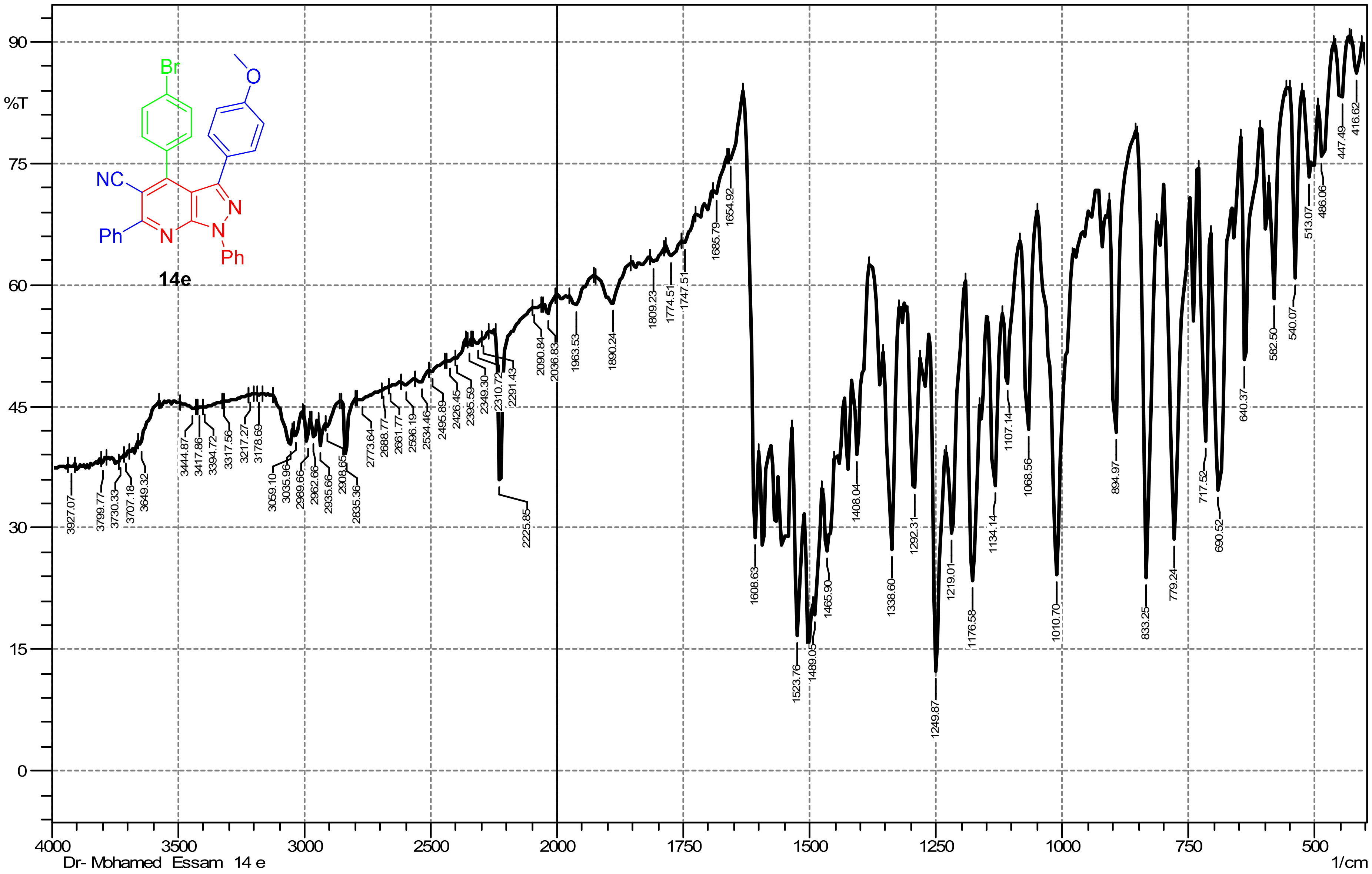

Figure S77. IR of compound **14e**

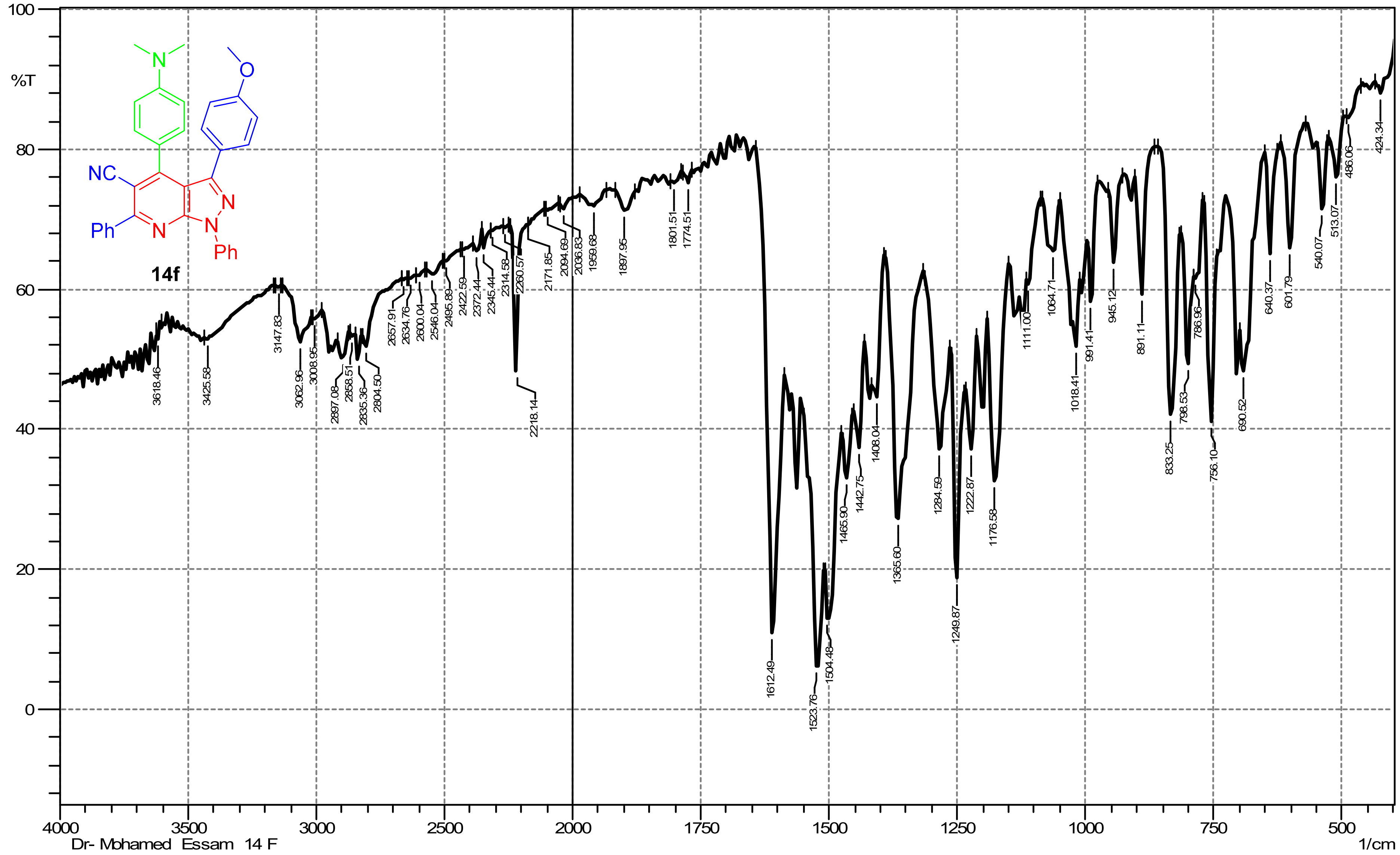

Figure S78. IR of compound 14f

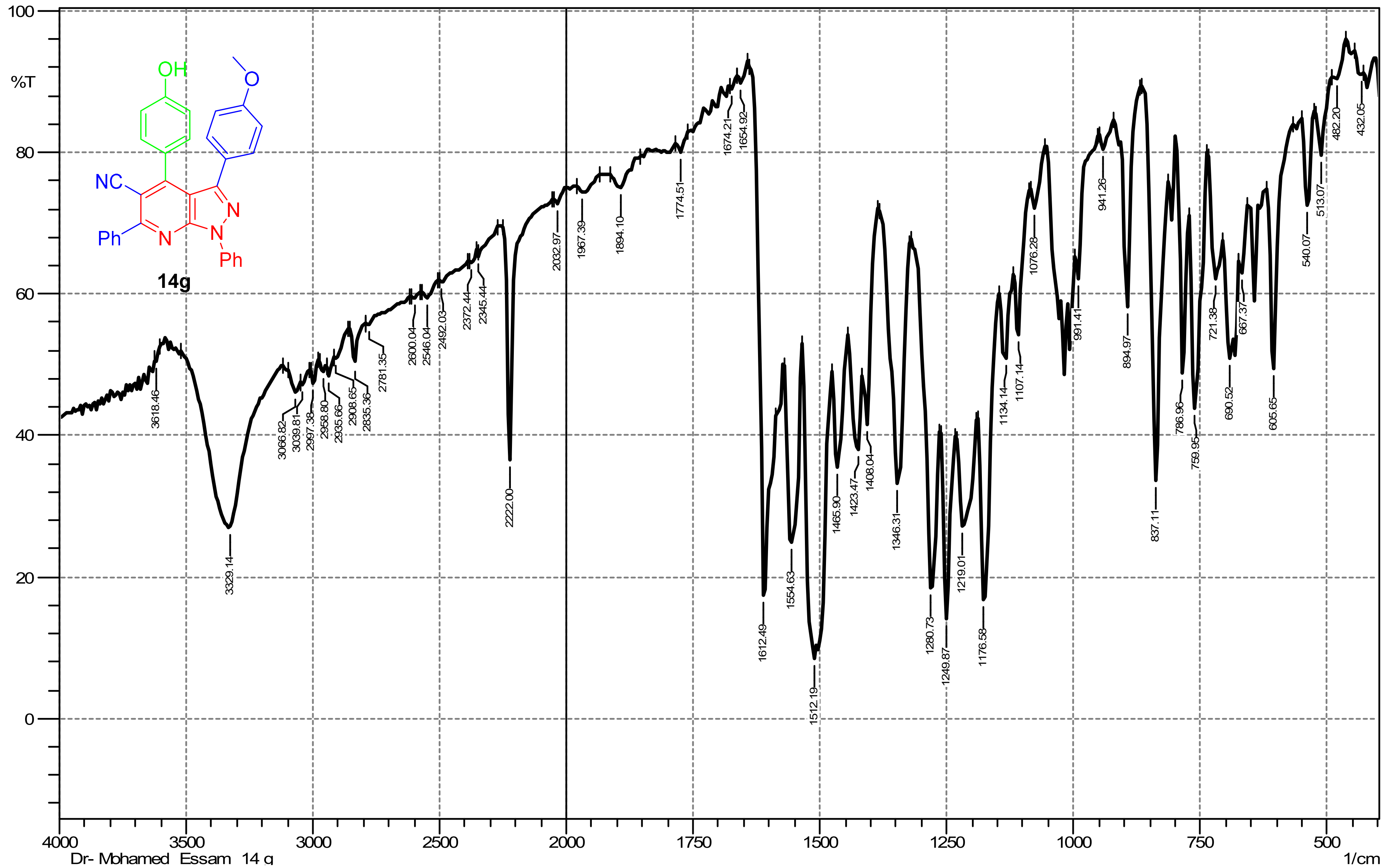

Figure S79. IR of compound **14g**

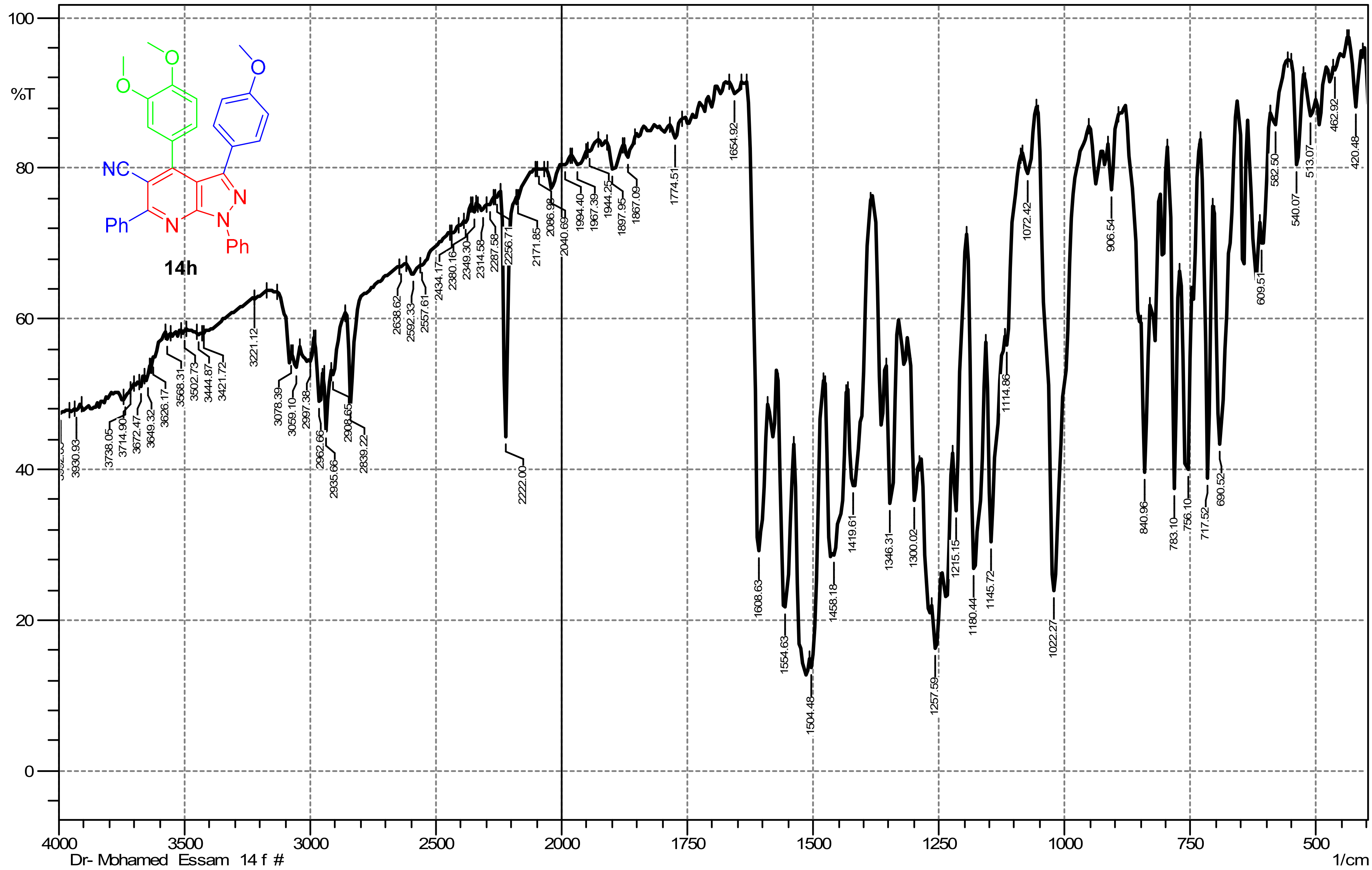

Figure S80. IR of compound **14h**

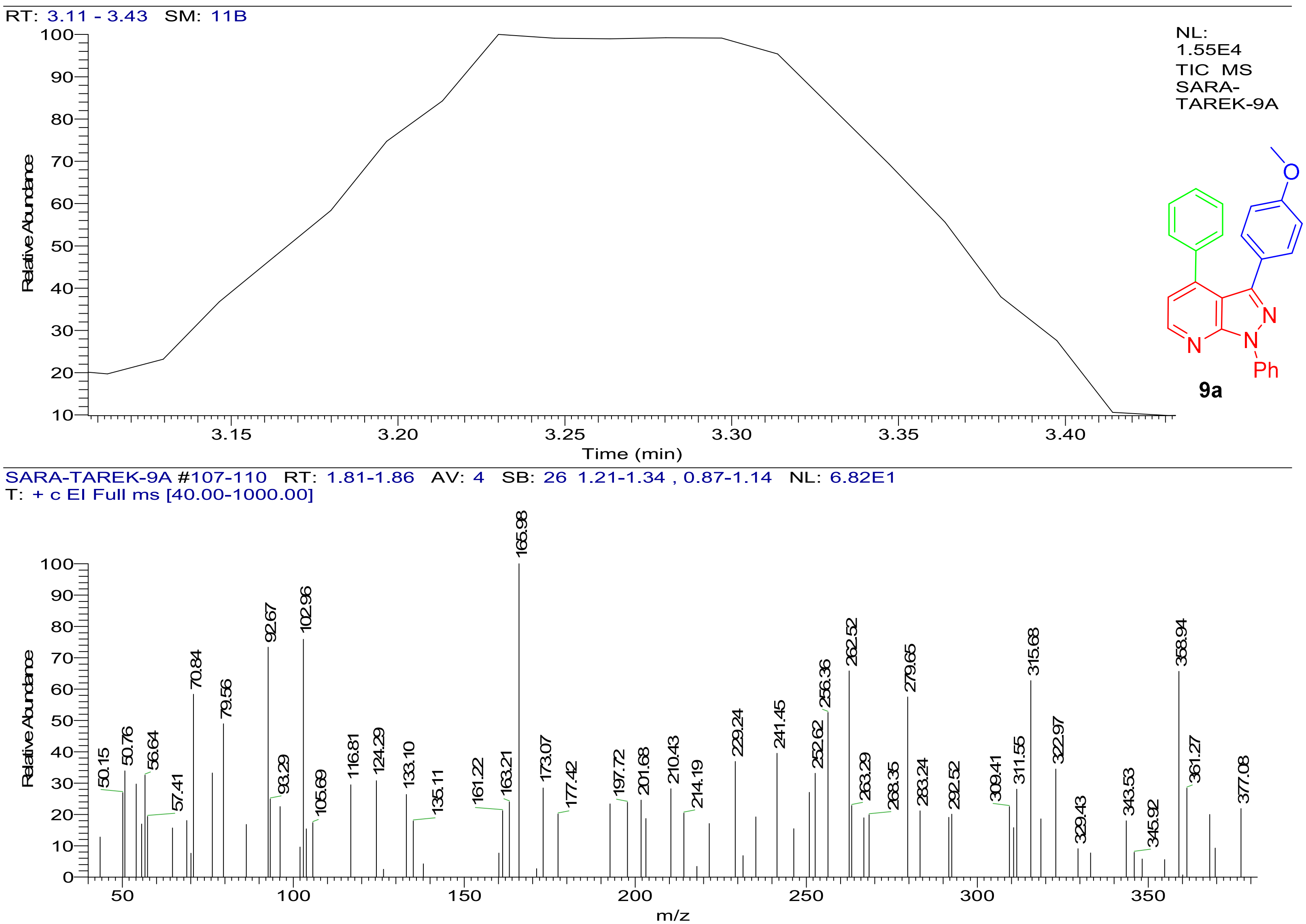

Figure S81. Mass of compound **9a**

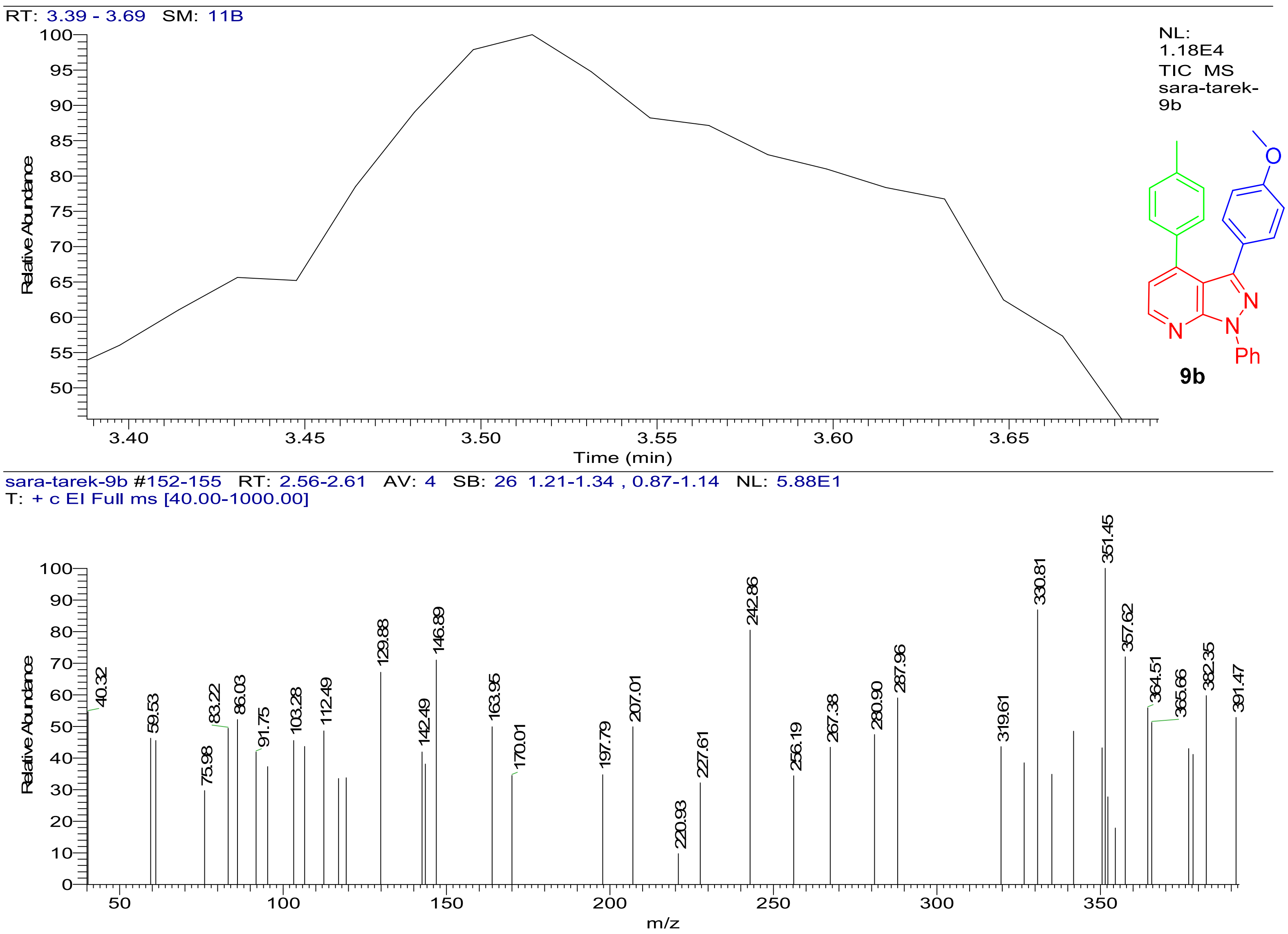

Figure S82. Mass of compound **9b**

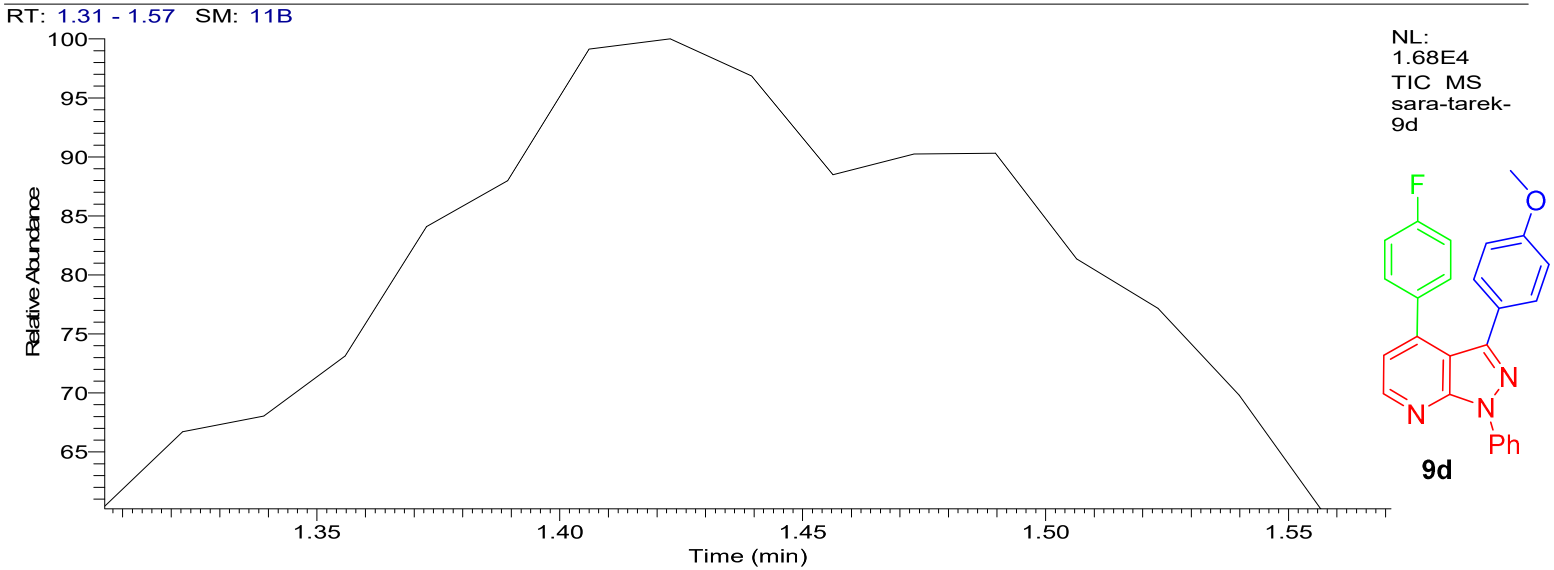

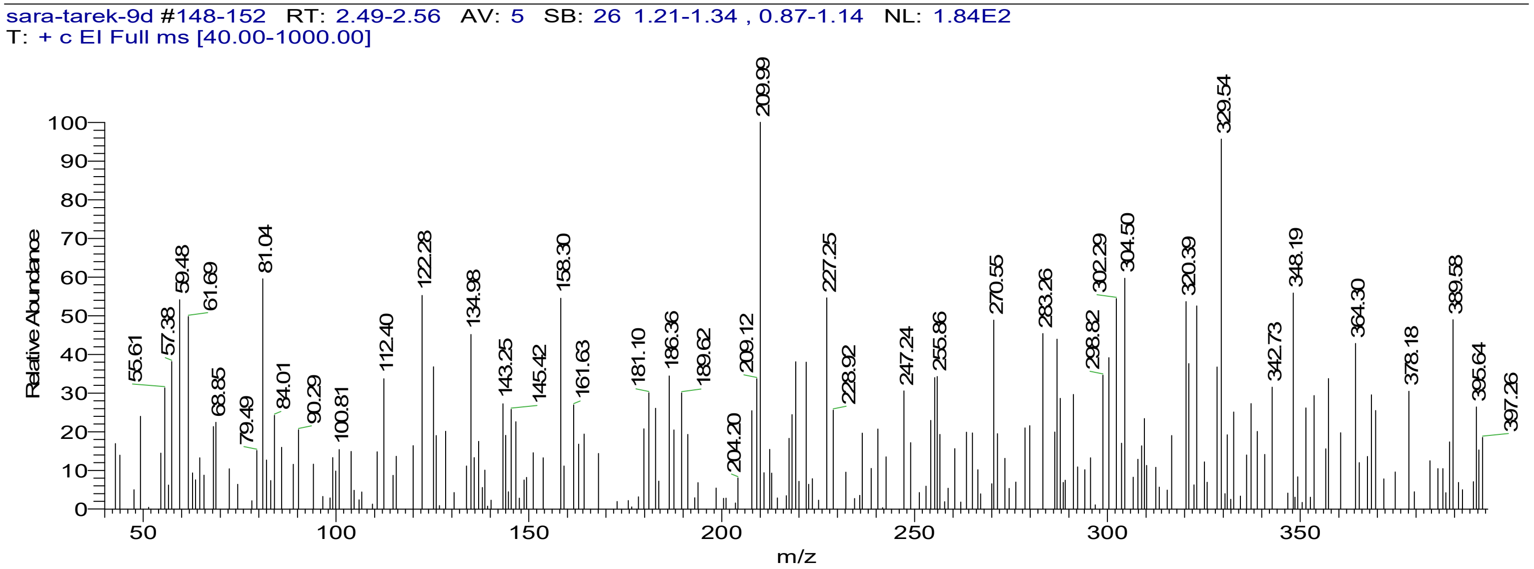

Figure S83. Mass of compound **9d**

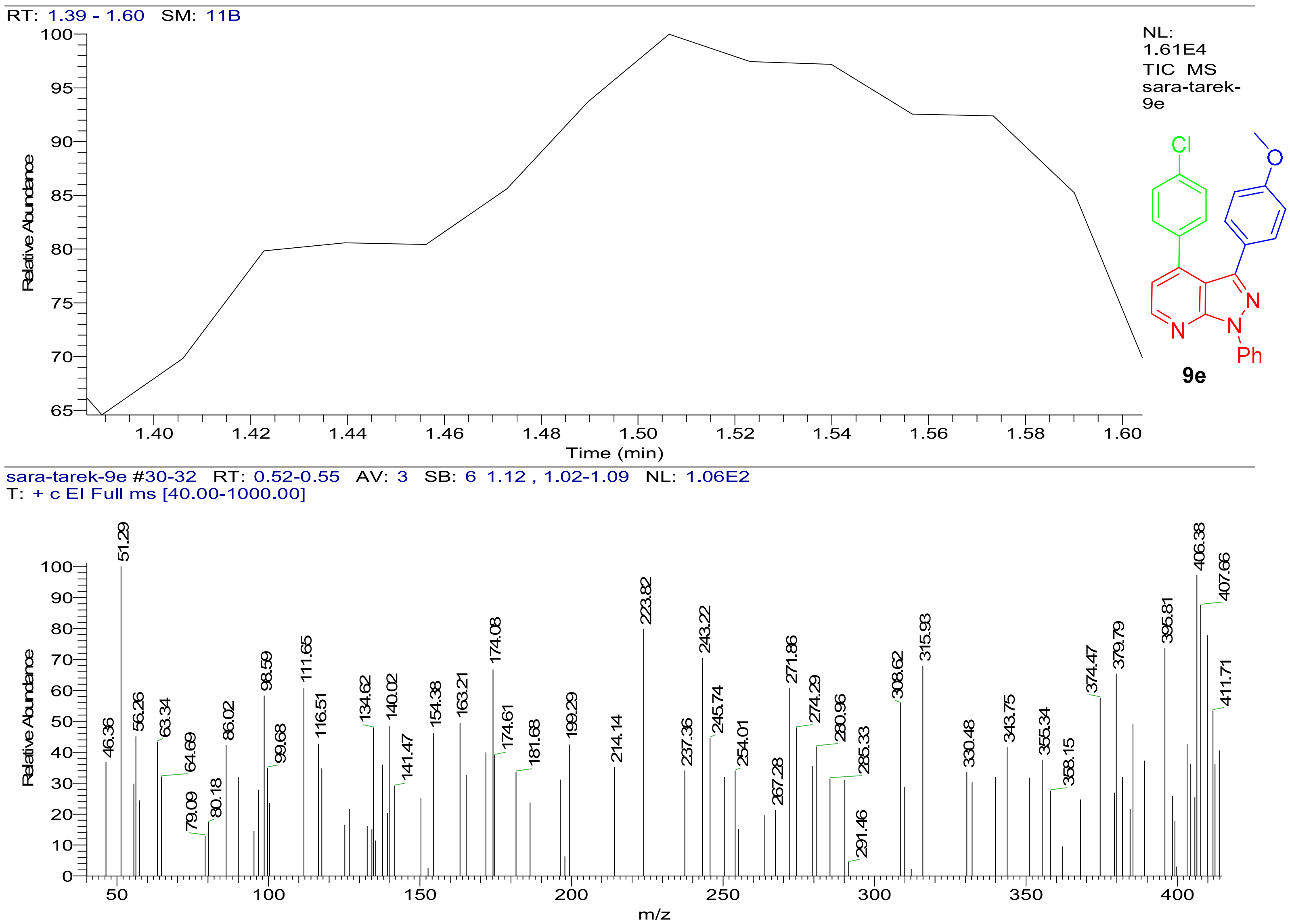

Figure S84. Mass of compound **9e**

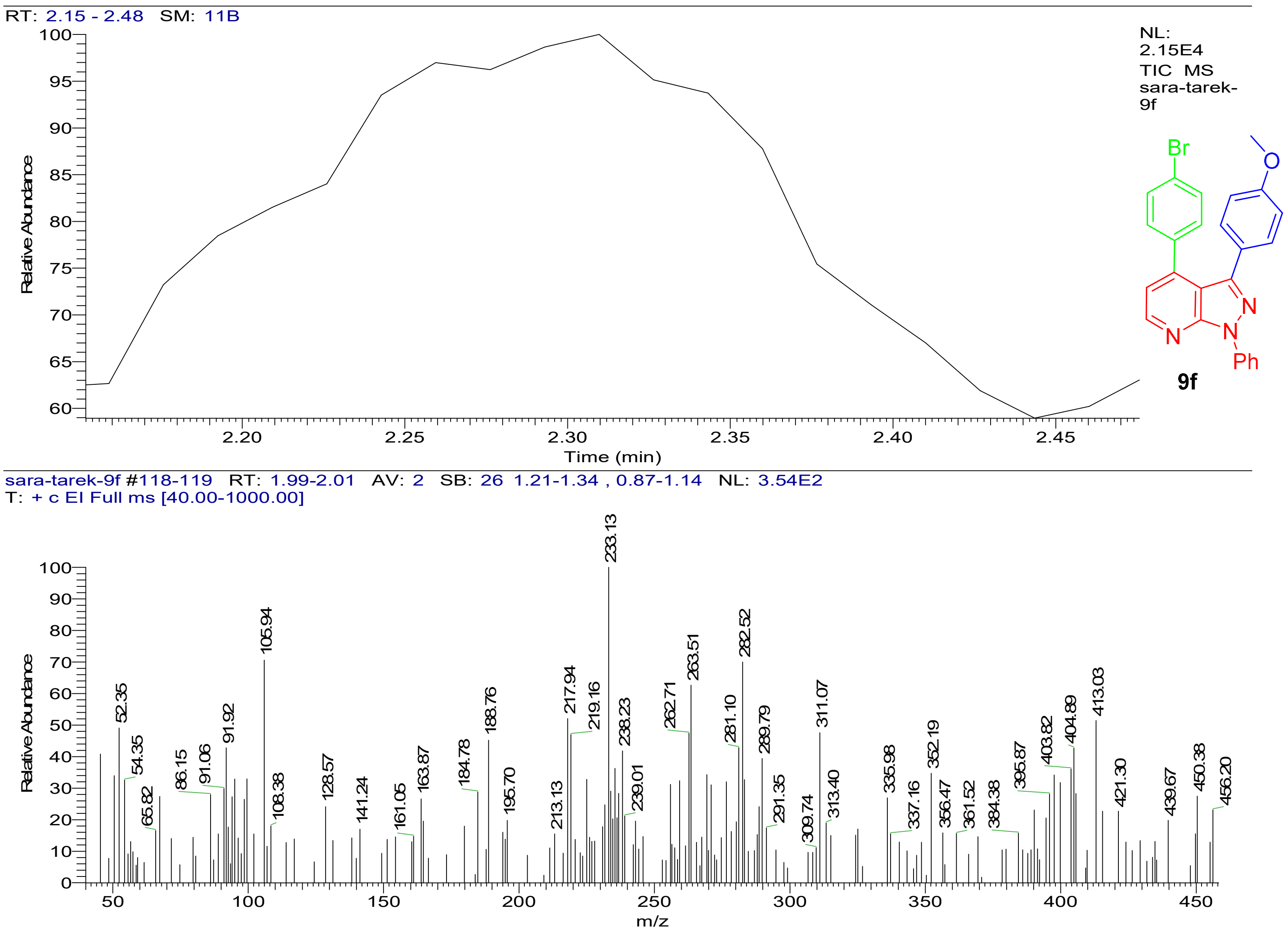

Figure S85. Mass of compound **9f**

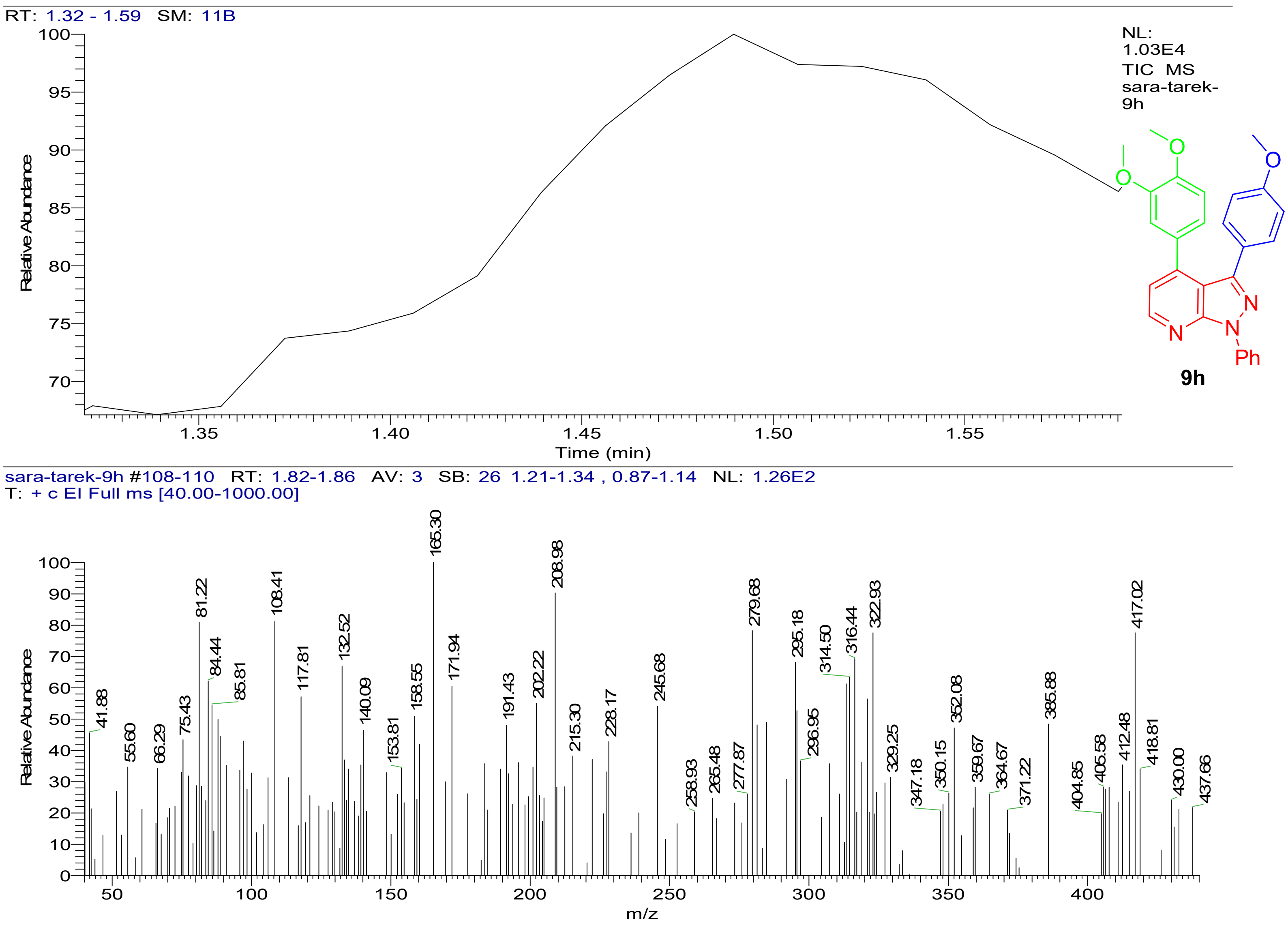

Figure S86. Mass of compound **9h**

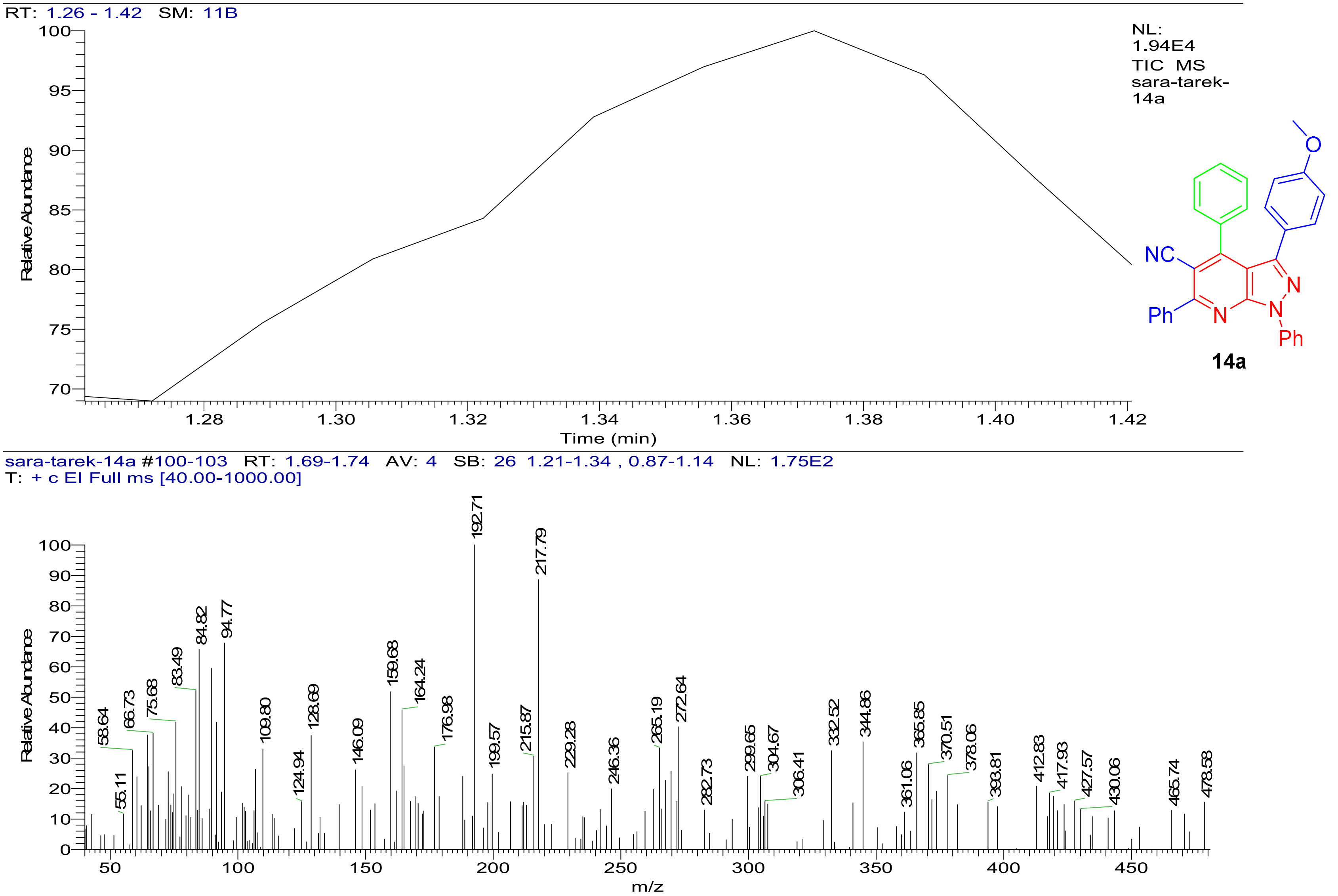

Figure S87. Mass of compound **14a**

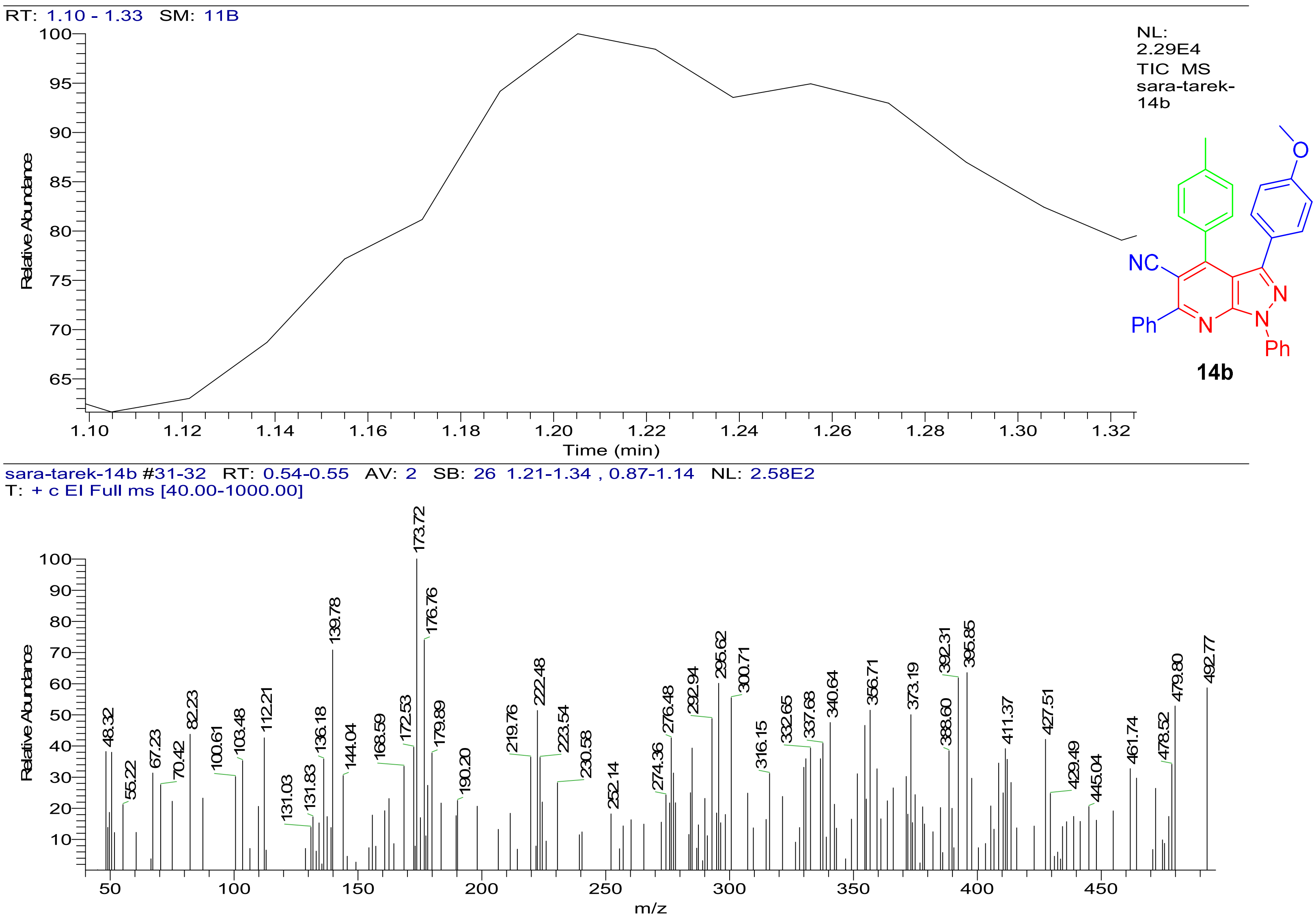

Figure S88. Mass of compound **14b**

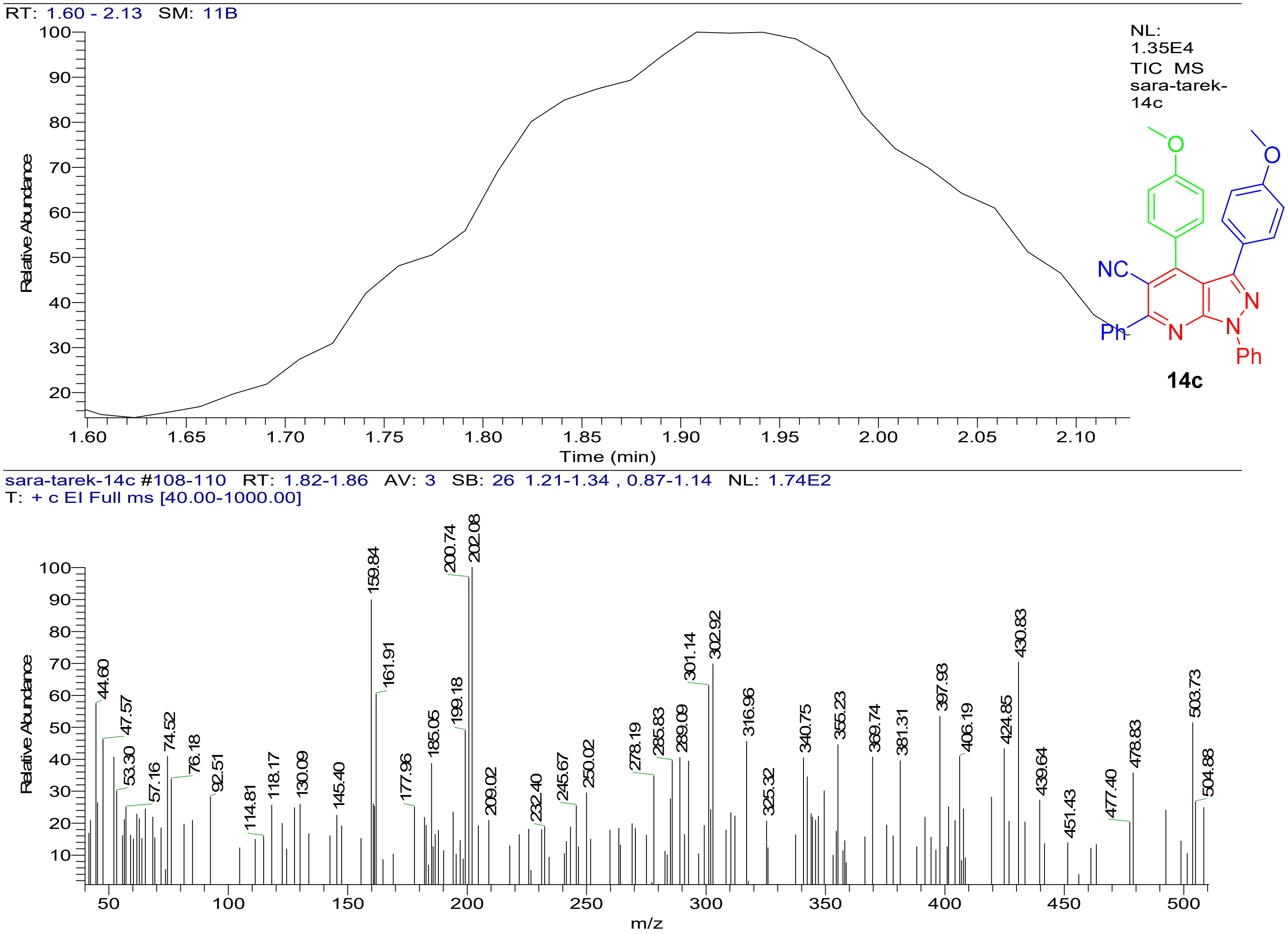

Figure S89. Mass of compound **14c**

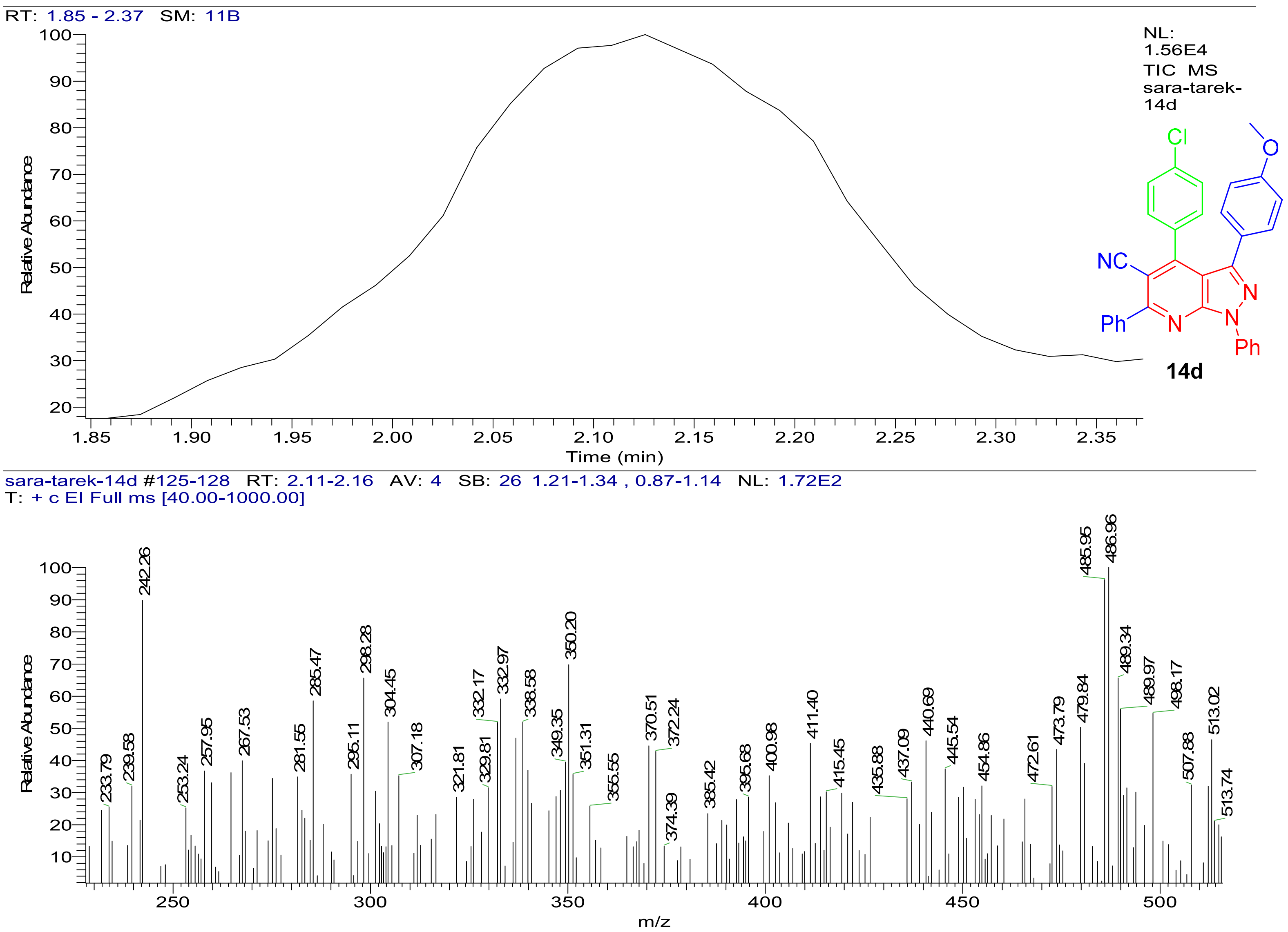

Figure S90. Mass of compound **14d**

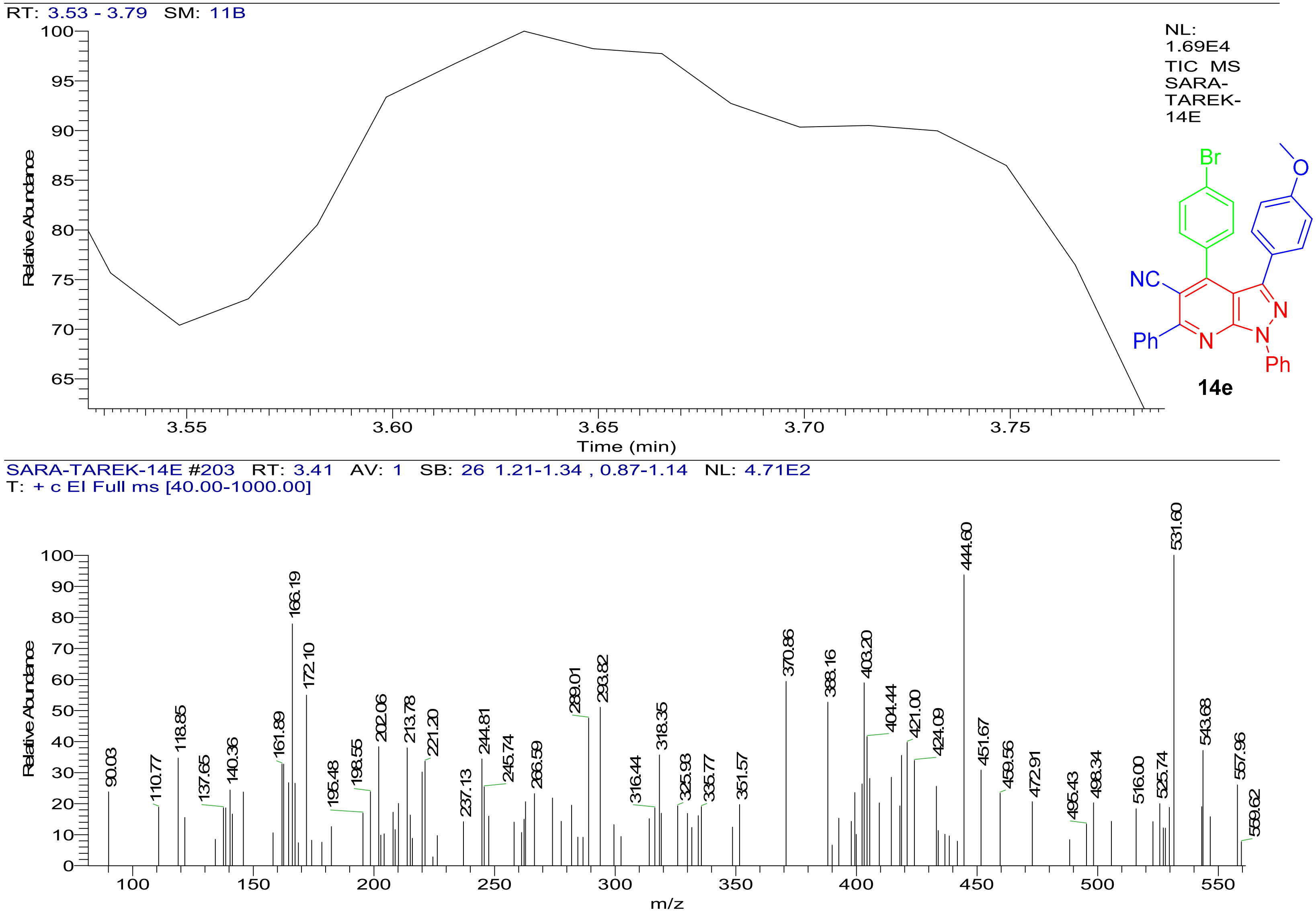

Figure S91. Mass of compound **14e**

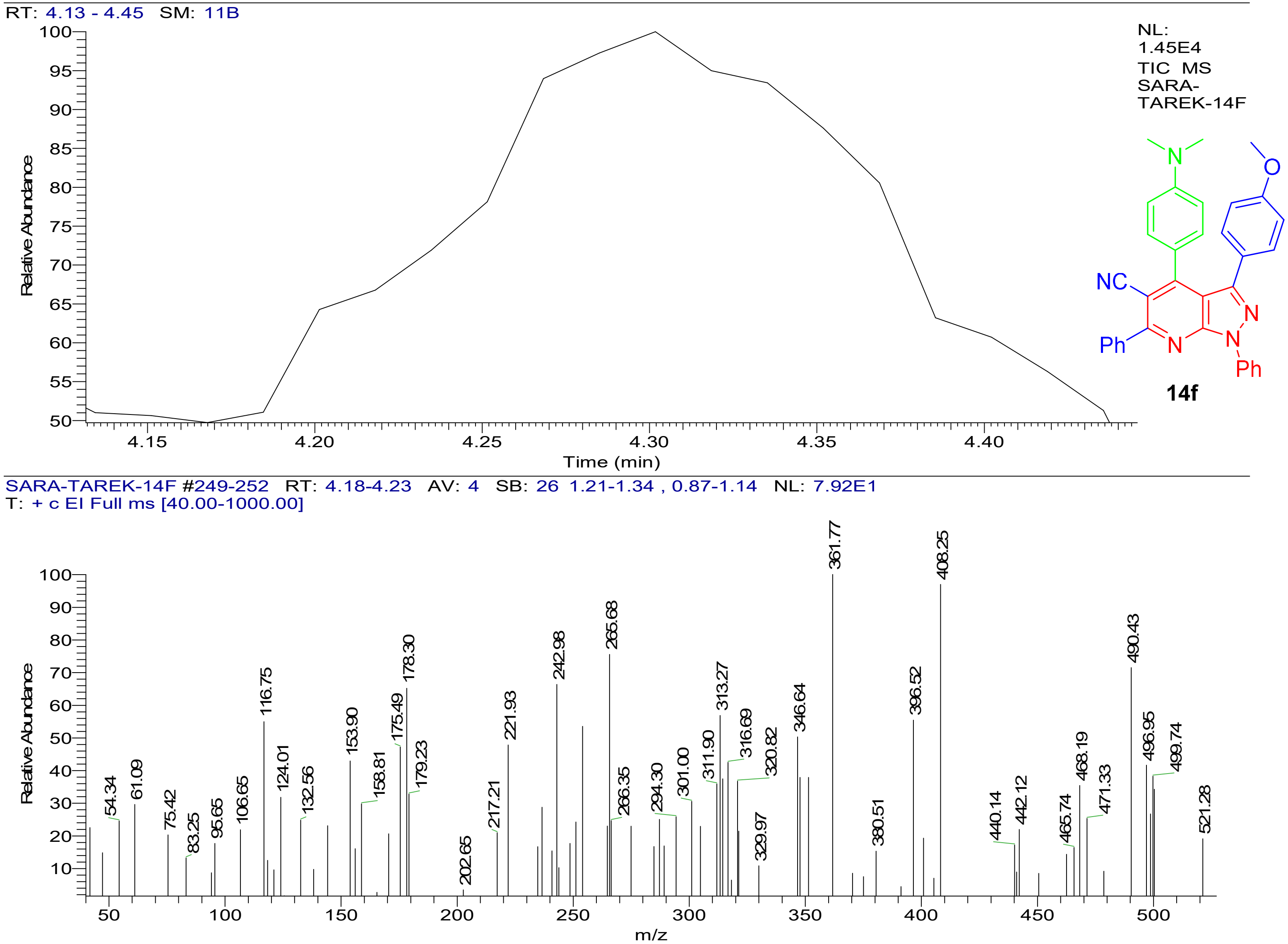

Figure S92. Mass of compound **14f**

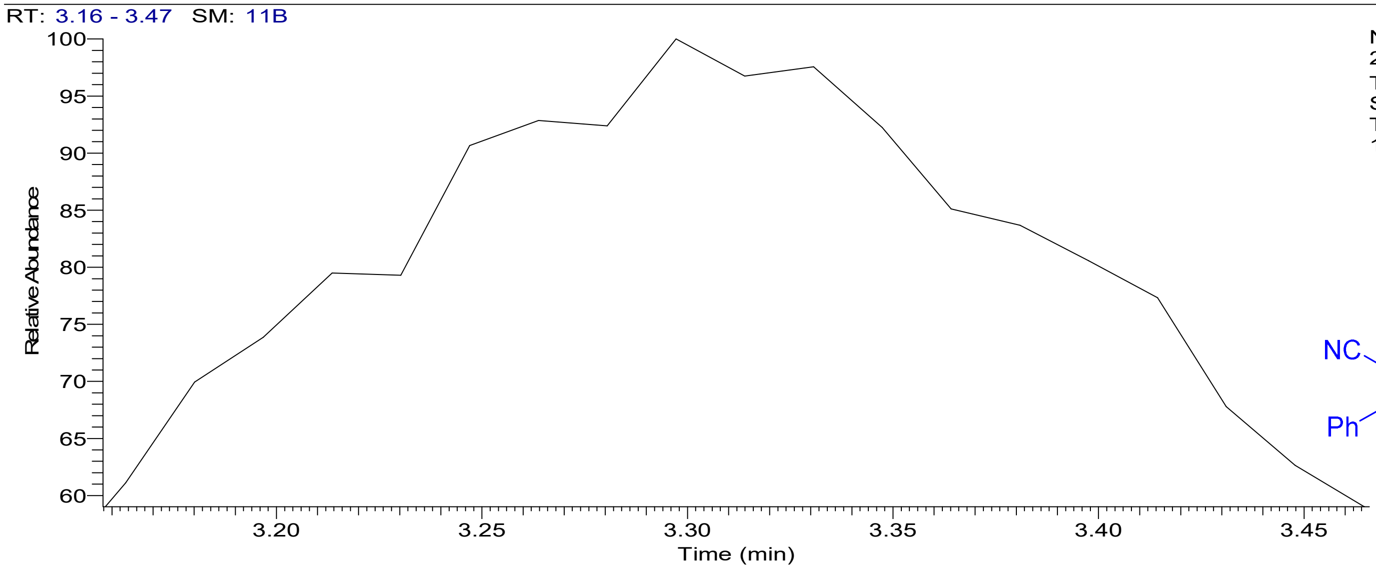

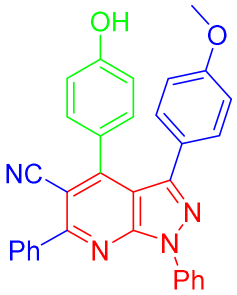

14g

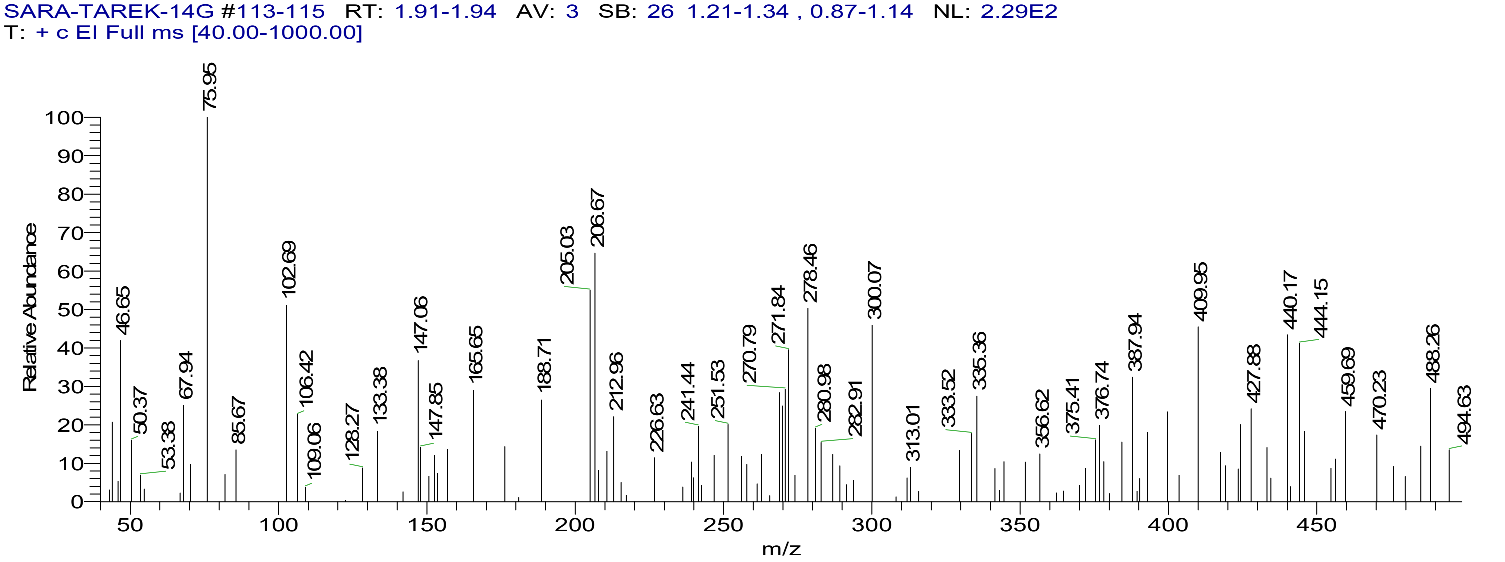

Figure S93. Mass of compound **14g**

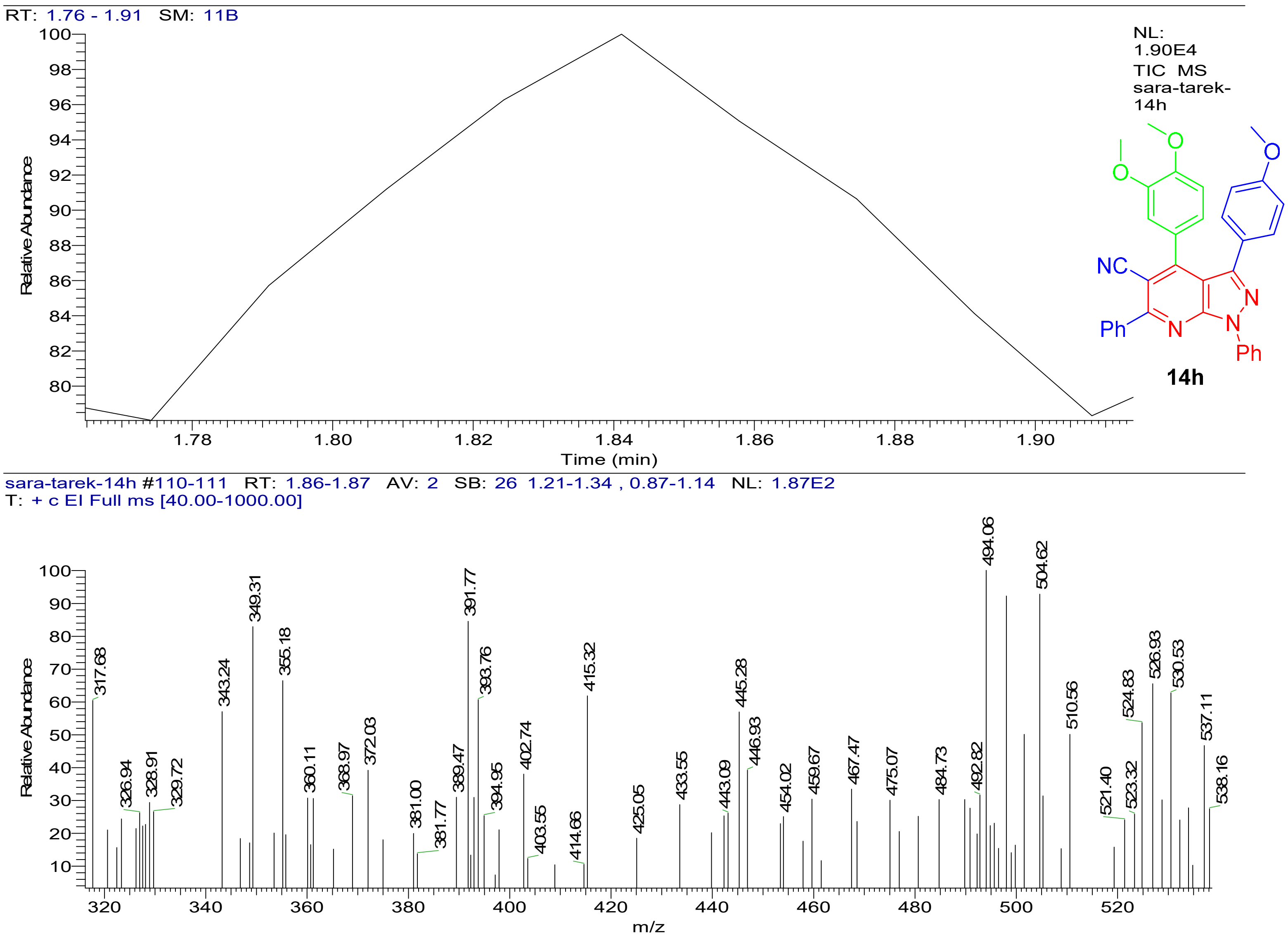

Figure S94. Mass of compound **14h**

Characterization instruments

A Stuart melting point apparatus was used for detection the melting points (°C, uncorrected).

Bruker alpha 2 ATR/FTIR spectrometer recorded all the IR spectra (KBr).

BRUKER 400 MHz NMR spectrometers recorded the NMR spectra.

Chemical shifts were recorded in parts per million (δ), and the coupling constants (J) were stated in Hertz.

TMS was used as the internal standard. ^{1}H and ^{13}C spectra were run at 400 and 100 MHz, respectively.

DMSO-d_6 is used as solvent and D_2O is used to predict the exchangeable protons.

Electron impact mass spectra were measured on Direct probe controller inlet part to single quadropole mass analyzer in THERMO SCIENTIFIC GCMS model (ISQ LT) using THERMO X-CALIBUR SOFTWARE.
